# Comparative Omics-Driven Genome Annotation Refinement: Application across Yersiniae

**DOI:** 10.1371/journal.pone.0033903

**Published:** 2012-03-27

**Authors:** Alexandra C. Schrimpe-Rutledge, Marcus B. Jones, Sadhana Chauhan, Samuel O. Purvine, James A. Sanford, Matthew E. Monroe, Heather M. Brewer, Samuel H. Payne, Charles Ansong, Bryan C. Frank, Richard D. Smith, Scott N. Peterson, Vladimir L. Motin, Joshua N. Adkins

**Affiliations:** 1 Biological Sciences Division, Pacific Northwest National Laboratory, Richland, Washington, United States of America; 2 J. Craig Venter Institute, Rockville, Maryland, United States of America; 3 University of Texas Medical Branch, Galveston, Texas, United States of America; 4 Environmental Molecular Sciences Laboratory, Pacific Northwest National Laboratory, Richland, Washington, United States of America; The Centre for Research and Technology, Hellas, Greece

## Abstract

Genome sequencing continues to be a rapidly evolving technology, yet most downstream aspects of genome annotation pipelines remain relatively stable or are even being abandoned. The annotation process is now performed almost exclusively in an automated fashion to balance the large number of sequences generated. One possible way of reducing errors inherent to automated computational annotations is to apply data from omics measurements (i.e. transcriptional and proteomic) to the un-annotated genome with a proteogenomic-based approach. Here, the concept of annotation refinement has been extended to include a comparative assessment of genomes across closely related species. Transcriptomic and proteomic data derived from highly similar pathogenic *Yersiniae* (*Y. pestis* CO92, *Y. pestis* Pestoides F, and *Y. pseudotuberculosis* PB1/+) was used to demonstrate a comprehensive comparative omic-based annotation methodology. Peptide and oligo measurements experimentally validated the expression of nearly 40% of each strain's predicted proteome and revealed the identification of 28 novel and 68 incorrect (i.e., observed frameshifts, extended start sites, and translated pseudogenes) protein-coding sequences within the three current genome annotations. Gene loss is presumed to play a major role in *Y. pestis* acquiring its niche as a virulent pathogen, thus the discovery of many translated pseudogenes, including the insertion-ablated *argD*, underscores a need for functional analyses to investigate hypotheses related to divergence. Refinements included the discovery of a seemingly essential ribosomal protein, several virulence-associated factors, a transcriptional regulator, and many hypothetical proteins that were missed during annotation.

## Introduction

The vast majority of global microarray and proteomic studies generate thousands of measurements representative of a system under a specific set of treatment or growth conditions. Interpretation of this high-throughput data is usually highly dependent on protein-coding genes being properly annotated within an organism's genome, making genome annotation a critical component of modern biological research. The rapid pace of technological improvement in genome sequencing has triggered the generation of genomic sequences at a pace previously inconceivable [1]. It took the coordination of countless people from both public and private sectors nearly a decade to sequence the first draft of the human genome [2], [3], yet Pushkarev et al. recently achieved this feat in several weeks on a single instrument with a single operator for under $50,000 [4]. Advances to the genome annotation process appear modest by comparison. Computational tools for high-throughput data are being steadily introduced [5], but many challenges still exist (e.g., a lack of gold-standard gene models for training the ‘exotic’ organisms that are the focus of many second-generation sequencing projects) [6]. Curation by knowledgeable scientists remains an essential component to complement and enhance computational work [7].

An understanding of the genome annotation process provides insight into how experimental measurements can be used to improve both the process itself and the extraction of biological knowledge. Automated annotation efforts reduce the burden of manual curation by employing algorithms to predict transcriptional and translational start and stop sites, promoter regions, protein coding regions, and untranslated regions, among other genetic features [8]. However, even for prokaryotes which are inherently less complex than eukaryotes in terms of gene structure, the reliability of computational predictions remains imperfect. For example, de Souza and colleagues analyzed a *Mycobacterium tuberculosis* culture filtrate sample to examine discrepancies between two established gene prediction methods. Even with the reduced complexity of a culture filtrate (∼10% of the predicted *M. tuberculosis* proteome), nearly 2% of the identified peptides were specific to only one of the two automated annotations suggesting that a substantial number of missed genes are present in the genome-wide annotation [9]. Bakke et al. compared annotations of the entire expressed proteome of the archean *Halorhabdus utahensis* using three different gene-calling platforms [10]. The authors found that less than half of the nearly 3000 predicted protein-coding regions were consistent across the automatic annotations, supporting the speculation that significant deviations in gene calling efforts would be revealed in global-scale analyses.

Evidence shows that automated annotations are subject to error, yet financial constraints lead to most gene predictions being directly incorporated into databases. Libraries of potential coding sequences with varying levels of confidence are generated. These libraries directly influence the quality of high-throughput biological studies. For example, microarray chip probe selections are typically chosen from libraries of predicted protein coding genes. The absence of a probe targeting a specific nucleotide sequence does not mean that complementary mRNA is not expressed; it simply reflects the inherent bias of the microarray design. An example of error propagation associated with proteomics relates to databases comprised of candidate proteins, a crux of many modern experimental proteomic studies. The majority of peptide-centric proteomic analyses occur by matching, not direct interpretation, of spectra [11]. Search tools rely on peptide-matching algorithms to compare experimental MS/MS spectra to *in-silico* peptide spectra generated from protein databases. Several of the unmatched high-quality spectra present in bottom-up proteomic analyses may be explained by errors in protein predictions from the genome. Importantly, the same omics datasets that suffer from incorrect or absent gene assignments can in fact help provide experimental revisions to existing genome annotations.

The practice of utilizing MS/MS data for genome annotation refinement was documented as early as 1995 when Yates et al. introduced the concept of searching a six frame translation of a nucleotide sequence with tandem MS data [12]. Annotations of a number of prokaryotic and eukaryotic systems have since been investigated using this approach, commonly referred to as proteogenomics, resulting in the validation of predicted genes and elucidation of annotation errors. Gupta and colleagues identified 8 novel genes, redefined boundaries for 30 genes, and observed expression of 13 pseudogenes in *Shewanella oneidensis* MR-1 [13]. Similarly, Castellana et al. estimated that 13% of the *Arabidopsis thaliana* protein coding genes were incorrect or absent from the genome annotation [14].

Comparative proteomics can be applied to extrapolate findings of peptide identifications from a single species to orthologs based on evolutionary constraints [15], [16], [17]. This approach allows researchers to either salvage or increase the confidence of evidence used for annotation refinement. This is extremely important for coding sequences that are expressed at low levels, encode short products, or exhibit poor detection efficiency. Transcriptional knowledge can also be used for annotation refinement. High density tiling arrays and RNA sequencing aid in the discovery of novel genetic features, and while their data alone does not confirm protein expression, detection provides supporting evidence. Complementary evidence is indispensable in instances when proteins are inferred by single peptide identifications, a situation that is typically insufficient for a protein's identification. The greatest potential for annotation refinement should come from simultaneously merging transcriptomic and proteomic data [18] with a comparative genomic approach.

We undertook a comparative omics approach to simultaneously refine the genome annotations of three highly orthologous *Yersinia* strains. The work presented herein extends typical proteogenomic methodology by incorporating not only peptide measurements, but also experimental oligo data and sequence comparisons across strains. *Yersinia* was chosen as a model in part due to the high genetic similarity between species possessing dissimilar characteristics [19], [20]. The examined strains were annotated by different groups of researchers using different methods over several years [21], [22]; as such, variations in both sensitivity and specificity of annotation are expected. *Yersinia* comprises three species pathogenic to humans: *Y. enterocolitica*, *Y. pseudotuberculosis*, and *Y. pestis*. The latter two species diverged most recently, and while their genomes are closely related, the bacteria exhibit markedly different modes of transmission and pathogenecities [23]. *Y. pseudotuberculosis* causes non-fatal gastrointestinal disease, and *Y. pestis* is the causative agent of plague. The data described here characterize *Y. pseudotuberculosis* PB1/+ and two *Y. pestis* strains, CO92 and Pestoides F. The *Y. pestis* strains represent “epidemic” (CO92) and “non-epidemic” (Pestoides F) isolates, which differ in their biochemical properties, virulence to different animal species, and rearrangements of the genome mediated by the insertion sequences [24], [25]. The genomes of the examined strains have been sequenced [21], [22] and are available at NCBI (www.ncbi.nlm.nih.gov). Each strain is currently annotated with approximately 4000 protein coding genes (Table 1). Our results validate predicted protein-coding genes and revise the current genome annotations through identification of 96 unannotated or erroneous protein coding regions among the three strains. The refined genome annotations are immediately useful for the entire *Yersiniae* research community, and the comparative omics-based approach is applicable to other organisms possessing similarity between strains or species.

**Table 1:** Omics-driven genome annotation summary.

	*Y. pestis* CO92 (YPO)	*Y. pestis P*estoides F (YPDSF)	*Y. pseudotuberculosis PB1/+* (YPTS)
Sequencing Center/year	Sanger Institute/2001	JGI/2007	JGI/2008
Predicted pseudo genes	123	84	15
Protein-coding genes (total)	4066	4068	4237
Protein-coding genes (ortholog in ≥1 alternate strain)	3866	3895	3695
Summary of protein identification validations			
Detected proteins (≥2 peptides)	1682 (1641)	1773 (1751)	1603 (1550)
Detected proteins (single peptide)	380 (277)	392 (271)	398 (303)
Summary of genome annotation refinements			
Novel genes	8 (4)	18 (16)	2 (1)
Translated “pseudo genes”	40 (36)	16 (15)	1 (0)
Extended start sites	3 (2)	3 (3)	2 (2)
Frameshifts	0	3 (3)	0

The total number of open reading frames with evidence (as described in Materials and Methods) are represented. Numbers within parentheses refer to the subset of genes that have orthologs with experimental evidence in at least one of the alternate strains.

## Results and Discussion

### Comparative *Yersinia* omics-based annotation

**Figure 1:**
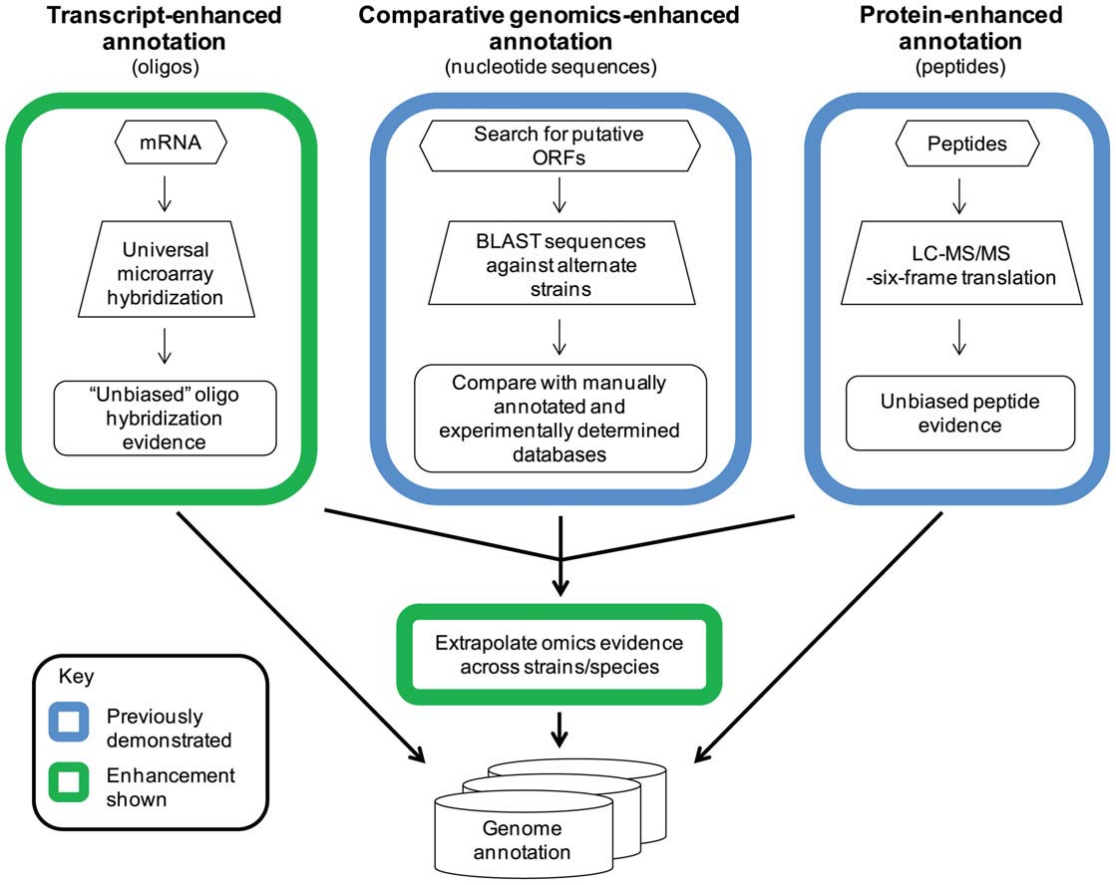
Schematic showing the comparative omics-based genome annotation workflow employed for annotation refinement. Transcriptomic data generated from an unbiased universal *Yersinia* microarray and peptide data matched to a 6-frame genome translation were layered on existing genome annotations to validate predicted protein coding sequences and identify annotation anomalies. This evidence can be used independently or combined with putative protein identifications derived from a comparative genomics approach for genome annotation refinement.

A typical proteogenomic workflow integrates experimental peptide evidence and computationally-predicted protein-coding sequences. The approach presented herein (Figure 1) extends that approach by also including orthogonal genome-wide measurements (i.e., microarray probe hybridization). This comparative omics approach was used to investigate current annotations for three highly similar *Yersinia* strains: *Y. pestis* CO92 (YPO), *Y. pestis* Pestoides F (YPDSF), and *Y. pseudotuberculosis* PB1/+ (YPTS). Seventy-five matched RNA and protein samples from each strain were collected across a range of thermal and temporal conditions (26 or 37°C for 1, 2, 4, or 8 h) to maximize transcriptome and proteome coverage. Analyses focused on validating predicted genome annotations and discovering experimentally-supported annotation errors. Existing genus level *Yersinia* annotations were also compared across the examined strains to extrapolate putative annotation errors. Experimental omics data were combined with these findings to predict sequences that may exhibit protein expression in the absence of experimental evidence.

**Figure 2:**
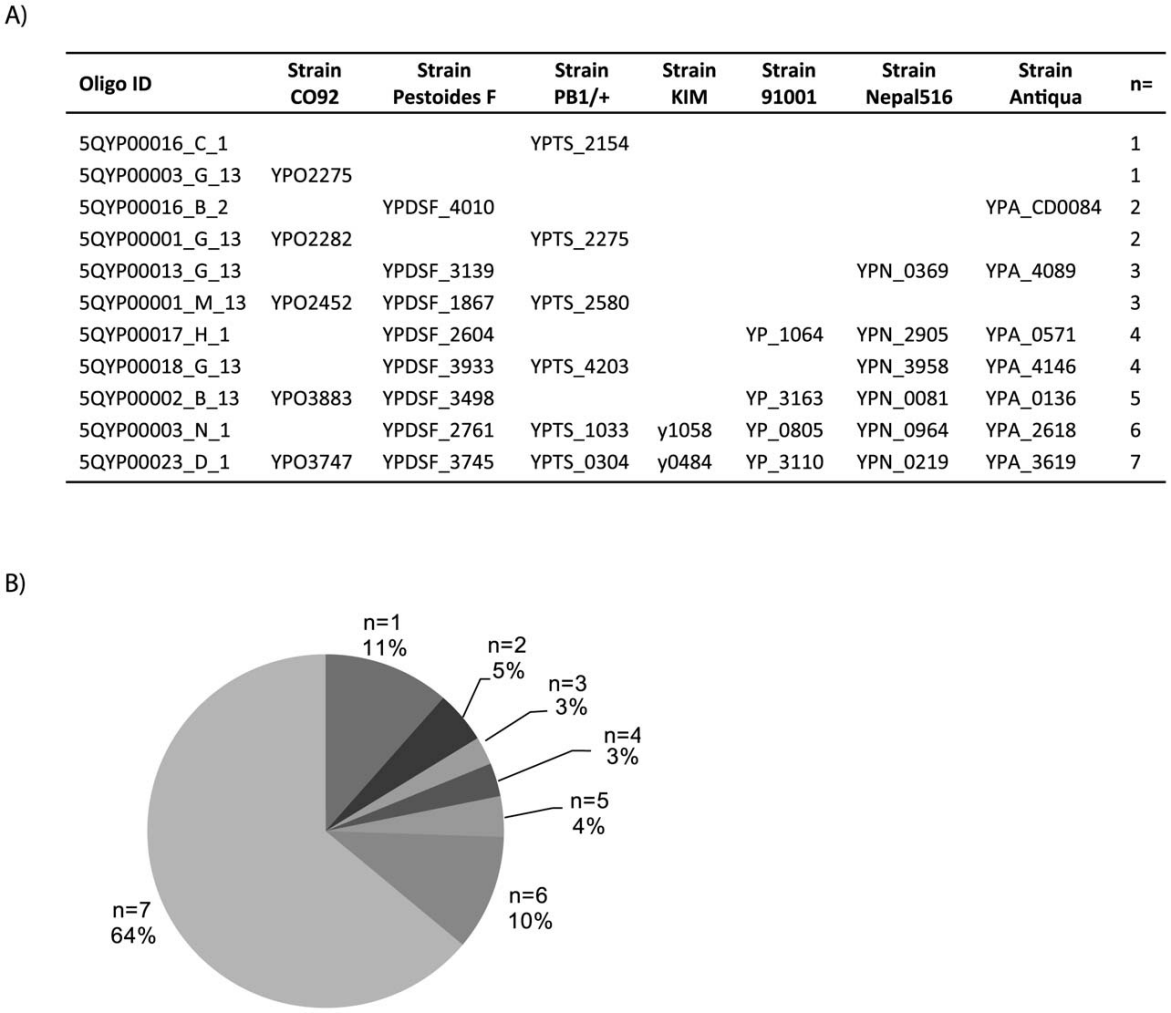
Distribution of oligos for universal *Yersinia* microarray. A universal array was designed to represent genes from seven different *Yersinia* strains on a single chip. A) A representative sample of oligos and their purported mapping to genes from each of the seven *Yersinia* strains represented on the array is shown. The number of strains (n = x) for which a given oligo corresponds to an annotated protein coding sequence is indicated, illustrating a subset of the possible combinations of unique or shared orthologs at the oligo level. B) The distribution of oligos based on existing annotations is provided; oligos were predicted to be either unique to any one of the seven strains (n = 1) or shared by multiple strains (n>1).

A universal microarray was used to obtain genome-wide expression measurements. Unlike a traditional microarray that targets annotated genes for a single strain, this universal array incorporated 7641 probes designed against seven sequenced *Yersinia* strains on a single chip. As illustrated in Figure 2, 89% of the probes were complementary to genes present in multiple, if not all, represented *Yersinia* strains. The remaining probes targeted genes annotated as purportedly unique to one of the *Yersinia* strains. Comprehensive *Yersinia* genus level gene expression measurements were made since each strain's sample was individually hybridized against the universal array. More specifically, the expression of a nucleotide sequence (represented by a probe targeted against at least one of the seven *Yersinia* genomes) was examined for individual strains regardless of whether or not a protein-coding sequence was currently annotated for the strain being analyzed. Results were assessed for all probes present on the universal array for all three strains.

In parallel to the transcriptional study, bottom-up proteomics was used to identify peptides for inference of protein expression. Approximately 1.6 million MS/MS spectra per strain were searched against a stop-stop FASTA file comprised of a six-frame translation (minimum length of 30 amino acids) of each corresponding *Yersinia* genome. Data were filtered to <0.4% false-discovery rate using a reversed sequence decoy strategy [26]. A modest number of redundant peptides (i.e., peptides that map to multiple genomic loci) were excluded from the workflow to remove potential ambiguity. Post-filtering, nearly 20,000 peptides per strain were mapped to each respective genome, corresponding to the expression of 41%, 44%, and 38% of YPO, YPDSF, and YPTS proteins (minimum of two peptides/protein), respectively. Table 1 summarizes these identifications and illustrates the high level of orthology between strains. Additionally, genome annotation refinements for both annotated and unannotated open reading frames (ORFs) are reported, indicating the utility of the comparative omics-based annotation presented herein.

### Identification of annotation anomalies exhibiting a minimum of two peptides per ORF

**Figure 3:**
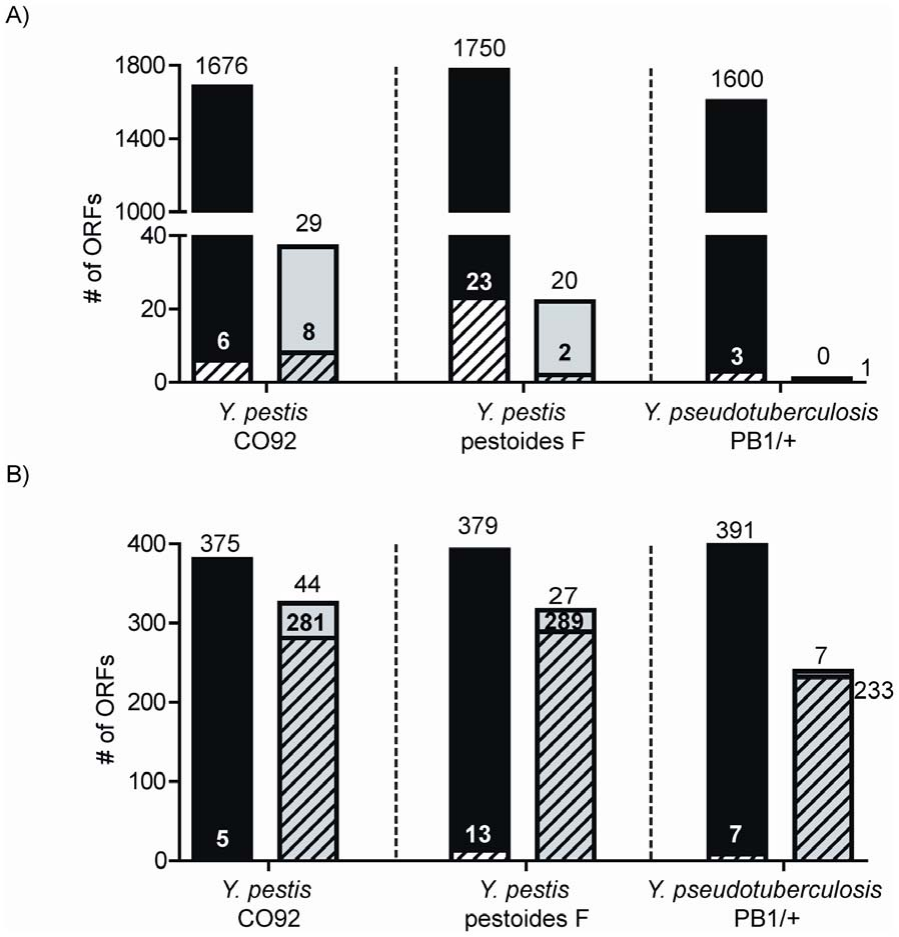
Categorization of experimental evidence. Preliminary analysis shows the distribution of peptide and oligo evidence across annotated open reading frames (ORFs) (i.e., predicted proteins) in black and unannotated ORFs in grey. Solid regions indicate ORFS with complementary oligo and peptide evidence and hashed regions show ORFs with only peptide evidence. A) shows ORFs exhibiting two or more unique non-redundant peptides and B) shows ORFs exhibiting a single unique non-redundant peptide.

Confident gene expression and peptide identification measurements, along with predicted protein coding genes, were independently layered on genome sequences for each *Yersinia* strain's chromosome and corresponding plasmid(s). Experimental evidence observed within a protein-coding region of a gene was considered validation of an existing annotation, and evidence outside of annotated ORFs highlighted regions suggestive of missed/incorrect annotation calls. Figure 3 shows the distribution of experimental evidence across annotated ORFs (i.e., predicted proteins) and unannotated ORFs (i.e., putative proteins not associated with any existing transcript/protein annotations). Using peptide measurements as primary evidence of protein expression, it was determined that 1682, 1773, and 1603 predicted protein-coding sequences were expressed with a minimum of two unique peptide identifications for YPO, YPDSF, and YPTS, respectively. Notably, greater than 98% of these protein identifications also had complementary oligo hybridization evidence as support. Kolker et al. generated both protein and gene expression data for a functional annotation study in *S. oneidensis* [27]. Gene expression was observed for 2082 of 2252 predicted proteins (93%), comparable to the overlap represented in this work. In contrast to Kolker's work, the microarrays utilized for the current studies possess a unique advantage important for an omics-based refinement study, i.e., the ‘unbiased’ nature of oligos present on the universal chip aid in the discovery of unannotated sequence expression. Figure 3a shows the identification of currently unannotated ORFs exhibiting experimental evidence; 37 (29), 22 (20), and 1 (0) unannotated ORFs mapped at least two unique peptides (and a minimum of one oligo) for YPO, YPDSF, and YPTS, respectively, warranting further investigation.

### Proteins identified by single peptide identifications

It has been shown that random peptides exhibit a peptide-to-protein distribution favoring single peptide correlations [28]; however many single peptide observations are in fact authentic, confounding the choice to include or exclude these identifications. Nearly universally, single peptides are discarded for proteomics applications due to the high probability of false-positives for proteins identified by a single unique peptide relative to proteins with multiple unique peptides using a given set of filtering criteria. These single peptide identifications (singlets) are often pejoratively referred to as one-hit wonders. Unfortunately, removal of singlets by increasing the stringency of filter criteria results in the loss of a large number of possible true-positive protein identifications [29], [30]. Omics-based annotation can benefit from retaining single peptide identifications as the main goal is to provide layers of experimental evidence of gene expression. Figure 3b illustrates 380, 392, and 398 annotated proteins that were identified by evidence of a single peptide. Using the stringent filters chosen for this study, >96.7% of the proteins corresponding to singlets also had evidence of at least one oligo with hybridization. These findings lend increased support to the validity of proteins inferred from single peptide identifications in this study [29] and prompted examination of a group of 44, 27, and 7 unannotated ORFs eliciting both singlets and oligo evidence for YPO, YPDSF, and YPTS, respectively.

### Orthologous and single peptide identifications

Orthology has been employed for global applications including the identification of core genomes and proteomes [31], [32], [33]. Proteomic profiles of diverse environmental and pathogenic bacteria [31] revealed the expression of a core genome, indicating conservation amid diverse speciation events. Bottom-up proteomic data have been measured in five model eukaryotic species and quantitative protein abundances were found to be significantly correlated for orthologs across a conserved core proteome [33]. Even for homologous proteins with varying sequences, individual peptide biases averaged out at the protein level. Relevant to the present work, core proteins with similar sequences are expected to have comparable peptide abundances given an inherent peptide “MS-detectability” [33]. While proteogenomics approaches are qualitative in nature, the above concepts support the notion that an orthologous peptide in one *Yersinia* strain has a high probability of being detected in the alternate strains in the current experiments. The caveat may be for regulated proteins where differential protein abundance is sample dependent. For pathogenic bacteria such as *Yersinia*, qualitative genomic divergence and quantitative expression profiles contribute to a switch from a less-pathogenic lifestyle (e.g., *Y. pseudotuberculosis*) to one of high virulence (e.g., *Y. pestis*).

Ortho-proteogenomics and comparative proteogenomics, two branches of proteogenomic pipelines, demonstrate the utility of evolutionary constraints for refinements of genome annotation. The ortho-proteogenomic approach has been demonstrated for the *Mycobacterium* genus using *M. smegmatis* as the reference sample. Genome refinements were made for the reference genome and knowledge was propagated to other *Mycobacterium* species [15]. This approach is invaluable, however care must be taken when extrapolating annotations to homologous genomes [34]. Comparative proteogenomics is a more definitive approach to refining highly similar genomes. This method incorporates parallel experimental peptide data from multiple orthologous proteomes with their respective genome sequences as performed for *Shewenella* [16].

Orthologous singlet peptides (i.e., related peptides found across multiple species) can be used to infer protein translation [16]. In this study, this group of putative annotation errors includes unannotated ORFs that exhibit singlet peptide evidence but lack oligo hybridization data. Identical singlet peptides are valued; however, identification is more reliable when peptides are correlated (i.e., differ in sequence in at least one position). Correlated peptides are related through sequence but either possess mutations or modifications at a given amino acid residue or comprise different lengths (ladder sequences). This results in different b- and y-ion fragments and thus differing mass spectra for matching. The likelihood of two independent false-positives for correlated peptides in conserved species is extremely low. Notably, 245 (63) of the 705 YPO singlet peptides, 246 (55) of the 708 YPDSF singlet peptides, and 234 (61) of the 638 YPTS singlet peptides are identical (correlated) to a peptide found in at least one of the other *Yersinia* strains examined in this study (File S1).

Based on the preliminary analysis shown in Figure 3, the following criteria were established to objectively generate a conservative list of potential annotation errors for examination: 1) the presence of a non-redundant peptide and either an additional non-redundant peptide or a hybridized oligo or 2) a non-redundant peptide that has an orthologous peptide observed in at least one alternate strain. Using these criteria, potential protein sequences from unannotated regions were aligned to other *Yersinia* species using BLASTp (NCBI). The presence of a highly related structural homolog to an unannotated protein sequence was considered evidence of a novel (i.e., missing) gene, incorrect protein start site, or frameshift. Additionally, expression of annotated pseudogenes was noted. This suggests that regions of many of these ‘dead’ genes are in fact expressed, albeit not necessarily functional, in agreement with other reports [17], [35]. A summary of these findings is shown in Table 1, and detailed information for all annotation refinements including peptide and oligo evidence, proposed gene boundaries, proposed protein sequence, and corresponding ortholog data is provided in File S2.

### Identification of novel genes

**Figure 4:**
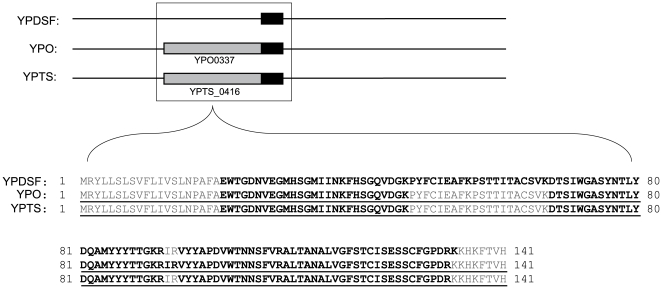
Representative identification of a novel gene. In the upper frame, gene level alignments are shown for the three strains examined in this study. Predicted protein coding genes are shown in grey and oligo evidence is shown in black. Sequence alignments are shown in the lower frame. Annotated protein sequences are underlined, and experimental peptide evidence is shown in black text. For YPDSF, this region reveals substantial oligo and peptide evidence in an unannotated region indicating a novel gene.

The first category of annotation anomalies identified represents ‘novel’ genes that were missed during the annotation process. These errors reveal experimental evidence in intergenic regions that currently lack annotation. The protein coding sequences corresponding to these novel genes are annotated in at least one other *Yersinia* strain (determined by BLASTp) but are missing in their entirety in the strain containing the error. This analysis found evidence indicative of 8, 18, and 2 novel genes in YPO, YPDSF, and YPTS, respectively (for detail see File S2). Figure 4 represents a novel gene found in *Y. pestis* Pestoides F: 13 peptides corresponding to the same translational frame were observed in a 427 nucleotide ORF between *YPDSF_3634* and *YPDSF_3635*. Using the high level of orthology between *Yersinia* strains, the potential sequence of this missed protein was predicted and maps with 100% identity to both *Y. pestis* CO92 and *Y. pseudotuberculosis* PB1/+. Peptide evidence represented 79% sequence coverage of the predicted protein, and correlated peptides were found in YPO and YPTS supporting the inclusion of this hypothetical protein in *Y. pestis* Pestoides F.

One major flaw of automated annotation is the under-prediction of small genes. This often occurs because large ORFs are conspicuous. Examination of protein lengths for these novel genes substantiates this issue. Protein length histograms (Figure S1) clearly show that the missed genes possess lengths that fall on the short end of the range of annotated *Yersinia* proteins. Given that short proteins yield fewer peptides, there is an increased likelihood that short proteins will be identified by single peptide hits. Thus the inclusion of confident singlet peptides is important, particularly for genome refinement studies.

Protein coding genes may also fail to be predicted due to the presence of an alternative translational start site. In the existing annotations, 82%, 88%, and 91% of predicted proteins for YPO, YPDSF, and YPTS, respectively, have an AUG initiation codon. Noticeably, YPDSF and YPTS have a higher percentage of purported AUG start codons relative to YPO and another enterobacteria, *E. coli*, which has been reported to use 83% AUG [36]. Based on comparison with orthologous sequences in other *Yersinia* strains, for YPO, YPDSF, and YPTS, respectively, 5, 6, and 2 of the novel genes found in this study are proposed to have a less common initiation codon: UUG/UUA or GUG rather than AUG. While no peptide evidence is present to confirm the predicted N-termini of these novel genes, it seems plausible that these genes were overlooked during annotation due to the presence of less common start codons.

Comparison of missed proteins from the targeted strain with orthologous sequences from the other two examined strains highlights a significant intention of this study: highly similar strains possess orthologous genes that can be used for cross-validation of proteomics data and for genome refinement. Of the 8 missed proteins in YPO, 6 and 4 orthologous proteins are not predicted in either YPDSF or YPTS, respectively. Similarly, of the 18 missed proteins in YPDSF, 5 and 2 orthologous proteins are presumed absent in YPO and YPTS, respectively. Two proteins were missed in YPTS; of these, 1 ortholog is not predicted in either YPO or YPDSF, and the remaining ortholog is not predicted in YPDSF. As YPO was the first *Yersinia* genome annotated, these results suggest transitive annotation omissions. During the genome annotation process, predicted protein coding genes are searched against protein databases. Homology findings are based on the quality of the software utilized and on the quality of the annotations that compile the protein databases [37]. The overlap of missing proteins across the three strains examined in this work suggests a propagation of errors for *Yersinia* strains. These overlaps may be a result of failed searches against previously annotated *Yersinia* genomes lacking predictions for the gene of interest.

### Identification of upstream start sites

**Figure 5:**
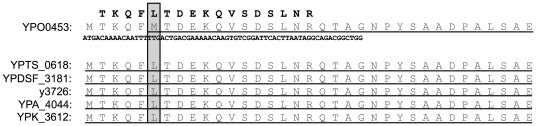
Representative identification of an incorrectly predicted translational start site. Protein sequence alignments are shown for the three strains examined (YPO, YPDSF, and YPTS) in addition to several other *Yersinia* species. Annotated protein sequences are underlined and experimental peptide and oligo evidence is shown in black text. For *YPO0453*, evidence flanks the predicted start site (shown by boxed region) and the observed peptide sequence reveals translation of a leucine instead of methionine confirming an N-terminal extension.

Another category of annotation error occurs when the translational start site of a gene is predicted incorrectly. This type of error has potentially deleterious effects for the structural genomics community which relies on accurate protein sequences for structure determinations and both function and localization predictions. The accuracy of start site prediction has been estimated to be as low as 80–90% [37]. Targeted proteogenomics strategies have successfully been used to characterize N-termini of *Deinococcus* proteins and findings revealed 73 incorrectly predicted start codon sites and the use of several non-canonical translational initiation codons [38]. Peptide evidence upstream of a predicted start site, but C-terminal to a stop codon, suggests that the start site was predicted to occur at the wrong codon. In YPO, YPDSF, and YPTS, there is evidence for the incorrect prediction of 3, 3, and 2 start sites, respectively (File S2). Comparative analysis of BLASTp results from sequences generated from a 5′ extension of each gene was used to assign new start sites. In all cases, orthologous sequences implied new start codons that were consistent with the observed experimental evidence. In 4 of the 8 start site anomalies, peptide evidence spanned the existing start codon substantiating extension to an upstream start site. Figure 5 shows an example of an incorrectly predicted start site in *Y. pestis* CO92 with peptide evidence spanning and oligo evidence 5′ of the existing TTG start codon of *YPO0453*. Comparison of orthologs suggests that translation should begin five residues upstream of the predicted start site. Notably, translation of TTG as a start codon would result in the generation of a methionine, however the unbiased search performed for this study identified this amino acid as a leucine supporting an incorrect start codon prediction.

### Identification of translated pseudogenes

Pseudogenes can be described as sequences of DNA that possess disruptions such as insertions, premature stop codons, or frameshifts that render them nonfunctional [39]. As such, these entries are typically excluded from protein databases because the DNA is not thought to be translated to protein. Pseudogenes are not uncommon in prokaryotes (although much less common than in eukaryotes which lack compact genomes), but *Y. pestis* is reported to have a disproportionately high number of predicted pseudogenes [20], [40]. Divergence from *Y. pseudotuberculosis* required a change in both the bacteria's phenotype and mode of transmission, and gene loss is presumed to play a major role in *Y. pestis* acquiring its niche as a virulent pathogen [20], [41], [42], [43]. It was originally reported that the genome for *Y. pestis* CO92 contained 149 pseudogenes [22]. Subsequently, comparative studies were performed and additional *Y. pestis* CO92 pseudogenes that were missed during the original annotation have been proposed [20], [40]. In this work, 40, 16, and 1 pseudogenes from YPO, YPDSF, and YPTS, respectively, exhibited experimental evidence of protein expression. The finding of pseudogene expression is not a novel proteogenomic observation. A number of authors present evidence challenging the belief that pseudogenes are translationally silent [13], [14], [17], [44], [45]. Since the function of expressed disrupted genes is ablated in most cases, the existence of inteins [44] and the active expression of interrupted genes *in vivo* [46] are very interesting. It seems valuable in high-throughput studies to include all coding sequences with the potential for expression in a protein database and allow biological validation studies to guide functional conclusions.

We identified the translation of genomic regions currently labeled as pseudogenes (File S2) belonging to two major categories. First, split genes containing insertion elements/transposons or other cargo, and second, altered genes containing indels or nonsense mutations. *The argD* locus represents an example of a split gene. In CO92, the DNA sequence homologous to *argD* is interrupted by the insertion of two IS21 genes (File S3). Notably, *argD* peptides and oligos were observed on both termini, flanking the insertion. The expression of the N-terminal portion of *argD* is presumably under the normal promotor structure. Inspection of the DNA region between the second IS21 element and the C-terminal portion of *argD* revealed that the promoter structure typically used to drive the IS element had likely been co-opted to drive expression of *argD* (Michael Chandler and Guy Duval, personal communication, and [47]).

The presence of the transposable element insertion was tested in a dozen Y. pestis strains of different biovars including eight biovar orientalis (same as CO92) strains from USA, Indonesia, South America, and Madagascar, and it was confirmed that the insertion occurs only in CO92 (data not shown). In *Yersinia*, as well as other species with high genomic fluidity [48], it is not unexpected to find ‘unique’ proteins lacking homology. Evolutionary relationships with transposons have been described [49], and speculatively, this process may provide one driver for reassembly of domains that could produce non-homologous proteins [50] for novel adaptive functions. The nature of the “pseudogenes” identified as present as translated proteins offers an enticing clue that the broad range of proteins that are found to be unique within many sequenced genomes may be driven by rapid evolution.

**Figure 6:**
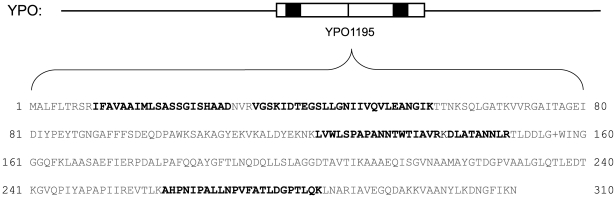
Representative identification of an expressed pseudogene. Pseudogenes are considered translationally silent and typically excluded from protein databases. *YPO1195* is categorized as a pseudogene due to the presence of a stop site mid sequence (shown by vertical black bar). Both oligo evidence (black boxes, upper frame) and peptide evidence (black text, lower frame) were observed on either side of the predicted stop codon, indicating expression of this feature.

Another example of pseudogene expression in *Y. pestis* CO92 is illustrated in Figure 6. *YPO1195* encodes a 310 aa protein, but a single point variation from A to T (confirmed by Sanger sequencing, File S4) renders a premature TAG stop codon in lieu of an AAG-encoded lysine rendering a truncated protein/predicted pseudogene with a 155 aa N-terminus and a 154 aa C-terminus. Interestingly, both peptide and oligo evidence span the regions on either side of the stop codon. Regardless of the means for expression, this example highlights a case where the *YPO1195* protein sequence should be included in the CO92 protein database.

### Identification of translational frameshifts

Determination of translational frameshifts is one of the more difficult tasks of genome annotation. This phenomenon, opposed to mutational or replication-based frameshifts, occurs when the ribosome either slips -1 base, stalls +1 base, or hops over a stretch of nucleotides during translation causing two translational reading frames to be expressed as a single protein [51]. Authentic frameshifting events can either be spontaneous or programmed, but what appear to be frameshifted proteins can also result from DNA sequencing errors. Annotation predictions can be guided by the referencing of known frameshifted proteins in similar species, but more often than not, there are few if any proteins to reference. Peptide chain release factor II (*prfB*) is one example of a +1 frameshift observed in several bacterial genomes. While *prfB* is annotated in some *Yersinia*, the frameshift is missing in many strains [17] revealing the challenge in annotating frameshifts even when reference frameshifts are known. As previously mentioned with regard to incorrectly predicted start sites, missed frameshifts result in truncated sequences that may impact structural biology studies. The current analysis identified three genes, all found in *Y. pestis* Pestoides F, that have evidence indicative of frameshifts (File S2). These genes currently have protein coding sequences predicted, but the predicted proteins appear to be truncated due to an apparent frameshift. Eight predicted pseudogenes in *Y. pestis* CO92 have experimental evidence in multiple reading frames suggestive of translational frameshifts which may explain a misclassification as a pseudogene.

**Figure 7:**
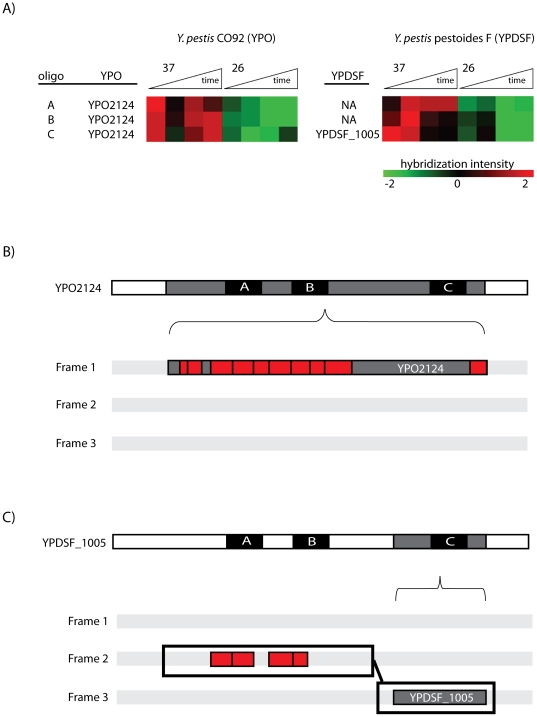
Representative identification of a putative translational frameshift. A) Hybridization evidence for oligos labeled A, B, and C is shown. Expression levels are shown normalized to each oligo's mean (via a Z-score calculation) across a time course/thermal switch (37°C/26°C) experiment for *Y. pestis* CO92 (YPO) and *Y. pestis* Pestoides F (YPDSF). Green indicates down-regulation relative to the mean, and red indicates up-regulation relative to the mean. Genome annotations are labeled for *YPDSF_1005* and *YPO2124* corresponding to annotated coding sequences. Although oligos A and B purportedly lack a corresponding transcription for YPDSF (NA = not applicable), evidence clearly shows hybridization consistent with oligo C. B) illustrates the 210 aa translation of *YPO2124* and C) illustrates the 63 aa translation of *YPDSF_1005*. Frame translations are shown below gene level detail with oligo evidence (black) overlaid on each gene and peptide evidence (red) overlaid on the appropriate reading frame. For *YPDSF_1005*, gene alignment with *YPO2124* reveals two oligos upstream of the coding region. Corroborating peptide evidence was also seen upstream but in a different reading frame than the existing annotation. This evidence supports the expression of *YPDSF_1005* as a frameshifted protein.

Figure 7 illustrates an apparent frameshift in *YPDSF_1005*, an ortholog of *YPO2124*. This gene encodes a hypothetical protein in both *Y. pestis* strains. *YPDSF_1005* encodes a 63 amino acid protein, and the expression of *YPO2124* produces a 210 amino acid product. Both genes have hybridization evidence showing similar thermal shift expression patterns for three oligos. All three oligos fall within the protein coding sequence boundaries for YPO, but two of the three oligos lie 5′ of the boundary for *YPDSF_1005*. Examination of YPDSF peptide data revealed four peptides upstream of the predicted start site, consistent with the oligo data. Importantly, Sanger sequencing (File S4) confirmed that these peptides all fell on a translational reading frame different from the predicted polypeptide. Extension of the N-terminus of *YPDSF_1005* on the alternate frame allowed the entire 210 amino acid product of *YPO2124* to be overlaid on the *YPDSF_1005* sequence indicating the presence of a frameshift.

### Utility of comparative data from orthologous strains

**Figure 8:**
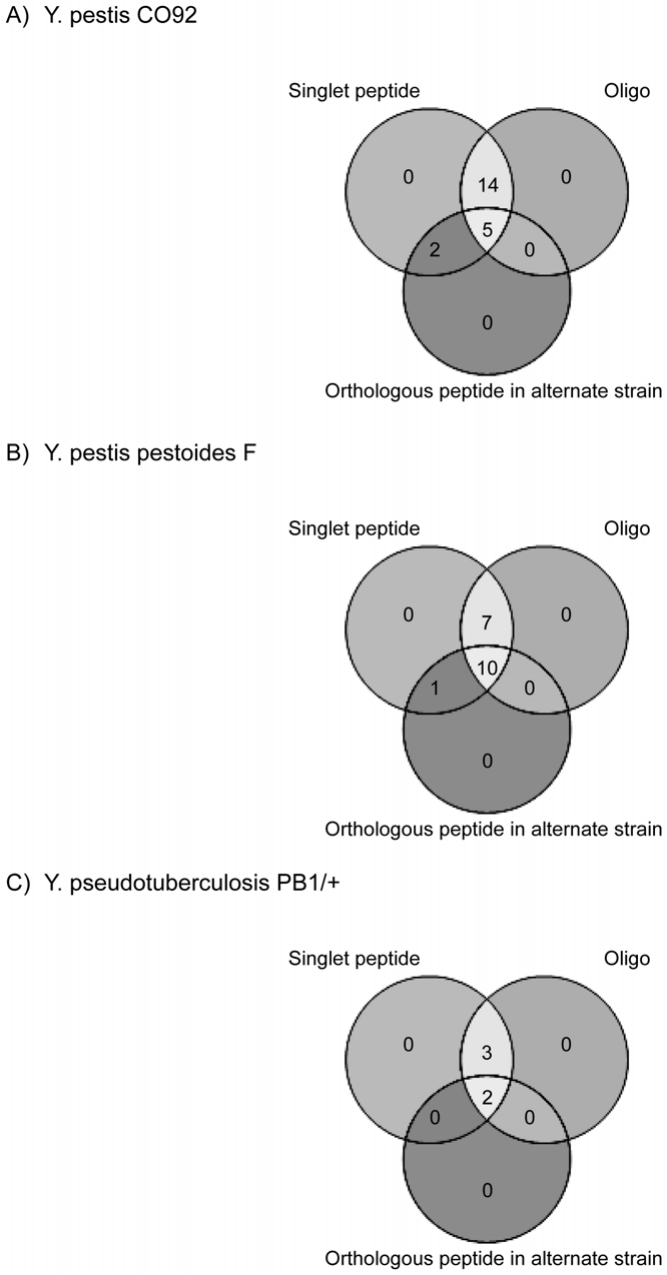
Venn diagram overlap of evidence for unannotated proteins identified by single peptide identifications. Open reading frames with evidence were initially filtered based on the presence of a non-redundant peptide. All singlet peptides were required to have corroborating oligo evidence or it was required that the singlet peptide mapped to an orthologous peptide in one of the alternate strains examined.

The genome annotation corrections presented here are in addition to the recent *Yersinia* annotation revisions by Payne et al. [17]. In that work, peptides were identified from *Y. pestis* KIM 6+ strain, and orthology clusters were used to extrapolate findings to the remaining 11 complete published *Yersinia* genomes (including the three examined in this work). Comparison of genome refinements made solely based on the orthology of experimental *Y. pestis* KIM 6+ results [17] with data gathered for this study revealed many annotation error overlaps thereby validating the prediction of proteins based on orthology. The comparative results from this omics-based study were confirmed by experimental design. Rather than extrapolating data to orthologous species, orthologous data was generated for multiple *Yersinia* strains in parallel. Unbiased oligo data is valuable evidence for these genome annotation refinements. The high level of overlap between peptide data and oligo data within coding sequences of predicted proteins (Figure 3) validates the use of oligo data as complementary support when the only evidence of protein expression is from singlet peptides. By allowing oligo evidence and orthologous peptide identifications to rescue singlets (Figure 8), 21, 18, and 5 annotation errors that otherwise would have been rejected by the two-peptide rule [52] were retained in YPO, YPDSF, and YPTS, respectively.

All errors described to this point were elucidated by the evidence thresholds described previously and are thus considered primary errors. Sequences of target strains possessing primary errors were aligned to the other strains examined in this study (File S2). Eight proteins had primary errors identified in multiple examined strains. Four of these errors indicate novel proteins in both strains, 2 errors suggest a novel protein in one strain and an upstream start site found in an alternate strain, 1 error shows an upstream start site in two strains, and 1 error shows a translated pseudogene in one strain and an upstream start site in the other. This level of overlap suggests a propagation of error during the predicted sequence searching portion of the annotation process. Primary errors found in 8 proteins from the target strain suggest 14 ‘secondary annotation errors’ in the orthologs. These secondary errors have experimental oligo evidence but lack peptide data, thus explaining their exclusion from the primary error list. Similar to ortho-proteogenomics studies, this information can be extrapolated to the respective genomes and these errors can be refined. It seems probable that as proteome coverage increases, expression of many of these proteins will be demonstrated with tangible evidence for each strain.

### Conclusions

Prokaryotic genome annotation is primarily performed *ab-initio*, but recent studies demonstrate that a combinatorial approach with both computational predictions and experimental evidence increases the accuracy of annotations [34], [35], [53]. The ease and utility of the proteogenomic approach is now well-documented and suggests that incorporation of experimental data for genome annotation refinement could become routine in the future. The finding of 96 previously undocumented errors in this study (Table 1) enforces the concept that genome annotation refinement is required for accurate studies by the scientific *Yersinia* community. Similar approaches could readily be applied to annotation refinement studies of other larger prokaryotic or eukaryotic [54] organisms.

The thresholds set for this comparative omics-based workflow were based on peptide evidence with oligo evidence included as optional secondary support, yet several hundred open reading frames across the genomes examined exhibited oligo hybridization without any confirmation that the mRNA was translated. This suggests that many additional annotation errors likely exist and implies that the refinement process is far from complete for these three genomes. Nearly 40% of each strain's predicted proteome was covered by peptide identifications in this work. Limitations of the technology contributed to incomplete coverage. Several bioanalytical factors inherent to bottom-up proteomics likely affected detection: post-translationally modified peptides and peptides from low abundance proteins or small proteins are difficult to observe. Itshould also be noted that the expression of many genes is tightly regulated and/or inducible only under specific conditions. For example, as much as a third of the *Salmonella* typhimurium proteome has been shown to be regulated at the translational level by the single virulence regulator Hfq [55], [56]. Hfq is only one mechanism of post-transcriptional regulation, but it does highlight the fact that regulation of translation and protein turn-over can lead to limited detection of highly regulated proteins. The conditions used here for *Yersinia* are intended to simulate important environmental transitions during pathogenesis so it is reasonable to assume that some RNA are transcribed but not translated into protein. A protein's observation is informative of a gene's presence, however, the absence of a protein when a transcript is present requires further analysis. Proteome depth will become greater and many of these oligo-supported protein predictions will be clarified as more experimental data is generated, growth conditions are tested, sample throughput is increased, and instrument sensitivity is improved.

The universal microarray used in these experiments proved fruitful for the discovery of annotation errors. High density tiling arrays and next generation sequencing technologies show even more promise for genome refinement studies. These newer technologies have the potential for increased sensitivity, specificity, and dynamic range relative to microarray profiling, and most importantly for annotation studies, the expression of open reading frames that have not been predicted to encode protein coding sequences can be measured [45], [57], [58].

The comparative omics-based approach employed in this study corroborates the utility of similar proteogenomic (simultaneously examining orthologous genome annotations) [16] and evidence-based (combining both transcriptional and peptide data) [18] work in different prokaryotic organisms. These results also help to define the transcription unit architecture [59], [60], a fundamental property that provides the basis for understanding key cellular processes such as metabolism and transcriptional regulation at the genome scale, of the *Yersinia* strains examined.

## Materials and Methods

### Cultivation of bacterial strains

*Y. pseudotuberculosis* PB1/+, *Y. pestis* Pestoides F, and a wild-type *Y. pestis* CO92 cured from pPCP1 plasmid were grown in a chemically defined medium BCS [61] in which neutral pH 7.2 was maintained by addition of 50 mM of morpholinopropanesulfonic acid (MOPS) as described previously [62]. Bacterial cultures were grown in Erlenmeyer flasks aerated at 200 rpm at 26°C. A starter culture was grown, diluted to optical density OD600 = 0.1 to begin overnight culture, and grown to an OD600 of ∼3.0. The overnight culture was back-diluted to OD600 = 0.1 and grown in two flasks at 26°C. When the cultures reached OD600 ∼0.5, one flask was moved to 37°C. Aliquots from both cultures were taken at 0, 1, 2, 4, and 8 hours, OD's measured, and samples prepared as described below for proteomics and transcriptomics.

### Reversed-phase nanocapillary LC-MS/MS analyses

Approximately 2×10^10^ bacteria were harvested from the culture at each time point, pelleted, and immediately frozen at −80°C. Thawed cell pellets were washed with 100 mM NH_4_HCO_3_ (pH 8), lysed via bead beating, and global protein digestions were performed as described previously [63]. Peptides were concentrated in a Speed-Vac (ThermoFisher, Savant) to ∼100 uL, a BCA protein assay performed to quantitate peptide concentration, and samples were either diluted for analysis or subjected to SCX fractionation. Aliquots (60 ug) from each time point were pooled together for each strain and subjected to offline LC fractionation by strong cation exchange (SCX) chromatography on a 200 mm×2.1 mm Polysulfoethyl A column (PolyLC, Columbia, MD) preceded by a 10 mm×2.1 mm guard column, using a flow rate of 0.2 mL/min. LC separations were performed using an Agilent 1100 series HPLC system (Agilent, Palo Alto, CA). Mobile phase solvents consisted of (A) 10 mM ammonium formate, 25% acetonitrile, pH 3.0 and (B) 500 mM ammonium formate, 25% acetonitrile, pH 6.8. Once loaded, isocratic conditions at 100% A were maintained for 10 min. Fraction collection began at 2.8 min. Peptides were separated using a gradient from 0 to 50% B over 40 min, followed by a gradient of 50 to 100% B over 10 min. The gradient was then held at 100% solvent B for 10 min. Following lyophilization, all 24 fractions for each of the two pools collected during this gradient were dissolved in 25 mM ammonium bicarbonate and stored at −80°C. Peptides (0.5 µg/µL) from global preparations (i.e., total unfractionated lysate) were run in triplicate on a linear ion trap (LTQ) Orbitrap Velos mass spectrometer (Thermo Scientific) (n = 30 LC-MS/MS runs per strain), and SCX fractionated samples were run on a LTQ mass spectrometer (Thermo Scientific) (n = 48 fractionated samples run per strain). Peptides were separated by a custom-built nanocapillary HPLC system as previously described [64], [65]. The eluate from the global preparations and fractionated samples was directly analyzed by electrospray ionization (ESI). The MS instruments were operated in data-dependent mode with *m/z* range of 400–2000, collision energy of 35 eV, and the 10 most intense peaks were selected for fragmentation. Raw data are available to the public at omics.pnl.gov and further information available at www.SysBEP.org.

### Six-frame peptide identification

MS/MS fragmentation spectra were searched against a six frame translation (minimum open reading frame length of 30 amino acids) of the *Y. pestis* CO92 genome and plasmids (NC_003143), *Y. pestis* Pestoides F genome and plasmids (NC_009381), and *Y. pseudotuberculosis* PB1/+ genome and plasmid (NC_010634) located at NCBI using SEQUEST [66] peptide identification software. The parent and fragment mass tolerances used for matching were set to ±3 and ±1 Da, respectively. The average peptide mass errors for the high resolution data were 0.79, 1.11, and 1.09 ppm for YPCO, YPPF, and YSTB, respectively. Peptide identifications were retained based upon the following criteria: 1) SEQUEST DelCn2 value ≥0.10; 2) SEQUEST correlation score (Xcorr) ≥1.9 for charge state 1+ for fully tryptic peptides and Xcorr ≥2.20 for 1+ for partially tryptic peptides; Xcorr ≥2.2 for charge state 2+ and fully tryptic peptides and Xcorr ≥3.3 for charge state 2+ and partially tryptic peptides; Xcorr ≥3.3 for charge state 3+ and fully tryptic peptides and Xcorr ≥4.0 for charge state 3+ and partially tryptic peptides. For each strain, the distribution of charges for detected peptides was as follows: YPCO (q1 = 3.8%, q2 = 55.5%, and q3 = 40.7%), YPPF (q1 = 3.8%, q2 = 56.7%, and q3 = 39.5%), and YSTB (q1 = 3.4%, q2 = 54.9%, and q3 = 41.7%). Redundant peptides (i.e., peptides that map to multiple proteins) were excluded from the analysis to minimize potential ambiguity. Using the reverse (decoy) database approach, the false discovery rate (FDR) of filter-passing spectra/proteins (minimum of two unique peptides per protein) was estimated to be 0.2%/0.1% for the Velos data, 0.8%/0.6% for the LTQ data, and 0.4%/0.8% for the combined data. Higher false-positive rates were associated with singlet peptide identifications, so annotations for these spectra are provided in the supporting data. As multiple pieces of evidence (peptide, oligo, and orthology) were used to identify annotation errors, we feel that the true FDR values are nominally lower than the peptide-based FDR values reported.

### Universal *Yersiniae* microarray design

A universal *Yersiniae* array was designed to contain probes targeting genes for seven different *Yersinia* genomes on a single microarray chip. In order to incorporate probes representative of both unique and homologous genes between the seven strains, gene FASTA files containing predicted ORF sequences for *Y. pestis* strains CO92, KIM, Pestoides F, Antiqua, Nepal516, and biovar Microtus str. 91001, and *Y. pseudotuberculosis* strain PB1/+ were collected as targets for oligonucleotide design. Homologous genes with high sequence similarity (i.e., >99% identity over the full length of the genes) were combined resulting in a single representative homolog for each homologous group. ArrayOligoSelector (http://arrayoligosel.sourceforge.net/) was used to design 70-mer oligonucleotides for each target with the goal of maximizing oligo to target gene binding energy (assessed as melting temperature, T_m_) while optimizing specificity by minimizing cross hybridization with non-target gene sequences. Following the first round of design, resultant oligos were assessed for the potential to cross hybridize with genes other than the intended target. Gene targets having ambiguous oligos mapping to other genes underwent a second round of design. The second round of design tiled the remaining gene sequences with matching oligos, followed by selection of the oligo for having the lowest number of unintended gene hits. Preference was made for designed oligos to target the 3′ end of each gene. Blastn (W = 15, bit score> = 280) was used to create a target map detailing each oligo's gene matches exhibiting a bit score > = 280 (90% identity over 70 bases). The final universal array included 7641 designed oligos which were printed in duplicate, in addition to 1000 control and 1958 empty probes. The array platform description and oligo list are available at NCBI Gene Expresssion Omnibus (GEO) under accession GPL9009.

### Microarray analysis of transcripts

At the appropriate times, 20–40 mL from each culture was removed and immediately mixed with an equal volume of cold RNAlater (Qiagen). Total RNA was isolated with a Qiagen Midi kit according to manufacturer's protocol. RNA was isolated from approximately 8×10^9^ bacteria per sample and concentrated with ethanol. RNA concentrations were determined by spectrophotometer (SmartSpecPlus, BioRad), quality was assessed by gel electrophoresis, and purified RNA was stored at −80°C until analysis. RNA samples were treated further to remove residual DNA contamination prior to labeling and microarray hybridizations with Ambion Turbo DNA-free DNAse. Microarray hybridizations with cDNA probes were accomplished on version 5QYP aminosilane-coated slides printed with a set of 18,240 elements; scanning, image analysis, and normalization were performed as outlined in PFGRC standard protocol (http://pfgrc.jcvi.org/index.php/microarray/protocols.html). Individual TIFF images from each channel were analyzed with JCVI Spotfinder software, and microarray data were normalized by LOWESS normalization using TM4 software MIDAS (both available at http://pfgrc.jcvi.org/index.php/bioinformatics.html). Oligos generating intensity signals ≥35,000 (≥3σ of control probes across all chips) were considered to have positive hybridization above background and therefore incorporated as experimental measurements. While not presented for all findings, in cases where multiple oligos map to a single open reading frame, expression patterns of annotated mRNA (as shown in Figure 7) can support the identification of anomalous hybridization signals across experimental samples. Transcriptomics data have been deposited in the GEO repository under series accession GSE30634.

### Data processing and genome refinement

Peptides that are correlated (i.e., differ at a single amino acid residue due to divergence/modification or otherwise identical peptides that differ in length) across strains were identified using PepAligner, an in-house program that compares two peptide files using Smith-Waterman alignment. The following criteria were established to objectively filter potential annotation errors based on experimental peptide and oligo evidence: 1) a non-redundant peptide and ≥1 additional non-redundant peptide or hybridized oligo or 2) a singlet peptide that has an orthologous peptide observed in ≥1 alternate strain. For the latter case, mass spectra were manually validated for confidence of protein expression (File S5). Distributions of evidence are provided in File S6. Experimental evidence was visualized using the Artemis genome browser [67] or Visual Exploration and Statistics for Proteomic Analyses (VESPA) ((https://www.biopilot.org/docs/Software/index.php). Using the established criteria, potential protein sequences from unannotated 5′ or intergenic regions were aligned to other *Yersinia* spp. and the non-redundant database using BLASTp [68].

## Supporting Information

File S1 **Lists of orthologous peptides.** All two-way comparisons from the three strains examined are provided. Singlet peptide observations from one strain are compared against all peptide observations from another strain. Detailed information including coverage and alignments is given.
(XLSX)

File S2 **Comparisons of errors across strains.** Both peptide and oligo evidence of errors is provided as visualized using the Artemis viewer. Errors were assigned arbitrary numerical values for organization only, and each proposed error (along with orthologous sequences from the other examined strains regardless of their error statuses) is represented on an individual page. .Forward and reverse DNA strands are labeled, along with each of the six translational reading frames. Vertical black bars represent stop codons, white regions represent DNA features, cyan regions represent protein coding sequences, yellow regions represent oligo evidence, and magenta regions represent peptide evidence.
(PDF)

File S3 **Peptide evidence related to the insertion-ablated pseudogene,** ***argD*** **.** The regions encompassing *argD* loci from Yersinia pestis strains CO92 and Pestoides F are shown. Annotated open reading frames are colored in yellow. Detected peptide evidence is mapped by blue arrows and sequences are provided.
(PDF)

File S4 **Sanger sequencing.** Methodology, primer sequences, primer maps, and sequence alignments are shown for YPO1195 and YPDSF_1005.
(PDF)

File S5 **Singlet peptide validations.** Three errors were supported by singlet identifications. Annotated spectra are provided for these peptides.
(PDF)

File S6 **Evidence summaries.** Evidence for all detected open reading frames is provided for each of the three strains.
(XLSX)

Figure S1 **Protein length histograms.** Bins are used to show the distribution of protein lengths for all protein coding genes (black) and novel annotation errors (grey).
(TIF)
