# Correction: Comparative Omics-Driven Genome Annotation Refinement: Application across Yersiniae

**DOI:** 10.1371/annotation/03110e8b-3e10-4334-9ff7-969c85ad25d8

**Published:** 2012-08-02

**Authors:** Alexandra C. Schrimpe-Rutledge, Marcus B. Jones, Sadhana Chauhan, Samuel O. Purvine, James A. Sanford, Matthew E. Monroe, Heather M. Brewer, Samuel H. Payne, Charles Ansong, Bryan C. Frank, Richard D. Smith, Scott N. Peterson, Vladimir L. Motin, Joshua N. Adkins

There were errors in the Funding section. The correct funding information is as follows: This research was supported by the National Institute of Allergy and Infectious Diseases NIH/DHHS through Interagency agreement Y1-AI-8401. Proteomics capabilities were developed under support from the U.S. Department of Energy (DOE) Office of Biological and Environmental Research and the NIH National Center for Research Resources (RR018522) and work was performed in the Environmental Molecular Sciences Laboratory, a DOE-BER national scientific user facility at Pacific Northwest National Laboratory (PNNL). PNNL is a multi-program national laboratory operated by Battelle Memorial Institute for the DOE under contract DE-AC05-76RLO 1830. The funders had no role in study design, data collection and analysis, decision to publish, or preparation of the manuscript. 

