# Supplementary material for: Comparative Omics-Driven Genome Annotation Refinement: Application across Yersiniae
Source: PLoS One. 2012 Mar 27;7(3):e33903. doi: 10.1371/journal.pone.0033903 (PMC3313959; doi:10.1371/journal.pone.0033903)
Supplement: File S2 — Comparisons of errors across strains. Both peptide and oligo evidence of errors is provided as visualized using the Artemis viewer. Errors were assigned arbitrary numerical values for organization only, and each proposed error (along with orthologous sequences from the other examined strains regardless of their error statuses) is represented on an individual page. .Forward and reverse DNA strands are labeled, along with each of the six translational reading frames. Vertical black bars represent stop codons, white regions represent DNA features, cyan regions represent protein coding sequences, yellow regions represent oligo evidence, and magenta regions represent peptide evidence. (PDF) [file pone.0033903.s002.pdf]

Peptide and oligo evidence for each of the three examined strains is provided as visualized using the Artemis viewer. Forward and reverse DNA strains are labeled along with each of the six translational reading frames. Vertical black bars represent stop codons, white regions represent DNA features, cyan regions represent protein coding regions, yellow regions represent oligo evidence, and magenta regions represent peptide evidence. Errors are assigned arbitrary numerical values for organization only, and each proposed protein error (along with orthologs from remaining examined strains) is represented on an individual page.

Error 1

Strain: Y. pestis CO92
Gene: YPO0089

Classification: expressed pseudogene
Boundaries of gene: complement (99525..100193)

Oligo evidence:

attccagacggtgacgttgctgcgtcaattttgacttgtatgccagaaagcgaagtggacgtgatgtact
gttatcttctctgccactggcattactaaaggtgacctgctagaaggtatctatcgtaaagggaatatgg

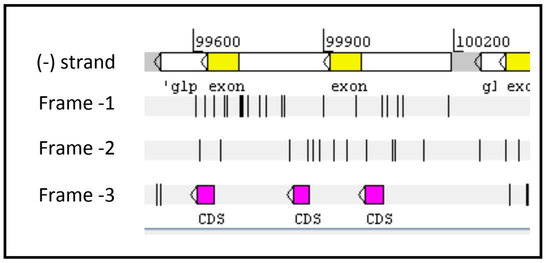

Peptide evidence:

HDGIIAAMQQLGVK
KGNMATTETLLIR
LDGDMQGRLLPR

Proposed sequence (peptide evidence in red): 222aa
APDMYMEKLVVGPAAKGAIDLNLPLEQNLCNIATALNKPLADLTVITLAKPRHDGIIAAMQQLGVKVFAIPDGDVAASILTCMPESEVDVMYCIGGAPEGVISAAVIRALDGDMQGRLLPRH
QVKGDSEDNRRIGEQELLRCKSMGIEAGNVLQLGDMARNDNVIFSATGITKGDLLEGIYRKGNMATTETLLIRGKSRTIRRIRSTHFLDRKDPALHEFLL

Strain: Y. pestis pestoides F
Gene: YPDSF_3814

Classification: annotated as glpX
Boundaries of gene: 4383581..4384591

Oligo evidence:

tagaaaggtgatagaattcttgcctatattcagtttttctattccgcaatcaacaggtctaaccatgaaa
atattcagtttttctattccgcaatcaacaggtctaaccatgaaacgtgaattagccatcgagttttccc
attccagacggtgacgttgctgcgtcaattttgacttgtatgccagaaagcgaagtggacgtgatgtact
gttatcttctctgccactggcattactaaaggtgacctgctagaaggtatctatcgtaaagggaatatgg

Peptide evidence:

GDKNAADGAAVQAMR
GNMATTETLLIR
HDGIIAAMQQLGVK
IMLNQVNIDGR
KGNMATTETLLIR
LDGDMQGRLLPR
NAADGAAVQAMR
NDNVIFSATGITK
SMGIEAGNVLQLGDMAR
VFAIPDGDVAASILTCMPESEVDVMYCIGGAPEGVISAAVIR
VTEAAALAGYK

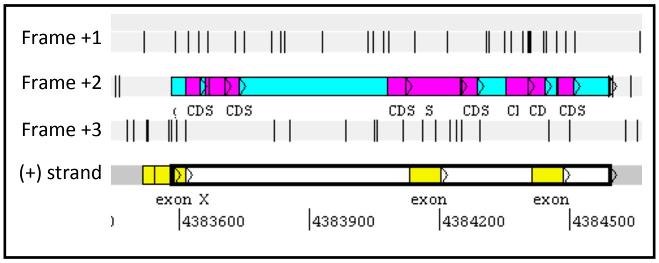

Current sequence (peptide evidence in red): 336aa
MKRELAIEFSRVTEAAALAGYKWLGRGDKNAADGAAVQAMRIMLNQVNIDGRIVIGEGEIDEAPMLFIGEHVGTGQGDAVDIAVDPIEGTRMTAMGQANALAVLAVGDQGTFLHAPDM
YMEKLVVGPAAKGAIDLNLPLEQNLCNIATALNKPLADLTVITLAKPRHDGIIAAMQQLGVKVFAIPDGDVAASILTCMPESEVDVMYCIGGAPEGVISAAVIRALDGDMQGRLLPRHQVKG
DSEDNRRIGEQELLRCKSMGIEAGNVLQLGDMARNDNVIFSATGITKGDLLEGIYRKGNMATTETLLIRGKSRTIRRIRSTHFLDRKDPALHEFLL

Strain: Y. pseudotuberculosis PB1/+
Gene: YPTS_0089

Classification: annotated as glpX
Boundaries of gene: complement (91704..92714)

Oligo evidence:

gttatcttctctgccactggcattactaaaggtgacctgctagaaggtatctatcgtaaagggaatatgg
attccagacggtgacgttgctgcgtcaattttgacttgtatgccagaaagcgaagtggacgtgatgtact
atattcagtttttctattccgcaatcaacaggtctaaccatgaaacgtgaattagccatcgagttttccc
atattcagtttttctattccgcaatcaacaggtctaaccatgaaacgtgaattagccatcgagttttccc

Peptide evidence:

ALDGDMQGR
GDKNAADGAAVQAMR
GDLLEGIYR
HDGIIAAMQQLGVK
IGEQELLR
IMLNQVNIDGR
KDPALHEFLL
KGNMATTETLLIR
NAADGAAVQAMR
NDNVIFSATGITK
STHFLDRKDPALHEFLL
VFAIPDGDVAASILTCMPESEVDVMYCIGGAPEGVISAAVIR
VTEAAALAGYK

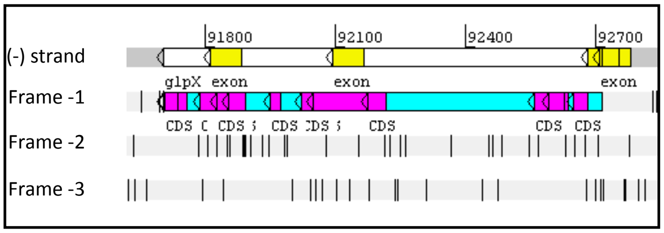

Current sequence (peptide evidence in red): 336aa
MKRELAIEFSRVTEAAALAGYKWLGRGDKNAADGAAVQAMRIMLNQVNIDGRIVIGEGEIDEAPMLFIGEHVGTGQGDAVDIAVDPIEGTRMTAMGQANALAVLAVGDQGTFLHAPDM
YMEKLVVGPAAKGAIDLNLPLEQNLCNIATALNKPLADLTVITLAKPRHDGIIAAMQQLGVKVFAIPDGDVAASILTCMPESEVDVMYCIGGAPEGVISAAVIRALDGDMQGRLLPRHQVKG
DSEDNRRIGEQELLRCKSMGIEAGNVLQLGDMARNDNVIFSATGITKGDLLEGIYRKGNMATTETLLIRGKSRTIRRIRSTHFLDRKDPALHEFLL

Error 2

Strain: Y. pestis CO92
Gene: YPO0090

Classification: expressed pseudogene (glpK)
Boundaries of gene: complement (100264..101355)

Oligo evidence:

tacgtgatgaattgaagttgataggcgatgccaccgattctgagtatttcgcgaccaaagtgaaaaacag

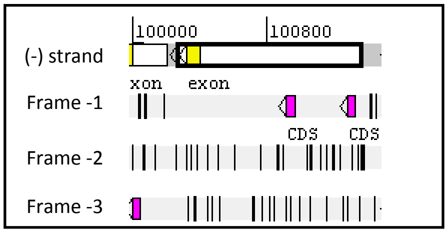

Peptide evidence:

HNTGLVVDPYFSGTK
YIVALDQGTTSSR

Proposed sequence (peptide evidence in red): 363aa
MTTENTTQKKYIVALDQGTTSSRAVVLDHNANIVSVSQREFTQIYPKAGWVEHDPMEIWATQSSTLIEVLAKAGINSDEIAGIGITNQRETTIVWDKVTGKPVYNAIVWQCRRTADICEKLKK
EGLEEYIRHNTGLVVDPYFSGTKVKWILDNVEGARERAERGELLFGTVDTWLVWNMTQGRVHVTDYTNASRTMMFNIRTKEWDDRMLKALNIPRAMLPEVRPSSEIYGKTNIGGKGGTRI
PIAGIAGDQQAALFGQLCVQPGMAKNTYGTGCFLLMNTGEEAVQSTHGLLTTIACGPRGEVNYALEGAVFIGGASIQWLRDELKLIGDATDSEYFATKVKNSNGVYVVPAFTGLGAPYWDP

Strain: Y. pestis pestoides F
Gene: YPDSF_3813

Classification: annotated as hypothetical protein
Boundaries of gene: 4381821..4383344

Oligo evidence:

tacgtgatgaattgaagttgataggcgatgccaccgattctgagtatttcgcgaccaaagtgaaaaacag
ttctgggatgacctcgatgaggtgaagagtaaagcaagcattgaacgcgaattccgtccagggattgaaa

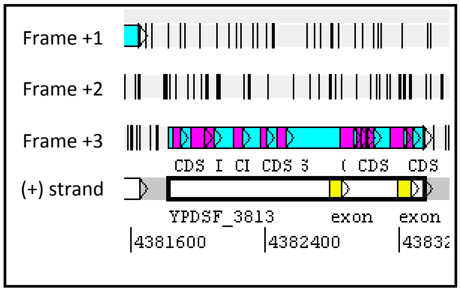

Peptide evidence:

AGINSDEIAGIGITNQR
AGWVEHDPMEIWATQSSTLIEVLAK
AMLPEVRPSSEIYGK
ATLESIAYQTR
DVLDAMQADSGAR
EFRPGIETTER
ESTALGAAFLAGLATGFWDDLDEVK
GVNSNHIIR
HNTGLVVDPYFSGTK
NSNGVYVVPAFTGLGAPYWDPYAR
VHVTDYTNASR
YIVALDQGTTSSR

Current sequence (peptide evidence in red): 507aa
MTTENTTQKKYIVALDQGTTSSRAVVLDHNANIVSVSQREFTQIYPKAGWVEHDPMEIWATQSSTLIEVLAKAGINSDEIAGIGITNQRETTIVWDKVTGKPVYNAIVWQCRRTADICEKLKK
EGLEEYIRHNTGLVVDPYFSGTKVKWILDNVEGARERAERGELLFGTVDTWLVWNMTQGRVHVTDYTNASRTMMFNIRTKEWDDRMLKALNIPRAMLPEVRPSSEIYGKTNIGGKGGTRI
PIAGIAGDQQAALFGQLCVQPGMAKNTYGTGCFLLMNTGEEAVQSTHGLLTTIACGPRGEVNYALEGAVFIGGASIQWLRDELKLIGDATDSEYFATKVKNSNGVYVVPAFTGLGAPYWDP
YARGAIFGLTRGVNSNHIIRATLESIAYQTRDVLDAMQADSGARLKSLRVDGGAVANNFLMQFQADILGTRVERPAIRESTALGAAFLAGLATGFWDDLDEVKSKASIEREFRPGIETTERDIR
YKGWKKAVARARDWEEHDE

Strain: Y. pseudotuberculosis PB1/+
Gene: YPTS_0090

Classification: annotated as glycerol kinase
Boundaries of gene: complement (92967..94490)

Oligo evidence:

ttctgggatgacctcgatgaggtgaagagtaaagcaagcattgaacgcgaattccgtccagggattgaaa
tacgtgatgaattgaagttgataggcgatgccaccgattctgagtatttcgcgaccaaagtgaaaaacag

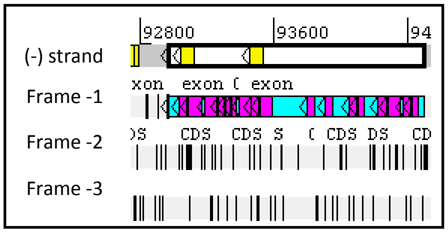

Peptide evidence:

AGINSDEIAGIGITNQR
AGWVEHDPMEIWATQSSTLIEVLAK
AMLPEVRPSSEIYGK
ATLESIAYQTR
AVVLDHNANIVSVSQR
DVLDAMQADSGAR
EFRPGIETTER
ESTALGAAFLAGLATGFWDDLDEVK
GEVNYALEGAVFIGGASIQWLRDELK
GVNSNHIIR
HNTGLVVDPYFSGTK
LIGDATDSEYFATK
LKKEGLEEYIR
NSNGVYVVPAFTGLGAPYWDPYAR
VDGGAVANNFLMQFQADILGTR
VHVTDYTNASR
YIVALDQGTTSSR

Current sequence (peptide evidence in red): 507aa
MTTENTTQKKYIVALDQGTTSSRAVVLDHNANIVSVSQREFTQIYPKAGWVEHDPMEIWATQSSTLIEVLAKAGINSDEIAGIGITNQRETTIVWDKVTGKPVYNAIVWQCRRTADICEKLKK
EGLEEYIRHNTGLVVDPYFSGTKVKWILDNVEGARERAERGELLFGTVDTWLVWNMTQGRVHVTDYTNASRTMMFNIRTKEWDDRMLKALNIPRAMLPEVRPSSEIYGKTNIGGKGGTRI
PIAGIAGDQQAALFGQLCVQPGMAKNTYGTGCFLLMNTGEEAVQSTHGLLTTIACGPRGEVNYALEGAVFIGGASIQWLRDELKLIGDATDSEYFATKVKNSNGVYVVPAFTGLGAPYWDP
YARGAIFGLTRGVNSNHIIRATLESIAYQTRDVLDAMQADSGARLKSLRVDGGAVANNFLMQFQADILGTRVERPAIRESTALGAAFLAGLATGFWDDLDEVKSKASIEREFRPGIETTERDIR
YKGWKKAVARARDWEEHDE

Error 3

Strain: Y. pestis CO92
Gene: YPO0115

Classification: expressed pseudogene (metB)
Boundaries of gene: Frame+3:122247..123235
Frame+2:123233..123406

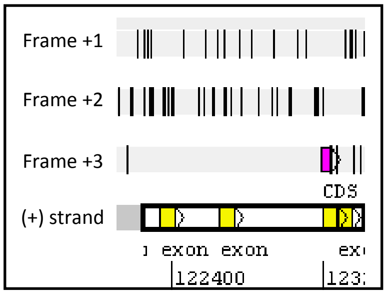

Oligo evidence:

tttccagtacctacaattttattgattttaatcagccgcgcacgcatgactattcacgtcgtggtaatcc
ggcggagaaaccgaagttggtcttgattgaaacaccgagtaatccattgctacgggttgttgatattgcc
ttgagctatttaccttggcagagtctttggggggtagaaagcctgatctcccatgcagcgaccatgacc
gcgggtatggcggcagaggcgcgtattgccgcaggcattactgatagtttgttgcgtatttccgtgggta

Peptide evidence:

FLSALELFTLAESLGG

Proposed sequence (peptide evidence in red):
Frame+3: 330aa
MTRKQATIAVRSGLNDDEQYGCVVPPIHLSSTYNFIDFNQPRTHDYSRRGNPTRDVVQRALAELEGGAGAVMTSSGMSALHLVCTTFLQPGDLLVAPHDCYGGSYRLFDSLSKRGAYRVLFV
DQGDEAALNCALAEKPKLVLIETPSNPLLRVVDIAAICQAARAAGALTVCDNTFLSPALQQPLSLGADLVVHSCTKYLNGHSDVVAGAVIAKDPELVVELAWWANNIGVTGAAFDSYLLLRGL
RTLSPRMAQQQRNADDIVRYLQQQPLVKKLYHPSLPQHPGHEIACRQQSGFGAMLSFELDGDEQVMRRFLSALELFTLAESLGG

Frame+2: 58aa
VESLISHAATMTHAGMAAEARIAAGITDSLLRISVGIEDSEDLIADLDHAFQLAVTR

Strain: Y. pestis pestoides F
Gene: YPDSF_3792

Classification: annotated as cystathionine gamma-synthase
Boundaries of gene: complement (4361729..4362889)

Oligo evidence:

gcgggtatggcggcagaggcgcgtattgccgcaggcattactgatagtttgttgcgtatttccgtgggta
ggcggagaaaccgaagttggtcttgattgaaacaccgagtaatccattgctacgggttgttgatattgcc
tttccagtacctacaattttattgattttaatcagccgcgcacgcatgactattcacgtcgtggtaatcc

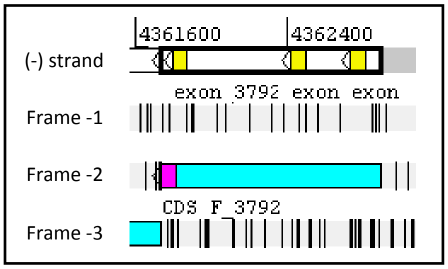

Peptide evidence:

ISVGIEDSEDLIADLDHAFQLAVTR

Current sequence (peptide evidence in red): 386aa
MTRKQATIAVRSGLNDDEQYGCVVPPIHLSSTYNFIDFNQPRTHDYSRRGNPTRDVVQRALAELEGGAGAVMTSSGMSALHLVCTTFLQPGDLLVAPHDCYGGSYRLFDSLSKRGAYRVLFV
DQGDEAALNCALAEKPKLVLIETPSNPLLRVVDIAAICQAARAAGALTVCDNTFLSPALQQPLSLGADLVVHSCTKYLNGHSDVVAGAVIAKDPELVVELAWWANNIGVTGAAFDSYLLLRGL
RTLSPRMAQQQRNADDIVRYLQQQPLVKKLYHPSLPQHPGHEIACRQQSGFGAMLSFELDGDEQVMRRFLSALELFTLAESLGGVESLISHAATMTHAGMAAEARIAAGITDSLLRISVGIE
DSEDLIADLDHAFQLAVTR

Strain: Y. pseudotuberculosis PB1/+
Gene: YPTS_0108

Classification: annotated as cystathionine gamma-synthase
Boundaries of gene: 112651..113892

Oligo evidence:

gttgaatgatgacgagcaatacggctgcgttgtccccccgattcacctttccagtacttacaattttatt
ggcggagaaaccgaagttggtcttgattgaaacaccgagtaatccattgctacgggttgttgatattgcc
gcgggtatggcggcagaggcgcgtattgccgcaggcattactgatagtttgttgcgtatttccgtgggta

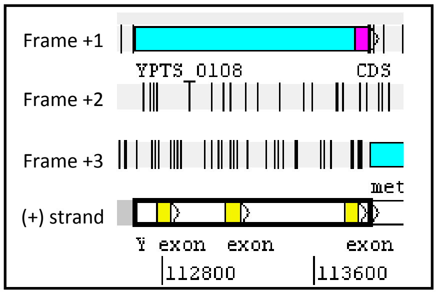

Peptide evidence:

ISVGIEDSEDLIADLDHAFQLAVTR

Current sequence (peptide evidence in red): 413aa
MSRCIDVRILCVYSGVTFSRSRLVEINMTRKQATIAVRSGLNDDEQYGCVVPPIHLSSTYNFIDFNQPRTHDYSRRGNPTRDVVQRALAELEGGAGAVMTSSGMSAIHLVCTTFLQPGDLLVA
PHDCYGGSYRLFDSLSKRGAYRVLFVDQGDEAALNRALAEKPKLVLIETPSNPLLRVVDIAAICQAARAAGALTVCDNTFLSPALQQPLSLGADLVVHSCTKYLNGHSDVVAGAVIAKDPELVV
ELAWWANNIGVTGAAFDSYLLLRGLRTLSPRMAQQQRNADDIVRYLQQQPLVKKLYHPSLPQHPGHEIACRQQSGFGAMLSFELDGDEQVMRRFLSALELFTLAESLGGVESLISHAATMT
HAGMAAEARIAAGITDSLLRISVGIEDSEDLIADLDHAFQLAVTR

Error 4

Strain: Y. pestis CO92
Gene: YPO0124

Classification: expressed pseudogene (malP)
Boundaries of gene: Frame+2: 134066..134389
Frame+1: 134389..136467

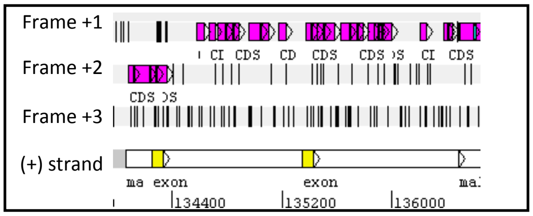

Oligo evidence:

tacatttcgatggagtttttaattggtcgtttgacagctaataacctaattaatctgggttggtatgaca
tgcattcggatctcattatcaaagatctgttccctgagtattaccaattgtggccaaataaattccacaa

Peptide evidence:

DDFLAALTR
FGLTSAQQMTPYQWWEAVSAALAEQLSAQPAPSKPK
HVNYISMEFLIGR
KDDFLAALTR
LTANNLINLGWYDTVDALLAEQQVK
DELASGAFSQGDK
DLFPEYYQLWPNK
DNPALDIAPR
FHNVTNGITPR
FLLAEQNGVEAEKLTK
GEAWDLPVLGFR
HFVIIKQINAQFK
HNAALAVDVGFGGNLVK
KLAELPDYEVIQLNDTHPTIAIPEMLR
LAELPDYEVIQLNDTHPTIAIPEMLR
LKVVFIPDYR
LWQATHQHPFDLTLFNDGK
NIIYAINQVADK
NIIYAINQVADKINNDPIVKDR
PNDNHLAGKR
QCNPALSGLIDDTLK
QHLNLLHILSLYR
QIRDNPALDIAPR
RAILNTAR
RVMGLVINPDAIFDVQIKR
VEWANDLDVLQDLEPYAEDPAFR
VGMFSSDR
VLLDEHQLSWDAAWAITSK
VLYPNDNHLAGK
VLYPNDNHLAGKR
VMGLVINPDAIFDVQIK
VSVAELMIPAADVSEQISTAGK

Proposed sequence (peptide evidence in red):
Frame+2: 109aa
MSQPMLKKDDFLAALTRQWQRFGLTSAQQMTPYQWWEAVSAALAEQLSAQPAPSKPKNVQRHVNYISMEFLIGRLTANNLINLGWYDTVDALLAEQQVKLSDLLEQET

Frame+1: 693aa
DPALGNGGLGRLAACFLDSMATVEQPATGYGLNYQYGLFRQSFRECKQQEAPDNWQRESYPWFRHNAALAVDVGFGGNLVKQADGRQLWRPAFTLRGEAWDLPVLGFRNGVTQPLRLWQATHQHPFDLTLFNDGKFLLAEQNGVEAEKLTKVLYPND
NHLAGKRLRLMQQYFQCACSVADILRKHHLAGRKLAELPDYEVIQLNDTHPTIAIPEMLRVLLDEHQLSWDAAWAITSKTFAYTNHTLMPEALECWDEKLVRSLLPRHFVIIKQINAQFKKLVNKQWPGNDEVWAKLAVHHNKQVRMANLCVVSGFAVNG
VAQLHSDLIIKDLFPEYYQLWPNKFHNVTNGITPRRWLKQCNPALSGLIDDTLKVEWANDLDVLQDLEPYAEDPAFRQRYQQIKYDNKVKLAHYVKRVMGLVINPDAIFDVQIKRLHEYKRQHLNLLHILSLYRQIRDNPALDIAPRVFLFGAKAAPGYYLAKNII
YAINQVADKINNDPIVKDRLKVVFIPDYRVSVAELMIPAADVSEQISTAGKEASGTGNMKMALNGALTVGTLDGANVEIAEQVGDENIFIFGHTVDQVKAILAKGYQPKKYVKADPHLKSILDELASGAFSQGDKQAFDMMLHSLLEGGDPYLVLADFASYCQ
AQKQIDALYRDKDEWTRRAILNTARVGMFSSDRSIRDYQQRIWQAKR

Strain: Y. pestis pestoides F
Gene: YPDSF_0052

Classification: glycogen/starch
/alpha-glucan phosphorylase
Boundaries of gene: 62556..64961

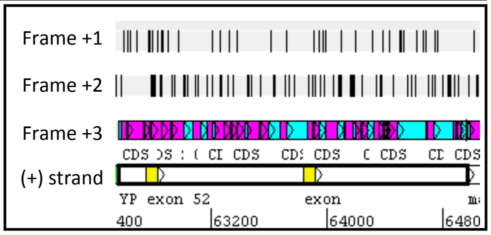

Oligo evidence:

tacatttcgatggagtttttaattggtcgtttgacagctaataacctaattaatctgggttggtatgaca
tgcattcggatctcattatcaaagatctgttccctgagtattaccaattgtggccaaataaattccacaa

Peptide evidence:

DDFLAALTR
DELASGAFSQGDK
DLFPEYYQLWPNK
DLFPEYYQLWPNKFHNVTNGITPR
DNPALDIAPR
DSMATVEQPATGYGLNYQYGLFR
FGLTSAQQMTPYQWWEAVSAALAEQLSAQPAPSKPK
FHNVTNGITPR
FLLAEQNGVEAEK
GEAWDLPVLGFR
HFVIIKQINAQFK
HNAALAVDVGFGGNLVK
HVNYISMEFLIGR
INNDPIVKDR
KDDFLAALTR
KLAELPDYEVIQLNDTHPTIAIPEMLR
LAELPDYEVIQLNDTHPTIAIPEMLR
LKVVFIPDYR
LMQQYFQCACSVADILR
LSDLLEQETDPALGNGGLGR
LTANNLINLGWYDTVDALLAEQQVK
LWQATHQHPFDLTLFNDGK
NIIYAINQVADK
NIIYAINQVADKINNDPIVK
NIIYAINQVADKINNDPIVKDR
PNDNHLAGKR
QHLNLLHILSLYR
QIRDNPALDIAPR
RAILNTAR
RVMGLVINPDAIFDVQIK
RVMGLVINPDAIFDVQIKR
SILDELASGAFSQGDK
VEWANDLDVLQDLEPYAEDPAFR
VGMFSSDR
VLLDEHQLSWDAAWAITSK
VLYPNDNHLAGK
VLYPNDNHLAGKR
VMGLVINPDAIFDVQIK
VMGLVINPDAIFDVQIKR
VSVAELMIPAADVSEQISTAGK

Current sequence (peptide evidence in red): 801aa
MSQPMLKKDDFLAALTRQWQRFGLTSAQQMTPYQWWEAVSAALAEQLSAQPAPSKPKNVQRHVNYISMEFLIGRLTANNLINLGWYDTVDALLAEQQVKLSDLLEQETDPALGNGGLGRLAACFLDSMATVEQPATGYGLNYQYGLFRQSFRECKQQE
APDNWQRESYPWFRHNAALAVDVGFGGNLVKQADGRQLWRPAFTLRGEAWDLPVLGFRNGVTQPLRLWQATHQHPFDLTLFNDGKFLLAEQNGVEAEKLTKVLYPNDNHLAGKRLRLMQQYFQCACSVADILRKHHLAGRKLAELPDYEVIQLNDTHP
TIAIPEMLRVLLDEHQLSWDAAWAITSKTFAYTNHTLMPEALECWDEKLVRSLLPRHFVIIKQINAQFKKLVNKQWPGNDEVWAKLAVHHNKQVRMANLCVVSGFAVNGVAQLHSDLIIKDLFPEYYQLWPNKFHNVTNGITPRRWLKQCNPALSGLIDDT
LKVEWANDLDVLQDLEPYAEDPAFRQRYQQIKYDNKVKLAHYVKRVMGLVINPDAIFDVQIKRLHEYKRQHLNLLHILSLYRQIRDNPALDIAPRVFLFGAKAAPGYYLAKNIIYAINQVADKINNDPIVKDRLKVVFIPDYRVSVAELMIPAADVSEQISTAGKEA
SGTGNMKMALNGALTVGTLDGANVEIAEQVGDENIFIFGHTVDQVKAILAKGYQPKKYVKADPHLKSILDELASGAFSQGDKQAFDMMLHSLLEGGDPYLVLADFASYCQAQKQIDALYRDKDEWTRRAILNTARVGMFSSDRSIRDYQQRIWQAKR

Strain: Y. pseudotuberculosis PB1/+
Gene: YPTS_3971

Classification: glycogen/starch/alpha-glucan phosphorylase
Boundaries of gene: complement (4411252..4413657)

Oligo evidence:

tgcattcggatctcattatcaaagatctgttccctgagtattaccaattgtggccaaataaattccacaa
tacatttcgatggagtttttaattggtcgtttgacagctaataacctaattaatctgggttggtatgaca

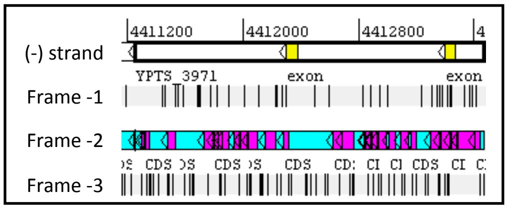

Peptide evidence:

DDFLAALTR
DNPALDIAPR
FGLTSAQQMTPYQWWEAVSAALAEQLSAQPAPSKPK
FHNVTNGITPR
FLLAEQNGVEAEK
FLLAEQNGVEAEKLTK
GEAWDLPVLGFR
HNAALAVDVGFGGNLVK
HVNYISMEFLIGR
KDDFLAALTR
KLAELPDYEVIQLNDTHPTIAIPEMLR
LAELPDYEVIQLNDTHPTIAIPEMLR
LKVVFIPDYR
LSDLLEQETDPALGNGGLGR
LTANNLINLGWYDTVDALLAEQQVK
LWQATHQHPFDLTLFNDGK
NIIYAINQVADK
NIIYAINQVADKINNDPIVK
NIIYAINQVADKINNDPIVKDR
QHLNLLHILSLYR
QIRDNPALDIAPR
QQEAPDNWQR
RAILNTAR
RVMGLVINPDAIFDVQIKR
SILDELASGAFSQGDK
VEWANDLDVLQDLEPYAEDPAFR
VGMFSSDR
VLLDEHQLSWDAAWAITSK
VLYPNDNHLAGK
VLYPNDNHLAGKR
VMGLVINPDAIFDVQIK
VSVAELMIPAADVSEQISTAGK

Current sequence (peptide evidence in red): 801aa
MSQPMLKKDDFLAALTRQWQRFGLTSAQQMTPYQWWEAVSAALAEQLSAQPAPSKPKNVQRHVNYISMEFLIGRLTANNLINLGWYDTVDALLAEQQVKLSDLLEQETDPALGNGGLGRLAACFLDSMATVEQPATGYGLNYQYGLFRQSFRECKQQE
APDNWQRESYPWFRHNAALAVDVGFGGNLVKQADGRQLWRPAFTLRGEAWDLPVLGFRNGVTQPLRLWQATHQHPFDLTLFNDGKFLLAEQNGVEAEKLTKVLYPNDNHLAGKRLRLMQQYFQCACSVADILRKHHLAGRKLAELPDYEVIQLNDTHP
TIAIPEMLRVLLDEHQLSWDAAWAITSKTFAYTNHTLMPEALECWDEKLVRSLLPRHFVIIKQINAQFKKLVNKQWPGNDEVWAKLAVHHNKQVRMANLCVVSGFAVNGVAQLHSDLIIKDLFPEYYQLWPNKFHNVTNGITPRRWLKQCNPALSGLIDDT
LKVEWANDLDVLQDLEPYAEDPAFRQRYQQIKYDNKVKLAHYVKRVMGLVINPDAIFDVQIKRLHEYKRQHLNLLHILSLYRQIRDNPALDIAPRVFLFGAKAAPGYYLAKNIIYAINQVADKINNDPIVKDRLKVVFIPDYRVSVAELMIPAADVSEQISTAGKEA
SGTGNMKMALNGALTVGTLDGANVEIAEQVGDENIFIFGHTVDQVKAILAKGYQPKKYVKADPHLKSILDELASGAFSQGDKQAFDMMLHSLLEGGDPYLVLADFASYCQAQKQIDALYRDKDEWTRRAILNTARVGMFSSDRSIRDYQQRIWQAKR

Error 5

Strain: Y. pestis CO92
Gene: YPO0170

Classification: expressed pseudogene
Boundaries of gene: Frame+2, Region 1: 189506..190051
Frame+2, Region 2: 192011-192694

Oligo evidence:
NA

Peptide evidence:

IIAFHNAFHGR

NTFDQVILPVYAPAQFIPVK

HGLFVQALQSINSK

LAPSLVIELEDIQQGMAR

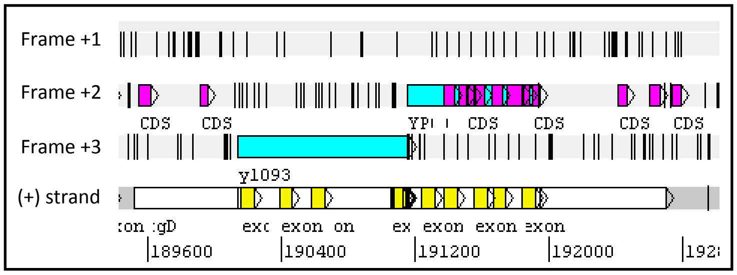

Proposed sequence (peptide evidence in red):
Region 1: 182aa
VGSVMTDKLAVNRNTFDQVILPVYAPAQFIPVKGKGSRVWDQQGTEYIDFAGGIAVTALGHCHPALVSALHQQGETLWHTSNVFTNEPALRLAQKLIAATFADRVFFANSGAEANEAAFKL
ARHYAIERHSPYKTKIIAFHNAFHGRTLFTVSVGGQPKYSDGFGPKPADIIHVPFNDLAAV

Region 2: 227aa
KAVMDDHTCAVVLEPIQGEGGITSATPEFLQGVRALCDQHNALLVFDEVQSGMGRSGKLFSYMHYGVTPDILTTAKALGGGFPISAMLTTEEIASVMTVGTHGTTYGGNPLACAVAEAALD
VINTPEVLNGIEQRHGLFVQALQSINSKYDVFSDIRGMGLLIGAELTAKYRGQAREFLAAAAANGLMILNAGPDVLRLAPSLVIELEDIQQGMARLEKAMASVIKG

Strain: Y. pestis pestoides F
Gene: YPDSF_0098

Classification: annotated as argD
Boundaries of gene: 117983..119200

Oligo evidence:
atatcatccatgtcccgtttaatgatctggcggcagtgaaagcggtgatggatgatcacacctgtgccgt

Peptide evidence:
IIAFHNAFHGR

LFSYMHYGVTPDILTTAK

HGLFVQALQSINSK

LAPSLVIELEDIQQGMAR

NTFDQVILPVYAPAQFIPVK

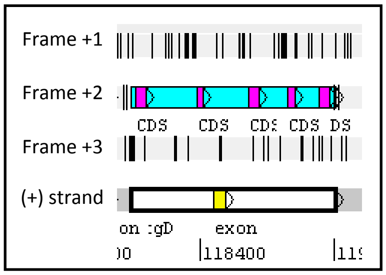

Current sequence (peptide evidence in red): 405aa
MTDKLAVNRNTFDQVILPVYAPAQFIPVKGKGSRVWDQQGTEYIDFAGGIAVTALGHCHPALVSALHQQGETLWHTSNVFTNEPALRLAQKLIAATFADRVFFANSGAEANEAAFKLARHY
AIERHSPYKTKIIAFHNAFHGRTLFTVSVGGQPKYSDGFGPKPADIIHVPFNDLAAVKAVMDDHTCAVVLEPIQGEGGITSATPEFLQGVRALCDQHNALLVFDEVQSGMGRSGKLFSYMHY
GVTPDILTTAKALGGGFPISAMLTTEEIASVMTVGTHGTTYGGNPLACAVAEAALDVINTPEVLNGIEQRHGLFVQALQSINSKYDVFSDIRGMGLLIGAELTAKYRGQAREFLAAAAANGLMI
LNAGPDVLRLAPSLVIELEDIQQGMARLEKAMASVIKG

Strain: Y. pseudotuberculosis PB1/+
Gene: YPTS_3924

Classification: annotated as argD
Boundaries of gene: complement (4356817..4358034)

Oligo evidence:
atatcatccatgtcccgtttaatgatctggcggcagtgaaagcggtgatggatgatcacacctgtgccgt

Peptide evidence:

HGLFVQALQSINSK
IIAFHNAFHGR
LAPSLVIELEDIQQGMAR
LFSYMHHGVTPDILTTAK
NTFDQVILPVYAPAQFIPVK
TLFTVSVGGQPK
VFFANSGAEANEAAFK
YDVFSDIR

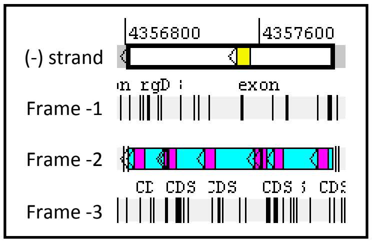

Current sequence (peptide evidence in red): 405aa
MTDKLAVNRNTFDQVILPVYAPAQFIPVKGKGSRVWDQQGTEYIDFAGGIAVTALGHCHPALVSALHQQGETLWHTSNVFTNEPALRLAQKLIAATFADRVFFANSGAEANEAAFKLARHY
AIERHSPYKTKIIAFHNAFHGRTLFTVSVGGQPKYSDGFGPKPADIIHVPFNDLAAVKAVMDDHTCAVVLEPIQGEGGITSATPEFLQGVRALCDQHNALLVFDEVQSGMGRSGKLFSYMHH
GVTPDILTTAKALGGGFPISAMLTTEEIASVMTVGTHGTTYGGNPLACAVAEAALDVINTPEVLNGIEQRHGLFVQALQSINSKYDVFSDIRGMGLLIGAELTAKYRGQAREFLAAAAANGLM
LNAGPDVLRLAPSLVIELEDIQQGMARLEKAMASVIKG

Error 6

Strain: Y. pestis CO92
Gene: YPO0188

Classification: expressed pseudogene
Boundaries of gene: Frame -1: complement (207454..207981)
Frame -2: complement (206063..207457)

Oligo evidence:

tgttatcgacggtgaccgcgaatatcgccagttggaggccgaacttcaggctgccaatgaaaaaaatgat
taccttggtattaatttcccatgaccgcgatttccttgatcctattatcgataagattttgcatattgaa

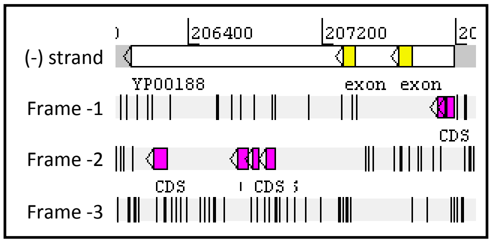

Peptide evidence:

MIVFSSLQIRRGTRVLLDNATATINPGQK
ADESPLQHMSR
DYLGGYGFQGDQVTDPTAR
LLAGTLEPQSGEIALSK
LSTELAAIEAQLADSALYDIAR

Proposed sequence (peptide evidence in red):
Frame-1: 176aa
MIVFSSLQIRRGTRVLLDNATATINPGQKVGLVGKNGCGKSTLLALLKGELSADGGNVTFPNNWALAWVNQETPALDIPAIEYVIDGDREYRQLEAELQAANEKNDGHAIATVHGKLDAIHA
WTIQSRAASLLHGLGFSQEKLQQPVRSFSGGWRMRLNLAQALVCRSDLLLLDEP

Frame-2: 464aa
TNHLDLDAVIWLEKWLKSYTGTLVLISHDRDFLDPIIDKILHIEQQTLNEYTGNYSSFERQRATKLSQQQSMYQHQQEKVAHLQSYIDRFRAQATKAKQAQSRIKMLERMELIAPAHVDNPFH
FSFRTPESLPDPLLRMDKVSAGYGENTVLQSIKLNLVPGSRIGLLGRNGAGKSTLIKLLAGTLEPQSGEIALSKGIKLGYFAQHQLEFLRADESPLQHMSRLAPKESEQQLRDYLGGYGFQGDQV
TDPTARFSGGEKARLVLALIIWQRPNLLLLDEPTNHLDLDMRQALTEALIDFEGALVVVSHDRHLLRSTTDDLYLVHDGKVEQFDGDLEDYQQWLVDSQRQQSQQDNPAKELSGNSAQQRK
DQKRRDAEFRTQTQPLRKQIMTLEKQMDKLSTELAAIEAQLADSALYDIARKADLTQCLLQQTQVKSKLEETEMQWLDVQEQLEILTKEFSDADI

Strain: Y. pestis pestoides F
Gene: YPDSF_0114

Classification: putative ABC transporter ATP-binding protein
Boundaries of gene: complement (132570..134492)

Oligo evidence:

taccttggtattaatttcccatgaccgcgatttccttgatcctattatcgataagattttgcatattgaa
tgttatcgacggtgaccgcgaatatcgccagttggaggccgaacttcaggctgccaatgaaaaaaatgat

Peptide evidence:

AASLLHGLGFSQEK
ADESPLQHMSR
AIEYVIDGDREYRQLEAELQAANEK
DYLGGYGFQGDQVTDPTAR
ELSGNSAQQR
LGYFAQHQLEFLR
LSQQQSMYQHQQEK
LSTELAAIEAQLADSALYDIAR
VAHLQSYIDR
VLLDNATATINPGQK

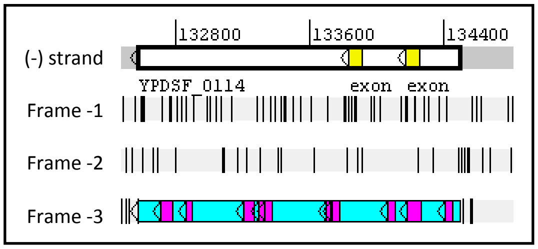

Current sequence (peptide evidence in red): 640aa
MIVFSSLQIRRGTRVLLDNATATINPGQKVGLVGKNGCGKSTLLALLKGELSADGGNVTFPNNWALAWVNQETPALDIPAIEYVIDGDREYRQLEAELQAANEKNDGHAIATVHGKLDAIHA
WTIQSRAASLLHGLGFSQEKLQQPVRSFSGGWRMRLNLAQALVCRSDLLLLDEPTNHLDLDAVIWLEKWLKSYTGTLVLISHDRDFLDPIIDKILHIEQQTLNEYTGNYSSFERQRATKLSQQQ
SMYQHQQEKVAHLQSYIDRFRAQATKAKQAQSRIKMLERMELIAPAHVDNPFHFSFRTPESLPDPLLRMDKVSAGYGENTVLQSIKLNLVPGSRIGLLGRNGAGKSTLIKLLAGTLEPQSGEIA
LSKGIKLGYFAQHQLEFLRADESPLQHMSRLAPKESEQQLRDYLGGYGFQGDQVTDPTARFSGGEKARLVLALIIWQRPNLLLLDEPTNHLDLDMRQALTEALIDFEGALVVVSHDRHLLRST
TDDLYLVHDGKVEQFDGDLEDYQQWLVDSQRQQSQQDNPAKELSGNSAQQRKDQKRRDAEFRTQTQPLRKQIMTLEKQMDKLSTELAAIEAQLADSALYDIARKADLTQCLLQQTQVKS
KLEETEMQWLDVQEQLEILTKEFSDADI

Strain: Y. pseudotuberculosis PB1/+
Gene: YPTS_3908

Classification: putative ABC transporter ATP-binding protein
Boundaries of gene: 4341544..4343466

Oligo evidence:

tgttatcgacggtgaccgcgaatatcgccagttggaggccgaacttcaggctgccaatgaaaaaaatgat
taccttggtattaatttcccatgaccgcgatttccttgatcctattatcgataagattttgcatattgaa

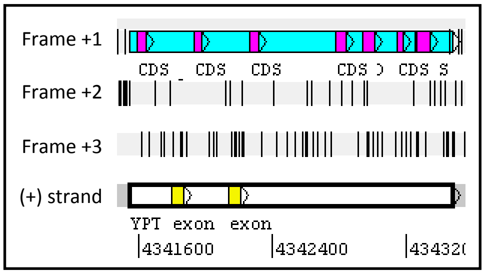

Peptide evidence:

AASLLHGLGFSQEK
DYLGGYGFQGDQVTDPTAR
ELSGNSAQQR
LSQQQSMYQHQQEK
LSTELAAIEAQLADSALYDIAR
QALTEALIDFEGALVVVSHDR
QMDKLSTELAAIEAQLADSALYDIAR
VLLDNATATINPGQK

Current sequence (peptide evidence in red): 640aa
MIVFSSLQIRRGTRVLLDNATATINPGQKVGLVGKNGCGKSTLLALLKGELSADGGNVTFPNNWALAWVNQETPALDIPAIEYVIDGDREYRQLEAELQAANEKNDGHAIATVHGKLDAIHA
WTIQSRAASLLHGLGFSQEKLQQPVRSFSGGWRMRLNLAQALVCRSDLLLLDEPTNHLDLDAVIWLEKWLKSYTGTLVLISHDRDFLDPIIDKILHIEQQTLNEYTGNYSSFERQRATKLSQQQ
SMYQHQQEKVAHLQSYIDRFRAQATKAKQAQSRIKMLERMELIAPAHVDNPFHFSFRTPESLPDPLLRMDKVSAGYGENTVLQSIKLNLVPGSRIGLLGRNGAGKSTLIKLLAGTLEPQSGEIA
LSKGIKLGYFAQHQLEFLRADESPLQHMSRLAPKESEQQLRDYLGGYGFQGDQVTDPTARFSGGEKARLVLALIIWQRPNLLLLDEPTNHLDLDMRQALTEALIDFEGALVVVSHDRHLLRST
TDDLYLVHDGKVEQFDGDLEDYQQWLVDSQRQQSQQDNPAKELSGNSAQQRKDQKRRDAEFRTQTQPLRKQIMTLEKQMDKLSTELAAIEAQLADSALYDIARKADLTQCLLQQTQVKS
KLEETEMQWLDVQEQLEILTKEFSDADI

Error 7

Strain: Y. pestis CO92
Gene: YPO0247

Classification: expressed pseudogene
Boundaries of gene: complement (245195..245725)

Oligo evidence:

agatggaacaataatagaagatgatgtaatgattggtgcaggtagcctcattactccaggtaaacgctta
tcaagtcatcattggggcacgttcaaatatccaagacggtagtgtgctacatgttacccaccaatctgag
ggtaaacgagtcatgatcgatagatcgagtgtgatcattggcaatgttattttaggtgatgatgtcagtg

Peptide evidence:

QIRPLTPLELEGLLYSAGNYVR

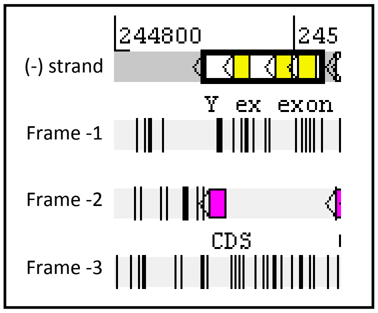

Proposed sequence (peptide evidence in red): 176aa
IRPYLHHSPTLGKRVMIDRSSVIIGNVILGDDVSVWPLVAIRGDVNQVIIGARSNIQDGSVLHVTHQSEHNPEGYPLIIGEDVTIGHKAMLHGCTIGNRVLVGMGSILLDGTIIEDDVMIGAGSLI
TPGKRLVSGYLYVGSPAKQIRPLTPLELEGLLYSAGNYVRWKDDYLAESK

Strain: Y. pestis pestoides F
Gene: YPDSF_0170

Classification: hypothetical protein
Boundaries of gene: complement (171706..172248)

Oligo evidence:

agatggaacaataatagaagatgatgtaatgattggtgcaggtagcctcattactccaggtaaacgctta
tcaagtcatcattggggcacgttcaaatatccaagacggtagtgtgctacatgttacccaccaatctgag
ggtaaacgagtcatgatcgatagatcgagtgtgatcattggcaatgttattttaggtgatgatgtcagtg
ctctatccgcccttatcttcatcattcaccgactttaggtaaacgagtcatgatcgatagatcgagtgtg

Peptide evidence:

QIRPLTPLELEGLLYSAGNYVR
LVSGYLYVGSPAK

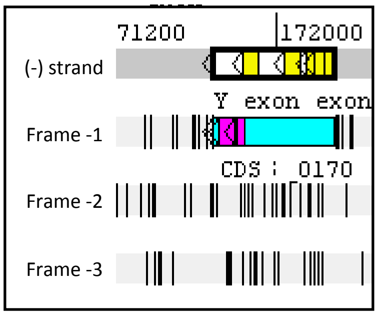

Current sequence (peptide evidence in red): 180aa
MCDSIRPYLHHSPTLGKRVMIDRSSVIIGNVILGDDVSVWPLVAIRGDVNQVIIGARSNIQDGSVLHVTHQSEHNPEGYPLIIGEDVTIGHKAMLHGCTIGNRVLVGMGSILLDGTIIEDDVMIG
AGSLITPGKRLVSGYLYVGSPAKQIRPLTPLELEGLLYSAGNYVRWKDDYLAESK

Strain: Y. pseudotuberculosis PB1/+
Gene: YPTS_3851

Classification: hexapaptide repeat-containing transferase
Boundaries of gene: 4304535..4305077

Oligo evidence:

ctctatccgcccttatcttcatcattcaccgactttaggtaaacgagtcatgatcgatagatcgagtgtg
ggtaaacgagtcatgatcgatagatcgagtgtgatcattggcaatgttattttaggtgatgatgtcagtg
tcaagtcatcattggggcacgttcaaatatccaagacggtagtgtgctacatgttacccaccaatctgag

Peptide evidence:

LVSGYLYVGSPAK
QIRPLTPLELEGLLYSAGNYVR
SSVIIGNVILGDDVSVWPLVAIR
VLVGMGSILLDGTIIEDDVMIGAGSLIAPGKR

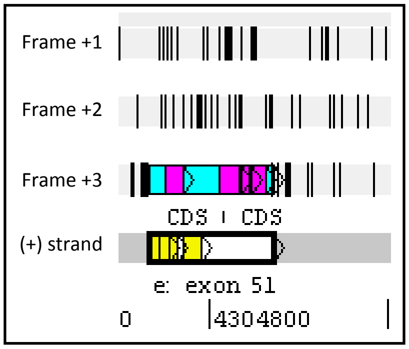

Current sequence (peptide evidence in red): 180aa
MCDSIRPYLHHSPTLGKRVMIDRSSVIIGNVILGDDVSVWPLVAIRGDVNQVIIGARSNIQDGSVLHVTHQSEHNPEGYPLIIGEDVTIGHKAMLHGCTIGNRVLVGMGSILLDGTIIEDDVMIG
AGSLIAPGKRLVSGYLYVGSPAKQIRPLTPLELEGLLYSAGNYVRWKDDYLAESK

Error 8

Strain: Y. pestis CO92
Gene: YPO0414

Classification: expressed pseudogene
Boundaries of gene: Frame+1: 431515..431733
Frame+2: 431735..432502

Oligo evidence:

atgagtgaagaggaactgctcgctcgcgttgcgtggttttattatcacgatggtttaacacaaggggata
atcacagcaggtccgtttagtcacgttatccggcggtgtcggctcctatatgacggggattggtcagttg

Peptide evidence:

DGLTQGDIGELLGLAR
ASNAHVADTFR
ASNAHVADTFRQENSVR
ILPSLASLSISSR

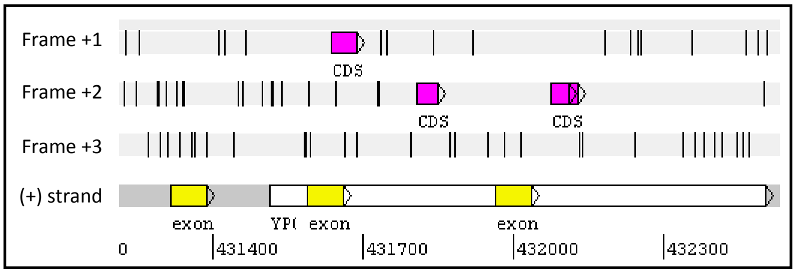

Proposed sequence (peptide evidence in red):
Frame+1: 74aa
MIKEKPAVKEKPLSSNDEYKYPGNSMSEEELLARVAWFYYHDGLTQGDIGELLGLARLKVSRLLEKGRQSGVI

Frame+2: 255aa
INSRYEGCLELENTLQQHFGLKHIRILPSLASLSISSRLGIGAAHLLMALIQPQQLLAVGFGETTMCALQHLSGFIASQQVRLVTLSGGVGSYMTGIGQLDAACQVSIIPAPLRASNAHVADTFRQENSVRDVMLAACAADIAVVGIGSVNQQKEATILRSGYISEGEQLMFSRKGAVGDILGYFIQADGALVPDMQIHQELIGISLADLTTIPTVIGVAGGVEKADVIVAALRGRYVNALVTDEVTARAIINLL

Strain: Y. pestis pestoides F
Gene: YPDSF_3217

Classification: transcriptional repressor
Boundaries of gene: complement (3666459..3667379)

Oligo evidence:

atcacagcaggtccgtttagtcacgttatccggcggtgtcggctcctatatgacggggattggtcagttg
atgagtgaagaggaactgctcgctcgcgttgcgtggttttattatcacgatggtttaacacaaggggata

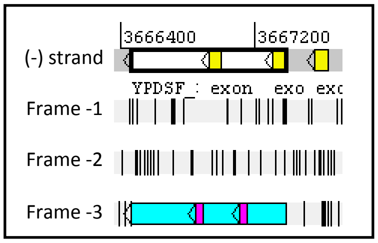

Peptide evidence:

ASNAHVADTFR
ILPSLASLSISSR

Current sequence (peptide evidence in red): 306aa
MSEEELLARVAWFYYHDGLTQGDIGELLGLARLKVSRLLEKGRQSGVIRVQINSRYEGCLELENTLQQHFGLKHIRILPSLASLSISSRLGIGAAHLLMALIQPQQLLAVGFGETTMCALQHLSGFIASQQVRLVTLSGGVGSYMTGIGQLDAACQVSIIPAPLRASNAHVADTFRQENSVRDVMLAACAADIAVVGIGSVNQQKEATILRSGYISEGEQLMFSRKGAVGDILGYFIQADGALVPDMQIHQELIGISLADLTTIPTVIGVAGGVEKADVIVAALRGRYVNALVTDEVTARAIINLL

Strain: Y. pseudotuberculosis PB1/+
Gene: YPTS_0577

Classification: DeoR family transcriptional regulator
Boundaries of gene: 635362..636357

Oligo evidence:

atgagtgaagaggaactgctcgctcgcgttgcgtggttttattatcacgatggtttaacacaaggggata

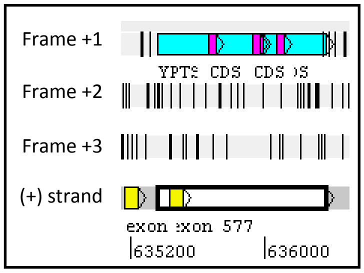

Peptide evidence:

ASSAHVADTFR
ASSAHVADTFRQENSVR
ILPSLASLSISSR
SGYISEGEQLMFSR

Current sequence (peptide evidence in red): 331aa
MIKEKPAVKEKPLSSNDEYKYPGNSMSEEELLARVAWFYYHDGLTQGDIGELLGLARLKVSRLLEKGRQSGVIRVQINSRYEGCLELENTLQQHFGLKHIRILPSLASLSISSRLGIGAAHLLMALIQPQQLLAVGFGETTMCALQHLSGFIASQQVRLVTLSGGVGSYMTGIGQLDAACQVSIIPAPLRASSAHVADTFRQENSVRDVMLAACAADIAVVGIGSVNQQKEATILRSGYISEGEQLMFSRKGAVGDILGYFIQADGALVPDMQIHQELIGISLADLTTIPTVIGVAGGVEKADVIVAALRGRYVNALVTDEVTARAIINLL

Error 9

Strain: Y. pestis CO92
Gene: YPO0437

Classification: expressed pseudogene (deoA)
Boundaries of gene: Frame+3:458100:458630
Frame+2: 458633..459511

Oligo evidence:

tattttcccggatgataatgcgttccgcaaaattattatgttggtgtggcgattatcggccaaaccagct
ggcggcagaggttgtgcgtgcggccatcaccttagggaataacacgccagaagaaacgccagtgatttat

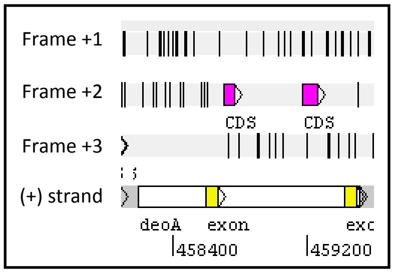

Peptide evidence:

DITATVDSIPLITASILAK
YDSYLPVATLSKPVFAEQTGIITAMDTR

Proposed sequence (peptide evidence in red):
Current annotation underlined
Frame+3: 177aa
MHSRLKACCVAISESACGRLSSHIPVKQGDALFLAQEIIRKKRDGQPLSEEEIRFFINGIRDNVVSEGQIAALAMTIYFHDMSMPERVALTMAMRDSGTVLNWKSLNLNGPLVDKHSTGGVG
DVTSLMLGPMVAACGGYVPMISGRGLGHTGGTLDKLEAIPGFDIFPDDNAFRKII

Frame+2: 292aa
VGVAIIGQTSSLAPADKRFYATRDITATVDSIPLITASILAKKLAEGLDALVMDVKVGSGAFMPTYSLSADLAQAIVGVANGAGCKTTALLTDMNQVLASSAGNGVEVREAVRFLTGEYRNPRL
LEVTMALCVEMLLSGGLAHDEADARAKLQAVLDNGKAAEVFGRMVAAQKGPADFVERYDSYLPVATLSKPVFAEQTGIITAMDTRALGMAVVALGGGRRRATDPIDYSVGLTEMARLGTR
VDGQQPLAVIHANNEDDWQQAAEVVRAAITLGNNTPEETPVIYRRITE

Strain: Y. pestis pestoides F
Gene: YPDSF_3196

Classification: annotated as deoA
Boundaries of gene: complement (3639439..3640854)

Oligo evidence:

ggcggcagaggttgtgcgtgcggccatcaccttagggaataacacgccagaagaaacgccagtgatttat
tattttcccggatgataatgcgttccgcaaaattattcagaatgttggtgtggcgattatcggccaaacc

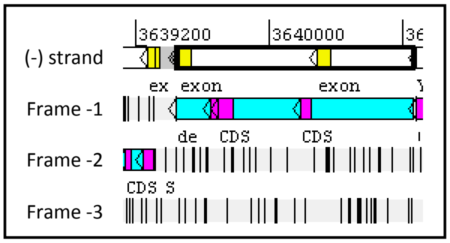

Peptide evidence:

ALGMAVVALGGGR
DITATVDSIPLITASILAK
YDSYLPVATLSKPVFAEQTGIITAMDTR

Current sequence (peptide evidence in red): 471aa
MHSRLKACCVAISESACGRLSSHIPVKQGDALFLAQEIIRKKRDGQPLSEEEIRFFINGIRDNVVSEGQIAALAMTIYFHDMSMPERVALTMAMRDSGTVLNWKSLNLNGPLVDKHSTGGVG
DVTSLMLGPMVAACGGYVPMISGRGLGHTGGTLDKLEAIPGFDIFPDDNAFRKIIQNVGVAIIGQTSSLAPADKRFYATRDITATVDSIPLITASILAKKLAEGLDALVMDVKVGSGAFMPTYSL
SADLAQAIVGVANGAGCKTTALLTDMNQVLASSAGNGVEVREAVRFLTGEYRNPRLLEVTMALCVEMLLSGGLAHDEADARAKLQAVLDNGKAAEVFGRMVAAQKGPADFVERYDSYLP
VATLSKPVFAEQTGIITAMDTRALGMAVVALGGGRRRATDPIDYSVGLTEMARLGTRVDGQQPLAVIHANNEDDWQQAAEVVRAAITLGNNTPEETPVIYRRITE

Strain: Y. pseudotuberculosis PB1/+
Gene: YPTS_0604

Classification: annotated as deoA
Boundaries of gene: 667792..669207

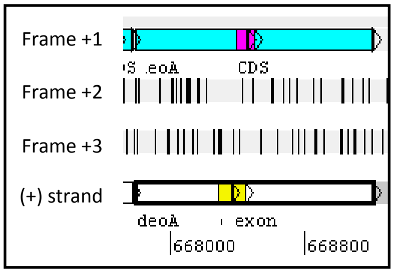

Oligo evidence:

tattttcccggatgataatgcgttccgcaaaattattcagaatgttggtgtggcgattatcggccaaacc
ataagcgtttttacgcgacccgcgatattacggcaacagttgattctattccattgattacggcctctat

Peptide evidence:

DITATVDSIPLITASILAK
KLAEGLDALVMDVK

Current sequence (peptide evidence in red): 471aa
MHCRLKACRVAISESACGRLSSHIQVKQGDALFLAQEIIRKKRDGQPLSEEEIRFFINGIRDNVVSEGQIAALAMTIYFHDMSMPERVALTMAMRDSGTVLNWKSLNLNGPLVDKHSTGGVG
DVTSLMLGPMVAACGGYVPMISGRGLGHTGGTLDKLEAIPGFDIFPDDNAFRKIIQNVGVAIIGQTSSLAPADKRFYATRDITATVDSIPLITASILAKKLAEGLDALVMDVKVGSGAFMPTYSL
SADLAQAIVGVANGAGCKTTALLTDMNQVLASSAGNGVEVREAVRFLTGEYRNPRLLEVTMALCVEMLLSGGLAHDEADARAKLQAVLDNGKAAEVFGRMVAAQKGPVDFVERYDSYLP
VATLSKPVFAEQTGIITAMDTRALGMAVVALGGGRRRATDPIDYSVGLTEMARLGTRVDGQQPLAVIHANNEDDWQQAAEAVRAAITLGNNAPEETPVIYRRITE

Error 10

Strain: Y. pestis CO92
Gene: YPO0446

Classification: expressed pseudogene
Boundaries of gene: Frame-1: complement (469162..469731)
Frame-2: complement (468068..469165)

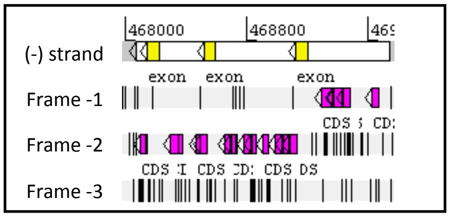

Oligo evidence:

gccacccacatcatcgattatcaagatgagggtaaggtagaattcttcgagggtaactttactgaatacg
gatacggtgcaactggcgtcggttgatcagttccgtgacaacatggatgacagcaaaaccgtttgggaag
aaaccagacatgctgctgctggacgaaccaaccacttggatgcagaatctgtggcctggctggagcgctt

Peptide evidence:

DEVYALYADPDADFDK
EQGELEAIIQSHDGHNLDNQLER
ESVEEAVGEVKR
LDEVYALYADPDADFDK
LDEVYALYADPDADFDKLAK
NISLSFFPGAK
FEELNSVEYQKR
FLHDYEGTVVAITHDR
GAIVGIIGPNGAGK
GEGIPWEGNYSSWLEQK
HDYEGTVVAITHDR
IDDLTFALPK
IGNFELPSR
KSIEKELEWVR
LLIDDLTFALPK
LLQVGGNMLLLDEPTNDLDIETLR
MLLLDEPTNDLDIETLR
NETSELFIPPGPR
RNETSELFIPPGPR
RTLGAEALEPHR
SIEKELEWVR
TLGAEALEPHR
TVWEEVSGGQDIMK
YFLDNVAGWILELDR
YFLDNVAGWILELDRGEGIPWEGNYSSWLEQK

Proposed sequence (peptide evidence in red):

Frame-1: 190aa

MAQYVYTMHRVGKVVPPKRHILKNISLSFFPGAKIGVLGLNGSGKSTLLRIMAGLDTEIEGEARPQLGIKIGYLPQEPKLNLEQTVRESVEEAVGEVKRALTRLDEVYALYADPDADFDKLAKEQGELEAIIQSHDGHNLDNQLERAADALRLPPWDAKIANLSGGERRRVAICRLLLEKPDMLLLDEPT

Frame-2: 365aa

NHLDAESVAWLERFLHDYEGTVVAITHDRYFLDNVAGWILELDRGEGIPWEGNYSSWLEQKNARLAQEASSEAARRKSIEKELEWVRQNPKGRQAKGKARLARFEELNSVEYQKRNETSELFIPPGPRLGDKVLEVEHLSKSYGDRLLIDDLTFALPKGAIVGIIGPNGAGKSTLFRMLSGQEQPDSGTISLGDTVQLASVDQFRDNMDDSKTVWEEVSGGQDIMKIGNFELPSRAYVGRFNFKGVDQGKRIGELSGGERGRIHLAKLLQVGGNMLLLDEPTNDLDIETLRALENALLEFPGCAMVISHDRWFLDRIATHIIDYQDEGKVEFFEGNFTEYEEWKKRTLGAEALEPHRIKYKKMTK

Strain: Y. pestis pestoides F
Gene: YPDSF_3187

Classification: putative ABC transporter ATP-binding protein
Boundaries of gene: 3629306..3630973

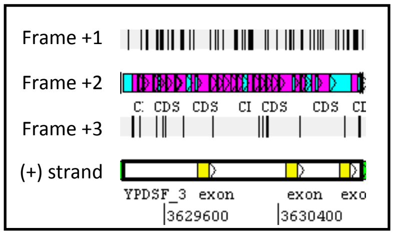

Oligo evidence:

actgctggaaaaaccagacatgctgctgctggacgaaccaaccaaccacttggatgcagaatctgtggcc
gatacggtgcaactggcgtcggttgatcagttccgtgacaacatggatgacagcaaaaccgtttgggaag
gccacccacatcatcgattatcaagatgagggtaaggtagaattcttcgagggtaactttactgaatacg

Peptide evidence:

ALTRLDEVYALYADPDADFDKLAK
DEVYALYADPDADFDK
DNVAGWILELDR
DNVAGWILELDRGEGIPWEGNYSSWLEQK
EQGELEAIIQSHDGHNLDNQLER
ESVEEAVGEVK
ESVEEAVGEVKR
FEELNSVEYQK
FEELNSVEYQKR
FLHDYEGTVVAITHDR
GAIVGIIGPNGAGK
GEGIPWEGNYSSWLEQK
HDYEGTVVAITHDR
IANLSGGER
IDDLTFALPK
IGELSGGER
IGNFELPSR
IGVLGLNGSGK
IGYLPQEPK
IMAGLDTEIEGEARPQLGIK
KSIEKELEWVR
LAQEASSEAAR
LDEVYALYADPDADFDK
LDEVYALYADPDADFDKLAK
LGDKVLEVEHLSK
LHDYEGTVVAITHDR
LLIDDLTFALPK
LLLEKPDMLLLDEPTNHLDAESVAWLER
LLQVGGNMLLLDEPTNDLDIETLR
MLSGQEQPDSGTISLGDTVQLASVDQFR
MVLGIISYSR
NETSELFIPPGPR
NISLSFFPGAK
RNETSELFIPPGPR
RTLGAEALEPHR
SIEKELEWVR
TLGAEALEPHR
TVWEEVSGGQDIMK
VLEVEHLSK
YFLDNVAGWILELDR
YFLDNVAGWILELDRGEGIPWEGNYSSWLEQK

Current sequence (peptide evidence in red): 555aa

VAQYVYTMHRVGKVVPPKRHILKNISLSFFPGAKIGVLGLNGSGKSTLLRIMAGLDTEIEGEARPQLGIKIGYLPQEPKLNLEQTVRESVEEAVGEVKRALTRLDEVYALYADPDADFDKLAKEQGELEAIIQSHDGHNLDNQLERAADALRLPPWDAKIANLSGGERRRVAICRLLLEKPDMLLLDEPTNHLDAESVAWLERFLHDYEGTVVAITHDRYFLDNVAGWILELDRGEGIPWEGNYSSWLEQKNARLAQEASSEAARRKSIEKELEWVRQNPKGRQAKGKARLARFEELNSVEYQKRNETSELFIPPGPRLGDKVLEVEHLSKSYGDRLLIDDLTFALPKGAIVGIIGPNGAGKSTLFRMLSGQEQPDSGTISLGDTVQLASVDQFRDNMDDSKTVWEEVSGGQDIMKIGNFELPSRAYVGRFNFKGVDQGKRIGELSGGERGRIHLAKLLQVGGNMLLLDEPTNDLDIETLRALENALLEFPGCAMVISHDRWFLDRIATHIIDYQDEGKVEFFEGNFTEYEEWKKRTLGAEALEPHRIKYKKMTK

Strain: Y. pseudotuberculosis PB1/+
Gene: YPTS_0612

Classification: putative ABC transporter ATP-binding protein
Boundaries of gene: complement (677816..679483)

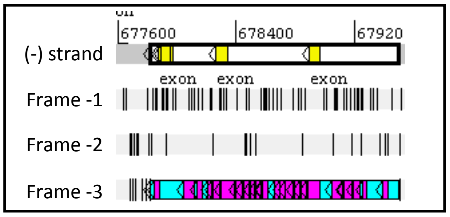

Oligo evidence:

attatcaagatgagggtaaggtagaattcttcgagggtaactttactgaatacgaagagtggaagaaacg
gccacccacatcatcgattatcaagatgagggtaaggtagaattcttcgagggtaactttactgaatacg
gatacggtgcaactggcgtcggttgatcagttccgtgacaacatggatgacagcaaaaccgtttgggaag
actgctggaaaaaccagacatgctgctgctggacgaaccaaccaaccacttggatgcagaatctgtggcc

Peptide evidence:

DEVYALYADPDADFDK
EQGELEAIIQSHDGHNLDNQLER
ESVEEAVGEVK
ESVEEAVGEVKR
FEELNSVEYQK
FEELNSVEYQKR
FLHDYEGTVVAITHDR
GAIVGIIGPNGAGK
GEGIPWEGNYSSWLEQK
HDYEGTVVAITHDR
IGELSGGER
IGNFELPSR
IGYLPQEPK
KSIEKELEWVR
LAQEASSEAAR
LDEVYALYADPDADFDK
LDEVYALYADPDADFDKLAK
LLIDDLTFALPK
LLLEKPDMLLLDEPTNHLDAESVAWLER
LLQVGGNMLLLDEPTNDLDIETLR
MLSGQEQPDSGTISLGDTVQLASVDQFR
NETSELFIPPGPR
NISLSFFPGAK
RIGELSGGER
RNETSELFIPPGPR
RTLGAEALEPHR
SIEKELEWVR
TLGAEALEPHR
TVWEEVSGGQDIMK
VLEVEHLSK
YFLDNVAGWILELDR
YFLDNVAGWILELDRGEGIPWEGNYSSWLEQK

Current sequence (peptide evidence in red): 555aa

VAQYVYTMHRVGKVVPPKRHILKNISLSFFPGAKIGVLGLNGSGKSTLLRIMAGLDTEIEGEARPQPGIKIGYLPQEPKLNLEQTVRESVEEAVGEVKRALTRLDEVYALYADPDADFDKLAKEQGELEAIIQSHDGHNLDNQLERAADALRLPPWDAKIANLSGGERRRVAICRLLLEKPDMLLLDEPTNHLDAESVAWLERFLHDYEGTVVAITHDRYFLDNVAGWILELDRGEGIPWEGNYSSWLEQKNARLAQEASSEAARRKSIEKELEWVRQNPKGRQAKGKARLARFEELNSVEYQKRNETSELFIPPGPRLGDKVLEVEHLSKSYGDRLLIDDLTFALPKGAIVGIIGPNGAGKSTLFRMLSGQEQPDSGTISLGDTVQLASVDQFRDNMDDSKTVWEEVSGGQDIMKIGNFELPSRAYVGRFNFKGVDQGKRIGELSGGERGRIHLAKLLQVGGNMLLLDEPTNDLDIETLRALENALLEFPGCAMVISHDRWFLDRIATHIIDYQDEGKVEFFEGNFTEYEEWKKRTLGAEALEPHRIKYKKMTK

Error 11

Strain: Y. pestis CO92
Gene: YPO0674

Classification: expressed pseudogene
Boundaries of gene: Frame+3: 735996..737525
Frame+2: 737525..738337

Oligo evidence:

ttttactgccttcatatgaaaaagtgaaggcggacaaagtgttgtatgcccatacctcgcggattttgca
actgtttgatacttactcgaaacaggccggtaaagagcagtatttgatcccttatttcatctccgcacat

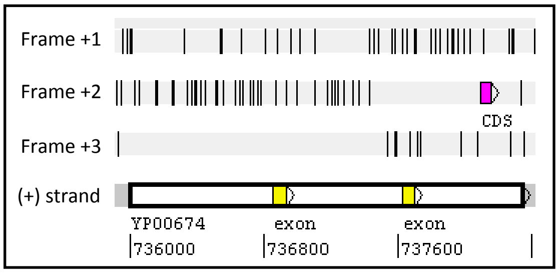

Peptide evidence:

QRQPSNRAPAASAGKK

Proposed sequence (peptide evidence in red):
Frame3: 510aa
MSISLIQPERDLFSYQPYWAECYGTAPFLPMSREEMDILGWDSCDIIVITGDAYVDHPSFGMAIVGRMLEAQGFRVGIIAQPDWTNKHDFMRLGEPNLFFGVTAGNMDSMINRYTADRKL
RHDDAYTPDNQSGKRPDRATLVYSQRCKEAYSHVPVLLGGIEASLRRIAHYDYWSDTVRRSVIVDAKADMLVYGNGERPLVEVAHRLAAGEKITDIQDVRNTVVMRKTPLPGWSGVDSTRL
DKPGRIEAIPNPYGEDLPCATDDISIPEAKPITVRAAKPKPWEKTYVLLPSYEKVKADKVLYAHTSRILHHETNPGCARALMQKHGDRYIWINPPAIPLSTEEMDSVFALPYQRVPHPSYGKSPIP
AYDMIRFSINIMRGCYGGCSFCSITEHEGRIIQSRSEDSIIREIEEIRDKVPGFTGIISDLGGPTANMYMLRCQSPRAEQTCRRASCVYPEICPHMDTNHQPTISLYRRARDLKGIKKILIASGVRYD
LAVEDPRYIKELASH

Frame2: 271aa
HVGGYLKIAPEHTEEGPLSKMMKPGMGSYQRFKELFDTYSKQAGKEQYLIPYFISAHPGTEDKDMVNLALWLKKNRFRLDQVQNFYPSPLANSTTMYYTGKNPLAKVDYKSEEVVVPKGDR
QRRLHKALLRYHDPANWPMLRSALEDMGLQHLIGARRECLVPAPTLEEQREARRALRHHTPALTKHTSITRQRQPSNRAPAASAGKKAPTVANGTSSAHSTSANQSTSANQSTSAAHSTLA
TKKSAGKTGVNKAAVNKPSAGSRGKNRQH

Strain: Y. pestis pestoides F
Gene: YPDSF_0458/YPDSF_0459

Classification: Both genes annotated as hypothetical proteins
Boundaries of gene: YPDSF_0458 Frame+1: 517558..519087
YPDSF_0459 Frame+3: 519087..519902

Oligo evidence:

ttttactgccttcatatgaaaaagtgaaggcggacaaagtgttgtatgcccatacctcgcggattttgca
actgtttgatacttactcgaaacaggccggtaaagagcagtatttgatcccttatttcatctccgcacat

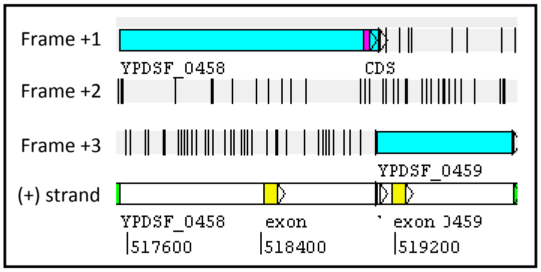

Peptide evidence:

KILIASGVR

Current sequence (peptide evidence in red):
Frame+1: 510aa (YPDSF_0458)
MSISLIQPERDLFSYQPYWAECYGTAPFLPMSREEMDILGWDSCDIIVITGDAYVDHPSFGMAIVGRMLEAQGFRVGIIAQPDWTNKHDFMRLGEPNLFFGVTAGNMDSMINRYTADRKL
RHDDAYTPDNQSGKRPDRATLVYSQRCKEAYSHVPVLLGGIEASLRRIAHYDYWSDTVRRSVIVDAKADMLVYGNGERPLVEVAHRLAAGEKITDIQDVRNTVVMRKTPLPGWSGVDSTRL
DKPGRIEAIPNPYGEDLPCATDDISIPEAKPITVRAAKPKPWEKTYVLLPSYEKVKADKVLYAHTSRILHHETNPGCARALMQKHGDRYIWINPPAIPLSTEEMDSVFALPYQRVPHPSYGKSPIP
AYDMIRFSINIMRGCYGGCSFCSITEHEGRIIQSRSEDSIIREIEEIRDKVPGFTGIISDLGGPTANMYMLRCQSPRAEQTCRRASCVYPEICPHMDTNHQPTISLYRRARDLKGIKKILIASGVRYD
LAVEDPRYIKELASH

Frame+3: 271aa (YPDSF_0459)
HVGGYLKIAPEHTEEGPLSKMMKPGMGSYQRFKELFDTYSKQAGKEQYLIPYFISAHPGTEDKDMVNLALWLKKNRFRLDQVQNFYPSPLANSTTMYYTGKNPLAKVDYKSEEVVVPKGDR
QRRLHKALLRYHDPANWPMLRSALEDMGLQHLIGARRECLVPAPTLEEQREARRALRHHTPALTKHTSITRQRQPSNRAPAASAGKKAPTVANGTSSAHSTSANQSTSANQSTSAAHSTLA
TKKSAGKTGVNKAAVNKPSAGSRGKNRQH

Strain: Y. pseudotuberculosis PB1/+
Gene: YPTS_3529

Classification: annotated as hypothetical protein
Boundaries of gene: complement (3934430..3936793)

Oligo evidence:

actgtttgatacttactcgaaacaggccggtaaagagcagtatttgatcccttatttcatctccgcacat

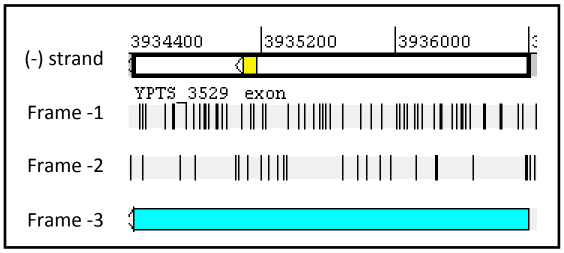

Peptide evidence:

NA

Current sequence (peptide evidence in red): 787aa
MSISLIQPERDLFSYQPYWAECYGTAPFLPMSREEMDILGWDSCDIIVITGDAYVDHPSFGMAIVGRMLEAQGFRVGIIAQPDWTNKHDFMRLGEPNLFFGVTAGNMDSMINRYTADRKL
RHDDAYTPDNQSGKRPDRATLVYSQRCKEAYSHVPVLLGGIEASLRRIAHYDYWSDTVRRSVIVDAKADMLVYGNGERPLVEVAHRLAAGEKITDIQDVRNTVVMRKTPLPGWSGVDSTRL
DKPGRIEAIPNPYGEDLPCATDDISIPEAKPITVRAAKPKPWEKTYVLLPSYEKVKADKVLYAHTSRILHHETNPGCARALMQKHGDRYIWINPPAIPLSTEEMDSVFALPYQRVPHPSYGKSPIP
AYDMIRFSINIMRGCYGGCSFCSITEHEGRIIQSRSEDSIIREIEEIRDKVPGFTGIISDLGGPTANMYMLRCQSPRAEQTCRRASCVYPEICPHMDTNHQPTISLYRRARDLKGIKKILIASGVRYD
LAVEDPRYIKELASHHVGGYLKIAPEHTEEGPLSKMMKPGMGSYQRFKELFDTYSKQAGKEQYLIPYFISAHPGTEDKDMVNLALWLKKNRFRLDQVQNFYPSPLANSTTMYYTGKNPLAKV
DYKSEEVVVPKGDRQRRLHKALLRYHDPANWPMLRSALEDMGLQHLIGARRECLVPAPTLEEQREARRALRHHTPALTKHTSITRQRQPSNRAPAASAGKKAPTVANGTSSAHSTSANQST
SANQSTSANQSTSAAHSMLATKKSAGKTGVNKAAVNKPSAGSRGKNRQH

Error 12

Strain: Y. pestis CO92
Gene: YPO1195

Classification: expressed pseudogene
Boundaries of gene: 1345498..1346430

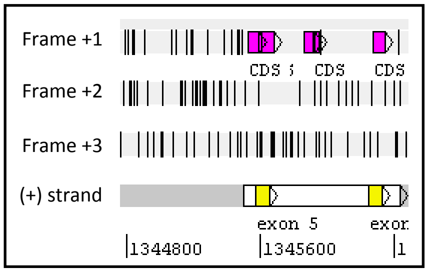

Oligo evidence:

ggcagataacgtccgtgtaggctctaaaattgataccgaaggctccttgctgggcaatattattgtgcag
atcatccgtgaagttactctgaaagctcacccgaacatcccggctttgcttaatccggtattcgccacac

Peptide evidence:

DLATANNLR
IDTEGSLLGNIIVQVLEANGIK
IFAVAAIMLSASSGISHAAD
LVWLSPAPANNTWTIAVR
VGSKIDTEGSLLGNIIVQVLEANGIK
AHPNIPALLNPVFATLDGPTLQK

Proposed sequence (peptide evidence in red): 310aa
MALFLTRSRIFAVAAIMLSASSGISHAADNVRVGSKIDTEGSLLGNIIVQVLEANGIKTTNKSQLGATKVVRGAITAGEIDIYPEYTGNGAFFFSDEQDPAWKSAKAGYEKVKALDYEKNKLVWL
SPAPANNTWTIAVRKDLATANNLRTLDDLG+WINGGGQFKLAASAEFIERPDALPAFQQAYGFTLNQDQLLSLAGGDTAVTIKAAAEQISGVNAAMAYGTDGPVAALGLQTLEDTKGVQPI
YAPAPIIREVTLKAHPNIPALLNPVFATLDGPTLQKLNARIAVEGQDAKKVAANYLKDNGFIKN

Strain: Y. pestis pestoides F
Gene: YPDSF_2500

Classification: annotated as hypothetical protein
Boundaries of gene: complement (2847097..2848029)

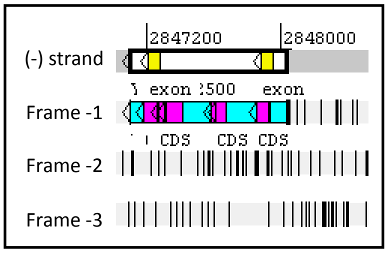

Oligo evidence:

atcatccgtgaagttactctgaaagctcacccgaacatcccggctttgcttaatccggtattcgccacac
ggcagataacgtccgtgtaggctctaaaattgataccgaaggctccttgctgggcaatattattgtgcag

Peptide evidence:

AAAEQISGVNAAMAYGTDGPVAALGLQTLEDTK
AHPNIPALLNPVFATLDGPTLQK
DLATANNLR
GVQPIYAPAPIIR
IDTEGSLLGNIIVQVLEANGIK
LVWLSPAPANNTWTIAVR
VGSKIDTEGSLLGNIIVQVLEANGIK

Current sequence (peptide evidence in red): 310aa
MALFLTRSRIFAVAAIMLSASSGISHAADNVRVGSKIDTEGSLLGNIIVQVLEANGIKTTNKSQLGATKVVRGAITAGEIDIYPEYTGNGAFFFSDEQDPAWKSAKAGYEKVKALDYEKNKLVWL
SPAPANNTWTIAVRKDLATANNLRTLDDLGKWINGGGQFKLAASAEFIERPDALPAFQQAYGFTLNQDQLLSLAGGDTAVTIKAAAEQISGVNAAMAYGTDGPVAALGLQTLEDTKGVQPI
YAPAPIIREVTLKAHPNIPALLNPVFATLDGPTLQKLNARIAVEGQDAKKVAANYLKDNGFIKN

Strain: Y. pseudotuberculosis PB1/+
Gene: YPTS_1324

Classification: substrate-binding region of ABC-type glycine betaine transport system
Boundaries of gene: 1471848..1472780

Oligo evidence:

ggcagataacgtccgtgtaggctctaaaattgataccgaaggctccttgctgggcaatattattgtgcag
atcatccgtgaagttactctgaaagctcacccgaacatcccggctttgcttaatccggtattcgccacac

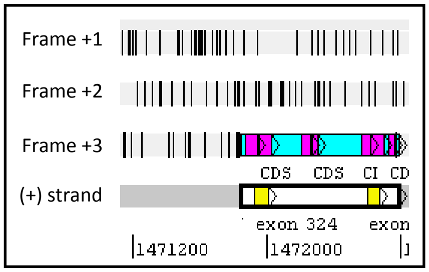

Peptide evidence:

AHPNIPALLNPVFATLDGPTLQK
DLATANNLR
GVQPIYAPAPIIR
IDTEGSLLGNIIVQVLEANGIK
IFAVAAIMLSASSGISHAAD
KDLATANNLR
KVAANYLK
LVWLSPAPANNTWTIAVR

Current sequence (peptide evidence in red): 310aa
MALFLTRSRIFAVAAIMLSASSGISHAADNVRVGSKIDTEGSLLGNIIVQVLEANGIKTTNKSQLGATKVVRGAITAGEIDIYPEYTGNGAFFFSDEQDPAWKSAKAGYEKVKALDYEKNKLVWL
SPAPANNTWTIAVRKDLATANNLRTLDDLGKWINGGGQFKLAASAEFIERPDALPAFQQAYGFTLNQDQLLSLAGGDTAVTIKAAAEQISGVNAAMAYGTDGPVAALGLQTLEDTKGVQPI
YAPAPIIREVTLKAHPNIPALLNPVFATLDGPTLQKLNARIAVEGQDAKKVAANYLKDNGFIKN

Error 13

Strain: Y. pestis CO92
Gene: YPO1219

Classification: expressed pseudogene
Boundaries of gene: Frame-2: complement (1377956..1379881)
Frame-3: complement (1377192..1377956)

Oligo evidence:

agcattggccgacagatacaatcatggttcggggctgacattttttatgtaaccaattatgtaaaaaat
aaggttgagtacattacagaagatgatataaacctctatgagaggcaactaaaaaccagtgactattatt

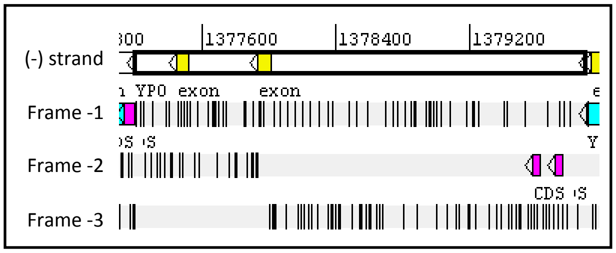

Peptide evidence:

HALTNIANGLQQR
LRPDVYYIEKPR

Proposed sequence (peptide evidence in red):
Frame-2: 642aa
MQNNSSTHSAANITRYFWLFIVLLLMTVGLYAYNYTNAYLTEKKHALTNIANGLQQRIDDYRYHTYQIYDLVNNPIKASEPPPVVQETRLRPDVYYIEKPRRKTDAIIFGNHEPATLTMALQISDYLDNLWGPQNDTYSMYYLNGQDNSLLLITTQALKEVTSRFKESYLTAFAESRRAEMLQQVNTLDERESFSPLRKLRFQNAYYFTLRTTFNRPGHLATVIAFDLPINDLIPPNMARSNFLLQPDKVPLNEGATPEDIAATSVSLNGSWVEFSAPLANVTLKIIYRVPVSHLIIDLLINNFWLILANIVLLALTILAAYFVRHQYGRPSADVTGQLEAQRLLSQEIITNLPQGLLIYDFSNSTVIASNKIAENLLPHLNLQKIATMAEQHHGVIQGTVNNEVYEIHMFRSQISPETYLFLLNDQDKEVLVNKKLQQARREYDKNLQARKLMLHNLGIELGQPVHHLSQLVRTLQDTTDLQQQHDIKTKLVEQSATILELIDNITLLTKLETQDWQTEQQVFSLSTLIDNLLLDLLPSINRKGLNLFNHFHVSLDQIYLGDEKVLRKILSLLLNYSIVTTAYGKITLNVDHEPGHPEQLVIQIMDTGAGISDEEIGNLNYPFLSQALADRYNHGSGLTFF

Frame-3: 255aa
LCNQLCKKLNGQLEIRSKVDIGTRYTIRLTIAVKNEQQDTQEKLLDGVTVLLDITSEEVRSIITILLNSFGANCILTDERLPGRDYDVTITDNPQHYDNYTLLLAADETGFHQLQNNYIRANYNLSSAVIDSILLLIERRILSEQSQQKVEYITEDDINLYERQLKTSDYYSLFVETVPVDLKKLYTELQQSDLTSLSQTVHRLKGVFAMLNLVLGKQLCETLEQHIADGDRLKIENSISQIDFFITRLLQEGNP

Strain: Y. pestis pestoides F
Gene: YPDSF_2473

Classification: hypothetical protein
Boundaries of gene: YPDSF_2473: 2812245..2814197
YPDSF_2474: 2814365..2814937

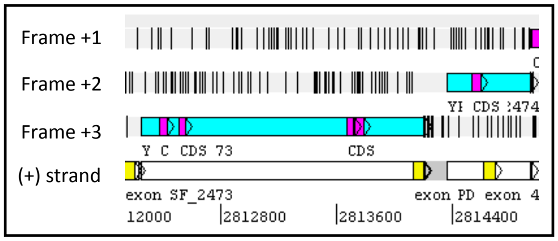

Oligo evidence:

agcattggccgacagatacaatcatggttcggggctgacattttttatgtaaccaattatgtaaaaaat
aaggttgagtacattacagaagatgatataaacctctatgagaggcaactaaaaaccagtgactattatt

Peptide evidence:

HALTNIANGLQQR
LVEQSATILELIDNITLLTK
LRPDVYYIEKPR
TLQDTTDLQQQHDIK

ANYNLSSAVIDSILLLIER

Current sequence (peptide evidence in red): YPDSF_2473: 650aa
MQNNSSTHSAANITRYFWLFIVLLLMTVGLYAYNYTNAYLTEKKHALTNIANGLQQRIDDYRYHTYQIYDLVNNPIKASEPPPVVQETRLRPDVYYIEKPRRKTDAIIFGNHEPATLTMALQISDYLDNLWGPQNDTYSMYYLNGQDNSLLLITTQALKEVTSRFKESYLTAFAESRRAEMLQQVNTLDERESFSPLRKLRFQNAYYFTLRTTFNRPGHLATVIAFDLPINDLIPPNMARSNFLLQPDKVPLNEGATPEDIAATSVSLNGSWVEFSAPLANVTLKIIYRVPVSHLIIDLLINNFWLILANIVLLALTILAAYFVRHQYGRPSADVTGQLEAQRLLSQEIITNLPQGLLIYDFSNSTVIASNKIAENLLPHLNLQKIATMAEQHHGVIQGTVNNEVYEIHMFRSQISPETYLFLLNDQDKEVLVNKKLQQARREYDKNLQARKLMLHNLGIELGQPVHHLSQLVRTLQDTTDLQQQHDIKTKLVEQSATILELIDNITLLTKLETQDWQTEQQVFSLSTLIDNLLLDLLPSINRKGLNLFNHFHVSLDQIYLGDEKVLRKILSLLLNYSIVTTAYGKITLNVDHEPGHPEQLVIQIMDTGAGISDEEIGNLNYPFLSQALADRYNHGSGLTFFYVTNYVKN

YPDSF_2474: 190 aa
LLNSFGANCILTDERLPGRDYDVTITDNPQHYDNYTLLLAADETGFHQLQNNYIRANYNLSSAVIDSILLLIERRILSEQSQQKVEYITEDDINLYERQLKTSDYYSLFVETVPVDLKKLYTELQQSDLTSLSQTVHRLKGVFAMLNLVLGKQLCETLEQHIADGDRLKIENSISQIDFFITRLLQEGNP

Strain: Y. pseudotuberculosis PB1/+
Gene: YPTS_1347

Classification: phosphotransfer intermediate protein in two-component regulatory system with RcsBC
Boundaries of gene: complement (1503527..1506220)

Oligo evidence:

aaggttgagtacattacagaagatgatataaacctctatgagaggcaactaaaaaccagtgactattatt

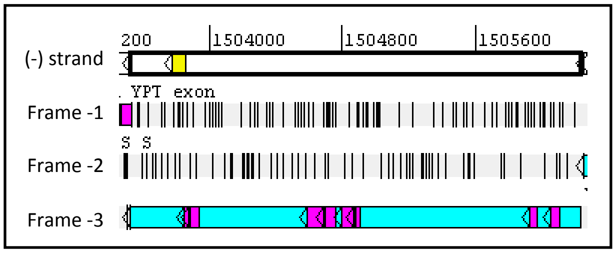

Peptide evidence:

ANYNLSSAVIDSILLLIER
HALTNIANGLQQR
ILSEQSQQK
LETQDWQTEQQVFSLSTLIDNLLLDLLPSINRK
LMLHNLGIELGQPVHHLSQLVR
LRPDVYYIEKPR
LVEQSATILELIDNITLLTK
REYDKNLQAR

Current sequence (peptide evidence in red): 897aa
MQNNSSTHSAANITRYFWLFIVLLLMTVGLYAYNYTNAYLTEKKHALTNIANGLQQRIDDYRYHTYQIYDLVNNPIKASEPPPVVQETRLRPDVYYIEKPRRKTDAIIFGNHEPATLTMALQISDYLDNLWGPQNDTYSMYYLNGQDNSLLLITTQALKEVTSRFKESYLTAFAESRRAEMLQQVNTLDERESFSPLRKLRFQNAYYFTLRTTFNRPGHLATVIAFDLPINDLIPPNMARSNFLLQPDKVPLNEGATPEDIAATSVSLNGSWVEFSAPLANVTLKIIYRVPVSHLIIDLLINNFWLILANIVLLALTILAAYFVRHQYGRPSADVTGQLEAQRLLSQEIITNLPQGLLIYDFSNSTVIASNKIAENLLPHLNLQKIATMAEQHHGVIQGTVNNEVYEIHMFRSQISPETYLFLLNDQDKEVLVNKKLQQARREYDKNLQARKLMLHNLGIELGQPVHHLSQLVRTLQDTTDLQQQHDIKTKLVEQSATILELIDNITLLTKLETQDWQTEQQVFSLSTLIDNLLLDLLPSINRKGLNLFNHFHVSLDQIYLGDEKVLRKILSLLLNYSIVTTAYGKITLNVDHEPGHPEQLVIQIMDTGAGISDEEIGNLNYPFLSQALADRYNHGSGLTFFLCNQLCKKLNGQLEIRSKVDIGTRYTIRLTIAVKNEQQDTQEKLLDGVTVLLDITSEEVRSIITILLNSFGANCILTDERLPGRDYDVTITDNPQHYDNYTLLLAADETGFHQLQNNYIRANYNLSSAVIDSILLLIERRILSEQSQQKVEYITEDDINLYERQLKTSDYYSLFVETVPVDLKKLYTELQQSDLTSLSQTVHRLKGVFAMLNLVLGKQLCETLEQHIADGDRLKIENSISQIDFFITRLLQEGNP

Error 14

Strain: Y. pestis CO92
Gene: YPO1337

Classification: expressed pseudogene
Boundaries of gene: region 1: complement (1504641..1505183)
region 2: complement (1502469..1502774)

Oligo evidence:

tccacttatatctccaccaacggcccaatattgatccaccgttttactcaggattagcttctgctataac
taaacatcctgaaattcagaccgtgtcttatgactgtcaacgacggatgaaaagtgatccacttatatct
acctacccgccatttgaatctatggatgccaataacgaaatcgttggctttgatatggatctggcaaaag

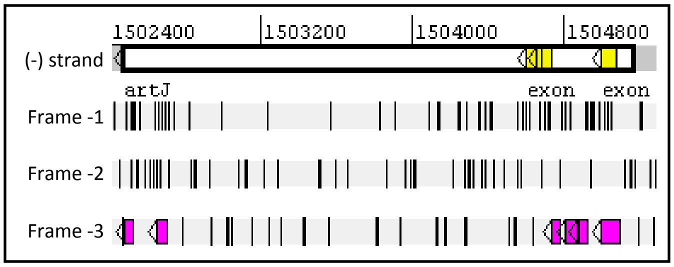

Peptide evidence:

FAASATYPPFESMDANNEIVGFDMDLAK
KIGMENGTTHQK
QVAFTQPYYANSAIVIAPK
SKQVAFTQPYYANSAIVIAPK
ADGTYQAINDK
DGVFGDTAVVNEWLK
IDGVFGDTAVVNEWLK

Proposed sequence (peptide evidence in red):
Region 1: 181aa
MKNSDRSLSMKKLLLATLLSGMAFSATAAETLRFAASATYPPFESMDANNEIVGFDMDLAKALCKQMEANCTFTNQAFDSLIPALKFKRYDAVISGMDITPERSKQVAFTQPYYANSAIVIAPKGKFSTFADLKGKKIGMENGTTHQKYLQDKHPEIQTVSYDCQRRMKSDPLISPPTAQY

Region 2: 102aa
MGVDHYCTVRYNDSYQNAIIDLKNGRIDGVFGDTAVVNEWLKTNPNLASVGEHVTDPQYFGTGLGIAVRPDNIALLTKLNKAIDAVKADGTYQAINDKWFPQ

Strain: Y. pestis pestoides F
Gene: YPDSF_2359

Classification: hypothetical protein
Boundaries of gene: 2688865..2689596

Oligo evidence:

acctacccgccatttgaatctatggatgccaataacgaaatcgttggctttgatatggatctggcaaaag
ataaacatcctgaaattcagaccgtgtcttatgacagctaccagaatgccatcattgatctgaaaaatgg

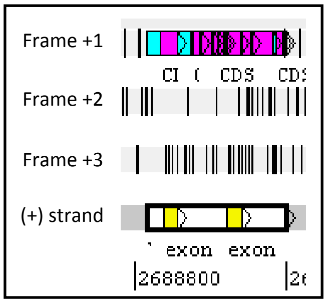

Peptide evidence:

ADGTYQAINDK
FAASATYPPFESMDANNEIVGFDMDLAK
GKFSTFADLK
GKKIGMEN
IDGVFGDTAVVNEWLK
KIGMENGTTHQK
QVAFTQPYYANSAIVIAPK
RYDAVISGMDITPER
SKQVAFTQPYYANSAIVIAPK
TNPNLASVGEHVTDPQYFGTGLGIAVRPDNIALLTK
YLQDKHPEIQTVSYDSYQNAIIDLK

Current sequence (peptide evidence in red): 243aa
MKKLLLATLLSGMAFSATAAETLRFAASATYPPFESMDANNEIVGFDMDLAKALCKQMEANCTFTNQAFDSLIPALKFKRYDAVISGMDITPERSKQVAFTQPYYANSAIVIAPKGKFSTFADLKGKKIGMENGTTHQKYLQDKHPEIQTVSYDSYQNAIIDLKNGRIDGVFGDTAVVNEWLKTNPNLASVGEHVTDPQYFGTGLGIAVRPDNIALLTKLNKAIDAVKADGTYQAINDKWFPQ

Strain: Y. pseudotuberculosis PB1/+
Gene: YPTS_1464

Classification: cationic amino acid ABC transporter, periplasmic binding protein
Boundaries of gene: complement (1626984..1627715)

Oligo evidence:

ataaacatcctgaaattcagaccgtgtcttatgacagctaccagaatgccatcattgatctgaaaaatgg
acctacccgccatttgaatctatggatgccaataacgaaatcgttggctttgatatggatctggcaaaag

Peptide evidence:

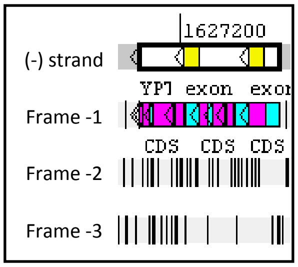

ADGTYQAINDK
ADGTYQAINDKWFPQ
DGVFGDTAVVNEWLK
FAASATYPPFESMDANNEIVGFDMDLAK
IDGVFGDTAVVNEWLK
KIGMENGTTHQK
QVAFTQPYYANSAIVIAPK
RYDAVISGMDITPER
SKQVAFTQPYYANSAIVIAPK
TNPNLASVGEHVTDPQYFGTGLGIAVRPDNIALLTK

Current sequence (peptide evidence in red): 243aa
MKKLLLATLLSGMVFSATAAETLRFAASATYPPFESMDANNEIVGFDMDLAKALCKQMEANCTFTNQAFDSLIPALKFKRYDAVISGMDITPERSKQVAFTQPYYANSAIVIAPKGKFSTFADLKGKKIGMENGTTHQKYLQDKHPEIQTVSYDSYQNAIIDLKNGRIDGVFGDTAVVNEWLKTNPNLASVGEHVTDPQYFGTGLGIAVRPDNIALLTKLNKAIDAVKADGTYQAINDKWFPQ

Error 15

Strain: Y. pestis CO92
Gene: YPO1584

Classification: expressed pseudogene
Boundaries of gene: complement (1808243..1808536)

Oligo evidence:

tacctgtcttctatggatcaaccacaagaagatgcgttggttgatagaacgctctggcttacgaccgatg
accgatgttgcacgccaattaattaatatattagaagctggtattgctaaaattgagtcatcagagagcc

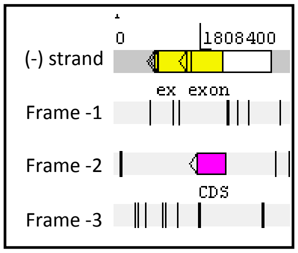

Peptide evidence:

LHYLSSMDQPQEDALVDR

Proposed sequence (peptide evidence in red): 98aa
MDRNNEVIQTHPVVGWDISTVDVYDAMMIRLHYLSSMDQPQEDALVDRTLWLTTDVARQLINILEAGIAKIESSESLSIILCNCHRIKGDRSGGHRTR

Strain: Y. pestis pestoides F
Gene: YPDSF_1861

Classification: annotated as BssS
Boundaries of gene: 2097596..2097892

Oligo evidence:

tacctgtcttctatggatcaaccacaagaagatgcgttggttgatagaacgctctggcttacgaccgatg
accgatgttgcacgccaattaattaatatattagaagctggtattgctaaaattgagtcatcagagagcc

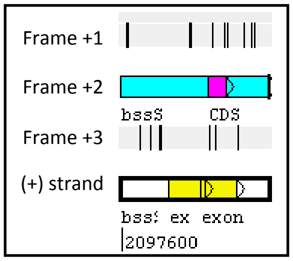

Peptide evidence:

QLINILEAGIAK

Current sequence (peptide evidence in red): 98aa
MDRNNEVIQTHPVVGWDISTVDVYDAMMIRLHYLSSMDQPQEDALVDRTLWLTTDVARQLINILEAGIAKIESSESLSIILCNCHRIKGDRSGGHRTR

Strain: Y. pseudotuberculosis PB1/+
Gene: YPTS_2572

Classification: annotated as BssS
Boundaries of gene: 2846646..2846900

Oligo evidence:

tgtagatgtgtacgatgctatgatgatacgtcttcattacctgtcttctatggagcaaccacaagaagat

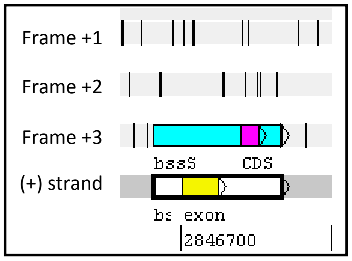

Peptide evidence:

QLINILEAGIAK

Current sequence (peptide evidence in red): 84aa
MDRNNEVIQTHPVVGWDISTVDVYDAMMIRLHYLSSMEQPQEDALVDRTLWLTTDVARQLINILEAGIAKIESSEYLDMDHRKH

Error 16

Strain: Y. pestis CO92
Gene: YPO1684

Classification: expressed pseudogene
Boundaries of gene: Frame+3:1919412..1921514
Frame+1: 1921498..1922043

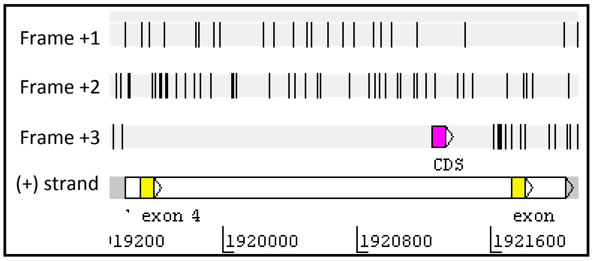

Oligo evidence:

ttttatggtgacgatatttgtgccaaatactgcattttctcaactctatgtgaacaatgccaatgatcct
atgtcgaatgtcaccaacgacgccaatgcttatacagaccagcgctattctgcacttaaagaagatctga

Peptide evidence:

YSTTDNTTSGTVSVGNAATGETR

Proposed sequence (peptide evidence in red):
Frame+3: 701aa
MKSIQKCDCNYLVRFAVSDSFIRDNSFRSLLAIFMVTIFVPNTAFSQLYVNNANDPGCYVIADGGFTGVTNSDQNCYTLSLNNINTNGGQLFVGGKGGIAGKPSFIATPWTGTFTTAVGTSNVATAYGFVVQSNGAFING
DTYVKGGLFLNGRKATNLAPATISSTSTDAVVGSQLYTVIQDGTRYFHANSVNPQDSVPAGQDAIAVGPATVVNGNNGIGIGSSAVVGPSAVGGIAIGPNTQATGIASTALGAGSQAHGSQSLALGAGATASQANSIAL
GASSVTTVGAESDYSAYGLTAPQTSVGEVGMGTAQGNRKITGVAAGSADYDVVNVAQLTAVGDKVEQNTADITSLGGRVTNVEGGMTRITNGGGIKYFHTHSTEPDSVASGSDSVAIGPNAQASGTTSIAMGAGSTA
QGAQSLALGAGAAASQANSIALGASSVTTVGAESDYSAYGLTAPQTSVGEVGVGTAQGNRKITGVAAGSADYDAVNVAQLTAVGDKVDQNTADITSLDGRVTNVEGEMASITNGGGVKYFHTHSTESDSVASGSDSV
AIGPNAQASGTASVASGKGTLASGNGAVAIGDAASVSAEGSVALGQGSADNGRGAESYTGKYSTTDNTTSGTVSVGNAATGETRTVSNVADGREAMDAVNLRQLDGAMAAVGDTVSGLQNGTDGMFQVNNNSG
QAKPSVTGTDAMAGGGRLRGVWQPQYRDGYGQQGDGGKQHRAGGQLSGGS

Frame+1: 182aa
MRWRGGAGSVASGSHSTAMGTGSKATAANSTALGANSVADRENSVSVGSVGNERQLTNIAVGTQGTDAVNLDQLNHSMSNVTNDANAYTDQRYSALKEDLKKQDSTLSAGIAGAMAMASLTQPYTPGASMATIG
AASYRGQSALSVGVSSISDSGRWVSKLQASSNTQGDMGVGVGVGYQW

Strain: Y. pestis pestoides F
Gene: YPDSF_1763 /YPDSF_1764

Classification: YPDSF_1763 annotated as surface protein
YPDSF_1764 annotated as hypothetical protein
Boundaries of gene: YPDSF_1764 Frame-2: complement (1988312..1990522)
YPDSF_1763 Frame-1: complement (1987891..1988436)

Oligo evidence:

ttttatggtgacgatatttgtgccaaatactgcattttctcaactctatgtgaacaatgccaatgatcct
atgtcgaatgtcaccaacgacgccaatgcttatacagaccagcgctattctgcacttaaagaagatctga

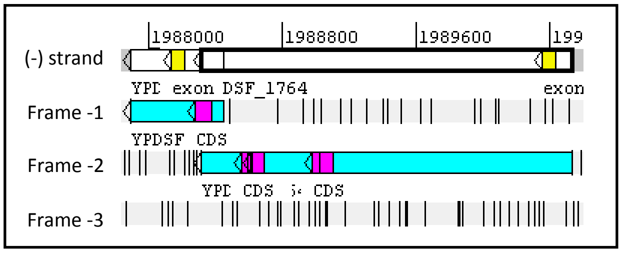

Peptide evidence:

VDQNTADITSLDGR
YSTTDNTTSGTVSVGNAATGETR
EAMDAVNLR
KITGVAAGSADYDAVNVAQLTAVGDKVDQNTADITSLDGR
TVSNVADGREAMDAVNLR
DQNTADITSLDGR
ATAANSTALGANSVADRENSVSVGSVGNER

Current sequence (peptide evidence in red):
Frame-2: 721aa (YPDSF_1764)
MKSIQKCDCNYLVRFAVSDSFIRDNSFRSLLAIFMVTIFVPNTAFSQLYVNNANDPGCYVIADGGFTGVTNSDQNCYTLSLNNINTNGGQLFVGGKGGIAGKPSFIATPWTGTFTTAVGTSNVATAYGFVVQSNGAFING
DTYVKGGLFLNGRKATNLAPATISSTSTDAVVGSQLYTVIQDGTRYFHANSVNPQDSVPAGQDAIAVGPATVVNGNNGIGIGSSAVVGPSAVGGIAIGPNTQATGIASTALGAGSQAHGSQSLALGAGATASQANSIAL
GASSVTTVGAESDYSAYGLTAPQTSVGEVGMGTAQGNRKITGVAAGSADYDVVNVAQLTAVGDKVEQNTADITSLGGRVTNVEGGMTRITNGGGIKYFHTHSTEPDSVASGSDSVAIGPNAQASGTTSIAMGAGSTA
QGAQSLALGAGAAASQANSIALGASSVTTVGAESDYSAYGLTAPQTSVGEVGVGTAQGNRKITGVAAGSADYDAVNVAQLTAVGDKVDQNTADITSLDGRVTNVEGEMASITNGGGVKYFHTHSTESDSVASGSDSV
AIGPNAQASGTASVASGKGTLASGNGAVAIGDAASVSAEGSVALGQGSADNGRGAESYTGKYSTTDNTTSGTVSVGNAATGETRTVSNVADGREAMDAVNLRQLDGAMAAVGDTVSGLQNGTDGMFQVNNNSG
QAKPSVTGTDAMAGGGQQGDGGKQHRAGGQLSGGS

Frame-1: 182aa (YPDSF_1763)
MRWRGGAGSVASGSHSTAMGTGSKATAANSTALGANSVADRENSVSVGSVGNERQLTNIAVGTQGTDAVNLDQLNHSMSNVTNDANAYTDQRYSALKEDLKKQDSTLSAGIAGAMAMASLTQPYTPGASMATIG
AASYRGQSALSVGVSSISDSGRWVSKLQASSNTQGDMGVGVGVGYQW

Strain: Y. pseudotuberculosis PB1/+
Gene: YPTS_2473

Classification: annotated as YadA domain-containing protein
Boundaries of gene: 2740553..2743195

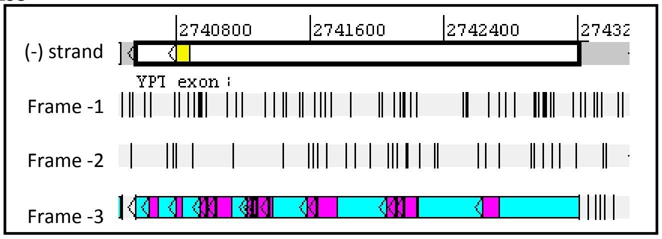

Oligo evidence:

atgtcgaatgtcaccaacgacgccaatgcttatacagaccagcgctattctgcacttaaagaagatctga

Peptide evidence:

ATAANSTALGANSVADR
ATAANSTALGANSVADRENSVSVGSVGNER
ATNLAPATVSSTSTDAVVGSQLYTVIQDGTR
EAMDAVNLR
ENSVSVGSVGNER
GAESYTGK
GQSALSVGVSSISDSGR
ITGVAAGSADYDAVNVAQLTAVGDKVDQNTADITSLDGR
ITGVAAGSAGYDAVNVTQLTAVGNK
KATNLAPATVSSTSTDAVVGSQLYTVIQDGTR
KITGVAAGSAGYDAVNVTQLTAVGNK
PSATGTDAMAGGAGSVASGSHSTAMGTGSK
TVSNVADGR
VTNVEGEMTSITNGGGVK
VTNVEGGMTSITNGGGIK
YSALKEDLKK
YSTTDNTTSGTVSVGNAATGETR

Current sequence (peptide evidence in red): 880aa
MKSIQKCDCNYLARFAVSDSFIRDNSFRGLLAIFMVTIFVPGTAFSELYVNNANDPGCYAVVDDNNLNFKGRITGIVTNHIYCNTLTEANLNTNGGQLFVGGQGGLPGYAPTPWTGTFTTAVGTSNVATAYGFVVQKNG
AFINGDTYVQGGLFLNGRKATNLAPATVSSTSTDAVVGSQLYTVIQDGTRYFHANSVNPQDSVPAGQDAIAVGPATVVNGNNGIGIGNSAVVGPSAVGGIAIGPNTQATGTASTALGAGTQAQGAQSLALGAGAVTR
QVNSIALGASSVTTVGAQGSYSAYGLPTTQASVGEVGIGTAQGNRKITGVAAGSAGYDAVNVTQLTAVGNKVDQNTADITSLDGRVTNVEGGMTSITNGGGIKYFHTHSTEPDSVASGSDSVAIGPNAQASGTTSIALG
AGSTAQGAQSLALGAGAAASQANSIALGASSVTTVGAESNYSAYGLTASQTSVGEVGVGTAQGNRKITGVAAGSADYDAVNVAQLTAVGDKVDQNTADITSLDGRVTNVEGEMTSITNGGGVKYFHTHSTEPDSVAS
GSDSVAIGPNAQASGTASVASGKGTLASGNGAVAIGDAASVSAEGSVALGQGSADNGRGAESYTGKYSTTDNTTSGTVSVGNAATGETRTVSNVADGREAMDAVNLRQLDGAMAAVGDTVSGLQNGTDGMFQVN
NNSGQAKPSATGTDAMAGGAGSVASGSHSTAMGTGSKATAANSTALGANSVADRENSVSVGSVGNERQLTNIAVGTQGTDAVNLDQLNHSMSNVTNDANAYTDQRYSALKEDLKKQDSTLSAGIAGAMAMASLT
QPYTPGASMATIGAASYRGQSALSVGVSSISDSGRWVSKLQASSNTQGDMGVGVGVGYQW

Error 17

Strain: Y. pestis CO92
Gene: YPO1726

Classification: expressed pseudogene
Boundaries of gene: 1971786..1972133

Oligo evidence:

gcacggtgcagtatcgttggaagacaacagcatcttgattgatacttttacgcctaaacgggatgatttt

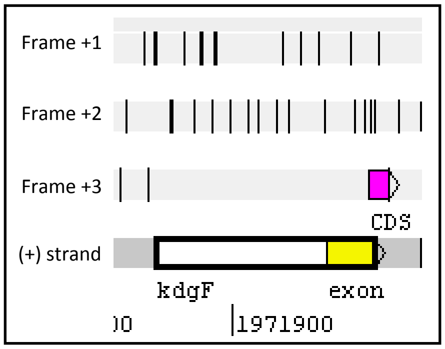

Peptide evidence:

DDFETVNNIV

Proposed sequence (peptide evidence in red): 116aa
MKMFFINDETPWEELGNGIKRKVMTWSDELMMVCVHFEKGAIGVAHKHDIHDQIAYVAAGSFEVEIEGQKRILKAGDAYRAVKNEMHGAVSLEDNSILIDTFTPKRDDFETVNNIV

Strain: Y. pestis pestoides F
Gene: YPDSF_1722

Classification: hypothetical protein
Boundaries of gene: complement (1937726..1938076)

Oligo evidence:

gcacggtgcagtatcgttggaagacaacagcatcttgattgatacttttacgcctaaacgggatgatttt

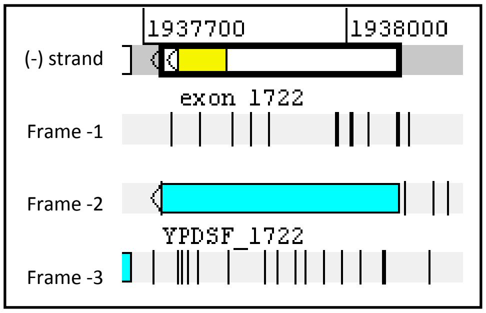

Peptide evidence:

NA

Current sequence (peptide evidence in red): 116aa
MKMFFINDETPWEELGNGIKRKVMTWSDELMMVCVHFEKGAIGVAHKHDIHDQIAYVAAGSFEVEIEGQKRILKAGDAYRAVKNEMHGAVSLEDNSILIDTFTPKRDDFETVNNIV

Strain: Y. pseudotuberculosis PB1/+
Gene: YPTS_2429

Classification: cupin 2 domain-containing protein
Boundaries of gene: complement (2692499..2692831)

Oligo evidence:

gcacggtgcagtatcgttggaagacaacagcatcttgattgatacttttacgcctaaacgggatgatttt

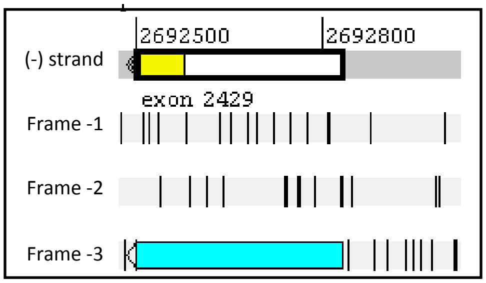

Peptide evidence:

NA

Current sequence (peptide evidence in red): 110aa
MKMFFINDETPWEALGNGIKRKVMTWSDELMMVCVHFEKGAIGVAHKHDIHDQIAYVAAGSFEVEIEGQKRILKAGDAYRAVKNEMHGAVSLEDNSILIDTFTPKRDDFL

Error 18

Strain: Y. pestis CO92
Gene: YPO1728

Classification: expressed pseudogene with 2 insertions
Boundaries of gene: Region1: 1973867..1974682
Region2: 1976642..1976842

Oligo evidence:

Gagtggatgcattgatcgtggctcatacccagcctgaagactttcggttgcagtatttgcagaaacaaaa
aagtggatcacttttcatccgtcgttgacacttatcgcttatgatgggctaccggatgatagtttgttag
accggatgatagtttgttagatattgccgtgaccccttattgttcaaaatacgcgtaccagtgtaggtaaa

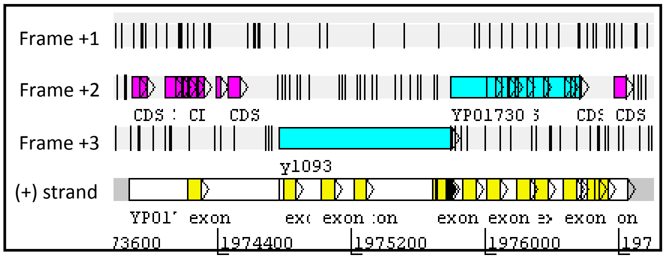

Peptide evidence:

ALNGYDDVAAETR
GLDLLLIPDEPGEK
IANELGLSSTTVSR
LLELGHQR
LQGYVQTMSEAGLMPLAGYLQK
QNFPFLALGR
RVDALIVAHTQPEDFR
VLNNSTFLEMISWIGIELGK
VLNNSTFLEMISWIGIELGKR
YQSLIHLVETR
ELQVLWQPEIGEGETDGVNR

Proposed sequence (peptide evidence in red):
Region1: 272aa
MSLKKIANELGLSSTTVSRALNGYDDVAAETRERIIDAAKRLGYQPNSLARRLKMGRTDAIALAYPSRPRVLNNSTFLEMISWIGIELGKRGLDLLLIPDEPGEKYQSLIHLVETRRVDALIVAHTQPEDFRLQYLQKQNFPFLALGRSHLPKPYAWFDFDNHAGASLAVKRLLELGHQRIAFVSTDARISYVDQRLQGYVQTMSEAGLMPLAGYLQKADPTRPGGYLAASRLLALEVPPTAIITDCNMLGDGVASALDKAGLLGGEGISLI

Region2: 66aa
AYDGLPDDSLLDIAVTPIVQNTRTSVGKQIASMICDLLGGKDPKELQVLWQPEIGEGETDGVNRRG

Strain: Y. pestis pestoides F
Gene: YPDSF_1392

Classification: hypothetical protein
Boundaries of gene: complement (1557701..1558717)

Oligo evidence:

accggatgatagtttgttagatattgccgtgaccccttattgttcaaaatacgcgtaccagtgtaggtaaa
gagtggatgcattgatcgtggctcatacccagcctgaagactttcggttgcagtatttgcagaaacaaaa

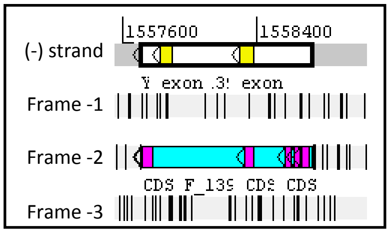

Peptide evidence:

ELQVLWQPEIGEGETDGVNR
IANELGLSSTTVSR
RLGYQPNSLAR
ALNGYDDVAAETR
RVDALIVAHTQPEDFR

Current sequence (peptide evidence in red): 338aa
MSLKKIANELGLSSTTVSRALNGYDDVAAETRERIIDAAKRLGYQPNSLARRLKMGRTDAIALAYPSRPRVLNNSTFLEMISWIGIELGKRGLDLLLIPDEPGEKYQSLIHLVETRRVDALIVAHTQPEDFRLQYLQKQNFPFLALGRSHLPKPYAWFDFDNHAGASLAVKRLLELGHQRIAFVSTDARISYVDQRLQGYVQTMSEAGLMPLAGYLQKADPTRPGGYLAASRLLALEVPPTAIITDCNMLGDGVASALDKAGLLGGEGISLIAYDGLPDDSLLDIAVTPIVQNTRTSVGKQIASMICDLLGGKDPKELQVLWQPEIGEGETDGVNRRG

Strain: Y. pseudotuberculosis PB1/+
Gene: YPTS_1729

Classification: LacI family transcription regulator
Boundaries of gene: 1959643..1960659

Oligo evidence:

gagtggatgcattgatcgtggctcatacccagcctgaagactttcggttgcagtatttgcagaaacaaaa
accggatgatagtttgttagatattgccgtgaccccttattgttcaaaatacgcgtaccagtgtaggtaaa

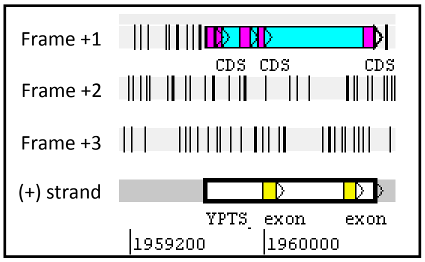

Peptide evidence:

ALNGYDDVAAETR
ELQVLWQPEIGEGETDGVNR
IANELGLSSTTVSR
VLNNSTFLEMISWIGIELGK
YQSLIHLVETR

Current sequence (peptide evidence in red): 338aa
MSLKKIANELGLSSTTVSRALNGYDDVAAETRERIIDAAKRLGYQPNSLARRLKMGRTDAIALAYPSRPRVLNNSTFLEMISWIGIELGKRGLDLLLIPDEPGEKYQSLIHLVETRRVDALIVAHTQPEDFRLQYLQKQNFPFLALGRSHLPKPYAWFDFDNHAGASLAVKRLLELGHQRIAFVSTDARISYVDQRLQGYVQTMSEAGLMPLAGYLQKADPTRPGGYLAASRLLALEVPPTAIITDCNMLGDGVASALDKAGLLGGEGISLIAYDGLPDDSLLDIAVTPIVQNTRTSVGKQIASMICDLLGGKDPKELQVLWQPEIGEGETDGVNRRG

Error 19

Strain: Y. pestis CO92
Gene: YPO1923
Oligo evidence:

aaccacaactggttgacttatctgtagaaataaagggtgatatacgggatattttcctgtggattattcc
taatgatggcatttgccatacgccctgacctgccagaattaaaaactattattgataaagtcattgaatc

Peptide evidence:

LIDYLNQILELLPTR
RTGIKFDPVYTK
LLQQESIINALPFAVFIR
SAQSLMLLLDDIIDSAK
TLSGGNPAVELQLLQSLLESNLQDAVTLTQLYR

Classification: expressed pseudogene
Boundaries of gene: Frame-3: complement (2180652..2182037)
Frame-1: complement (2178187..2180649)

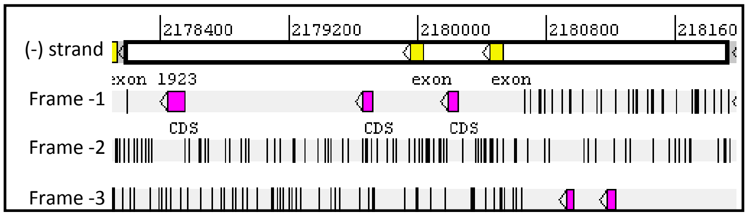

Proposed sequence (peptide evidence in red):
Frame-3: 462aa
MHTPIIWCNSLRTTFLLRGRFSLRTVLYCLLLISGQLHASLMTGRQGCDTGQPLTIHSIANSYNDDLLLTPATDTRQSSRRTVKVGVVMDANTPFVVNRDDNAIEGIVADYLKIISDASQLSFQMIGYCDYGLVLNALENGQ
IDLMAGTPMLAQPGLIASHAFFTNRHVEVRSKNWDPTKRTHPETVAIVNNEPLSPEFLFNYHADRIVAYPNQLQGLLAVAYGNADVFVANATSANYLIDQLQLLTLQIRNFAPYHPAPYSFLARESNQKLIDYLNQILELLP
TRATGDIQQRWFGSKHHYNIDAKLLLTEQELSWIQHHPVVTYIAPLDLAPLIFRDRRTGEMAGFSVDLIDIIARRTGIKFDPVYTKDTGEGVRSFKAGKVDLLPIVAVRNGQYGNNLYSSSIAQSLWGIMTREDRMDINNV
GDLAGKRVGIQAGSASSSIISNPLLAQRITFVEAPDT

Frame-1: 820aa
TLVRWLQQGKVDAVVKNMMTANYLSAQNFSPNIKTVAVAGEEPLMMAFAIRPDLPELKTIIDKVIESIPPEEFDNLTSEWSTFKPKPISFDESSLDNELLMIALKISSGLLLVFGLYLCYLVFNKRRQAKLLHARLLQQESIIN
ALPFAVFIRTASGELAVYNSHFADVHSDKLYDMLNQTHEPAHWPMTSPLDREIDKYCRSVLLNRKPQLVDLSVEIKGDIRDIFLWIIPLNNAEQGVLGGWLDISQRKTVERQLEAARVEAESANRTKSTFLATISHELRTPM
YAIMGLLELEIRSNRPVEKDTLVTVSKSAQSLMLLLDDIIDSAKIEAGQLSIHPTAVDFHQEMERMFTLYQPIANERGLRFSGWLDDHIPPLLMADMLRVRQVMGNLLGNALKFTEQGSVSVDITWEPSDEQHGVMNIDI
TDTGIGISPAAQATLFQPFSQANEGKSPRFGGSGLGLWICHQLIHKMGGKIALESQLGKGTSLLITLPLNIATADDLAQDAAITHVDDERLNQLRNLRILVVDDLPANRQLLQQQLAFIGIEQVVTAENGAKACQILQHNN
FDVVITDCSMPVMDGYELAAHIRQDPALKDLIVIGCTADAREESAARCIDAGMNACMIKPVAIDTLQATLLRKDIVSQISNTNHSQSTEDNDRGGNQSNLSEKNTAQQENAPAIHSTTITSSVNRLAAAQNKLRTLSGGN
PAVELQLLQSLLESNLQDAVTLTQLYRQLCVEDEDVVETVDTAETVDTADTVEDGETRGPGSTVGRHSTAGSDHIPHADIYKQMASLVHRIKGSVQLIDAQELVASCVKFESQ

Strain: Y. pestis pestoides F
Gene: YPDSF_1200
Oligo evidence:

taatgatggcatttgccatacgccctgacctgccagaattaaaaactattattgataaagtcattgaatc
aaccacaactggttgacttatctgtagaaataaagggtgatatacgggatattttcctgtggattattcc

Peptide evidence:

LIDYLNQILELLPTR
SAQSLMLLLDDIIDSAK
DDNAIEGIVADYLK
ILVVDDLPANR
TKSTFLATISHELR
TLSGGNPAVELQLLQSLLESNLQDAVTLTQLYR

Classification: signal transduction histidine kinase
Boundaries of gene: 1352820..1356821

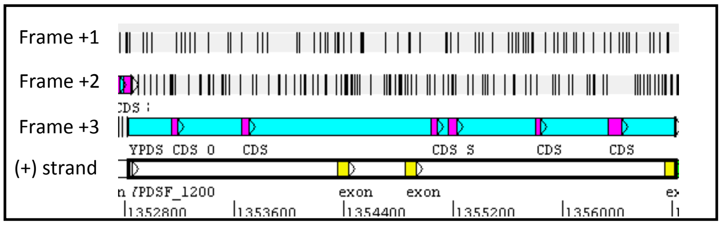

Current sequence (peptide evidence in red): 1333aa
MHTPIIWCNSLRTTFLLRGRFSLRSWFSLRTVLYCLLLISGQLHASLMTGRQGCDTGQPLTIHSIANSYNDDLLLTPATDTRQSSRRTVKVGVVMDANTPFVVNRDDNAIEGIVADYLKIISDASQLSFQMIGYCDYGLVLN
ALENGQIDLMAGTPMLAQPGLIASHAFFTNRHVEVRSKNWDPTKRTHPETVAIVNNEPLSPEFLFNYHADRIVAYPNQLQGLLAVAYGNADVFVANATSANYLIDQLQLLTLQIRNFAPYHPAPYSFLARESNQKLIDYL
NQILELLPTRATGDIQQRWFGSKHHYNIDAKLLLTEQELSWIQHHPVVTYIAPLDLAPLIFRDRRTGEMAGFSVDLIDIIARRTGIKFDPVYTKDTGEGVRSFKAGKVDLLPIVAVRNGQYGNNLYSSSIAQSLWGIMTREDR
MDINNVGDLAGKRVGIQAGSASSSIISNPLLAQRITFVEAPDTMTLVRWLQQGKVDAVVKNMMTANYLSAQNFSPNIKTVAVAGEEPLMMAFAIRPDLPELKTIIDKVIESIPPEEFDNLTSEWSTFKPKPISFDESSLDNE
LLMIALKISSGLLLVFGLYLCYLVFNKRRQAKLLHARLLQQESIINALPFAVFIRTASGELAVYNSHFADVHSDKLYDMLNQTHEPAHWPMTSPLDREIDKYCRSVLLNRKPQLVDLSVEIKGDIRDIFLWIIPLNNAEQGLLG
GWLDISQRKTVERQLEAARVEAESANRTKSTFLATISHELRTPMYAIMGLLELEIRSNRPVEKDTLVTVSKSAQSLMLLLDDIIDSAKIEAGQLSIHPTAVDFHQEMERMFTLYQPIANERGLRFSGWLDDHIPPLLMADML
RVRQVMGNLLGNALKFTEQGSVSVDITWEPSDEQHGVMNIDITDTGIGISPAAQATLFQPFSQANEGKSPRFGGSGLGLWICHQLIHKMGGKIALESQLGKGTSLLITLPLNIATADDLAQDAAITHVDDERLNQLRNLR
ILVVDDLPANRQLLQQQLAFIGIEQVVTAENGAKACQILQHNNFDVVITDCSMPVMDGYELAAHIRQDPALKDLIVIGCTADAREESAARCIDAGMNACMIKPVAIDTLQATLLRKDIVSQISNTNHSQSTEDNDRGGN
QSNLSEKNTAQQENAPAIHSTTITSSVNRLAAAQNKLRTLSGGNPAVELQLLQSLLESNLQDAVTLTQLYRQLCVEDEDVVETVDTAETVDTADTVEDGETRGPGSTVGRHSTAGSDHIPHADIYKQMASLVHRIKGSVQ
LIDAQELVASCVKFESQLHAQNKQAAMTHGADCLALFIESNQLLVTLISQYPKAATEDSPQ

Strain: Y. pseudotuberculosis PB1/+
Gene: YPTS_1971

Classification: histidine kinase
Boundaries of gene: complement (2172650..2176678)

Oligo evidence:

atctaatcagttattagtcacattaatttctcaatatcctaaagccgctactgaagactcgccacaataa
taatgatggcatttgccatacgccctgacctgccagaattaaaaactattattgataaagtcattgaatc

Peptide evidence:

SAQSLMLLLDDIIDSAK
SKNWDPTKR
TLSGGNPAVELQLLQSLLESNLQDAVTLTQLYR

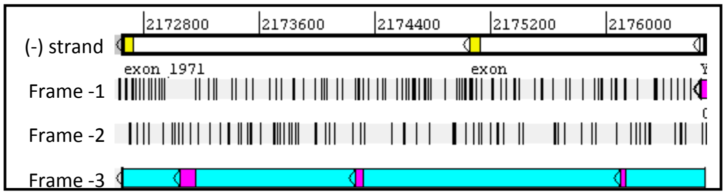

Current sequence (peptide evidence in red): 1342aa
MHTPIIWCNSLRTTFLLRARFLLRARFSLRSWFFLRTVLCCLLLISGQLHASLMTGRQGCDTGQPLTIHSIANSYNDDLLLTPATDTRQSSRRTVKVGVVMDANTPFVVNRDDNAIEGIVADYL
KIISDASQLSFQMIGYCDYGLVLNALENGQIDLMAGTPMLAQPRLIASHAFFTNRHVEVRSKNWDPTKRTHPETVAIVNNEPLSPEFLFNYHADRIVAYPNQLQGLLAVAYGNADVFVANAT
SANYLIDQLQLLTLQIRNFAPYHPAPYSFLARESNQKLIDYLNQILELLPTRATGDIQQRWFGSKHHYNIDAKLLLTEQEISWIQHHPVVTYIAPLDLAPLIFRDRRTGEMAGFSVDLIDIIARRTGI
KFDPVYTKDTGEGVRSFKAGKVDLLPIVAVRNGQYGNNLYSSSIAQSLWGIMTREDRMDINNVGDLAGKRVGIQAGSASSSIIGNPLLAQRITFVEAPDTMTLVRWLQQGKVDAVVKNMM
TANYLSAQNFSPNIKTVAVAGEEPLMMAFAIRPDLPELKTIIDKVIESIPPEEFDNLTSEWSTFKPKPISFDESSLDNELLMIALKISSGLLLVFGLYLCYLVFNKRRQAKLLHARLLQQESIINALPFA
VFIRTASGELAVYNSHFADVHSDKLYDMLNQTHEPAHWPMTSPLDREIDKYCRSVLLNRKPQLVDLSVEIKGEIRDIFLWIIPLNNAEQGLLGGWLDISQRKTVERQLEAARVEAESANRTKST
FLATISHELRTPMYAIMGLLELEIRSNRPVEKDTLVTVSKSAQSLMLLLDDIIDSAKIEAGQLSIHPTAVDFHQEMERMFTLYQPIANERGLRFSGWLDDHIPPLLMADMLRVRQVMGNLLGN
ALKFTEQGSVSVDITWEPSDEQHGVMNIDITDTGIGISPAAQATLFQPFSQANEGKSPRFGGSGLGLWICHQLIHKMGGKIALESQLGKGTSLLITLPLNIATADDLAQDAAITHVDDERLNQL
RNLRILVVDDLPANRQLLQQQLAFIGIEQVVTAENGAKACQILQHNNFDVVITDCSMPVMDGYELAAHIRQDPALKDLIVIGCTADAREESAARCIDAGMNACMIKPVAIDTLQATLLRKDIV
SQIANTNHSHSTEDNDRGGNQHNLSEKDTAQQENAPAIHSTTITSSVNRLAAAQNKLRTLSGGNPAVELQLLQSLLESNLQDAVTLTQLYRQLCVEDEDVVETVDTAETVDTAETADTVEDG
ETRGPGSTVGRHSTTGSDHIPHADIYKQMASLVHRIKGSVQLIDAQELVASCVKFESQLHAQNKQAAMTHGADCLALFIESNQLLVTLISQYPKAATEDSPQ

Error 20

Strain: Y. pestis CO92
Gene: YPO1967

Classification: expressed pseudogene
Boundaries of gene: 2235390..2236337

Oligo evidence:

tgatggtttcggtttgtcttttaattatcaaaacattcagggcagcggcgtcagttttgcaggcgcatac
ctcaataccagtttgagaatggtatccgcccgtctcttgtaacgaacggtgcaatagtgatccacaccca

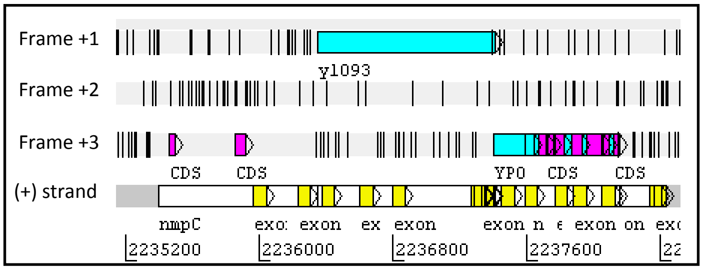

Peptide evidence:

NDNFFGLVEGLKVAAQYQGR

Proposed sequence (peptide evidence in red): 315aa
MKNIAMALAATLGIIPALSSAAEIYNKDGNKLDFYGRVAAKYLFKKQSNEDATYVRIGFKGETKINSQLTGFGQWEYNIAANNTESQGDKGNKTRLGFAGLKLADYGSFDYGRNYGVVYDVLS
YTDMLPEFGGDSMAATDNYMTARSTGLATYRNDNFFGLVEGLKVAAQYQGRNEDGDVTRNERSIQKANGDGFGLSFNYQNIQGSGVSFAGAYSSSNRTEGQKELVNSAAGDKAQAWAT
GLKYDANQAYIAAMYGETLNMTPYKRLIANKTQNVELVAQYQFENGIRPSLVTNGAIVIHTQRLKSDPGGNLLS

Strain: Y. pestis pestoides F
Gene: YPDSF_1156

Classification: hypothetical protein
Boundaries of gene: complement (1298434..1299516)

Oligo evidence:

taacgataagaacccattgggcttgaatacggatgatgttgttgcggtaaacttgacttaccgtttttaa
tgttgattataagattaatttattggataacgataagaacccattgggcttgaatacggatgatgttgtt
aataccagtttgagaatggtatccgcccgtctcttgcctacatacaatctaaaggtaaggatctggcggg
tgatggtttcggtttgtcttttaattatcaaaacattcagggcagcggcgtcagttttgcaggcgcatac

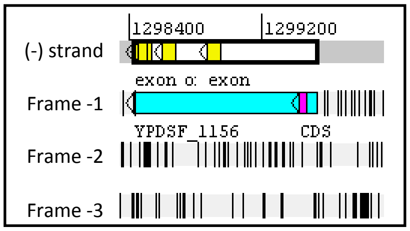

Peptide evidence:

NA

Current sequence (peptide evidence in red): 360aa
MKNIAMALAATLGIIPALSSAAEIYNKDGNKLDFYGRVAAKYLFKKQSNEDATYVRIGFKGETKINSQLTGFGQWEYNIAANNTESQGDKGNKTRLGFAGLKLADYGSFDYGRNYGVVYDVLS
YTDMLPEFGGDSMAATDNYMTARSTGLATYRNDNFFGLVEGLKVAAQYQGRNEDGDVTRNERSIQKANGDGFGLSFNYQNIQGSGVSFAGAYSSSNRTEGQKELVNSAAGDKAQAWAT
GLKYDANQAYIAAMYGETLNMTPYKRLIANKTQNVELVAQYQFENGIRPSLAYIQSKGKDLAGVGDTDLVKYAEIGVTYHFNKNMYTYVDYKINLLDNDKNPLGLNTDDVVAVNLTYRF

Strain: Y. pseudotuberculosis PB1/+
Gene: YPTS_2016

Classification: porin
Boundaries of gene: 2230091..2230789

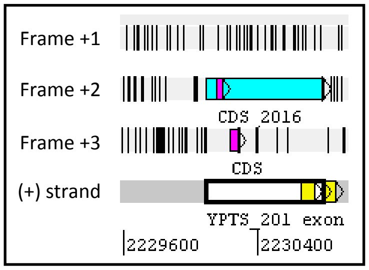

Oligo evidence:

tgatggtttcggtttgtcttttaattatcaaaacattcagggcagcggcgtcagttttgcaggcgcatac

Peptide evidence:

NA

Current sequence (peptide evidence in red): 232aa
MKNIAMALAATLGIIPALSSAAEIYNKDGNKLDFYGRVAAKYLFKKQSNEDATYVRIGFKGETKINSQLTGFGQWEYNIAANNTESQGDKGNKTRLGFAGLKLADYGSFDYGRNYGVVYDVLS
YTDMLPEFGGDSMAATDNYMTARSTGLATYRNDNFFGLVEGLKVAAQYQGRNEDGDVTRNERSIQKANGDGFGLSFNYQNIQGSGVSFAGAYSSSNRTEGQKVSTTDEK

Error 21

Strain: Y. pestis CO92
Gene: YPO2171

Classification: expressed pseudogene (purU)
Boundaries of gene: complement (2442302..2443171)

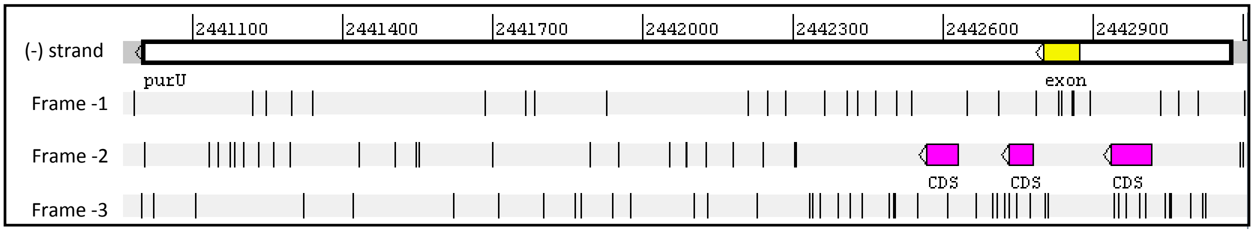

Oligo evidence:

gacttattaatgaagagtgcttatggtggcttagatgtagaaattgcggcagtcattggtaaccatgatg

Peptide evidence:

FDIPFHLVSHEGLTR
FPYQIINIHHSFLPAFIGAR
TELEGIFNDTTLLADLDDALPEGTNR

Proposed sequence (peptide evidence in red): 289aa
MPHQNVQKKVLRTICPDAKGLIAKITNICYKHQLNIVQNNEFVDHLTGRFFMRTELEGIFNDTTLLADLDDALPEGTNRELHVAGRRRIIMVTKEAHCLGDLLMKSAYGGLDVEIAAVIGNHD
ALQNLVERFDIPFHLVSHEGLTREQHDQQLIEKIEQYQPDYVVLAKYMRVLTPAFVQRFPYQIINIHHSFLPAFIGARPYHQAYERGVKIIGATAHYVNDSLDEGPIIMQDVINVDHSYTAEDM
MRAGRDVEKNVLSSALYRSLSIILCNCHRIKGDRSGGHRTR

Strain: Y. pestis pestoides F
Gene: YPDSF_0963

Classification: annotated as purU
Boundaries of gene: 1093071..1093919

Oligo evidence:

gacttattaatgaagagtgcttatggtggcttagatgtagaaattgcggcagtcattggtaaccatgatg

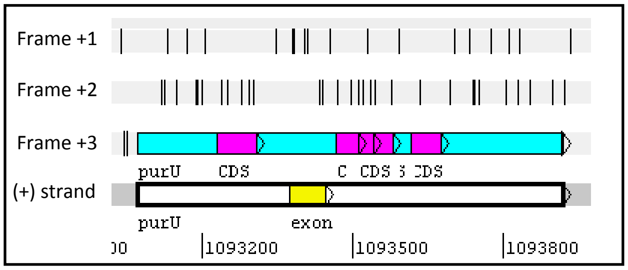

Peptide evidence:

IEQYQPDYVVLAK
EQHDQQLIEK
FDIPFHLVSHEGLTR
FPYQIINIHHSFLPAFIGAR
TELEGIFNDTTLLADLDDALPEGTNR

Current sequence (peptide evidence in red): 282aa
MPHQNVQKKVLRTICPDAKGLIAKITNICYKHQLNIVQNNEFVDHLTGRFFMRTELEGIFNDTTLLADLDDALPEGTNRELHVAGRRRIIMVTKEAHCLGDLLMKSAYGGLDVEIAAVIGNHD
ALQNLVERFDIPFHLVSHEGLTREQHDQQLIEKIEQYQPDYVVLAKYMRVLTPAFVQRFPYQIINIHHSFLPAFIGARPYHQAYERGVKIIGATAHYVNDSLDEGPIIMQDVINVDHSYTAEDM
MRAGRDVEKNVLSSALYRVLAQRVFVYGNRTVIL

Strain: Y. pseudotuberculosis PB1/+
Gene: YPTS_2163

Classification: annotated as purU
Boundaries of gene: complement (2386069..2386917)

Oligo evidence:

gacttattaatgaagagtgcttatggtggcttagatgtagaaattgcggcagtcattggtaaccatgatg

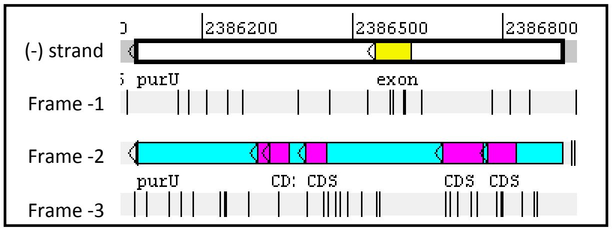

Peptide evidence:

FPYQIINIHHSF
FPYQIINIHHSFLPAFIGAR
HQLNIVQNNEFVDHLTGR
IEQYQPDYVVLAK
TELEGIFNDTTLLADLDDALPEGTNR

Current sequence (peptide evidence in red): 282aa
MPHQNVQKKVLRTICPDAKGLIAKITNICYKHQLNIVQNNEFVDHLTGRFFMRTELEGIFNDTTLLADLDDALPEGTNRELHVAGRRRIIMVTKEAHCLGDLLMKSAYGGLDVEIAAVIGNHD
ALQNLVERFDIPFHLVSHEGLTREQHDQQLIEKIEQYQPDYVVLAKYMRVLTPAFVQRFPYQIINIHHSFLPAFIGARPYHQAYERGVKIIGATAHYVNDSLDEGPIIMQDVINVDHSYTAEDM
MRAGRDVEKNVLSSALYRVLAQRVFVYGNRTVIL

Error 22

Strain: Y. pestis CO92
Gene: YPO2249

Classification: expressed pseudogene
Boundaries of gene: Frame+1: 2524378..2526162
Frame+2: 2526215..2526436

Oligo evidence:

aacacgagtggatatattattaggcgggaagttgtttcaacatttaatcaggttgccgttaagttacttt
ataacacaaggccgagtcaccgaacttgccagccatgacgaattactgcaattaaacggcagttacgctc

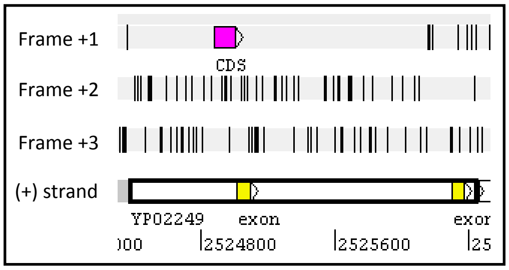

Peptide evidence:

QVIMDKVLVHHALTTLEVLIFILVIVGVYEVILKGLR

Proposed sequence (peptide evidence in red):
Frame+1: 594aa
MENHVHSALICASRLIRLAGLNPMDIETFSQKVAIDTSVKTTLASLQRYIVQFSQTASVRFKIRKQALEKIRQEQLPLAFRDRQGNFILLARMNEQQVLLQYADDKKPQMLSYQSLADMWGG
MILCCSHSRFDIRWFIPPLRRHRKPLMQVLLLSLLLQFLALISPLFFQVIMDKVLVHHALTTLEVLIFILVIVGVYEVILKGLREYIFTHTTTRVDILLGGKLFQHLIRLPLSYFKQRHVGNIVARVRELD
NIRDFITGSALTLCVDVVFTLVLFIVMWCISPLLTLIILSTLPFYLLLAVMTTRPLQKKVEALCGFAAQNGAFLTETVSGVETVKSLALEPRMRQRWESQTRDFAQANFQVQNLQNLSSQAAQLL
QKVAGALVIVIGAYHVMSVQLSIGQLIAFNMLALQALMPMSKLVDLWQQSIRAQVGLKLISDILSLPVEPEADPAAPHPAISGNILLKNVAFRYRPDLDPVLHDLNLSIRAGEHIGLVGPSGSG
KSTVARLLQRLYNTEQGVITIDGYPINHLSPEYLRRQVGVVMQESYLFNRTVRENIAHSRPTATLTEVVNAASLAGANAFILALRESPDLNF

Frame+2: 108aa
VLTPLFWRYANPRILIFDEATSALDDESQSEIQKNMARIIANRTVITIAHRLSTVRHCHRIAVITQGRVTELASHDELLQLNGSYARLWQQQVHFNQNKSTLTKTSPL

Strain: Y. pestis pestoides F
Gene: YPDSF_0885 /YPDSF_0886

Classification: YPDSF_0885 annotated as RTX toxin ABC transporter ATP binding/permease subunits.
YPDSF_0886 annotated as hypothetical protein
Boundaries of gene: YPDSF_0886 Frame-2: complement (1010123..1011907)
YPDSF_0885 Frame-1: complement(1009849..1010175)

Oligo evidence:

aacacgagtggatatattattaggcgggaagttgtttcaacatttaatcaggttgccgttaagttacttt
ataacacaaggccgagtcaccgaacttgccagccatgacgaattactgcaattaaacggcagttacgctc

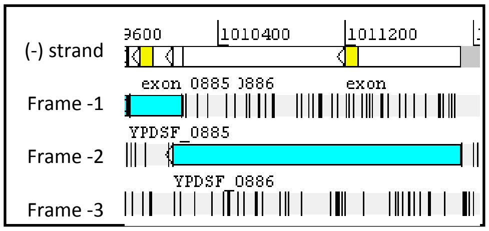

Peptide evidence:

NA

Current sequence (peptide evidence in red):
Frame-2: 594aa (YPDSF_0886)
MENHVHSALICASRLIRLAGLNPMDIETFSQKVAIDTSVKTTLASLQRYIVQFSQTASVRFKIRKQALEKIRQEQLPLAFRDRQGNFILLARMNEQQVLLQYADDKKPQMLSYQSLADMWGG
MILCCSHSRFDIRWFIPPLRRHRKPLMQVLLLSLLLQFLALISPLFFQVIMDKVLVHHALTTLEVLIFILVIVGVYEVILKGLREYIFTHTTTRVDILLGGKLFQHLIRLPLSYFKQRHVGNIVARVRELD
NIRDFITGSALTLCVDVVFTLVLFIVMWCISPLLTLIILSTLPFYLLLAVMTTRPLQKKVEALCGFAAQNGAFLTETVSGVETVKSLALEPRMRQRWESQTRDFAQANFQVQNLQNLSSQAAQLL
QKVAGALVIVIGAYHVMSVQLSIGQLIAFNMLALQALMPMSKLVDLWQQSIRAQVGLKLISDILSLPVEPEADPAAPHPAISGNILLKNVAFRYRPDLDPVLHDLNLSIRAGEHIGLVGPSGSG
KSTVARLLQRLYNTEQGVITIDGYPINHLSPEYLRRQVGVVMQESYLFNRTVRENIAHSRPTATLTEVVNAASLAGANAFILALRESPDLNF

Frame-1: 108aa (YPDSF_0885)
VLTPLFWRYANPRILIFDEATSALDDESQSEIQKNMARIIANRTVITIAHRLSTVRHCHRIAVITQGRVTELASHDELLQLNGSYARLWQQQVHFNQNKSTLTKTSPL

Strain: Y. pseudotuberculosis PB1/+
Gene: YPTS_2239

Classification: type I secretion system ATPase
Boundaries of gene: 2465281..2467428

Oligo evidence:

aacacgagtggatatattattaggcgggaagttgtttcaacatttaatcaggttgccgttaagttacttt
ataacacaaggccgagtcaccgaacttgccagccatgacgaattactgcaattaaacggcagttacgctc

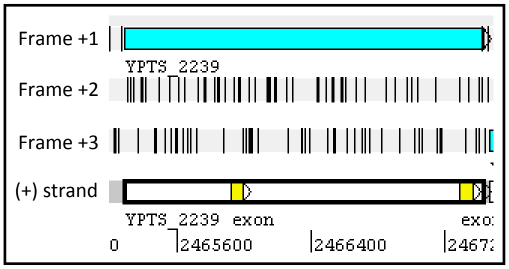

Peptide evidence:

NA

Current sequence (peptide evidence in red): 715aa
MENHVHSALICASRLIRLAGLNPMDIETFSQKVAIDTSVKTTLASLQRYIVQFSQTASVRFKIRKQALEKIRQEQLPLAFRDRQGNFILLARMNEQQVLLQYADDKKPQMLSYQSLADMWGG
MILCCSHSRFDIRWFIPPLRRHRKPLMQVLLLSLLLQFLALISPLFFQVIMDKVLVHHALTTLEVLIFILVIVGVYEVILKGLREYIFTHTTTRVDILLGGKLFQHLIRLPLSYFKQRHVGNIVARVRELD
NIRDFITGSALTLCVDVVFTFVLFIVMWCISPLLTLIILSTLPFYLLLAVMTTRPLQKKVEALCGFAAQNGAFLTETVSGVETVKSLALEPRMRQRWESQTRDFAQANFQVQNLQNLSSQAAQLL
QKVAGALVIVIGAYHVMFVQLSIGQLIAFNMLALQALMPMSKLVDLWQQSIRAQVGLKLISDILSLPVEPEADPAAPHPAISGNILLKNVAFRYRPDLDPVLHDLNLSIRAGEHIGLVGPSGSG
KSTVARLLQRLYNTEQGVITIDGYPINHLSPEYLRRQVGVVMQESYLFNRTVRENIAHSRPTATLTEVVNAASLAGANAFILALPLGYDTVLSEGGSSLSGGQRQRIAIARTLLANPRILIFDEATS
ALDDESQSEIQKNMARIIANRTVITIAHRLSTVRHCHRIAVITQGRVTELASHDELLQLNGSYARLWQQQVHFNQNKSTLTKTSPP

Error 23

Strain: Y. pestis CO92
Gene: YPO2398

Classification: expressed pseudogene
Boundaries of gene: 2694609..2695628

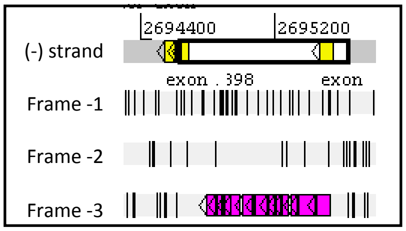

Oligo evidence:

actgccgcccgctaatagtcgcttaattggcgaaaatacaacctttactgttccaaatgatggtcgccct
gcctgtagcgtctcaggatgagcaacaggcacaggctgaagcactactgtcaacgacggatgaaaagtga

Peptide evidence:

DTPTMVTSISQK
EGIVINLAELR
GEYLIHGTNADFGIGLR
IGLLGMLEANPGTDPYLPLPGSVLTIPNQMLLPDTPR
IPNPTWTPTANIR
KNYLAEGITLPSVVPAGPDNPMGQFALR
LIGENTTFTVPNDGRPLEAIAADYK
LRPDDIEALFNSVPK
LSAGRGEYLIHGTNADFGIGLR
NYLAEGITLPSVVPAGPDNPMGQFALR
RYVEVHQPLSR
VDSDDPQTMPIAIGSGLQK
VIVYPIGIGQLGR
VQIINEPVK
YVEVHQPLSR

Proposed sequence (peptide evidence in red): 339aa

MKRALTLIGMLFATCLAGSLTAANANEYPLPPANSRLIGENTTFTVPNDGRPLEAIAADYKIGLLGMLEANPGTDPYLPLPGSVLTIPNQMLLPDTPREGIVINLAELRLYYYPKGQNKVIVYPIGIGQLGRDTPTMVTSISQKIPNPTWTPTANIRKNYLAEGITLPSVVPAGPDNPMGQFALRLSAGRGEYLIHGTNADFGIGLRVSSGCIRLRPDDIEALFNSVPKGTRVQIINEPVKYAVEPDGKRYVEVHQPLSRVDSDDPQTMPIAIGSGLQKFINDSQTDAKAVQDAIVRRSGMPTIVTVGEASTQQAPTPVASQDEQQAQAEALLSTTDEK

Strain: Y. pestis pestoides F
Gene: YPDSF_0750

Classification: hypothetical protein
Boundaries of gene: 847913..848932

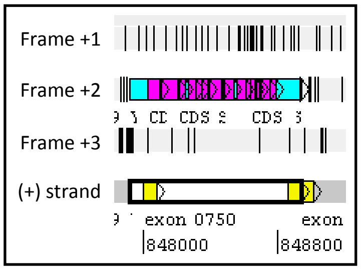

Oligo evidence:

actgccgcccgctaatagtcgcttaattggcgaaaatacaacctttactgttccaaatgatggtcgccct
gcctgtagcgtctcaggatgagcaacaggcacaggctgaagcactactgtcaacgacggatgaaaagtga

Peptide evidence:

DTPTMVTSISQK
EGIVINLAELR
FINDSQTDAK
GEYLIHGTNADFGIGLR
IGLLGMLEANPGTDPYLPLPGSVLTIPNQMLLPDTPR
IPNPTWTPTANIR
KNYLAEGITLPSVVPAGPDNPMGQFALR
LIGENTTFTVPNDGRPLEAIAADYK
LRPDDIEALFNSVPK
LSAGRGEYLIHGTNADFGIGLR
NYLAEGITLPSVVPAGPDNPMGQFALR
RYVEVHQPLSR
VDSDDPQTMPIAIGSGLQK
VIVYPIGIGQLGR
VQIINEPVKYAVEPDGKR
YAVEPDGKR
YVEVHQPLSR

Current sequence (peptide evidence in red): 339aa

MKRALTLIGMLFATCLAGSLTAANANEYPLPPANSRLIGENTTFTVPNDGRPLEAIAADYKIGLLGMLEANPGTDPYLPLPGSVLTIPNQMLLPDTPREGIVINLAELRLYYYPKGQNKVIVYPIGIGQLGRDTPTMVTSISQKIPNPTWTPTANIRKNYLAEGITLPSVVPAGPDNPMGQFALRLSAGRGEYLIHGTNADFGIGLRVSSGCIRLRPDDIEALFNSVPKGTRVQIINEPVKYAVEPDGKRYVEVHQPLSRVDSDDPQTMPIAIGSGLQKFINDSQTDAKAVQDAIVRRSGMPTIVTVGEASTQQAPTPVASQDEQQAQAEALLSTTDEK

Strain: Y. pseudotuberculosis PB1/+
Gene: YPTS_2382

Classification: ErfK/YbiS/YcfS/YnhG family protein
Boundaries of gene: complement (2641423..2642487)

Oligo evidence:

gtctcaggatgagcaacaggcacaggctgaagcactacaaccgacagaagagtcagtgatgcctaagctg
actgccgcccgctaatagtcgcttaattggcgaaaatacaacctttactgttccaaatgatggtcgccct

Peptide evidence:

DTPTMVTSISQK
EGIVINLAELR
FINDSQTDAK
GEYLIHGTNADFGIGLR
IGLLGMLEANPGTDPYLPLPGSVLTIPNQMLLPDTPR
IPNPTWTPTANIR
IPNPTWTPTANIRK
KNYLAEGITLPSVVPAGPDNPMGQFALR
LIGENTTFTVPNDGRPLEAIAADYK
LRPDDIEALFNSVPK
LSAGRGEYLIHGTNADFGIGLR
LVQPGPVYSENN
NYLAEGITLPSVVPAGPDNPMGQFALR
RYVEVHQPLSR
SGMPTIVTVGEASTQQAPTPVASQDEQQAQAEALQPTEESVMPK
VDSDDPQTMPIAIGSGLQK
VIVYPIGIGQLGR
VQIINEPVKYAVEPDGKR
YLAEGITLPSVVPAGPDNPMGQFALR
YVEVHQPLSR

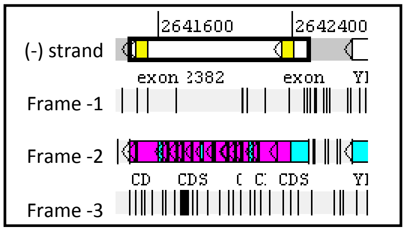

Current sequence (peptide evidence in red): 354aa

MKRALTLIGMLFATCLAGSLTAANANEYPLPPANSRLIGENTTFTVPNDGRPLEAIAADYKIGLLGMLEANPGTDPYLPLPGSVLTIPNQMLLPDTPREGIVINLAELRLYYYPKGQNKVIVYPIGIGQLGRDTPTMVTSISQKIPNPTWTPTANIRKNYLAEGITLPSVVPAGPDNPMGQFALRLSAGRGEYLIHGTNADFGIGLRVSSGCIRLRPDDIEALFNSVPKGTRVQIINEPVKYAVEPDGKRYVEVHQPLSRVDSDDPQTMPIAIGSGLQKFINDSQTDAKAVQDAIVRRSGMPTIVTVGEASTQQAPTPVASQDEQQAQAEALQPTEESVMPKLVQPGPVYSENN

Error 24

Strain: Y. pestis CO92
Gene: YPO2412

Classification: expressed pseudogene
Boundaries of gene: complement (2713182..2714645)

Oligo evidence:

tttcgagcatttctactatcggcagcaacctaagcaagttcaacaattagcggattatgttattgcccgc
ctgaatttgatgatttagcggcgttacccccagattggggaaaacacctcgagatctcttgttccagctg

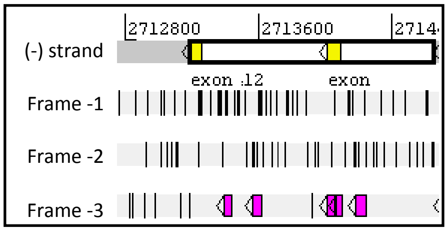

Peptide evidence:

DSQDNDLLTGLLSLMIK
LQQESIDDDQR
EFLASEALHHLGIPTSR
FGHFEHFYYR
QVQQLADYVIAR

Proposed sequence (peptide evidence in red): 487aa
MKPRMEYAPEFDNSYARQLSGFYTRLQPTPLKGARLLYHSKPLAQELGLDAHWFTEPKTAVWAGEALLPGMEPLAQVYSGHQFGMWAGQLGDGRGILLGEQRLNDGRYMDWHLKGAG
LTPYSRMGDGRAVLRSVIREFLASEALHHLGIPTSRALTIVTSDHPIYREQTERGAMLLRVAESHIRFGHFEHFYYRQQPKQVQQLADYVIARHWPQWVGHQECYRLWFTDVVERTARLMA
HW#TVGFAHGVMNTDNMSILGITMDYGPFGFLDDYVPGYICNHSDHQGRYAYDNQPAVALWNLHRLGHALSGLMSADQLQLALEAYEPALMVAYGEQMRAKLGFLERDSQDNDLLTG
LLSLMIKEGRDYTRTFRLLSEVEVHSAQSPLRDDFIDRAAFDDWYRRYRSRLQQESIDDDQRQQSMKAANPKYILRNYLAQQAITQAEKDDIQPLQRLHQALQQPFTDQPEFDDLAALPPD
WGKHLEISCSS

Strain: Y. pestis pestoides F
Gene: YPDSF_0737

Classification: annotated as hypothetical protein
Boundaries of gene: 828912..830375

Oligo evidence:

tttcgagcatttctactatcggcagcaacctaagcaagttcaacaattagcggattatgttattgcccgc
gcaaaccgtcgggtttgctcatggtgtgatgaataccgataatatgtcaattcttggcattactatggat
ctgaatttgatgatttagcggcgttacccccagattggggaaaacacctcgagatctcttgttccagctg

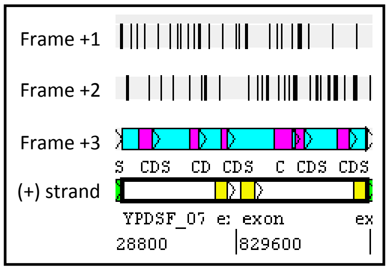

Peptide evidence:

DSQDNDLLTGLLSLMIK
EFLASEALHHLGIPTSR
LGHALSGLMSADQLQLALEAYEPALMVAYGEQMR
LLYHSKPLAQELGLDAHWFTEPK
NYLAQQAITQAEKDDIQPLQR
QVQQLADYVIAR

Current sequence (peptide evidence in red): 487aa
MKPRMEYAPEFDNSYARQLSGFYTRLQPTPLKGARLLYHSKPLAQELGLDAHWFTEPKTAVWAGEALLPGMEPLAQVYSGHQFGMWAGQLGDGRGILLGEQRLNDGRYMDWHLKGAG
LTPYSRMGDGRAVLRSVIREFLASEALHHLGIPTSRALTIVTSDHPIYREQTERGAMLLRVAESHIRFGHFEHFYYRQQPKQVQQLADYVIARHWPQWVGHQECYRLWFTDVVERTARLMA
HWQTVGFAHGVMNTDNMSILGITMDYGPFGFLDDYVPGYICNHSDHQGRYAYDNQPAVALWNLHRLGHALSGLMSADQLQLALEAYEPALMVAYGEQMRAKLGFLERDSQDNDLLT
GLLSLMIKEGRDYTRTFRLLSEVEVHSAQSPLRDDFIDRAAFDDWYRRYRSRLQQESIDDDQRQQSMKAANPKYILRNYLAQQAITQAEKDDIQPLQRLHQALQQPFTDQPEFDDLAALPPD
WGKHLEISCSS

Strain: Y. pseudotuberculosis PB1/+
Gene: YPTS_2396

Classification: annotated as hypothetical protein
Boundaries of gene: complement (2660170..2661633)

Oligo evidence:

tttcgagcatttctactatcggcagcaacctaagcaagttcaacaattagcggattatgttattgcccgc
gcaaaccgtcgggtttgctcatggtgtgatgaataccgataatatgtcaattcttggcattactatggat
ctgaatttgatgatttagcggcgttacccccagattggggaaaacacctcgagatctcttgttccagctg

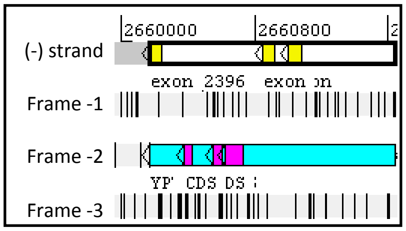

Peptide evidence:

DSQDNDLLTGLLSLMIK
LGHALSGLMSADQLQLALEAYEPALMVAYGEQMR
LQQESIDDDQR

Current sequence (peptide evidence in red): 487aa
MKPRMEYAPEFDNSYARQLSGFYTRLQPTPLKGARLLYHSKPLAQELGLDAHWFTEPKTAVWAGEALLPGMEPLAQVYSGHQFGMWAGQLGDGRGILLGEQRLNDGRYMDWHLKGAG
LTPYSRMGDGRAVLRSVIREFLASEALHHLGIPTSRALTIVTSDHPIYREQTERGAMLLRVAESHIRFGHFEHFYYRQQPKQVQQLADYVIARHWPQWVGHQECYRLWFTDVVERTARLMA
HWQTVGFAHGVMNTDNMSILGITMDYGPFGFLDDYVPGYICNHSDHQGRYAYDNQPAVALWNLHRLGHALSGLMSADQLQLALEAYEPALMVAYGEQMRAKLGFLERDSQDNDLLT
GLLSLMIKEGRDYTRTFRLLSEVEVHSAQSPLRDDFIDRAAFDDWYRRYRSRLQQESIDDDQRQQSMKAANPKYILRNYLAQQAITQAEKDDIQPLQRLHQALQQPFTDQPEFDDLAALPPD
WGKHLEISCSS

Error 25

Strain: Y. pestis CO92
Gene: YPO2570

Classification: expressed pseudogene
Boundaries of gene: 2885536..2885790

Oligo evidence:

gtcagtgctgaagtcgatcatgttgacctcggttccgcaaaaggtacgccaagcgatatctttatcggta

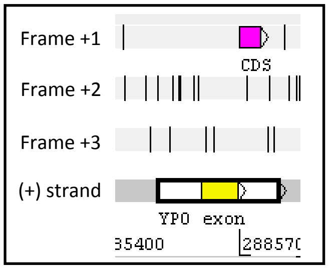

Peptide evidence:

DIAEQLVAQAVGGK

Proposed sequence (peptide evidence in red): 84aa
MKITVVCGNGLGTSLMMEISIKSILKELAVSAEVDHVDLGSAKGTPSDIFIGTKDIAEQLVAQAVGGKIVALDNMIDKKARACP

Strain: Y. pestis pestoides F
Gene: YPDSF_1977

Classification: hypothetical protein
Boundaries of gene: 2235695..2235982

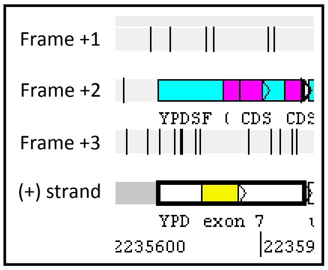

Oligo evidence:

gtcagtgctgaagtcgatcatgttgacctcggttccgcaaaaggtacgccaagcgatatctttatcggta

Peptide evidence:

DIAEQLVAQAVGGK
GTPSDIFIGTKDIAEQLVAQAVGGK
LSAALTELGAL

Current sequence (peptide evidence in red): 95aa
MKITVVCGNGLGTSLMMEISIKSILKELAVSAEVDHVDLGSAKGTPSDIFIGTKDIAEQLVAQAVGGKIVALDNMIDKKAMKERLSAALTELGAL

Strain: Y. pseudotuberculosis PB1/+
Gene: YPTS_2695

Classification: phosphotransferase system lactose/cellobiose-specific IIB subunit
Boundaries of gene: 2995345..2995632

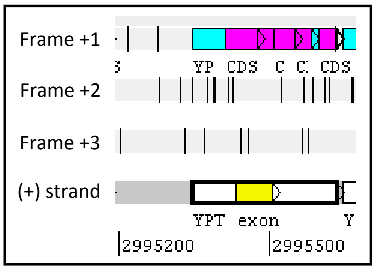

Oligo evidence:

gtcagtgctgaagtcgatcatgttgacctcggttccgcaaaaggtacgccaagcgatatctttatcggta

Peptide evidence:

DIAEQLVAQAVGGK
GTPSDIFIGTKDIAEQLVAQAVGGK
IVALDNMIDKK
LSAALTELGAL
SILKELAVSAEVDHVDLGSAK

Current sequence (peptide evidence in red): 95aa
MKITVVCGNGLGTSLMMEISIKSILKELAVSAEVDHVDLGSAKGTPSDIFIGTKDIAEQLVAQAVGGKIVALDNMIDKKAMKERLSAALTELGAL

Error 26

Strain: Y. pestis CO92
Gene: YPO2644

Classification: expressed pseudogene
Boundaries of gene: 2970789..2971556

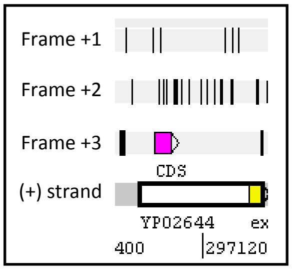

Oligo evidence:

ggtgtgggtttgtcggcgtcaagagattgcggagcattgggttaaacatcacccgtttgaaggtatcaat

Peptide evidence:

VFGVAMALERNPLIVEAIKCAGYDVVCHGWR

Proposed sequence (peptide evidence in red): 255aa
VWITIAPFVTHMSMESLYEYGTRAGFWRIHQEFVSRGLPMTVFGVAMALERNPLIVEAIKCAGYDVVCHGWRWLHYQHVDEQTEREHMQRAIKILHDLFGQPPAGWYTGRDSPNTRRLV
VENGHLLYDSDYYGDDLPFWSQVRGVDGSTTPHLVVPYTLDANDMRFASAQGFNSSEQFYTYLKDSFDVLYAEGETAPKMMSVGMHCRLLGRPGRFRALQRFLDYIQQHERVWVCRRQE
IAEHWVKHHPFEGINGR

Strain: Y. pestis pestoides F
Gene: YPDSF_1628

Classification: annotated as hypothetical protein
Boundaries of gene: complement (1823526..1824476)

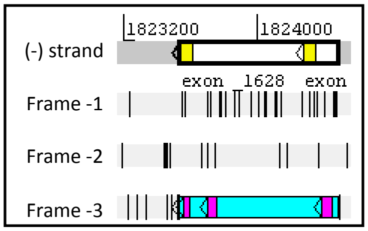

Oligo evidence:

aaataacgttctacatggcgatgcgggatctgaacaatttctttccgatattatcggtgcggacagttat
ggtgtgggtttgtcggcgtcaagagattgcggagcattgggttaaacatcacccgtttgaaggtatcaat

Peptide evidence:

DSFDVLYAEGETAPK
DLAGYGGQPPVANWPGQAR
RQEIAEHWVK

Current sequence (peptide evidence in red): 316aa
MHETELNHDYPRDLAGYGGQPPVANWPGQARIAVQFVLNIEEGAENNVLHGDAGSEQFLSDIIGADSYPDRHMSMESLYEYGTRAGFWRIHQEFVSRGLPMTVFGVAMALERNPLIVEAI
KCAGYDVVCHGWRWLHYQHVDEQTEREHMQRAIKILHDLFGQPPAGWYTGRDSPNTRRLVVENGHLLYDSDYYGDDLPFWSQVRGVDGSTTPHLVVPYTLDANDMRFASAQGFNSSE
QFYTYLKDSFDVLYAEGETAPKMMSVGMHCRLLGRPGRFRALQRFLDYIQQHERVWVCRRQEIAEHWVKHHPFEGINGR

Strain: Y. pseudotuberculosis PB1/+
Gene: YPTS_3080

Classification: annotated as urate catabolism protein
Boundaries of gene: complement (3408841..3409791)

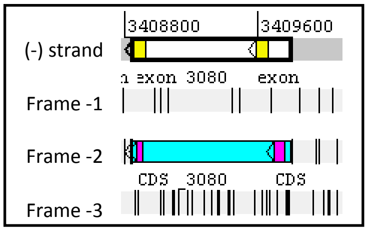

Oligo evidence:

aaataacgttctacatggcgatgcgggatctgaacaatttctttccgatattatcggtgcggacagttat
ggtgtgggtttgtcggcgtcaagagattgcggagcattgggttaaacatcacccgtttgaaggtatcaat

Peptide evidence:

DLAGYGGQPPVANWPGQAR
RQEIAEHWVK

Current sequence (peptide evidence in red): 316aa
MHETELNHDYPRDLAGYGGQPPVANWPGQARIAVQFVLNIEEGAENNVLHGDAGSEQFLSDIIGADSYPDRHMSMESLYEYGTRAGFWRIHQEFVSRGLPMTVFGVAMALERNPLIVEAI
KCAGYDVVCHGWRWLHYQHVDEQTEREHMQRAIKILHDLFGQPPAGWYTGRDSPNTRRLVVENGHLLYDSDYYGDDLPFWSQVRGVDGSTTPHLVVPYTLDANDMRFASAQGFNSSE
QFYTYLKDSFDVLYAEGETAPKMMSVGMHCRLLGRPGRFRALQRFLDYIQQHERVWVCRRQEIAEHWVKHHPFEGINGR

Error 27

Strain: Y. pestis CO92
Gene: YPO2698

Classification: expressed pseudogene
Boundaries of gene: 3026599..3027255

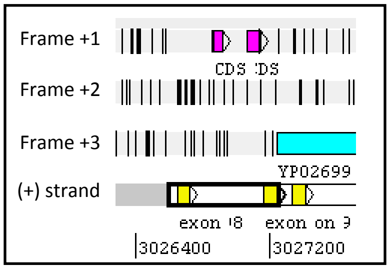

Oligo evidence:

ttagaaccaccggtaacattagccagccagcaacggatttggggcttagctgatcgcctaattcatcatg
tgtttaatccagtggcgatgccaccgaccttattgcgacctggggataatgtgcgctttctgccgctaaa

Peptide evidence:

DITIPVMYGGEVGPDLADVAR
LVVAPGSVGIGGSQTGIYPLATPGGW
PVMYGGEVGPDLADVAR

Proposed sequence (peptide evidence in red): 218aa
VQRARCYLLGESAVVLELEPPVTLASQQRIWGLADRLIHHADILEVIPGMNNLTLLLADPHNTALDAIERLQRWWEESESLIPESRDITIPVMYGGEVGPDLADVARHTGMSERQVVECHAG
ARYIVYFLGFQPGFSYLGGMPEQLATPRRADPRLVVAPGSVGIGGSQTGIYPLATPGGW+LIGRTSLALFNPVAMPPTLLRPGDNVRFLPLKEGVC

Strain: Y. pestis pestoides F
Gene: YPDSF_1573

Classification: hypothetical protein
Boundaries of gene: complement (1769395..1769940)

Oligo evidence:

ttagaaccaccggtaacattagccagccagcaacggatttggggcttagctgatcgcctaattcatcatg

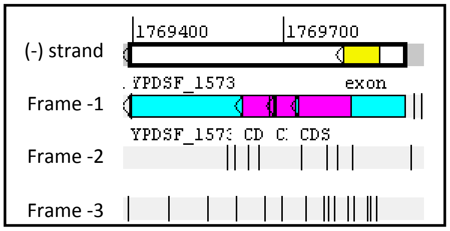

Peptide evidence:

DITIPVMYGGEVGPDLADVAR
LIHHADILEVIPGMNNLTLLLADPHNTALDAIER
PVMYGGEVGPDLADVAR
WWEESESLIPESR

Current sequence (peptide evidence in red): 181aa
VQRARCYLLGESAVVLELEPPVTLASQQRIWGLADRLIHHADILEVIPGMNNLTLLLADPHNTALDAIERLQRWWEESESLIPESRDITIPVMYGGEVGPDLADVARHTGMSERQVVECHAG
ARYIVYFLGFQPGFSYLGGMPEQLATPRRADPRLVVAPGSVGIGGSQTGIYPLATPGGW

Strain: Y. pseudotuberculosis PB1/+
Gene: YPTS_3023

Classification: allophanate hydrolase subunit 1
Boundaries of gene: complement (3353932..3354588)

Oligo evidence:

tgtttaatccagtggcgatgccaccgaccttattgcgacctggggataatgtgcgctttctgccgctaaa
ttagaaccaccggtaacattagccagccagcaacggatttggggcttagctgatcgcctaattcatcatg

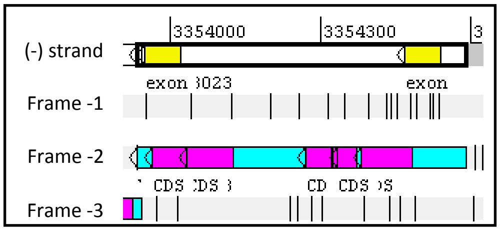

Peptide evidence:

DITIPVMYGGEVGPDLADVAR
IPVMYGGEVGPDLADVAR
LIHHADILEVIPGMNNLTLLLADPHNTALDAIER
LVVAPGSVGIGGSQTGIYPLATPGGWQLIGR
PVMYGGEVGPDLADVAR
TSLALFNPVAMPPTLLRPGDNVR
WWEESESLIPESR

Current sequence (peptide evidence in red): 218aa
VQRARCYLLGESAVVLELEPPVTLASQQRIWGLADRLIHHADILEVIPGMNNLTLLLADPHNTALDAIERLQRWWEESESLIPESRDITIPVMYGGEVGPDLADVARHTGMSERQVVECHAG
ARYIVYFLGFQPGFSYLGGMPEQLATPRRADPRLVVAPGSVGIGGSQTGIYPLATPGGWQLIGRTSLALFNPVAMPPTLLRPGDNVRFLPLKEGVC

Error 28

Strain: Y. pestis CO92
Gene: YPO2909

Classification: expressed pseudogene
Boundaries of gene: Frame+1: 3252487..3253062
Frame+2:3253064..3253420

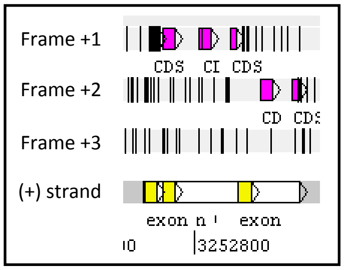

Oligo evidence:

acataaggctagatcgttacatcatgagtaaaagattcataaaaaaagaagggttagcattagtctctat
ttaaaactattgatgatctatcaggtgattttggtatttcagtcggtgtgattcaacacgccttgaaagt
aaaaatccgccgggttgggtttgactcacgttcacctgatcaatgtttattgactgaaattaagtttgcc

Peptide evidence:

EGLALVSMAR
EGLASGLKEQFTLLPLYFAHMR
EQFTLLPLYFAHMR
GSYVDGHQLIFR
TIDDLSGDFGISVGVIQHALK
AVQSGIVEPR
GDIDAVIWNQEQIVPSEYLQSIK

Proposed sequence (peptide evidence in red):
Frame+1: 192aa
MPGNIRLDRYIMSKRFIKKEGLALVSMARYLIGERSGNRLKTIDDLSGDFGISVGVIQHALKVLEADGAIVVERRGRNGTRLLDLNMTLLLQQADLGNMVCAMPLPYTKLYEGLASGLKEQFTL
LPLYFAHMRGAEVRIECLIDGVYDMAVVSHLAAKNYLKQEKVTLALNLGRGSYVDGHQLIFRRGEQKK

Frame+2: 118aa
IRRVGFDSRSPDQCLLTEIKFAGQPIERVELSYSNCIPHLIRGDIDAVIWNQEQIVPSEYLQSIKLQGDERYIQASQAVILIRPDNYPIKLLLERGINQTQLLRHQRAVQSGIVEPRY

Strain: Y. pestis pestoides F
Gene: YPDSF_2255

Classification: hypothetical protein
Boundaries of gene: 2554072..2554971

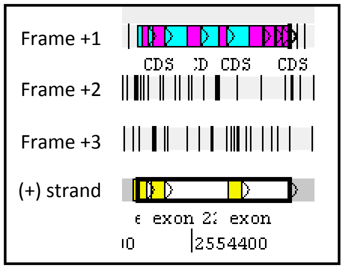

Oligo evidence:

acataaggctagatcgttacatcatgagtaaaagattcataaaaaaagaagggttagcattagtctctat
ttaaaactattgatgatctatcaggtgattttggtatttcagtcggtgtgattcaacacgccttgaaagt
aaaaatccgccgggttgggtttgactcacgttcacctgatcaatgtttattgactgaaattaagtttgcc

Peptide evidence:

EGLALVSMAR
LYEGLASGLKEQFTLLPLYFAHMR
TIDDLSGDFGISVGVIQHALK
AVQSGIVEPR
GDIDAVIWNQEQIVPSEYLQSIK
GINQTQLLR
GSYVDGHQLIFR
YIQASQAVILIRPDNYPIK

Current sequence (peptide evidence in red): 299aa
MSKRFIKKEGLALVSMARYLIGERSGNRLKTIDDLSGDFGISVGVIQHALKVLEADGAIVVERRGRNGTRLLDLNMTLLLQQADLGNMVCAMPLPYTKLYEGLASGLKEQFTLLPLYFAHMRG
AEVRIECLIDGVYDMAVVSHLAAKNYLKQEKVTLALNLGRGSYVDGHQLIFRRGEQKKIRRVGFDSRSPDQCLLTEIKFAGQPIERVELSYSNCIPHLIRGDIDAVIWNQEQIVPSEYLQSIKLQG
DERYIQASQAVILIRPDNYPIKLLLERGINQTQLLRHQRAVQSGIVEPRY

Strain: Y. pseudotuberculosis PB1/+
Gene: YPTS_2980

Classification: hypothetical protein
Boundaries of gene: 3311014..3311946

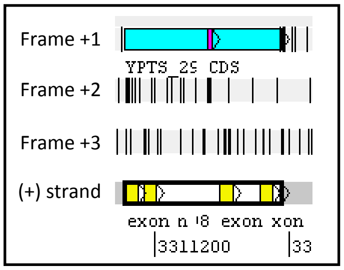

Oligo evidence:

acataaggctagatcgttacatcatgagtaaaagattcataaaaaaagaagggttagcattagtctctat
ttaaaactattgatgatctatcaggtgattttggtatttcagtcggtgtgattcaacacgccttgaaagt
aaaaatccgccgggttgggtttgactcacgttcacctgatcaatgtttattgactgaaattaagtttgcc
tatccttatccgcccagataactatccgatcaagctactgctggaaagaggcattaaccaaacacagtta

Peptide evidence:

VTLALNLGR

Current sequence (peptide evidence in red): 310aa
LPGNIRLDRYIMSKRFIKKEGLALVSMARYLIGERSGNRLKTIDDLSGDFGISVGVIQHALKVLEADGAIVVERRGRNGTRLLDLNMTLLLQQADLGNMVCAMPLPYTKLYEGLASGLKEQFTLL
PLYFAHMRGAEVRIECLIDGVYDMAVVSHLAAKNYLKQEKVTLALNLGRGSYVDGHQLIFRRGEQKKIRRVGFDSRSPDQCLLTEIKFAGQPIERVELSYSNCIPHLIRGDIDAVIWNQEQIVPS
EYLQSIKLQGDERYIQASQAVILIRPDNYPIKLLLERGINQTQLLRHQRAVQSGIVEPRY

Error 29

Strain: Y. pestis CO92
Gene: YPO3207

Classification: expressed pseudogene
Boundaries of gene: 3570770..3574477

Oligo evidence:

aacaacaacacgtacagcaatatcagcagcaacaggtgctcgttgagcaagagtatcaacaggcactacc
aattttgagcctacgtctgaaaaacattaactcattacagggcgaatggaagatagatttcactgccgaa

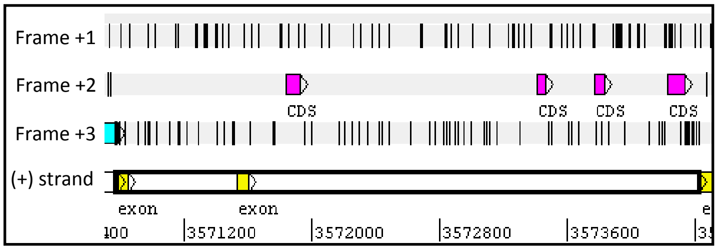

Peptide evidence:

IVPLDNLITQQQQTLSQLAGQIQQLR
LQQQQAELAQQNAEK
SSELTLLQQQSEQLLAQLK
TSIDSLFLDEGFGTLDAETLDTALDALDSLNASGK

Proposed sequence (peptide evidence in red): 1235aa
MKILSLRLKNINSLQGEWKIDFTAEPFASNGLFAITGPTGAGKTTLLDAICLALYHQTPRLIVTPSQNELMTRHTAESLAEVEFDVKGIRYRAFWSQRRARNSPDGNLQAPKVELALCENGKILADKVRDKLDMIAAITGLDF
GRFTKSMMLSQGQFAAFLNADANDRAELLEELTGTDIYGRLSERVFEKHKQAKIDLDALHQRASGIELLNEEQRLALAQQIDALSQQEQQLSKEQLVTQNQINWLTGWQQQQQHVQQYQQQQVLVEQEYQQALPG
LQRLARSEPAEKLRPLQRERDRSQKDLQQTQQRITALAQQQQQYLAQLTPLTQAVEQATAARQQQQLNQHEQETLIEQRIVPLDNLITQQQQTLSQLAGQIQQLRAKEQQNSQQLALNEQKLLQTHQRLQQLADYA
NLHAHHQHWEKHLPLWHEQFRQLQLQQQQSAQSEQQLHQQTTLLATLQQQATTLSAQEKQQQVALAEARAQASYLQQKLLVLEQQQPSAQLRQQLNEFNEQRQICQQLAALSPLAQQIQALYDKQQQQFTAQ
QQQLKQLEQQLTEKRQLYQQQKQHLVDLEALLEREKQIVTLEAERAKLQPGDACPLCGAVEHPAITAYQAVKPSETAVRVAKLRLQVEQLYTEGTELRTQVASMQQHQQRIEQELQDHRQQLAAYQQRWQTLAQPL
SLAFTLNEPDALALWLEQHEQQEQACQLKLVEYERLTQQYQQAKDILTQLEQRQQEHQQQLALITERQKNAQQTYQQLQSQYQHQQEALIAQQQVLNHTLTELSLSVPDADQQQDWLAQREEECQRWQQHQQE
QQRLTIEQKTLETRIENERRHLQECIDQLSALSQQRQQAETLLQQQIQQRQALFGEDIVAEVRQRLRLQQQQAELAQQNAEKALQQAQSQLNRLSGELTGLEQQCQQYQQRATTTQAELQQALSTSEFADETALTAAL
LSEEERQHLQQLQQQLNERRQQAQIRLQQAREILDQHLQLCPQGVDKSSELTLLQQQSEQLLAQLKTTTLRQGELRNQLESDTTRRHNQRTLFEQIERSQQQYDDWSYLNQLIGSKEGDKFRKFAQGLTLDHLVYLAN
NQLSRLHGRYLLQRKTTDALELQVVDTWQADAIRDTRTLSGGESFLVSLALALALSDLVSHKTSIDSLFLDEGFGTLDAETLDTALDALDSLNASGKTIGVISHVEAMKDRIPVQIKVKKVNGLGVCQRRMKSDPLISPPTA
QY

Strain: Y. pestis pestoides F
Gene: YPDSF_2840
Oligo evidence:

Classification: hypothetical protein
Boundaries of gene: 3216993..3220682

aattttgagcctacgtctgaaaaacattaactcattacagggcgaatggaagatagatttcactgccgaa
cagataaaagtgaaaaaagttaacgggttaggcgtcagccgtttggatataatgccttccgagtcaatcaag

Peptide evidence:

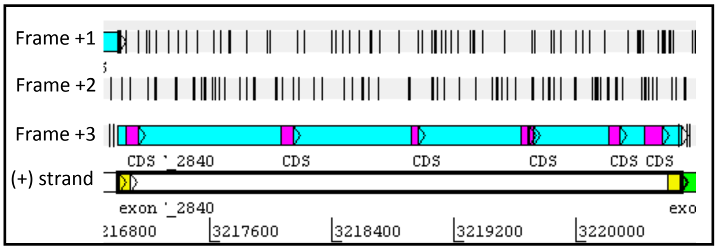

ALQQAQSQLNR
IDFTAEPFASNGLFAITGPTGAGK
IVPLDNLITQQQQTLSQLAGQIQQLR
KFAQGLTLDHLVYLANNQLSR
LQQQQAELAQQNAEK
TQVASMQQHQQR
TSIDSLFLDEGFGTLDAETLDTALDALDSLNASGK

Current sequence (peptide evidence in red): 1229aa
MKILSLRLKNINSLQGEWKIDFTAEPFASNGLFAITGPTGAGKTTLLDAICLALYHQTPRLIVTPSQNELMTRHTAESLAEVEFDVKGIRYRAFWSQRRARNSPDGNLQAPKVELALCENGKILADKVRDKLDMIAAITGLDF
GRFTKSMMLSQGQFAAFLNADANDRAELLEELTGTDIYGRLSERVFEKHKQAKIDLDALHQRASGIELLNEEQRLALAQQIDALSQQEQQLSKEQLVTQNQINWLTGWQQQQQHVQQYQQQQVLVEQEYQQALPG
LQRLARSEPAEKLRPLQRERDRSQKDLQQTQQRITALAQQQQQYLAQLTPLTQAVEQATAARQQQQLNQHEQETLIEQRIVPLDNLITQQQQTLSQLAGQIQQLRAKEQQNSQQLALNEQKLLQTHQRLQQLADYA
NLHAHHQHWEKHLPLWHEQFRQLQLQQQQSAQSEQQLHQQTTLLATLQQQATTLSAQEKQQQVALAEARAQASYLQQKLLVLEQQQPSAQLRQQLNEFNEQRQICQQLAALSPLAQQIQALYDKQQQQFTAQ
QQQLKQLEQQLTEKRQLYQQQKQHLVDLEALLEREKQIVTLEAERAKLQPGDACPLCGAVEHPAITAYQAVKPSETAVRVAKLRLQVEQLYTEGTELRTQVASMQQHQQRIEQELQDHRQQLAAYQQRWQTLAQPL
SLAFTLNEPDALALWLEQHEQQEQACQLKLVEYERLTQQYQQAKDILTQLEQRQQEHQQQLALITERQKNAQQTYQQLQSQYQHQQEALIAQQQVLNHTLTELSLSVPDADQQQDWLAQREEECQRWQQHQQE
QQRLTIEQKTLETRIENERRHLQECIDQLSALSQQRQQAETLLQQQIQQRQALFGEDIVAEVRQRLRLQQQQAELAQQNAEKALQQAQSQLNRLSGELTGLEQQCQQYQQRATTTQAELQQALSTSEFADETALTAAL
LSEEERQHLQQLQQQLNERRQQAQIRLQQAREILDQHLQLCPQGVDKSSELTLLQQQSEQLLAQLKTTTLRQGELRNQLESDTTRRHNQRTLFEQIERSQQQYDDWSYLNQLIGSKEGDKFRKFAQGLTLDHLVYLAN
NQLSRLHGRYLLQRKTTDALELQVVDTWQADAIRDTRTLSGGESFLVSLALALALSDLVSHKTSIDSLFLDEGFGTLDAETLDTALDALDSLNASGKTIGVISHVEAMKDRIPVQIKVKKVNGLGVSRLDNAFRVNQD

Strain: Y. pseudotuberculosis PB1/+
Gene: YPTS_0956

Classification: SMC domain-containing protein
Boundaries of gene: complement (1083806..1087495)

Oligo evidence:

cagataaaagtgaaaaaagttaacgggttaggcgtcagccgtttggatataatgccttccgagtcaatcaag
aattttgagcctacgtctgaaaaacattaactcattacagggcgaatggaagatagatttcactgccgaa

Peptide evidence:

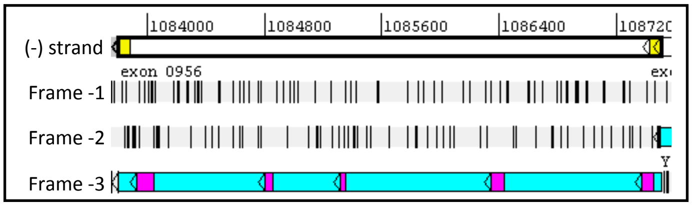

IDFTAEPFASNGLFAITGPTGAGK
IVPLDNLITQQQQTLSQLAGQIQQLR
LQQQQAELAQQNAEK
LTQQYQQAK
TSIDSLFLDEGFGTLDAETLDTALDALDSLNASGK

Current sequence (peptide evidence in red): 1229aa
MKILSLRLKNINSLQGEWKIDFTAEPFASNGLFAITGPTGAGKTTLLDAICLALYHQTPRLIVTPSQNELMTRHTAESLAEVEFDVKGIRYRAFWSQRRARNSPDGNLQAPKVELALCENGKILADKVRDKLDMIAAITGLDF
GRFTKSMMLSQGQFAAFLNADANDRAELLEELTGTDIYGRLSERVFEKHKQAKIDLDALHQRASGIELLNEEQRLALAQQIDALSQQEQQLSKEQLVTQNQINWLTGWQQQQQHVQQYQQQQVLVEQEYQQALPG
LQRLARSEPAEKLRPLQRERDRSQKDLQQTQQRITALAQQQQQYLAQLTPLTQAVEQATAARQQQQLNQHEQETLIEQRIVPLDNLITQQQQTLSQLAGQIQQLRAKEQQNSQQLALNEQKLLQTHQRLQQLADYA
NLHAHHQHWEKHLPLWHEQFRQLQLQQQQSAQSEQQLHQQTTLLATLQQQATTLSAQEKQQQVALAEARAQASYLQQKLLVLEQQQPSAQLRQQLNEFNEQRQICQQLAALSPLAQQIQALYDKQQQQFTAQ
QQQLKQLEQQLTEKRQLYQQQKQHLVDLEALLEREKQLVTLEAERAKLQPGDACPLCGAVEHPAITAYQAVKPSETAVRVAKLRLQVEQLYTEGTELRTQVASMQQHQQRIEQELQDHRQQLAAYQQRWQTLAQPL
SLAFTLNEPDALALWLEQHEQQEQACQLKLVEYERLTQQYQQAKDILTQLEQRQQEHQQQLALITERQKNAQQTYQQLQSQYQHQQEALIAQQQVLNHTLTELSLSVPDADQQQNWLAQREEECQRWQQHQQE
QQRLTIEQKTLETRIENERRHLQECIDQLSALSQQRQQAETLLQQQIQQRRALFGEDIVAEVRQRLRLQQQQAELAQQNAEKALQQVQSQLNRLSGELTGLEQQCQQYQQRATTTQAELQQALSTSEFADETALTAAL
LSEEERQHLQQLQQQLNERRQQAQIRLQQAREILDQHLQLCPQGVDKSSELTLLQQQSEQLLAQLKTTTLRQGELRNQLESDTTRRHNQRTLFEQIERSQQQYDDWSYLNQLIGSKEGDKFRKFAQGLTLDHLVYLAN
NQLSRLHGRYLLQRKTTDALELQVVDTWQADAIRDTRTLSGGESFLVSLALALALSDLVSHKTSIDSLFLDEGFGTLDAETLDTALDALDSLNASGKTIGVISHVEAMKDRIPVQIKVKKVNGLGVSRLDNAFRVNQD

Error 30

Strain: Y. pestis CO92
Gene: YPO3370

Classification: expressed pseudogene
Boundaries of gene: frame-1: complement (3757695..3758154)
frame-3: complement (3757428..3757700)

Oligo evidence:

gtggagggtattgagcgttataacaacctcaataaagttgaaccaatgaatcgggcgttggaggctttag
aatcgccaagtttatcaatacctcacacaacatggcctgagttatcacccactgtgggaacaaggttatt

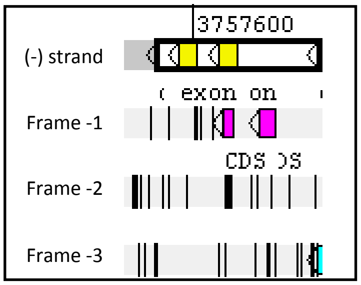

Peptide evidence:

QRPDIPVILTDTGYLFPETYR
YNNLNKVEPMNR

Proposed sequence (peptide evidence in red):
Frame-1: 154aa
VSQFNLSELNALPKAKQAAALVLVNGQLEHLTAQERVSWALDNLPGEFVLSSSFGIQAAVCLHLVTRQRPDIPVILTDTGYLFPETYRFIDDLTEKLQLNLQVFRAAHSPAWQEARYGKLWEQGVEGIERYNNLNKVEPMNRALEALGAQTWFA

Frame-3: 90aa
GLRREQSGGRSQLPVLALQRGIFKLLPIIDWDNRQVYQYLTQHGLSYHPLWEQGYLSVGDTHTTRKWEPGMSEEETRFFGLKRECGLHEG

Strain: Y. pestis pestoides F
Gene: YPDSF_2989 / YPDSF_2990

Classification:YPDSF_2989 annotated as hypothetical protein
YPDSF_2990 annotated as phosphoadenylyl-sulfate reductase (thioredoxin)
Boundaries of gene: YPDSF_2989 Frame+2: 3396791..3397258
YPDSF_2990 Frame+3: 3397233..3397517

Oligo evidence:

gtggagggtattgagcgttataacaacctcaataaagttgaaccaatgaatcgggcgttggaggctttag
aatcgccaagtttatcaatacctcacacaacatggcctgagttatcacccactgtgggaacaaggttatt

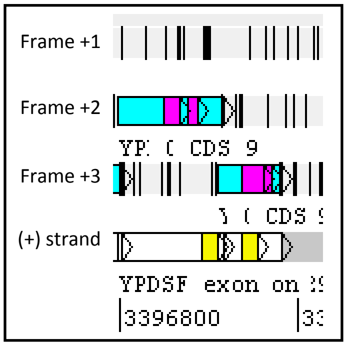

Peptide evidence:

AAHSPAWQEAR
QRPDIPVILTDTGYLFPETYR
KWEPGMSEEETR
QVYQYLTQHGLSYHPLWEQGYLSVGDTHTTR

Current sequence (peptide evidence in red):
Frame2: 154aa
VSQFNLSELNALPKAKQAAALVLVNGQLEHLTAQERVSWALDNLPGEFVLSSSFGIQAAVCLHLVTRQRPDIPVILTDTGYLFPETYRFIDDLTEKLQLNLQVFRAAHSPAWQEARYGKLWEQGVEGIERYNNLNKVEPMNRALEALGAQTWFA

Frame3: 90aa
GLRREQSGGRSQLPVLALQRGIFKLLPIIDWDNRQVYQYLTQHGLSYHPLWEQGYLSVGDTHTTRKWEPGMSEEETRFFGLKRECGLHEG

Strain: Y. pseudotuberculosis PB1/+
Gene: YPTS_0794

Classification: annotated as phosphoadenosine phosphosulfate reductase
Boundaries of gene: 899444..900178

Oligo evidence:

gtttatcaatacctcacacaacatggcctgagttatcacccactgtgggagcaaggttacttatctgtcg

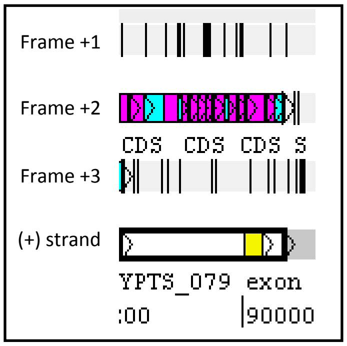

Peptide evidence:

AAHSPAWQEAR
AKQAAALVLVNGQLEHLTAQER
ALEALGAQTWFAGLR
KWEPGMSEEETR
KWEPGMSEEETR
LLPIIDWDNR
LQLNLQVFR
LWEQGVEGIER
QAAALVLVNGQLEHLTAQER
QRPDIPVILTDTGYLFPETYR
QVYQYLTQHGLSYHPLWEQGYLSVGDTHTTR
RALEALGAQTWFAGLR
SQFNLSELNALPK
SQLPVLALQR
YLTQHGLSYHPLWEQGYLSVGDTHTTR
YNTLNKVEPMNR
YNTLNKVEPMNR

Current sequence (peptide evidence in red): 244aa
VSQFNLSELNALPKAKQAAALVLVNGQLEHLTAQERVSWALDNLPGEFVLSSSFGIQAAVCLHLVTRQRPDIPVILTDTGYLFPETYRFIDDLTEKLQLNLQVFRAAHSPAWQEARYGKLWEQGVEGIERYNTLNKVEPMNRALEALGAQTWFAGLRREQSGGRSQLPVLALQRGIFKLLPIIDWDNRQVYQYLTQHGLSYHPLWEQGYLSVGDTHTTRKWEPGMSEEETRFFGLKRECGLHEG

Error 31

Strain: Y. pestis CO92
Gene: YPO3544

Classification: expressed pseudogene
Boundaries of gene: 3952284..3953225

Oligo evidence:

ctaacggaagctgatttacggttatggactacgctagtgcgttttgatccggtgtatgtgacgcatttca

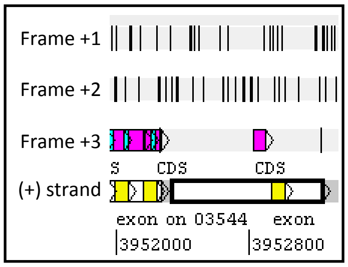

Peptide evidence:

AGFATTQEAYDEAVGTLFSALDR

Proposed sequence (peptide evidence in red): 313aa
MGRWWRYKWITFHPSLTTTDGQAGPQGKGGFNAEAHRYHLYVSLACPWAHRALLMRTLKGLESLISVSVVHPLMQENGWTFSSDFPAATGDALYHLDYLYQLYLRAAPDYSGRVTVPVL
WDKQQQTVVSNESADIIRMFNNAFDDVGAKAGDYYPTALRNDIDDINGWVYDQVNNGVYKAGFATTQEAYDEAVGTLFSALDRLEQILGQHRYLTGNQLTEADLRLWTTLVRFDPVYVT
HFKCDKRRISDYPNLYGFLRDIYQMPGIAETVDFAHIRTHYYRSHGTSILMGLSLSAHSKTCWSHMIGRIALYKN

Strain: Y. pestis pestoides F
Gene: YPDSF_0353

Classification: hypothetical protein
Boundaries of gene: complement (395973..396959)

Oligo evidence:

ctaacggaagctgatttacggttatggactacgctagtgcgttttgatccggtgtatgtgacgcatttca
ctcatcaatggcgtctggaaagacgtttggtacgacacccaatcgaccggtggccatttcaaacgctcaa

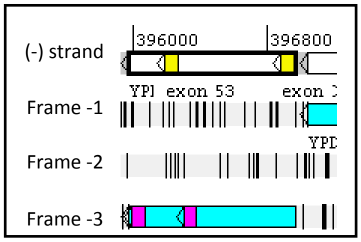

Peptide evidence:

AGFATTQEAYDEAVGTLFSALDR
SHGTINPYGIISIGPQQNLLEPHDR

Current sequence (peptide evidence in red): 328aa
MGQLINGVWKDVWYDTQSTGGHFKRSTAQFRNWVTTDGQAGPQGKGGFNAEAHRYHLYVSLACPWAHRALLMRTLKGLESLISVSVVHPLMQENGWTFSSDFPAATGDALYHLDYLY
QLYLRAAPDYSGRVTVPVLWDKQQQTVVSNESADIIRMFNNAFDDVGAKAGDYYPTALRNDIDDINGWVYDQVNNGVYKAGFATTQEAYDEAVGTLFSALDRLEQILGQHRYLTGNQLTE
ADLRLWTTLVRFDPVYVTHFKCDKRRISDYPNLYGFLRDIYQMPGIAETVDFAHIRTHYYRSHGTINPYGIISIGPQQNLLEPHDRANRFV

Strain: Y. pseudotuberculosis PB1/+
Gene: YPTS_3674

Classification: putative glutathione S-transferase
Boundaries of gene: 4093874..4094860

Oligo evidence:

ctcatcaatggcgtctggaaagacgtttggtacgacacccaatcgaccggtggccatttcaaacgctcaa
ctaacggaagctgatttacggttatggactacgctagtgcgttttgatccggtgtatgtgacgcatttca

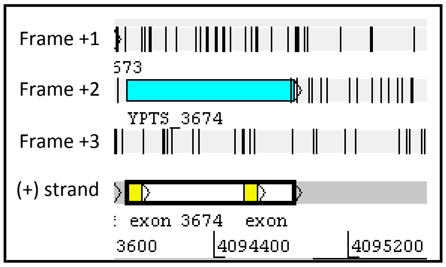

Peptide evidence:

NA

Current sequence (peptide evidence in red): 328aa
MGQLINGVWKDVWYDTQSTGGHFKRSTAQFRNWVTTDGQAGPQGKGGFNAEAHRYHLYVSLACPWAHRALLMRTLKGLESLISVSVVHPLMQENGWTFSSDFPAATGDALYHLDYLY
QLYLRAAPDYSGRVTVPVLWDKQQQTVVSNESADIIRMFNNAFDDVGAKAGDYYPTALRNDIDDINGWVYDQVNNGVYKAGFATTQEAYDEAVGTLFSALDRLEQILGQHRYLTGNQLTE
ADLRLWTTLVRFDPVYVTHFKCDKRRISDYPNLYGFLRDIYQMPGIAETVDFAHIRTHYYRSHGTINPYGIISIGPQQNLLEPHDRANRFV

Error 32

Strain: Y. pestis CO92
Gene: YPO3687

Classification: expressed pseudogene
Boundaries of gene: Frame+1: 4122382..4123473
Frame+3:4123473..4123850

Oligo evidence:

aacatggggccgttgattaatttggctggtctggagaaagtagaagagcacgtaaaagatgcgcttgaga
tcaatgaatgctcggtttcgaccgagttggcccctttcggtggtgttaaagagtctggcctaggtcgtga

Peptide evidence:

EPIGVVAAITPWNFPLAMLTR
FYIQDAIYDEFVNR
KISFTGSTNVGK
PAQNPLQHQR
QHDGIYHIGYFVGGK
VEEHVKDALEK
VEEHVKDALEKGGR
WAVISSSQR
WSQAQEQFDVYNPATGELVAR

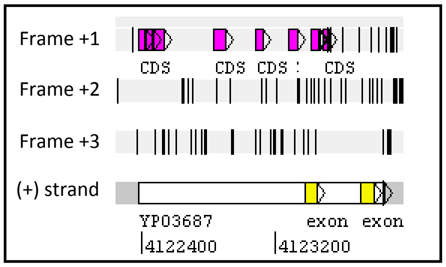

Sequence (peptide evidence in red):
Frame+1: 377aa
MPAQNPLQHQRQHDGIYHIGYFVGGKWSQAQEQFDVYNPATGELVARVAKSGKQETEAAIKAASEAFPAWRKTPAKQRAEILQRWYLLIMEHQQSLAELMVSEQGKPLKEALVEVAYAA
SFIQWFSEQAKRANGEIIPSAKEGARILATREPIGVVAAITPWNFPLAMLTRKLGPALAAGCTGLIKPANNTPLSAFALLALAEQAGVPAGVLNGVVGDTHAISDAIMASSDVRKISFTGSTNV
GKTLMRNAAATMKRISMELGGNAPYIVFDDADLEAAVAGAMACKFRNAGQVCVCVNRFYIQDAIYDEFVNRLAAEVKKLKVGNGMDKDVNMGPLINLAGLEKVEEHVKDALEKGGRLLA
GGIAMRWAVISSSQR

Frame+3: 125aa
HRHALGGNFFQPTVIADANEQMKVASEETFGPLAACFRFSTEAEVIQRANNTPFGLAAYFYTQNLQRVFRVSEALESGMIGVNECSVSTELAPFGGVKESGLGREGSVLGLDEFMEVKTLHL
GNL

Error 102

Classification: YPDSF_0294 annotated as hypothetical protein
error:YPDSF_0295 annotated as succinate-semialdehyde dehydrogenase, upstream start site
Boundaries of gene: YPDSF_0294 Frame+1: 338206..339339
YPDSF_0295 Frame+3: 339336..339674

Strain: Y. pestis pestoides F
Gene: YPDSF_0294 and 0295

Oligo evidence:

aacatggggccgttgattaatttggctggtctggagaaagtagaagagcacgtaaaagatgcgcttgaga
tcaatgaatgctcggtttcgaccgagttggcccctttcggtggtgttaaagagtctggcctaggtcgtga

Peptide evidence:

DVNMGPLINLAGLEK
ISFTGSTNVGK
VEEHVKDALEK
VGNGMDKDVNMGPLINLAGLEK
WSQAQEQFDVYNPATGELVAR
EPIGVVAAITPWNFPLAMLTR
FYIQDAIYDEFVNR
KISFTGSTNVGK
QHDGIYHIGYFVGGK
WAVISSSQR
ANNTPFGLAAYFYTQ
ANNTPFGLAAYFYTQNLQR
FSTEAEVIQR
HALGGNFFQPTVIADANEQMK

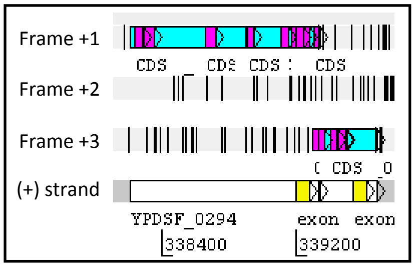

Proposed sequence (peptide evidence in red):
Frame+1: 377aa (YPDSF_0294)
MPAQNPLQHQRQHDGIYHIGYFVGGKWSQAQEQFDVYNPATGELVARVAKSGKQETEAAIKAASEAFPAWRKTPAKQRAEILQRWYLLIMEHQQSLAELMVSEQGKPLKEALVEVAYAA
SFIQWFSEQAKRANGEIIPSAKEGARILATREPIGVVAAITPWNFPLAMLTRKLGPALAAGCTGLIKPANNTPLSAFALLALAEQAGVPAGVLNGVVGDTHAISDAIMASSDVRKISFTGSTNV
GKTLMRNAAATMKRISMELGGNAPYIVFDDADLEAAVAGAMACKFRNAGQVCVCVNRFYIQDAIYDEFVNRLAAEVKKLKVGNGMDKDVNMGPLINLAGLEKVEEHVKDALEKGGRLLA
GGIAMRWAVISSSQR

Frame+3: 125aa (YPDSF_0295)
Current sequence underlined
HRHALGGNFFQPTVIADANEQMKVASEETFGPLAACFRFSTEAEVIQRANNTPFGLAAYFYTQNLQRVFRVSEALESGMIGVNECSVSTELAPFGGVKESGLGREGSVLGLDEFMEVKTLHL
GNL

Strain: Y. pseudotuberculosis PB1/+
Gene: YPTS_3731

Classification: annotated as succinic semialdehyde dehydrogenase
Boundaries of gene: complement (4150763..4152232)

Oligo evidence:

tcaatgaatgctcggtttcgaccgagttggcccctttcggtggtgttaaagagtctggcctaggtcgtga

Peptide evidence:

ANNTPFGLAAYFYTQ
ANNTPFGLAAYFYTQNLQR
EGSVLGLDEFMEVK
EPIGVVAAITPWNFPLAMLTR
FSTEAEVIQR
FYIQDAIYDEFVNR
HALGGNFFQPTVIADANEQMK
ISFTGSTNVGK
QHDGIYHIGYFVGGK
VEEHVKDALEK

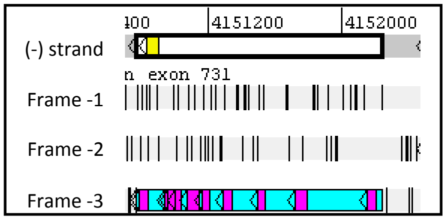

Current sequence (peptide evidence in red): 489aa
MPAQNPLQHQRQHDGIYHIGYFVGGKWSQAQEQFDVCNPATGELVARVAKSGKQETEAAIKAASEAFPAWRKTPAKQRAEILQRWYLLIMEHQQSLAELMVSEQGKPLKEALVEVAYAA
SFIQWFSEQAKRANGEIIPSAKEGARILATREPIGVVAAITPWNFPLAMLTRKLGPALAAGCTGLIKPANNTPLSAFALLALAEQAGVPAGVLNGVVGDTHAISDAIMASSDVRKISFTGSTNV
GKTLMRNAAATMKRISMELGGNAPYIVFDDADLEAAVAGAMACKFRNAGQVCVCVNRFYIQDAIYDEFVNRLAAEVKKLKVGNGMDKDVNMGPLINLAGLEKVEEHVKDALEKGGRLLA
GGHRHALGGNFFQPTVIADANEQMKVASEETFGPLAACFRFSTEAEVIQRANNTPFGLAAYFYTQNLQRVSRVSEALESGMIGVNECSVSTELAPFGGVKESGLGREGSVLGLDEFMEVKTL
HLGNL

Error 33

Strain: Y. pestis CO92
Gene: YPO3723

Classification: expressed pseudogene (iclR)
Boundaries of gene: Frame+1: 4167025:4167291
Frame+3:4167294:4167869

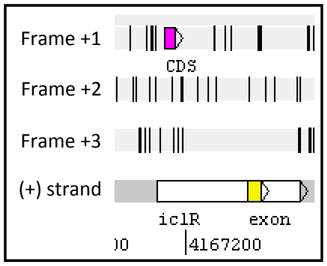

Oligo evidence:

acataaaaaggggttgcacgcttacacccagcacacccgtaccaatccaaccagcctgaaagaaaatcta

Peptide evidence:

AATQNTPAATGQVQSLTR

Proposed sequence (peptide evidence in red):
Frame+1: 89aa
MALPIPIKRGKKPKAATQNTPAATGQVQSLTRGLKLLEYISESDGNVALTDLAQQAGLPNSTTHRLLTTMQQQGFVRQVGDLGLWTMGA

Frame+3: 191aa
HAFIVGSSFLQSRNLLAMVHPMLRRLMDESGETVNLAVLDHSDYQAIIIDQVQCTALMRMSAPIGGKLPMHASGAGKAFLSTLPDDQLVQLLHKKGLHAYTQHTRTNPTSLKENLALIRKQG
YSFDDEEHALGLRCIAACLFDEHHEAFAAISISGPISRITDDRVTELGALVIHAAKEITQSYGGGTGVK

Strain: Y. pestis pestoides F
Gene: YPDSF_0176

Classification: transcriptional repressor IclR
Boundaries of gene: complement (185391..186233)

Oligo evidence:

acataaaaaggggttgcacgcttacacccagcacacccgtaccaatccaaccagcctgaaagaaaatcta

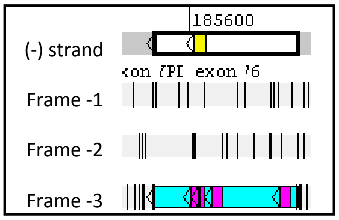

Peptide evidence:

AATQNTPAATGQVQSLTR
GLHAYTQHTR
MSAPIGGKLPMHASGAGK
TNPTSLKENLALIR

Current sequence (peptide evidence in red): 280aa
MALPIPIKRGKKPKAATQNTPAATGQVQSLTRGLKLLEYISESDGNVALTDLAQQAGLPNSTTHRLLTTMQQQGFVRQVGDLGLWTMGAHAFIVGSSFLQSRNLLAMVHPMLRRLMDESG
ETVNLAVLDHSDYQAIIIDQVQCTALMRMSAPIGGKLPMHASGAGKAFLSTLPDDQLVQLLHKKGLHAYTQHTRTNPTSLKENLALIRKQGYSFDDEEHALGLRCIAACLFDEHHEAFAAISIS
GPISRITDDRVTELGALVIHAAKEITQSYGGGTGVK

Strain: Y. pseudotuberculosis PB1/+
Gene: YPTS_3840

Classification: transcriptional repressor IclR
Boundaries of gene: 4290734..4291576

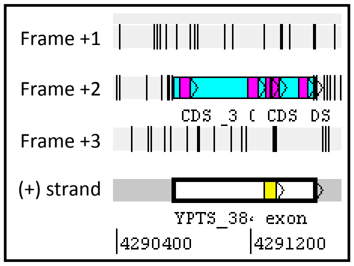

Oligo evidence:

acataaaaaggggttgcacgcttacacccagcacacccgtaccaatccaaccagcctgaaagaaaatcta

Peptide evidence:

AATQNTPAATGQVQSLTR
GLHAYTQHTR
ITDDRVTELGALVIHAAK
MSAPIGGKLPMHASGAGK
TNPTSLKENLALIR

Current sequence (peptide evidence in red): 280aa
MALPIPIKRGKKPKAATQNTPAATGQVQSLTRGLKLLEYISESDGNVALTDLAQQAGLPNSTTHRLLTTMQQQGFVRQVGDLGLWTMGAHAFIVGSSFLQSRNLLAMVHPMLRRLMDESG
ETVNLAVLDHSDYQAIIIDQVQCTALMRMSAPIGGKLPMHASGAGKAFLSTLPDDQLVQLLHKKGLHAYTQHTRTNPTSLKENLALIRKQGYSFDDEEHALGLRCIAACLFDEHHEAFAAISIS
GPISRITDDRVTELGALVIHAAKEITQSYGGGTGVK

Error 34

Strain: Y. pestis CO92
Gene: YPO3775

Classification: expressed pseudogene with 2 insertions
Boundaries of gene: Frame-2, region 1: complement (4238744..4239223)
Frame-2, region 2: complement (4236482..4236784)

Oligo evidence:

aattagctctggctgccgaactttcgctgccggtatttttgcattgtcgtgatgcccatgagcgttttat
tattcctgttcagcagctattgttggaaactgatgctccctatttattgccaagggatttgaatcctaaa

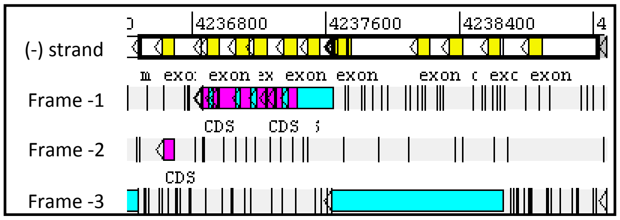

Peptide evidence:

IPVQQLLLETDAPYLLPR

Proposed sequence (peptide evidence in red):

Region1: 162aa
MFDIGVNLTSVQFAKDYHQVVNRAKEAGVLGILITGTDADESLAAQTLAAEYPGYCWSTTGVHPHHASSWQDSVEQKIRTLAATASVVGIGECGLDFNRNFSTPAQQEVAFTAQLALAAELS
LPVFLHCRDAHERFIDLLVPWLDKIPAAVVHCFTGNSD

Region2: 100aa
ELDACLALGLSIGITGWVCDERRGLDLRALLPRIPVQQLLLETDAPYLLPRDLNPKPASRRNEPCFLPHIVQQVAAWRQEDPNWLGQKTDENARRVFRLV

Strain: Y. pestis pestoides F
Gene: YPDSF_3392

Classification: DNase TatD
Boundaries of gene: complement (3894442..3895224)

Oligo evidence:

tattcctgttcagcagctattgttggaaactgatgctccctatttattgccaagggatttgaatcctaaa
aattagctctggctgccgaactttcgctgccggtatttttgcattgtcgtgatgcccatgagcgttttat

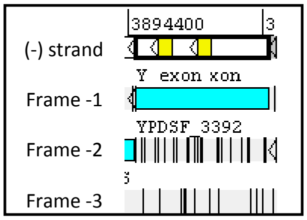

Peptide evidence:

NA

Current sequence (peptide evidence in red): 260aa
MFDIGVNLTSVQFAKDYHQVVNRAKEAGVLGILITGTDADESLAAQTLAAEYPGYCWSTTGVHPHHASSWQDSVEQKIRTLAATASVVGIGECGLDFNRNFSTPAQQEVAFTAQLALAAELS
LPVFLHCRDAHERFIDLLVPWLDKIPAAVVHCFTGNSDELDACLALGLSIGITGWVCDERRGLDLRALLPRIPVQQLLLETDAPYLLPRDLNPKPASRRNEPCFLPHIVQQVAAWRQEDPNWL
GQKTDENARRVFRLV

Strain: Y. pseudotuberculosis PB1/+
Gene: YPTS_0277

Classification: DNase TatD
Boundaries of gene: 301522..302304

Oligo evidence:

aattagctctggctgccgaactttcgctgccggtatttttgcattgtcgtgatgcccatgagcgttttat
tattcctgttcagcagctattgttggaaactgatgctccctatttattgccaagggatttgaatcctaaa

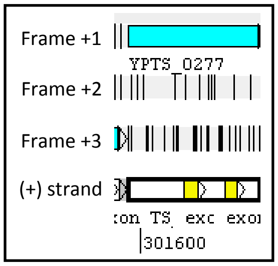

Peptide evidence:

NA

Current sequence (peptide evidence in red): 260aa
MFDIGVNLTSVQFAKDYHQVVNRAKEAGVLGILITGTDADESLAAQTLAAEYPGYCWSTTGVHPHHASSWQDSVEQKIRTLAATASVVAIGECGLDFNRNFSTPAQQEVAFTAQLALAAELS
LPVFLHCRDAHERFIDLLVPWLDKIPAAVVHCFTGNSGELDACLALGLSIGITGWVCDERRGLDLRALLPRIPVQQLLLETDAPYLLPRDLNPKPASRRNEPCFLPHIVQQVAAWRQEDPNWL
GQKTDENARRVFRLV

Error 35

Strain: Y. pestis CO92
Gene: YPO3870

Classification: expressed pseudogene
Boundaries of gene: Frame+1: 4345237..4345503
Frame+3: 4345506..4345661
Frame2+: 4345661..4346731

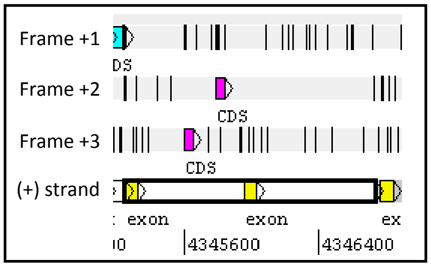

Oligo evidence:

taagttccacctcactttatgctgccatcgatcttggctccaatagttttcatatgttggtagtacgtga
tcaaacaaagagcgattcagtgtggcaaattagaagagttggaaatccctggtttaaccttggaacgagc

Peptide evidence:

IYHGVAHTTGGPEQR
LIYHGVAHTTGGPEQR
AELAAHEMIKPVAQR

Proposed sequence (peptide evidence in red):
Frame+1: 90aa
MMLSSTSLYAAIDLGSNSFHMLVVREVAGSIQTLARIKRKVRLAAGLDNQNHLSQEAMERGWQCLKLFSERLQDIPLDQIRVVATATLR

Frame+3: 52aa
ASNADEFLRTATEILGCPIQVISGEEEARLIYHGVAHTTGGPEQRLVVDIGG

Frame+2: 356aa
GSTELVTGNGAQANILVSLSMGCVTWLERYFGDRHLAKENFERAELAAHEMIKPVAQRFREHGWQVCVGASGTVQALQEIMVAQGMDELITLAKLQQLKQRAIQCGKLEELEIPGLTLERA
LVFPSGLSILIAIFQELSIESMTLAGGALREGLVYGMLHLPVEQDIRRRTLRNLQRRYLLDTEQAKRVSCLADNFFLQVEKEWHLDGRCREFLQNACLIHEIGLSVDFKHAPQHAAYLIRNLDLPG
FTPAQKLLLSALLQNQSDTIDLSLLNQQNALPADMAQHLCRLLRLAIIFSSRRRDDTLPAVRLRADNNALYVLVPQGWLEQHPYRAEALEQESHWQSYVQWPLLLEELS

Strain: Y. pestis pestoides F
Gene: YPDSF_3488

Classification: guanosine pentaphosphate phosphohydrolase
Boundaries of gene: 4001302..4002798

Oligo evidence:

taagttccacctcactttatgctgccatcgatcttggctccaatagttttcatatgttggtagtacgtga
tcaaacaaagagcgattcagtgtggcaaattagaagagttggaaatccctggtttaaccttggaacgagc

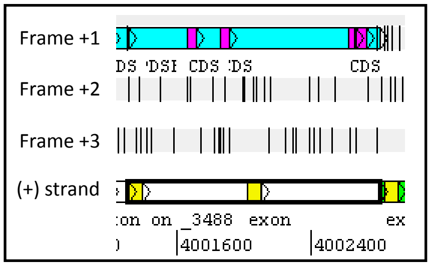

Peptide evidence:

AELAAHEMIKPVAQR
LIYHGVAHTTGGPEQR
LRADNNALYVLVPQGWLEQHPYR
RRDDTLPAVR

Current sequence (peptide evidence in red): 498aa
MMLSSTSLYAAIDLGSNSFHMLVVREVAGSIQTLARIKRKVRLAAGLDNQNHLSQEAMERGWQCLKLFSERLQDIPLDQIRVVATATLRLASNADEFLRTATEILGCPIQVISGEEEARLIYHGV
AHTTGGPEQRLVVDIGGGSTELVTGNGAQANILVSLSMGCVTWLERYFGDRHLAKENFERAELAAHEMIKPVAQRFREHGWQVCVGASGTVQALQEIMVAQGMDELITLAKLQQLKQRA
IQCGKLEELEIPGLTLERALVFPSGLSILIAIFQELSIESMTLAGGALREGLVYGMLHLPVEQDIRRRTLRNLQRRYLLDTEQAKRVSCLADNFFLQVEKEWHLDGRCREFLQNACLIHEIGLSVDFK
HAPQHAAYLIRNLDLPGFTPAQKLLLSALLQNQSDTIDLSLLNQQNALPADMAQHLCRLLRLAIIFSSRRRDDTLPAVRLRADNNALYVLVPQGWLEQHPYRAEALEQESHWQSYVQWPLLL
EELS

Strain: Y. pseudotuberculosis PB1/+
Gene: YPTS_0176

Classification: guanosine pentaphosphate phosphohydrolase
Boundaries of gene: complement (190347..191843)

Oligo evidence:

taagttccacctcactttatgctgccatcgatcttggctccaatagttttcatatgttggtagtacgtga
tcaaacaaagagcgattcagtgtggcaaattagaagagttggaaatccctggtttaaccttggaacgagc

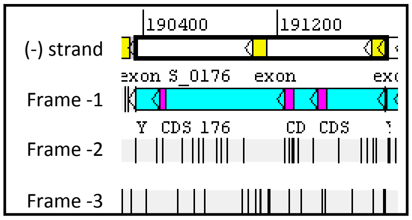

Peptide evidence:

AELAAHEMIKPVAQR
LIYHGVAHTTGGPEQR
RRDDTLPAVR

Current sequence (peptide evidence in red): 498aa
MMLSSTSLYAAIDLGSNSFHMLVVREVAGSIQTLARIKRKVRLAAGLDNQNHLSQEAMERGWQCLKLFSERLQDIPLDQIRVVATATLRLASNADEFLRTATEILGCPIQVISGEEEARLIYHGV
AHTTGGPEQRLVVDIGGGSTELVTGNGAQANILVSLSMGCVTWLERYFGDRHLAKENFERAELAAHEMIKPVAQRFREHGWQVCVGASGTVQALQEIMVAQGMDELITLAKLQQLKQRA
IQCGKLEELEIPGLTLERALVFPSGLSILIAIFQELSIESMTLAGGALREGLVYGMLHLPVEQDIRRRTLRNLQRRYLLDTEQAKRVSCLADNFFLQVEKEWHLDGRCREFLQNACLIHEIGLSVDFK
HAPQHAAYLIRNLDLPGFTPAQKLLLSALLQNQSDTIDLSLLNQQNALPADMAQHLCRLLRLAIIFSSRRRDDTLPAVRLRADNNALYVLVPQGWLEQHPYRAEALEQESHWQSYVQWPLLL
EELS

Error 36

Strain: Y. pestis CO92
Gene: YPO4052

Classification: expressed pseudogene
Boundaries of gene: Frame-3: complement (4571292..4571510)
Frame-2: complement (4569557..4571290)

Oligo evidence:

cgtgatcgactcggtattggtcgcaaactggcgatccaaattcttgaattctttgaccgcagtggtttca
actgctaggtcaccagcagttaattgacgaccaacaaccgttaattgaccatcaacaaatcataaataat

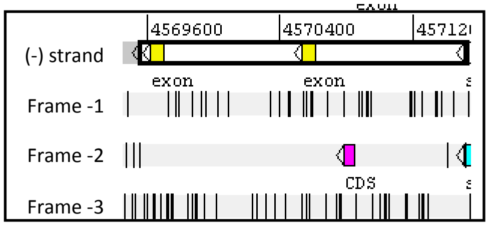

Peptide evidence:

GIHAQNQPAQQAQAGQR

Proposed sequence (peptide evidence in red):
Frame-3: 74aa
MIIVTAGHVDHGKTTLLQAITGVNADRLPEEKQRGMTIDLGYAYWPLPDGRIMGFIDVPGHEKFLANMLAGGG

Frame-2: 578aa
GIDHALLVVACDDGVMAQTREHLAILRLSGRPALTVALTKADRVDDERIAQVHQQILQELVAQGWSAEQISLFVTAAVTERGIGELREHLAQCHQQRDPKGRLQRRFRLAIDRVFSVKGAGL
VVTGTALAGQVAVGDTLWLTGGDCPVRVRGIHAQNQPAQQAQAGQRIALNISGDISKQQINRGDWLLAQPPLPPVDRVLVTVDADTPLQHWQPLHLYHGARHITGRFSLLGHQQLIDD
QQPLIDHQQIINNQSTSGGHSSQILAELLLDSPLWLVENDRVILRDIGAKKTLGGARVIHLAVPSRGKRQPAYLAWLTALAQATSDREVLDLHLAQGPVSVRTFSWARQLTDNEMAALLAAT
DTVVAGDIALSHPHAQQAQQTLLHVLALYHQQHGDQLGLGRARLRRMALPTLDEGVVFRLIDNLLAEGALHNTRGWLHLPEHGLAFTEQEQIGWQQAAPYFVDDPWWVRDLAAELQA
AEGEMRSLLRKAAQLGYITAVVTDRYYLSQRIEQLADLVRELDSRQGSVSAADFRDRLGIGRKLAIQILEFFDRSGFTRRRGNGHILRDSGLFSATGS

Strain: Y. pestis pestoides F
Gene: YPDSF_0040

Classification: selenocysteinyl-tRNA-specific translation factor
Boundaries of gene: 48255..50207

Oligo evidence:

actgctaggtcaccagcagttaattgacgaccaacaaccgttaattgaccatcaacaaatcataaataat
cgtgatcgactcggtattggtcgcaaactggcgatccaaattcttgaattctttgaccgcagtggtttca

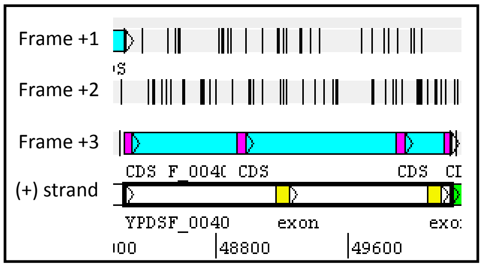

Peptide evidence:

MIIVTAGHVDHGK
DLAAELQAAEGEMR
DSGLFSATGS
GIHAQNQPAQQAQAGQR

Current sequence (peptide evidence in red): 650aa
MIIVTAGHVDHGKTTLLQAITGVNADRLPEEKQRGMTIDLGYAYWPLPDGRIMGFIDVPGHEKFLANMLAGGGGIDHALLVVACDDGVMAQTREHLAILRLSGRPALTVALTKADRVDDE
RIAQVHQQILQELVAQGWSAEQISLFVTAAVTERGIGELREHLAQCHQQRDPKGRLQRRFRLAIDRVFSVKGAGLVVTGTALAGQVAVGDTLWLTGGDCPVRVRGIHAQNQPAQQAQAG
QRIALNISGDISKQQINRGDWLLAQPPLPPVDRVLVTVDADTPLQHWQPLHLYHGARHITGRFSLLGHQQLIDDQQPLIDHQQIINNQSTSGGHSSQILAELLLDSPLWLVENDRVILRDIGAK
KTLGGARVIHLAVPSRGKRQPAYLAWLTALAQATSDREVLDLHLAQGPVSVRTFSWARQLTDNEMAALLAATDTVVAGDIALSHPHAQQAQQTLLHVLALYHQQHGDQLGLGRARLRRM
ALPTLDEGVVFRLIDNLLAEGALHNTRGWLHLPEHGLAFTEQEQIGWQQAAPYFVDDPWWVRDLAAELQAAEGEMRSLLRKAAQLGYITAVVTDRYYLSQRIEQLADLVRELDSRQGSVSA
ADFRDRLGIGRKLAIQILEFFDRSGFTRRRGNGHILRDSGLFSATGS

Strain: Y. pseudotuberculosis PB1/+
Gene: YPTS_4155

Classification: selenocysteinyl-tRNA-specific translation factor
Boundaries of gene: 4640810..4642783

Oligo evidence:

acattgcgctatcacattctcacgcacagcaagcgcaacagaccctcttacatgtactcgccctctatca
cgtgatcgactcggtattggtcgcaaactggcgatccaaattcttgaattctttgaccgcagtggtttca

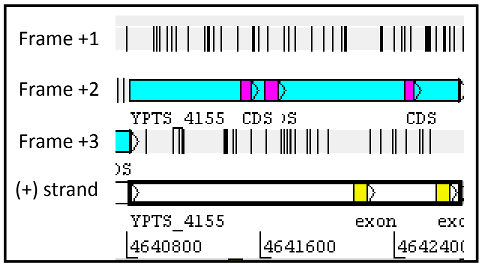

Peptide evidence:

DLAAELQAAEGEMR
GIHAQNQPAQQAQAGQR
VLVTVDADTPLQHWQPLHLYHGAR

Current sequence (peptide evidence in red): 657aa
MIIVTAGHVDHGKTTLLQAITGVNADRLPEEKQRGMTIDLGYAYWPLPDGRIMGFIDVPGHEKFLANMLAGVGGIDHALLVVACDDGVMAQTREHLAILRLSGRPALTVALTKADRVDDER
IAQVHQQILQELVAQGWSAEQISLFVTAAVTERGIGELREHLAQCHQQRDPKGRLQRRFRLAIDRVFSVKGAGLVVTGTALAGQVAVGDTLWLTGGDCPVRVRGIHAQNQPAQQAQAGQ
RIALNISGDISKQQINRGDWLLAQPPLPPVDRVLVTVDADTPLQHWQPLHLYHGARHITGRFSLLGHQQLIDDQQPLIDDQQPLIGHQQIINNQSTSGGHSSQILAELLLDSPLWLVENDRVIL
RDIGAKKTLGGARVIHLAVPSRGKRQPAYLAWLTALAQATSDREVLDLHLAQGPVSVRTFSWARQLTDNEMAALLAATDTVVAGDIALSHSHAQQAQQTLLHVLALYHQQHGDQLGLGR
ARLRRMALPTLDEGVVFRLIDNLLAEGALHNTRGWLHLPEHGLAFTEQEQIGWQQAAPYFVDDPWWVRDLAAELQAAEGEMRSLLRKAAQLGYITAVVTDRYYLSQRIEQLADLVRELDSS
QGSVSAADFRDRLGIGRKLAIQILEFFDRSGFTRRRGNGHILRDSGLFSATGS

Error 37

Strain: Y. pestis CO92
Gene: YPO2267

Classification: expressed pseudogene
Boundaries of gene: Frame+1: 2549308.. 2549508
Frame+3: 2549517.. 2550239

Oligo evidence:

gaagaacagattggggcccgcttattggcacgaaggcacgaaataagcgtaatatcagtctcacccaagc

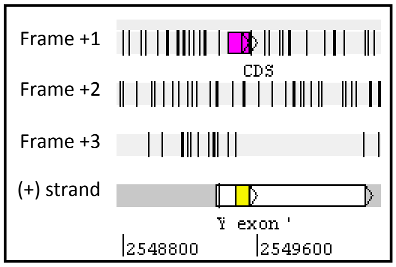

Peptide evidence:

LHISQPPLSQQIQILEEQIGAR
RHEISVISVSPK

Proposed sequence (peptide evidence in red): current sequence underlined
Frame+1: 84aa
LSNILIIANNTFKISMSIELRHLRYFIAVAEELHFGRAAERLHISQPPLSQQIQILEEQIGARLLARRHEISVISVSPKRESSF

Frame+3: 240aa
NKRNISLTQAGKLFLKESYQILAQVNSATEKAACLHRGESGVLTIGFTSSAPLINVVSKNLRTFRQIYPQVHIKMQEVSTKQQIEALLDGRLDLGIMRNTRLPEALQYRLLLREPLVAVVHEDHPL
ASLPNGSVKFSSLDKQPFVFFSREVGTALYDEFFTLLTRAGITPYITQEVGEAMTIIGLVSSGLGVSILPASFRRVNVDGVKYLSLDEADATTEMWLVNHKHRPMPPLQKHCSV

Strain: Y. pestis pestoides F
Gene: YPDSF_0866

Classification: annotated as transcriptional regulator LYSR-type
Boundaries of gene: complement(986074..986952)

Oligo evidence:

ttattggcacgaaataagcgtaatatcagtctcacccaagcgggaaagctctttttaaaagagtcctacc

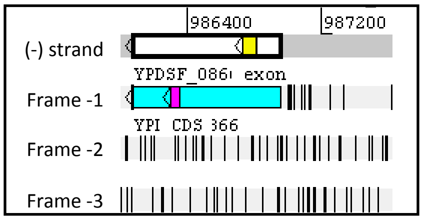

Peptide evidence:

EVGTALYDEFFTLLTR

Current sequence (peptide evidence in red): 292aa
MSIELRHLRYFIAVAEELHFGRAAERLHISQPPLSQQIQILEEQIGARLLARNKRNISLTQAGKLFLKESYQILAQVNSATEKAACLHRGESGVLTIGFTSSAPLINVVSKNLRTFRQIYPQVHIKMQ
EVSTKQQIEALLDGRLDLGIMRNTRLPEALQYRLLLREPLVAVVHEDHPLASLPNGSVKFSSLDKQPFVFFSREVGTALYDEFFTLLTRAGITPYITQEVGEAMTIIGLVSSGLGVSILPASFRRVNV
DGVKYLSLDEADATTEMWLVNHKHRPMPPLQKHCSV

Strain: Y. pseudotuberculosis PB1/+
Gene: no ortholog

Error 38

Strain: Y. pestis CO92
Gene: YPO3436

Classification: expressed pseudogene
Boundaries of gene: complement (3838850..3839971)

Oligo evidence:

tgatgttgaacaagactggaccgctttttgtcccactgattctgtaaatgaattatatcaggctacagct

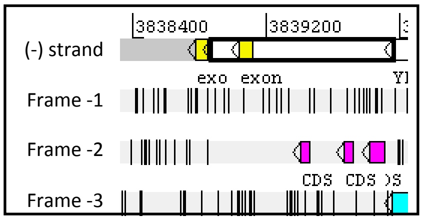

Peptide evidence:

EKYQGFNVEFPTGR
ELGQVAHVPQVALTELVANAWDAGASR
KLPDADEILTVLSAR
LPDADEILTVLSAR

Proposed sequence (peptide evidence in red): 373aa
MRPPMSEQPLQNSLFEDDYLLRELGQVAHVPQVALTELVANAWDAGASRVDLVLPTDIGGILTVTDDGHGMTPEQFKKRWMTLRYTREKYQGFNVEFPTGRTARPRKAYGRNGIGRHGL
LCFADEYEVKTWRDGILATFVVGTGAGSSPFVCRSETEEKHIGSGTRLQVQVTRKLPDADEILTVLSARFIHDPEFEVRVNGKLRPFTEIDGRISEETLCLSAGRHAKVIVIDSTRLNHSSLHQGIAF
WVQHRLVGTPSWAVGQIANFDGRTRFARRYKVIVDTEGFESDVEQDWTAFCPTDSVNELYQATAEHIRDVAQRLAVEFTEITSEDALMQNRSELATLGQGARLGVAEFTTAVAQEHPTVS
PDVNDG

Strain: Y. pestis pestoides F
Gene: YPDSF_3246

Classification: hypothetical protein
Boundaries of gene: complement (3697940..3699049)

Oligo evidence:

tgatgttgaacaagactggaccgctttttgtcccactgattctgtaaatgaattatatcaggctacagct

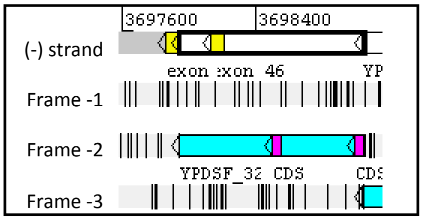

Peptide evidence:

PLQNSLFEDDYLLR
KLPDADEILTVLSAR

Current sequence (peptide evidence in red): 369aa
MSEQPLQNSLFEDDYLLRELGQVAHVPQVALTELVANAWDAGASRVDLVLPTDIGGILTVTDDGHGMTPEQFKKRWMTLRYTREKYQGFNVEFPTGRTARPRKAYGRNGIGRHGLLCFAD
EYEVKTWRDGILATFVVGTGAGSSPFVCRSETEEKHIGSGTRLQVQVTRKLPDADEILTVLSARFIHDPEFEVRVNGKLRPFTEIDGRISEETLCLSAGRHAKVIVIDSTRLNHSSLHQGIAFWVQ
HRLVGTPSWAVGQIANFDGRTRFARRYKVIVDTEGFESDVEQDWTAFCPTDSVNELYQATAEHIRDVAQRLAVEFTEITSEDALMQNRSELATLGQGARLGVAEFTTAVAQEHPTVSPDVN
DG

Strain: Y. pseudotuberculosis PB1/+
Gene: no ortholog

Error 39

Strain: Y. pestis CO92
Gene: YPO3679

Classification: expressed pseudogene (tcaB)
Boundaries of gene: Frame -3: complement (4112526..4113893)
Frame-1: complement (4110301..4112526)

Oligo evidence:

ctcatagataaatttgaaattagaatatttccatataatgaattggtagccagcactattgtattcaata

Peptide evidence:

NISTHFSSDNFLHGWNSYNKR
HNLNLDGQPLHLPLFATPVDPK
IASAALNGESLVPLGLSAVLDTAPNVFGLADGGSR
IHYIQASQLLGPRPNLNSSHQWENIK
LLLQSLSDIILHIR
QDNEAMTLLFQQQQQK

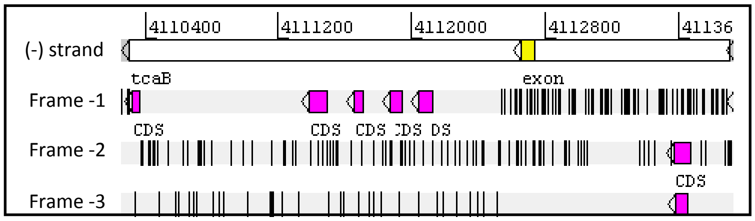

Proposed sequence (peptide evidence in red):
Frame-3: 458aa
MPTSTVLNKINESRRDALVNYYLAKNVSGDEKIKTAEQLYQYLLLDTKIGHEVKTSPIAEAISSLQIYINRCVDGEENDLHEKNISTHFSSDNFLHGWNSYNKRYARWAGKEKLMYYAADYIDPT
LRYNKTELFNTFEQSINNSRLTEKSVKSALQSYLISYEKLAQIDTIKELYVENIKTHFFLGKTRESPCQYYWRSGEQLSNDSHHLRWSEWKKVNCNINGTEEKFFINLSWHRSSLYIDWINKFSIRT
DKDETTEKYHYNRVYKNDDNTWSELIINMDIGFKLSTSTEVITLPPFFVNQGDPNAKEEVENLFLTNGTSIKRIESRLHGYINGHIKILCNDDGISFEIAEKLINGDNGTIGEVKIIENEKIISTINNEK
GENKKINLIDKFEIRIFPYNELVASTIVFNRIKPEEKIIDELDDKISLLSPNSLPLKEKFQTSVDELLSYNTQK

Frame-1: 741aa
NNVKLNAFNGSYGIYLWELFFHIPLLASMRFLNEQRFDLAQHWLKYLFNSAGYRDGNGNLLKEGDNILYWNILPLQQDTAWDKNTLIQATDDPDVIAMQDPMQYKLAIFMRTLDLIISQGD
QAYRQLERDTLAEAKIHYIQASQLLGPRPNLNSSHQWENIKLAEESRQLENSHFLPPYNELLLSYWDKLEIRLYNLRHNLNLDGQPLHLPLFATPVDPKALQRQHGAGNGINSSEQIATAQTSL
YRFPLLIERAKSAVSAVIQFGNSLQSVLERQDNEAMTLLFQQQQQKVLQHTKDIQNNNIQVLQASLEATDSLKSAAEQRRKHYKELLDNGISSDEQLAINIRIASAALNGESLVPLGLSAVLDTA
PNVFGLADGGSRWGAISQAVGWGMQSMAMALETTAGVRDAKANYSRRAQEWTLQKDQADKDIEQLAHQYTSVQEQLNMAQKQLNLAELEQGHADALYQMQSTRFTGKELYNWM
AGRLSGLYFQLFDATQPLCLMAKAALEKEVDKAKTDGLFIRSGWNDLYQGLLAGEDLQLNLQKLENVWLMEEQRALEVERTVSLAQHYQQLSDHKFNLAEIVTGYMAQDKDQKTGNEQDF
VELKNGTLITSLSIKGLNLVEDYPETMHLGDIRRIKQISVSLPALLGPYQDVQATLDYAGENTHLAKGCTALAISRGMNDSGQFLLDFNDGKYLPFEGIDISDKGTLVLRFPNATSKQKLLLQSLSD
IILHIRYTIRS

Strain: Y. pestis pestoides F
Gene: YPDSF_0286

Classification: annotated as toxin subunit
Boundaries of gene: 326124..329717

Oligo evidence:

ctcatagataaatttgaaattagaatatttccatataatgaattggtagccagcactattgtattcaata
agcctgccattaaaagaaaaatttcaaacatcagttgatgagttactcagttataacacccaaaaaaata

Peptide evidence:

IHYIQASQLLGPRPNLNSSHQWENIK
LLLQSLSDIILHIR
SAVSAVIQFGNSLQSVLER

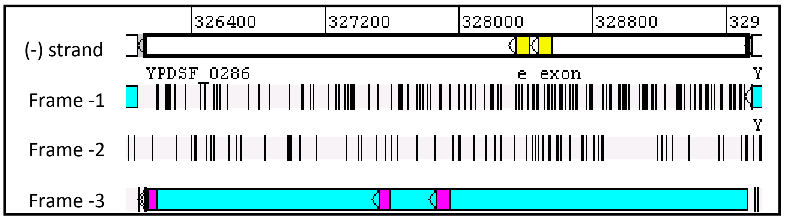

Current sequence (peptide evidence in red): 1197aa
MPTSTVLNKINESRRDALVNYYLAKNVSGDEKIKTAEQLYQYLLLDTKIGHEVKTSPIAEAISSLQIYINRCVDGEENDLHEKNISTHFSSDNFLHGWNSYNKRYARWAGKEKLMYYAADYIDPT
LRYNKTELFNTFEQSINNSRLTEKSVKSALQSYLISYEKLAQIDTIKELYVENIKTHFFLGKTRESPCQYYWRSGEQLSNDSHHLRWSEWKKVNCNINGTEEKFFINLSWHRSSLYIDWINKFSIRT
DKDETTEKYHYNRVYKNDDNTWSELIINMDIGFKLSTSTEVITLPPFFVNQGDPNAKEEVENLFLTNGTSIKRIESRLHGYINGHIKILCNDDGISFEIAEKLINGDNGTIGEVKIIENEKIISTINNEK
GENKKINLIDKFEIRIFPYNELVASTIVFNRIKPEEKIIDELDDKISLLSPNSLPLKEKFQTSVDELLSYNTQKNNVKLNAFNGSYGIYLWELFFHIPLLASMRFLNEQRFDLAQHWLKYLFNSAGYRD
GNGNLLKEGDNILYWNILPLQQDTAWDKNTLIQATDDPDVIAMQDPMQYKLAIFMRTLDLIISQGDQAYRQLERDTLAEAKIHYIQASQLLGPRPNLNSSHQWENIKLAEESRQLENSHFLP
PYNELLLSYWDKLEIRLYNLRHNLNLDGQPLHLPLFATPVDPKALQRQHGAGNGINSSEQIATAQTSLYRFPLLIERAKSAVSAVIQFGNSLQSVLERQDNEAMTLLFQQQQQKVLQHTKDIQ
NNNIQVLQASLEATDSLKSAAEQRRKHYKELLDNGISSDEQLAINIRIASAALNGESLVPLGLSAVLDTAPNVFGLADGGSRWGAISQAVGWGMQSMAMALETTAGVRDAKANYSRRAQE
WTLQKDQADKDIEQLAHQYTSVQEQLNMAQKQLNLAELEQGHADALYQMQSTRFTGKELYNWMAGRLSGLYFQLFDATQPLCLMAKAALEKEVDKAKTDGLFIRSGWNDLYQGLLAGE
DLQLNLQKLENVWLMEEQRALEVERTVSLAQHYQQLSDHKFNLAEIVTGYMAQDKDQKTGNEQDFVELKNGTLITSLSIKGLNLVEDYPETMHLGDIRRIKQISVSLPALLGPYQDVQATLDY
AGENTHLAKGCTALAISRGMNDSGQFLLDFNDGKYLPFEGIDISDKGTLVLRFPNATSKQKLLLQSLSDIILHIRYTIRS

Strain: Y. pseudotuberculosis PB1/+
Gene: no ortholog

Error 40

Strain: Y. pestis CO92
Gene: YPMT1.89

Classification: expressed pseudo gene annotated as similar to DNA polymerase III subunit alpha
Boundaries of gene: 93170..96208

Oligo evidence:

gcgtcttactaccaaagagtttggctacacaccaccggcttctgagcaccgcgtgtatgttgatcgtctg

Peptide evidence:

DQAELMGNLVIDAVK

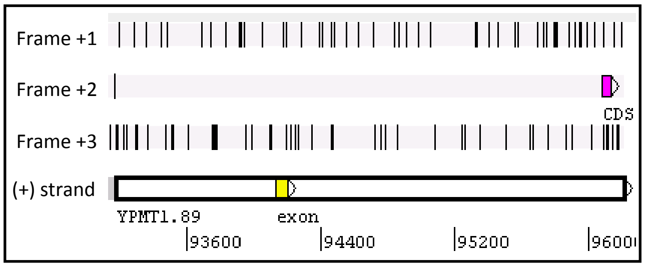

Proposed sequence (peptide evidence in red): 1013aa
MKALMVRTDFSLGESALKAENAVKIARDAGYTAVISADSMNIASVIPLQRAAGDDMAVICGVKLNVVDDPTYEHRARLAKESERCMESLVRDRSYCFTALIKNEQGYRDVCELMTLANKREQ
FYFVPRLALDQLAAAYAKGNIILLTSDIGSVFQRRDFAKIIGTLVTAGGRDNFYSVVYPHPTPFYDQINVRAMKVASALKIEPVAFYPAYYEAVDDADIKDIAHMVTNNIKIDQPHRLRIPHQRD
NAVNGRRHLLEALKAFSVRMDVPVTAAMASTTQDTIIEACTWRWHELPPALPKMADDEPATLMKLAVAGLRKRLTTKEFGYTPPASEHRVYVDRLKYEMDTLTRLGFCGYFLMVRDLMN
HSRETGIPVGPGRGSSAGSLVAWCIGITNVDPIRHGLLFERFINPERLDLPDADLDFSQARRHEVIEYLNERYGEDYVAGIPNFTYLGAASALRDTARIYGVDAADMAVSKEFKNLEDDSLSLEEL
REQLASLDKYATKYPEAFKAACKLQSLMRGFGRHAAGMIVAGVPLVERTPVELRGNARCIAFDKRYCEAMGLIKLDVLGLATLDLLDSAKRYIKESTGEDINLDAIPLDDRKVLDGFAAGYTQG
VFQLESGPMRKLLKDLGGGIEPMSFKTVVATTALFRPGPIQSGMLDDYVSVAKGFMAPQSLHPVLDELTAETNGVILYQEQTMNATRLLAGFTMAEADGVRKAIGKKDMEKMKSMGEKFV
VQAQAGWIDVEMEDGTTQRIHRAEHFKCEDGALRTVEEALEAGVKLPMAAVRVTGSQPGLSETKAKEIWDAFEKNGAYQFNKSHPVAYSLISYQSMWLKTHYPAEFFAAALTILGEDKHQ
GLVKDALTYGIHVLPPDVNVSSNRIEIRTLEDGSQVLYAPFSAVKGCSENGCQAIMRAREKVGGKFESLEQFEEAVEKRACACNSRVRESLQKVGAFASIEPGSLPATDPERLRDQAELMGNLV
IDAVKASRPFEMNPKRSAEVNVLMTRMAAEMGL

Strain: Y. pestis pestoides F plasmid
Gene: YPDSF_4091

Classification: annotated as DNA polymerase III
Boundaries of gene: complement (62215..65739)

Oligo evidence:

gcgtcttactaccaaagagtttggctacacaccaccggcttctgagcaccgcgtgtatgttgatcgtctg
Tactgatgacacgcatggcggctgaaatgggtctgggagacgacctgatacgcccaagcattggcattaa
ggctacttcatggagaacggctacgacgactttaaggcgaagttgcttactgcaggcgatttgcgcatgg

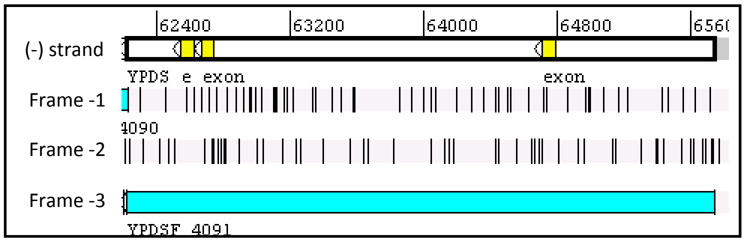

Peptide evidence:

NA

Current sequence (peptide evidence in red): 1174aa
MKALMVRTDFSLGESALKAENAVKIARDAGYTAVISADSMNIASVIPLQRAAGDDMAVICGVKLNVVDDPTYEHRARLAKESERCMESLVRDRSYCFTALIKNEQGYRDVCELMTLANKREQ
FYFVPRLALDQLAAAYAKGNIILLTSDIGSVFQRRDFAKIIGTLVTAGGRDNFYSVVYPHPTPFYDQINVRAMKVASALKIEPVAFYPAYYEAVDDADIKDIAHMVTNNIKIDQPHRLRIPHQRD
NAVNGRRHLLEALKAFSVRMDVPVTAAMASTTQDTIIEACTWRWHELPPALPKMADDEPATLMKLAVAGLRKRLTTKEFGYTPPASEHRVYVDRLKYEMDTLTRLGFCGYFLMVRDLMN
HSRETGIPVGPGRGSSAGSLVAWCIGITNVDPIRHGLLFERFINPERLDLPDADLDFSQARRHEVIEYLNERYGEDYVAGIPNFTYLGAASALRDTARIYGVDAADMAVSKEFKNLEDDSLSLEEL
REQLASLDKYATKYPEAFKAACKLQSLMRGFGRHAAGMIVAGVPLVERTPVELRGNARCIAFDKRYCEAMGLIKLDVLGLATLDLLDSAKRYIKESTGEDINLDAIPLDDRKVLDGFAAGYTQG
VFQLESGPMRKLLKDLGGGIEPMSFKTVVATTALFRPGPIQSGMLDDYVSVAKGFMAPQSLHPVLDELTAETNGVILYQEQTMNATRLLAGFTMAEADGVRKAIGKKDMEKMKSMGEKFV
VQAQAGWIDVEMEDGTTQRIHRAEHFKCEDGALRTVEEALEAGVKLPMAAVRVTGSQPGLSETKAKEIWDAFEKNGAYQFNKSHPVAYSLISYQSMWLKTHYPAEFFAAALTILGEDKHQ
GLVKDALTYGIHVLPPDVNVSSNRIEIRTLEDGSQVLYAPFSAVKGCSENGCQAIMRAREKVGGKFESLEQFEEAVEKRACACNSRVRESLQKVGAFASIEPGSLPATDPERLRDQAELMGNLV
IDAVKASRPFEMNPKRSAEVNVLMTRMAAEMGLGDDLIRPSIGIKPKIMVILDNANGNDGRTGYFMENGYDDFKAKLLTAGDLRMGDLYVTGVCKKVKDKEKDYTKDEIGQFTDFMREEI
NLVRPTYVLTCGSRATSLFNNKSKPSDLVGRKEYLPELDVTVFYGFNPNILYFRPEEGEKLEAILAEVAETISK

Strain: Y. pseudotuberculosis PB1/+
Gene: no ortholog

Error 41

Strain: Y. pestis CO92
Gene: YPCO_2638.5

Classification: novel gene
Boundaries of gene: 2967439..2967690

Oligo evidence:

acaccaaatcagacaagtggggaaatggagcataaagcatctgctgcactcgataatccacgttccagtg

Peptide evidence:

LNDPNSSAIQR
QLAASVLAQANK
SLAGSALSQR
TLNDPNSSAIQR

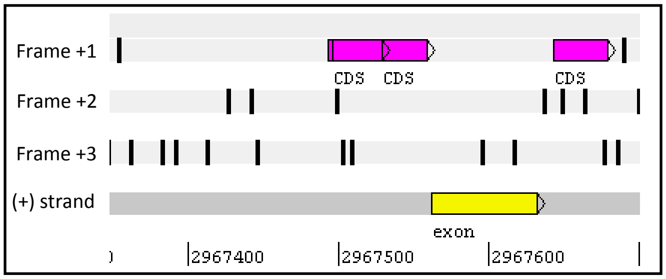

Proposed sequence (peptide evidence in red): 83aa
MSKNTKQTSGKVASIAAKTLNDPNSSAIQRSLAGSALSQRGTPNQTSGEMEHKASAALDNPRSSALTKQLAASVLAQANKDRK

Error 75

Strain: Y. pestis pestoides F
Gene: YPDSF_2615.5

Classification: novel gene
Boundaries of gene: complement(2961352..2961603)

Oligo evidence:

acaccaaatcagacaagtggggaaatggagcataaagcatctgctgcactcgataatccacgttccagtg

Peptide evidence:

QLAASVLAQANK
TLNDPNSSAIQR
SLAGSALSQR

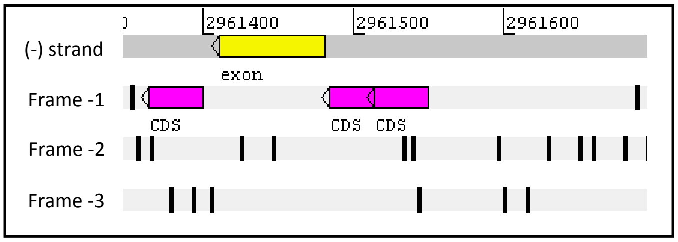

Proposed sequence (peptide evidence in red): 83aa
MSKNTKQTSGKVASIAAKTLNDPNSSAIQRSLAGSALSQRGTPNQTSGEMEHKASAALDNPRSSALTKQLAASVLAQANKDRK

Strain: Y. pseudotuberculosis PB1/+
Gene: YPTS_1189

Classification: annotated as hypothetical protein
Boundaries of gene: 1338362..1338613

Oligo evidence:

acaccaaatcagacaagtggggaaatggagcataaagcatctgctgcactcgataatccacgttccagtg

Peptide evidence:

QLAASVLAQANK
SLAGSALSQR
TLNDPNSSAIQR

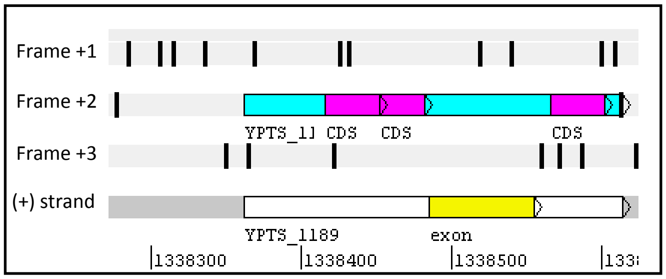

Current sequence (peptide evidence in red): 83aa
MSKNTKQTSGKVASIAAKTLNDPNSSAIQRSLAGSALSQRGTPNQTSGEMEHKASAALDNPRSSALTKQLAASVLAQANKDRK

Error 42

Strain: Y. pestis CO92
Gene: YPO1506.5

Classification: novel gene
Boundaries of gene: 1710711..1710884

Oligo evidence:

atatttctattgctcgctatagccgccttaatctcaatttagtctgttatcattttgtttttacaacaac

Peptide evidence:

SLVGFDDISIAR

Proposed sequence (peptide evidence in red): 57aa
LQDNTVSTLRSLVGFDDISIARYSRLNLNLVCYHFVFTTTIPAIRVLKATHHPTAPP

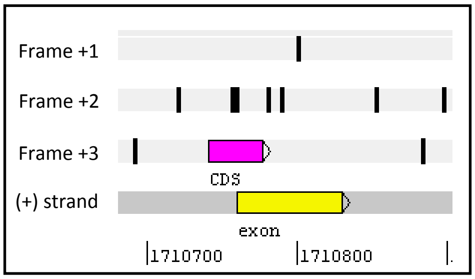

Error lacking threshold of evidence

Strain: Y. pestis pestoides F
Gene: YPDSF_1470.5

Classification: novel gene
Boundaries of gene: complement (1655404..1655577)

Oligo evidence:

atatttctattgctcgctatagccgccttaatctcaatttagtctgttatcattttgtttttacaacaac

Peptide evidence:

NA

Proposed sequence (peptide evidence in red): 57aa
LQDNTVSTLRSLVGFDDISIARYSRLNLNLVCYHFVFTTTIPAIRVLKATHHPTAPP

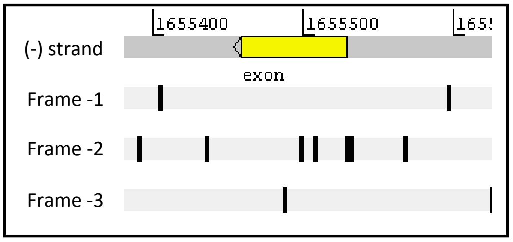

Error lacking threshold of evidence

Strain: Y. pseudotuberculosis PB1/+
Gene: YPTS_1631.5

Classification: novel gene
Boundaries of gene: 1828938..1829111

Oligo evidence:

atatttctattgctcgctatagccgccttaatctcaatttagtctgttatcattttgtttttacaacaac

Peptide evidence:

NA

Proposed sequence (peptide evidence in red): 57aa
LQDNTVSTLRSLVGFDDISIARYSRLNLNLVCYHFVFTTTIPAIRVLKATHHPTAPP

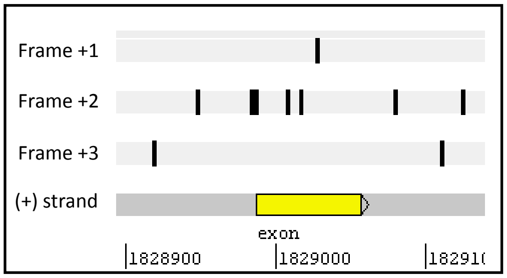

Error 43

Strain: Y. pestis CO92
Gene: YPO2180.5

Classification: novel gene
Boundaries of gene:2455494..2455703

Oligo evidence:

ggttctatgttttatcacggctctgagcttaactattcttaggtatgattttcgtgatttaaatcacaaa

Peptide evidence:

MIVIIAIWVVFNDVKITLSLLLINK

Proposed sequence (peptide evidence in red): 69aa
VVLCFITALSLTILRYDFRDLNHKNIKLTPFSIHFLRMIVIIAIWVVFNDVKITLSLLLINKKHFNGIG

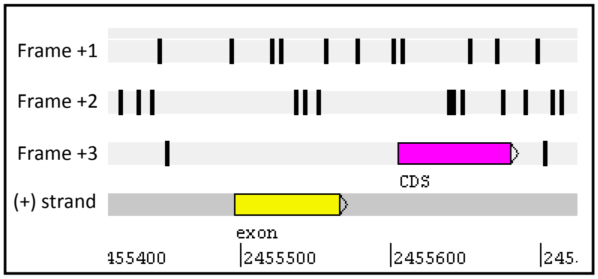

Error lacking threshold of evidence

Strain: Y. pestis pestoides F
Gene: YPDSF_0955.5

Classification: novel gene
Boundaries of gene: complement(1081169..1081375)

Oligo evidence:

ggttctatgttttatcacggctctgagcttaactattcttaggtatgattttcgtgatttaaatcacaaa

Peptide evidence:

NA

Proposed sequence (peptide evidence in red): 69aa
VVLCFITALSLTILRYDFRDLNHKNIKLTPFSIHFLRMIVIIAIWVVFNDVKITLSLLLINKKHFNGIG

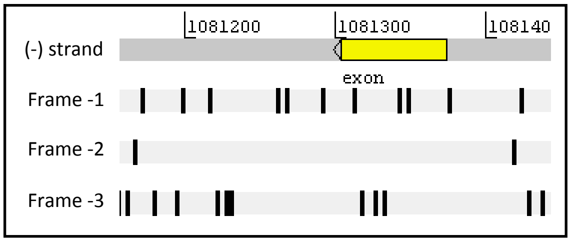

Error lacking threshold of evidence

Strain: Y. pseudotuberculosis PB1/+
Gene: YPTS_2171.5

Classification: novel gene
Boundaries of gene: 2397290..2397496

Oligo evidence:

ggttctatgttttatcacggctctgagcttaactattcttaggtatgattttcgtgatttaaatcacaaa

Peptide evidence:

NA

Proposed sequence (peptide evidence in red): 69aa
VVLCFITALSLTILRYDFRDLNHKNIKLTPFSIHFLRMIVIIAIWVVFNDVKITLSLLLINKKHFNEIG

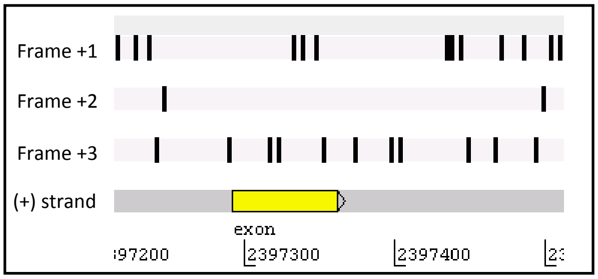

Error 44

Strain: Y. pestis CO92
Gene: YPO199.5

Classification: novel gene
Boundaries of gene: complement(215050..215436)

Oligo evidence:

attattaaaaaagctcctggttttttgcttcgtaaacacgtgataaatccccctcgtttgcactactggc

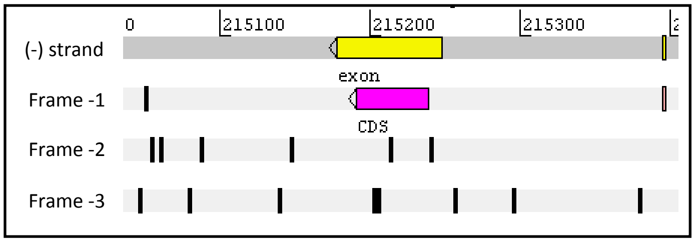

Peptide evidence:

KAPGFLLRKHVINPPR

Proposed sequence (peptide evidence in red): 128aa
VTSKPLVKRTRQTLRSAELGFFGVVVYTRVHTPRFCGHASNAGTLLLATFMLRGLRTSWLIVAIIKKAPGFLLRKHVINPPRLHYWQRTRTQNFMADYLRCQEIYSITRITMPVAADVLGSNPRSEYS

Error lacking threshold of evidence

Strain: Y. pestis pestoides F
Gene: YPDSF_124.5

Classification: novel gene
Boundaries of gene: complement(141564..141947)

Oligo evidence:

attattaaaaaagctcctggttttttgcttcgtaaacacgtgataaatccccctcgtttgcactactggc

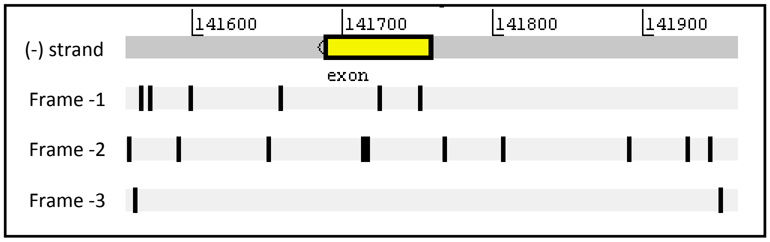

Peptide evidence:

NA

Proposed sequence (peptide evidence in red): 128aa
VTSKPLVKRTRQTLRSAELGFFGVVVYTRVHTPRFCGHASNAGTLLLATFMLRGLRTSWLIVAIIKKAPGFLLRKHVINPPRLHYWQRTRTQNFMADYLRCQEIYSITRITMPVAADVLGSNPRSEYS

Error lacking threshold of evidence

Strain: Y. pseudotuberculosis PB1/+
Gene: YPTS_3897.5

Classification: novel gene
Boundaries of gene: 4334109..4334492

Oligo evidence:

attattaaaaaagctcctggttttttgcttcgtaaacacgtgataaatccccctcgtttgcactactggc

Peptide evidence:

NA

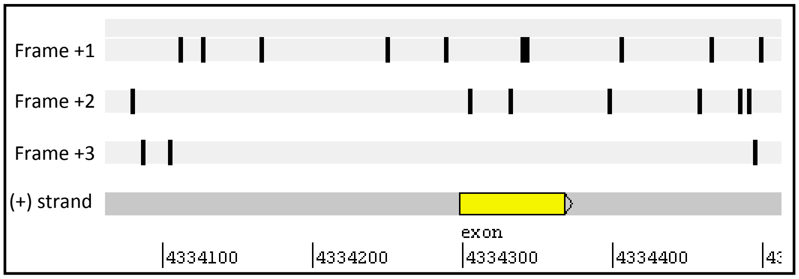

Proposed sequence (peptide evidence in red): 128aa
VTSKPLVKRTRQTLRSAELGFFGVVVYTRVHTPRFCGHASNAGTLLLATFMLRGLRTSWLIVAIIKKAPGFLLRKHVINPPRLHYWQRTRTQNFMADYLRCQEIYSITRITMPVAADVLGSNPRSEYS

Error 45

Strain: Y. pestis CO92
Gene: YPO1651.5

Classification: novel gene
Boundaries of gene: complement (1877589..1877807)

Oligo evidence:

ggtcagtacgctttgtaccgccaggctccttaactcttctcagcatgttatatcccctactgattacctc

Peptide evidence:

RIQTLSLAVILAIVNALIFNMPMFNMPMDNMPMDNA

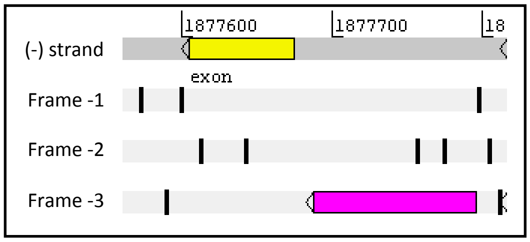

Proposed sequence (peptide evidence in red): 72a
LGHKRIQTLSLAVILAIVNALIFNMPMFNMPMDNMPMDNAPMRQWSVRFVPPGSLTLLSMLYPLLITSFDGM

Error lacking threshold of evidence

Strain: Y. pestis pestoides F
Gene: YPDSF_1795

Classification: annotated as hypothetical protein/upstream start site
Boundaries of gene: 2032150..2032383

Oligo evidence:

ggtcagtacgctttgtaccgccaggctccttaactcttctcagcatgttatatcccctactgattacctc

Peptide evidence:

NA

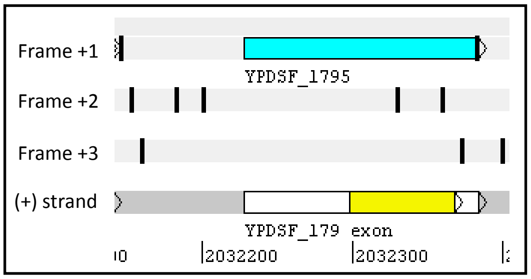

Proposed sequence (peptide evidence in red): 77aa
Current sequence underlined
LGHKRIQTLSLAVILAIVNALIFNIP<u>MFNMPMDNMPMDNMPMDNAPMRQWSVRFVPPGSLTLLSMLYPLLITSFDGM</u>

Error lacking threshold of evidence

Strain: Y. pseudotuberculosis PB1/+
Gene: YPTS_2499.5

Classification: novel gene
Boundaries of gene: 2776866..2777084

Oligo evidence:

ggtcagtacgctttgtaccgccaggctccttaactcttctcagcatgttatatcccctactgattacctc

Peptide evidence:

NA

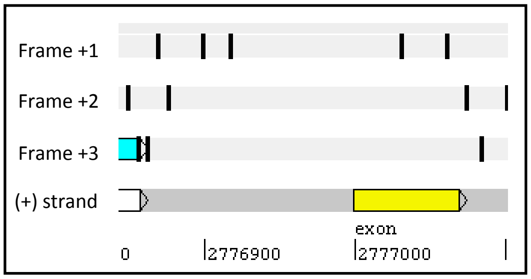

Proposed sequence (peptide evidence in red): 72aa
LGHKRIQTLSLAVILAIVNALIFNIPMFNMPMDNMPMDNAPMRQWSVRFVPPGSLTLLSMLYPLLITSFDGM

Error 47

Strain: Y. pestis CO92
Gene: YPO2654.5

Classification: novel gene
Boundaries of gene: complement(2982147..2982344)

Oligo evidence:

NA

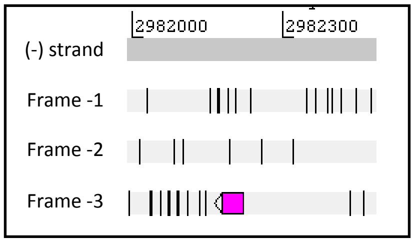

Peptide evidence:

DAAAFNLHLNSQHK

Proposed sequence (peptide evidence in red): 65aa
LPVSVVGVKISKTGNYTILFTVSSLVRQMKMLGAHLDALAKDAAAFNLHLNSQHKNTDNFSGDNT

Strain: Y. pestis pestoides F
Gene: YPDSF_1618

Classification: annotated as hypothetical protein
Boundaries of gene: 1812608..1812946

Oligo evidence:

cagtcaacttttgagaaacagcataagacatggcaatcaagctataatgcgttgcaatatgagctggaac
aaactcaatacggcgaatacactaccggaacagcattcactggtcaggcaaatgaaaatgctgggggcac

Peptide evidence:

DAAAFNLHLNSQHK
MLGAHLDALAK
KLNTANTLPEQHSLVR

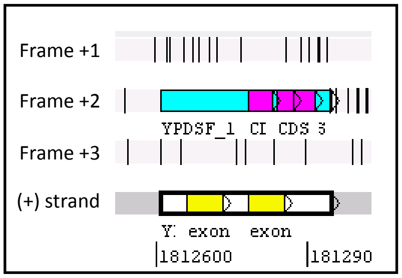

Current sequence (peptide evidence in red): 112aa
MTMRELEVQFLKAMSELQSTFEKQHKTWQSSYNALQYELEQSKARESALRVKNEMLLRKLNTANTLPEQHSLVRQMKMLGAHLDALAKDAAAFNLHLNSQHKNTDNFSGDNT

Strain: Y. pseudotuberculosis
Gene: YPTS_3068

Classification: annotated as hypothetical protein
Boundaries of gene: 3397910..3398248

Oligo evidence:

cagtcaacttttgagaaacagcataagacatggcaatcaagctataatgcgttgcaatatgagctggaac
aaactcaatacggcgaatacactaccggaacagcattcactggtcaggcaaatgaaaatgctgggggcac

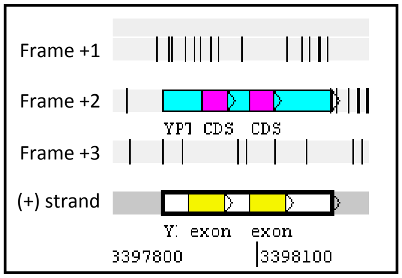

Peptide evidence:

KLNTANTLPEQHSLVR
TWQSSYNALQYELEQSK

Current sequence (peptide evidence in red): 112aa
MTMRELEVQFLKAMSELQSTFEKQHKTWQSSYNALQYELEQSKARESALRVKNEMLLRKLNTANTLPEQHSLVRQMKMLGAHLDALAKDAAAFNLHLNSQHKNTDNFSGDNT

Error 88

Strain: Y. pestis pestoides F plasmid MT
Gene: YPDSF_4088.5

Classification: novel gene
Boundaries of gene: 60858..61109

Oligo evidence:

NA

Peptide evidence:

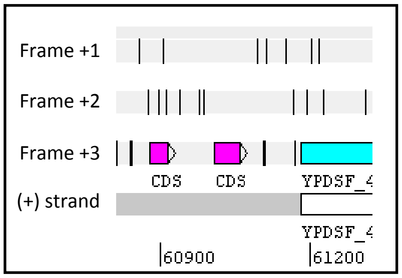

HIQEAIEYALSK
SVWSTPDVPEHHATQIR

Proposed sequence (peptide evidence in red): 83aa
MKSHPNKHIQEAIEYALSKGWVWVPAGKSAHCFCKLRCGDKSGEHTSHHRSVWSTPDVPEHHATQIRQAVDQCGRIKNQMSKK

Error 48

Strain: Y. pestis CO92 plasmid MT
Gene: YPMT1.53c.5

Classification: novel gene
Boundaries of gene: 55421..55672

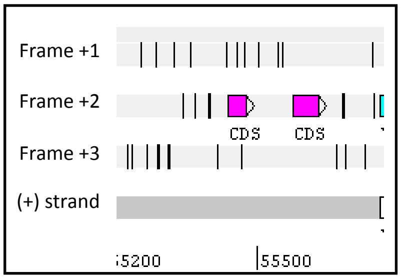

Oligo evidence:

NA

Peptide evidence:

HIQEAIEYALSK
SVWSTPDVPEHHATQIR

Proposed sequence (peptide evidence in red): 83aa
MKSHPNKHIQEAIEYALSKGWVWVPAGKSAHCFCKLRCGDKSGEHTSHHRSVWSTPDVPEHHATQIRQAVDQCGRIKNQMSKK

Strain: Y. pseudotuberculosis PB1/+
Gene: no ortholog

Error 49

Strain: Y. pestis CO92
Gene: YPO0099

Classification: upstream start site
Boundaries of gene: complement (108358..109674)

Oligo evidence:

atattagaacaaaatggttggggaaggcgctgttaacttcgggttattccacaacgttcatagctgcttt
aagtcaatatacgctatgacgctttccccttgaaaagtttggtcaatttcctgggcgtataatctctat

Peptide evidence:

DAVEYIR

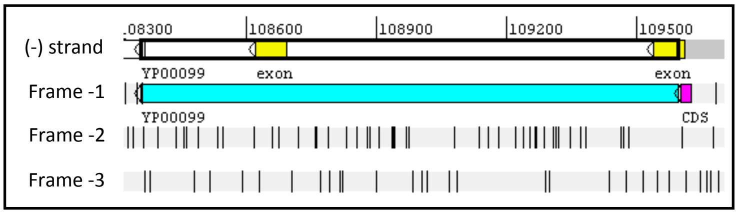

Proposed sequence (peptide evidence in red): 438aa
MMGITERGMSSNQIFRRDAVEYIRTKWLGKALLTSGYSTTFIAALCAIFLVLLITLIIYGTYTRRINVNGEVISQPHPINIFSPQQGFITKKWVEVGDIVRKGQHLYQIDVSRTTFSGNVSLNSLEAI
NNQLSQIDSIINNTQKNKELTLLNLRQQLAQYQKAHKKSQELVDNAGKGMDDMRRTMASYGTYQRQGLITKDQLTNQRSLFYQQQNAFQSLNTQLIQESLQIAKLESEISTRASDFDNDISQ
YLFQKGDLKRQLAEVDASGMLLINSPSDGKIENMSVTQGQMVNVNDSLVQLTPSDNPYYCLVLWVPNNSVPYINTGDKVNIRYDAFPFEKFGQFPGRIISISNVPVSQQEIASYNIAPRLPNG
GLIEPYYKVIVALDDIHFRYQSKPLMLSNGLKANVTLFLEKRPLYQWMLSPFYDIKKSVTGPVNE

Strain: Y. pestis pestoides F
Gene: YPDSF_3807

Classification: annotated as hypothetical protein
Boundaries of gene: 4375437..4376750

Oligo evidence:

atattagaacaaaatggttggggaaggcgctgttaacttcgggttattccacaacgttcatagctgcttt
aagtcaatatacgctatgacgctttccccttgaaaagtttggtcaatttcctgggcgtataatctctat

Peptide evidence:

NA

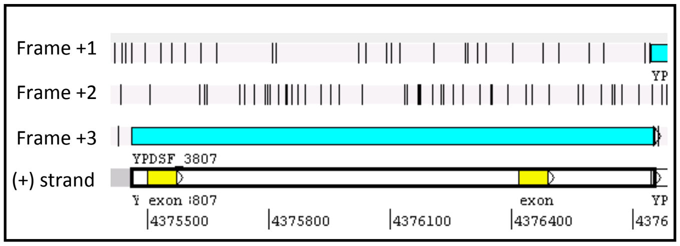

Current sequence (peptide evidence in red): 430aa
MSSNQIFRRDAVEYIRTKWLGKALLTSGYSTTFIAALCAIFLVLLITLIIYGTYTRRINVNGEVISQPHPINIFSPQQGFITKKWVEVGDIVRKGQHLYQIDVSRTTFSGNVSLNSLEAINNQLSQIDSI
INNTQKNKELTLLNLRQQLAQYQKAHKKSQELVDNAGKGMDDMRRTMASYGTYQRQGLITKDQLTNQRSLFYQQQNAFQSLNTQLIQESLQIAKLESEISTRASDFDNDISQYLFQKGDLK
RQLAEVDASGMLLINSPSDGKIENMSVTQGQMVNVNDSLVQLTPSDNPYYCLVLWVPNNSVPYINTGDKVNIRYDAFPFEKFGQFPGRIISISNVPVSQQEIASYNIAPRLPNGGLIEPYYKVI
VALDDIHFRYQSKPLMLSNGLKANVTLFLEKRPLYQWMLSPFYDIKKSVTGPVNE

Strain: Y. pseudotuberculosis PB1/+
Gene: YPTS_0094

Classification: annotated as secretion protein HlyD family protein
Boundaries of gene: 98853..100169

Oligo evidence:

aagtcaatatacgctatgacgctttccccttgaaaagtttggtcaatttcctgggcgtataatctctat
tattagaacaaaatggttggggaaggcgctgttaacttcgggttattccacaacgttcatagctgcttt

Peptide evidence:

NA

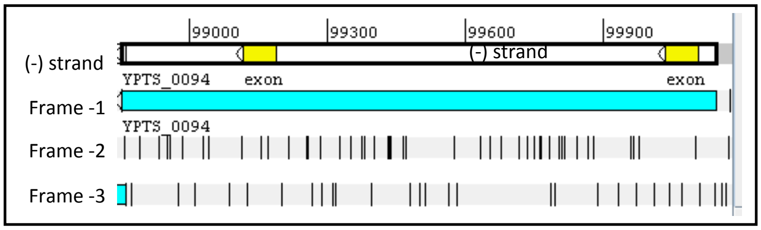

Current sequence (peptide evidence in red): 430aa
MSSNQIFRRDAVEYIRTKWLGKALLTSGYSTTFIAALCAIFLVLLITLIIYGTYTRRINVNGEVISQPHPINIFSPQQGFITKKWVEVGDIVRKGQHLYQIDVSRTTFSGNVSLNSLEAINNQLSQIDSI
INNTQKNKELTLLNLRQQLAQYQKAHKKSQELVDNAGKGMDDMRRTMASYGTYQRQGLITKDQLTNQRSLFYQQQNAFQSLNTQLIQESLQIAKLESEISTRASDFDNDISQYLFQKGDLK
RQLAEVDASGMLLINSPSDGKIENMSVTQGQMVNVNDSLVQLTPSDNPYYCLVLWVPNNSVPYINTGDKVNIRYDAFPFEKFGQFPGRIISISNVPVSQQEIASYNIAPRLPNGGLIEPYYKVI
VALDDIHFRYQSKPLMLSNGLKANVTLFLEKRPLYQWMLSPFYDIKKSVTGPVNE

Error 50

Strain:Y. pestis CO92
Gene: YPO0453

Classification: upstream start site
Boundaries of gene: 478173..478562

Oligo evidence:

ttatcatgacaaaacaatttttgactgacgaaaaacaagtgtcggattcacttaataggcagacggctgg
agcactggttgagctttgtcgcgctgttgcaaaacgcgattacacaagatcttcatctgcctttactgca

Peptide evidence:

SQLGAGIATITR
SWLEAQLLANKR
TKQFLTDEKQVSDSLNR

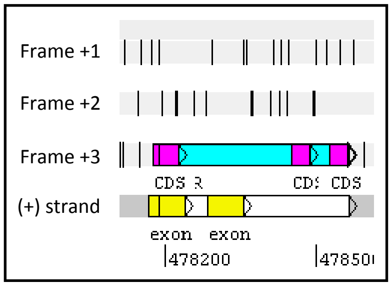

Proposed sequence (peptide evidence in red): 130aa
Current annotation is underlined

MTKQFLTDEKQVSDSLNRQTAGNPYSAADPALSAEDNQHWLSFVALLQNAITQDLHLPLLQLMLTPDERTALGTRVRIIEELMRGELSQRELKSQLGAGIATITRGSNSLKTAPPQLKSWLEA
QLLANKR

Strain: Y. pestis pestoides F
Gene: YPDSF_3181

Classification: annotated as Trp operon repressor
Boundaries of gene: complement (3620475..3620873)

Oligo evidence:

ttatcatgacaaaacaatttttgactgacgaaaaacaagtgtcggattcacttaataggcagacggctgg
agcactggttgagctttgtcgcgctgttgcaaaacgcgattacacaagatcttcatctgcctttactgca

Peptide evidence:

SQLGAGIATITR
SWLEAQLLANKR

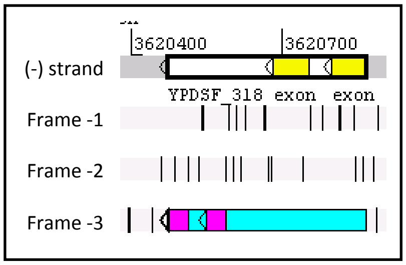

Current sequence (peptide evidence in red): 130aa
MTKQFLTDEKQVSDSLNRQTAGNPYSAADPALSAEDNQHWLSFVALLQNAITQDLHLPLLQLMLTPDERTALGTRVRIIEELMRGELSQRELKSQLGAGIATITRGSNSLKTAPPQLKSWLEA
QLLANKR

Strain: Y. pseudotuberculosis PB1/+
Gene: YPTS_0618

Classification: annotated as Trp operon repressor
Boundaries of gene: 687936..688334

Oligo evidence:

ttatcatgacaaaacaatttttgactgacgaaaaacaagtgtcggattcacttaataggcagacggctgg
agcactggttgagctttgtcgcgctgttgcaaaacgcgattacacaagatcttcatctgcctttactgca

Peptide evidence:

SQLGAGIATITR
SWLEAQLLANKR

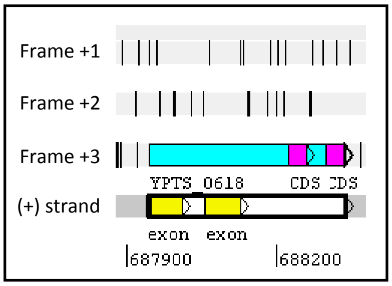

Current sequence (peptide evidence in red): 130aa
MTKQFLTDEKQVSDSLNRQTAGNPYSAADPALSAEDNQHWLSFVALLQNAITQDLHLPLLQLMLTPDERTALGTRVRIIEELMRGELSQRELKSQLGAGIATITRGSNSLKTAPPQLKSWLEA
QLLANKR

Error 51

Strain: Y. pestis pestoides F
Gene: YPDSF_0112 /YPDSF_0111

Classification: expressed pseudogene
Boundaries of gene: Frame-2: complement (130808..131353)
Frame -1: complement (130372..130806)

Oligo evidence:

acatgcgctgaaacaacgttggccagcagcagaaatccacgtattagcgaataaatataatcaatccgtg
aaactcaatgcgcaggctgaattgctcatttttcatgatccagcagaacaaccatccgagcagcaattag

Peptide evidence:

HEALSGFALMPELGR
NPDVSAVHTYVYSK

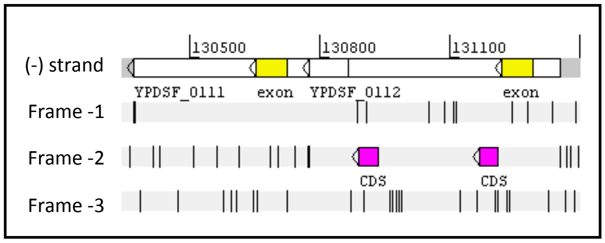

Proposed sequence (peptide evidence in red):
Frame-2: 182aa
MQRILVIRIDFLGDMVCTTALLHALKQRWPAAEIHVLANKYNQSVLARNPDVSAVHTYVYSKQCERNQRPGRLMALFDRLALIWRLRRLGFDLLLIPNGGMNKNSIQFAKQLNVADRRWH
TAETEFDDRNPEHVATRPIRHEALSGFALMPELGRVDIETLKLHVYPEPQLQAKWQADLGGK

Frame-1: 145aa
DRPRVGLFISNKSAQRRWSWDKWRQLALKLNAQAELLIFHDPAEQPSEQQLAGLTARCLSTPSTDDLMAAMSQLDLVISADSAPVHIGSALQIPVVALFESRPEKYLRWYPLGVRHVLVHAG
PQVEDIHVDAVESAARSLLTTD

Strain: Y. pestis CO92
Gene: YPO0186

Classification: annotated as putative sugar transferase
Boundaries of gene: 203866..204846

Oligo evidence:

acatgcgctgaaacaacgttggccagcagcagaaatccacgtattagcgaataaatataatcaatccgtg
aaactcaatgcacaggctgaattgctcatttttcatgatccagcagaacaaccatccgagcagcaattag

Peptide evidence:

HEALSGFALMPELGR
HVLVHAGPQVEDIHVDAVESAAR
LHVYPEPQLQAK
LNAQAELLIFHDPAEQPSEQQLAGLTAR
NPDVSAVHTYVYSK
WHTAETEFDDRNPEHVATRPIR
WPAAEIHVLANKYNQSVLAR

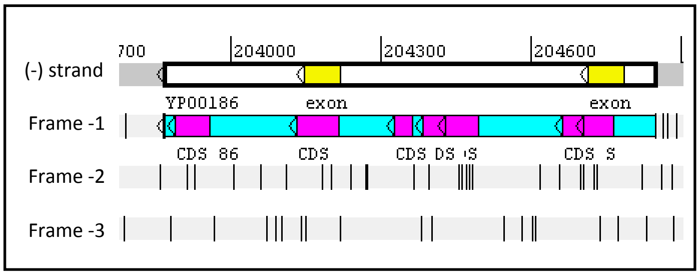

Current sequence (peptide evidence in red): 326aa
MQRILVIRIDFLGDMVCTTALLHALKQRWPAAEIHVLANKYNQSVLARNPDVSAVHTYVYSKQCERNQRPGRLMALFDRLALIWRLRRLGFDLLLIPNGGMNKNSIQFAKQLNVADRRWH
TAETEFDDRNPEHVATRPIRHEALSGFALMPELGRVDIETLKLHVYPEPQLQAKWQADLGGKDRPRVGLFISNKSAQRRWSWDKWRQLALKLNAQAELLIFHDPAEQPSEQQLAGLTARCL
STPSTDDLMAAMSQLDLVISADSAPVHIGSALQIPVVALFESRPEKYLRWYPLGVRHVLVHAGPQVEDIHVDAVESAARSLLTTD

Strain: Y. pseudotuberculosis PB1/+
Gene: YPTS_3910

Classification: annotated as glycosyl transferase family protein
Boundaries of gene: 4344683..4345663

Oligo evidence:

acatgcgctgaaacaacgttggccagcagcagaaatccacgtattagcgaataaatataatcaatccgtg
aaactcaatgcacaggctgaattgctcatttttcatgatccagcagaacaaccatccgagcagcaattag

Peptide evidence:

HEALSGFALMPELGR
HVLVHAGPQVEDIHVDAVESAAR
LHVYPEPQLQAK
NPDVSAVHTYVYSK

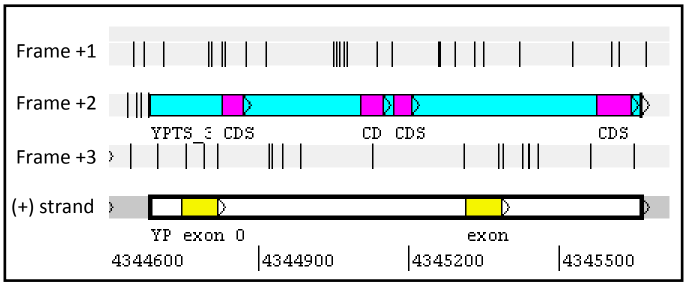

Current sequence (peptide evidence in red): 326aa
MQRILVIRIDFLGDMVCTTALLHALKQRWPAAEIHVLANKYNQSVLTRNPDVSAVHTYVYSKQCERNPRPGRLMALFDRLALIWRLRRLGFDLLLIPNGGMNKNSIQFAKQLNVADRRWHT
AETEFDDRNPEHVATRPIRHEALSGFALMPELGRVDIETLKLHVYPEPQLQAKWRADLGEKDRPRVGLFISNKSAQRRWSWDKWRQLALKLNAQAELLIFHDPAEQPSEQQLAGLTARCLST
PSTDDLMAAMSQLDLVISADSAPVHIGSALQIPVVALFESRPEKYLRWYPLGVRHVLVHAGPQVEDIHVDAVESAARSLLTTD

Error 52

Strain: Y. pestis pestoides F
Gene: YPDSF_0343 /YPDSF_0344

Classification: expressed pseudogene/frameshift
Boundaries of gene: Frame+3: 386646..387149
Frame+2: 387149.. 387298

Oligo evidence:

atataagttaattcaattaatgcataagtccggtaaaccaattggattgatgtgtatttctccggtgatg
attgaaattatgggggggagcatgttatttgccctgctgatgacgttgtcattgatttggaaaataaagt
aaagtggtgacgacaccggcttacatgttggctggttccatttcagaagcggctaaaggtattgataaat
ttggctggttccatttcagaagcggctaaaggtattgataaattggtgacgaaagtcttggatttaactg

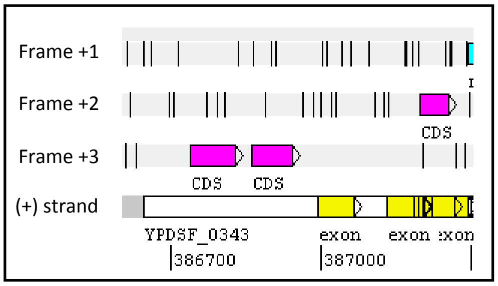

Peptide evidence:

AGAEVLFFAPDKPQLHVINHITGEIVAEER
GLITPLSAADPEVLDALIVLGGFGAAK
VVTTPAYMLAGSISEAAK

Proposed sequence (peptide evidence in red):

Frame+3: 168aa
MKTVGVVLSGCGVLDGAEIHESVLTMLALDRAGAEVLFFAPDKPQLHVINHITGEIVAEERNVLVESARIARGLITPLSAADPEVLDALIVLGGFGAAKNLCDFAIKGGECSVEPDLYKLIQLMH
KSGKPIGLMCISPVMLPKLLGKPIRLTIGNDPDTIDAIEIMGG

Frame+2: 49aa
EHVICPADDVVIDLENKVVTTPAYMLAGSISEAAKGIDKLVTKVLDLTE

Strain: Y. pestis CO92
Gene: YPO3553

Classification: annotated as isoprenoid biosynthesis protein with amidotransferase-like domain
Boundaries of gene: 3961893..3962546

Oligo evidence:

tggcctgatcacccccctttctgcggctgatccagaggtattagatgcactaatcgtaccgggaggtttt
aataagttaattcaattaatgcataagtccggtaaaccaattggattgatgtgtatttctccggtgatg
aaagtggtgacgacaccggcttacatgttggctggttccatttcagaagcggctaaaggtattgataaat
ttggctggttccatttcagaagcggctaaaggtattgataaattggtgacgaaagtcttggatttaactg

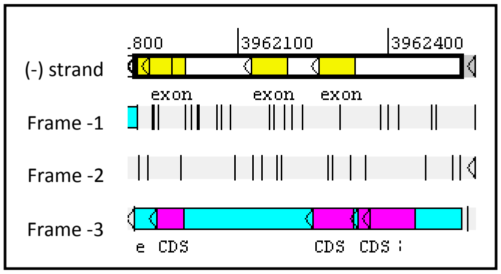

Peptide evidence:

AGAEVLFFAPDKPQLHVINHITGEIVAEER
GLITPLSAADPEVLDALIVPGGFGAAK
NVLVESAR
VVTTPAYMLAGSISEAAK

Current sequence (peptide evidence in red): 217aa
MKTVGVVLSGCGVLDGAEIHESVLTMLALDRAGAEVLFFAPDKPQLHVINHITGEIVAEERNVLVESARIARGLITPLSAADPEVLDALIVPGGFGAAKNLCDFAIKGGECSVEPDLYKLIQLMH
KSGKPIGLMCISPVMLPKLLGKPIRLTIGNDPDTIDAIEIMGGEHVICPADDVVIDLENKVVTTPAYMLAGSISEAAKGIDKLVTKVLDLTE

Strain: Y. pseudotuberculosis PB1/+
Gene: YPTS_3683

Classification: annotated as isoprenoid biosynthesis protein with amidotransferase-like domain
Boundaries of gene: 4103437..4104090

Oligo evidence:

tggcctgatcacccccctttctgcggctgatccagaggtattagatgcactaatcgtaccgggaggtttt
atataagttaattcaattaatgcataagtccggtaaaccaattggattgatgtgtatttctccggtgatg
aaagtggtgacgacaccggcttacatgttggctggttccatttcagaagcggctaaaggtattgataaat

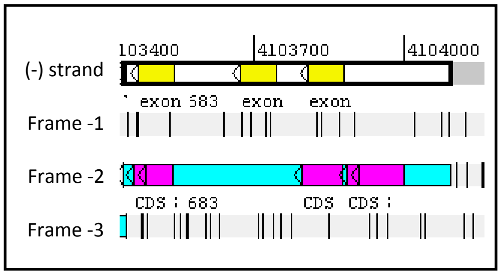

Peptide evidence:

AGAEVLFFAPDKPQLHVINHITGEIVAEER
GIDKLVTK
GLITPLSAADPEVLDALIVPGGFGAAK
NVLVESAR
VVTTPAYMLAGSISEAAK

Current sequence (peptide evidence in red): 217aa
MKTVGVVLSGCGVLDGAEIHESVLTMLALDRAGAEVLFFAPDKPQLHVINHITGEIVAEERNVLVESARIARGLITPLSAADPEVLDALIVPGGFGAAKNLCDFAIKGGECSVEPDLYKLIQLMH
KSGKPIGLMCISPVMLPKLLGKPIRLTIGNDPDTIDAIEIMGGEHVICPADDVVIDLENKVVTTPAYMLAGSISEAAKGIDKLVTKVLDLTE

Error 53

Strain: Y. pestis pestoides F
Gene: YPDSF_0661/YPDSF_0662
Oligo evidence:

Classification: expressed pseudogene
Boundaries of gene: Frame+1:743029..743982
Frame+3: 743994..745100

actaactttatggagtacttctacgattacgactactggaagttggcgcaggtgattgattttatctcct
ctcaccgtcgtaccgatggtgagagtgaatttatcgtggtagaaaactacagtaatgacagcaaatcgct
agcagccggtacaaggtagcctgacgcttgccccatggggaagccgagtgctaactcgctatctcgaata

Peptide evidence:

FHGAVVDHVGHIDTR
DMVDQQPVQGSLTLAPWGSR

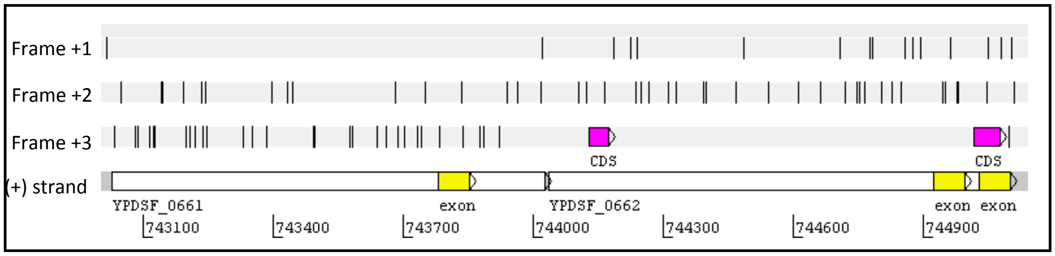

Proposed sequence (peptide evidence in red):
Frame+1: 318aa
MSKFPPLSAKVSALLHGADYNPEQWENYPDIIDKDIAMMKQAKCNVMSVGIFSWVKLEPSEGEYNFSWLDELIEKLYAAGIHIFLATPSGARPAWMSQKYPEVLRVGRDRVPALHGGRHNHCMTSPVYRQKVRQINQ
KLAERYAHHPAVIGWHISNEYGGECHCHSCQQKFRLWLQDRYQTLDNLNEAWWSAFWSHTYSDWSQIESPAPQGEVSIHGLNLDWRRFNTAQVTEFCSEEAKPLKAANPELPVTTNFMEYFYDYDYWKLAQVIDFIS
WDSYPMWHREKDETQLACYTAMYHDLMRTLKQGRPFVLMESTPS

Frame+3:368aa
ATNWQPTSKLKKPGMHILSSLQAVAHGADAVQYFQWRKSRGSVEKFHGAVVDHVGHIDTRVGREVSELGRILEAMSPVMGSKVDADVAIIFDWESRWAMDDAEGPRNCGLEYEKTVAEHYRPFWERGIAVDIINAD
CDLSGYKLVIAPMLYMVREGFAERATRFVEQGGQFVATYWSGIVNESDLCHLGGFPGPLRPLLGIWSEEIDCLADGESNQVQGLAGNKAGLQGPYQAIHLCDLIHLEGATAVARYRDDFYADRAAVTVNFVGEGKAWY
VASRNDAAFQRDFFMNIAEELNLARALDTQFPYGVTAHRRTDGESEFIVVENYSNDSKSLVLPAVYRDMVDQQPVQGSLTLAPWGSRVLTRYLE

Strain: Y. pestis CO92
Gene: YPO0852

Classification: annotated as puative beta-galactosidase
Boundaries of gene: 936164..938224

Oligo evidence:

actaactttatggagtacttctacgattacgactactggaagttggcgcaggtgattgattttatctcct
ctcaccgtcgtaccgatggtgagagtgaatttatcgtggtagaaaactacagtaatgacagcaaatcgct
agcagccggtacaaggtagcctgacgcttgccccatggggaagccgagtgctaactcgctatctcgaata

Peptide evidence:

DFFMNIAEELNLAR
DMVDQQPVQGSLTLAPWGSR
FHGAVVDHVGHIDTR

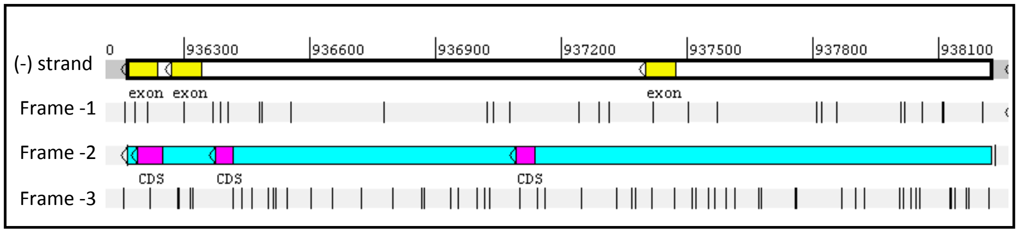

Current sequence (peptide evidence in red): 686aa
MSKFPPLSAKVSALLHGADYNPEQWENYPDIIDKDIAMMKQAKCNVMSVGIFSWVKLEPSEGEYNFSWLDELIEKLYAAGIHIFLATPSGARPAWMSQKYPEVLRVGRDRVPALHGGRHNHCMTSPVYRQKVRQINQ
KLAERYAHHPAVIGWHISNEYGGECHCHSCQQKFRLWLQDRYQTLDNLNEAWWSAFWSHTYSDWSQIESPAPQGEVSIHGLNLDWRRFNTAQVTEFCSEEAKPLKAANPELPVTTNFMEYFYDYDYWKLAQVIDFIS
WDSYPMWHREKDETQLACYTAMYHDLMRTLKQGRPFVLMESTPSATNWQPTSKLKKPGMHILSSLQAVAHGADAVQYFQWRKSRGSVEKFHGAVVDHVGHIDTRVGREVSELGRILEAMSPVMGSKVDADVAII
FDWESRWAMDDAEGPRNCGLEYEKTVAEHYRPFWERGIAVDIINADCDLSGYKLVIAPMLYMVREGFAERATRFVEQGGQFVATYWSGIVNESDLCHLGGFPGPLRPLLGIWSEEIDCLADGESNQVQGLAGNKAGL
QGPYQAIHLCDLIHLEGATAVARYRDDFYADRAAVTVNFVGEGKAWYVASRNDAAFQRDFFMNIAEELNLARALDTQFPYGVTAHRRTDGESEFIVVENYSNDSKSLVLPAVYRDMVDQQPVQGSLTLAPWGSRVLT
RYLE

Strain: Y. pseudotuberculosis PB1/+
Gene: YPTS_3219

Classification: annotated as glycoside hydrolase family 42 protein
Boundaries of gene: 3568067..3570127

Oligo evidence:

actaactttatggagtacttctacgattacgactactggaagttggcgcaggtgattgattttatctcct
ctcaccgtcgtaccgatggtgagagtgaatttatcgtggtagaaaactacagtaatgacagcaaatcgct
agcagccggtacaaggtagcctgacgcttgccccatggggaagccgagtgctaactcgctatctcgaata

Peptide evidence:

DMVDQQPVQGSLTLAPWGSR
FHGAVVDHVGHIDTR

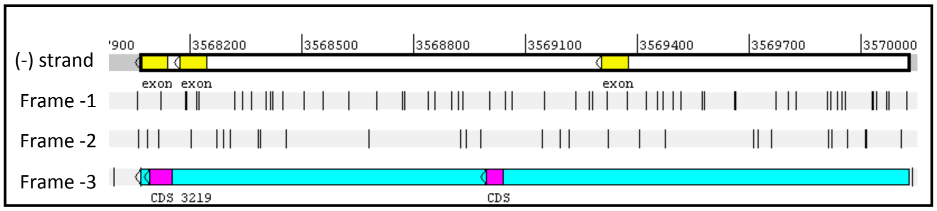

Current sequence (peptide evidence in red): 686aa
MSKFPPLSAKVSALLHGADYNPEQWENYPDIIDKDIAMMKQAKCNVMSVGIFSWVKLEPSEGEYNFSWLDELIEKLYAAGIHIFLATPSGARPAWMSQKYPEVLRVGRDRVPALHGGRHNHCMTSPVYRQKVRQINQ
KLAERYAHHPAVIGWHISNEYGGECHCHSCQQKFRLWLQDRYQTLDNLNEAWWSAFWSHTYSDWSQIESPAPQGEVSIHGLNLDWRRFNTAQVTEFCSEEAKPLKAANPELPVTTNFMEYFYDYDYWKLAQVIDFIS
WDSYPMWHREKDETQLACYTAMYHDLMRTLKQGRPFVLMESTPSATNWQPTSKLKKPGMHILSSLQAVAHGADAVQYFQWRKSRGSVEKFHGAVVDHVGHIDTRVGREVSELGRILEAMSPVMGSKVDADVAII
FDWESRWAMDDAEGPRNCGLEYEKTVAEHYRPFWERGIAVDIINADCDLSGYKLVIAPMLYMVREGFAERATRFVEQGGQFVATYWSGIVNESDLCHLGGFPGPLRPLLGIWSEEIDCLADGESNQVQGLAGNKAGL
QGPYQAIHLCDLIHLEGATAVARYRDDFYADRAAVTVNFVGEGKAWYVASRNDAAFQRDFFMNIAEELNLARALDTQFPYGVTAHRRTDGESEFIVVENYSNDSKSLVLPAVYRDMVDQQPVQGSLTLAPWGSRVLT
RYLE

Error 54

Strain: Y. pestis pestoides F
Gene: YPDSF_0663

Classification: expressed pseudogene
Boundaries of gene: 745212..745523

Oligo evidence:

ggtaaacgatctctccaaggttaattctgaagcgctacaacaagcgggggcattaggcgttattattctt

Peptide evidence:

VNSEALQQAGALGVIILGQEVHAIFGK

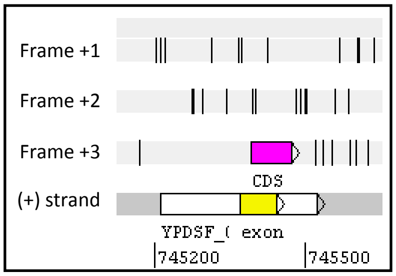

Proposed sequence (peptide evidence in red): 103aa
MISLKSFMHYFSHPKPTPSFTDEEKQQAEMLLHYLGGKENIKNIDACITRLRVTVNDLSKVNSEALQQAGALGVIILGQEVHAIFGKQSDNLRKILDEHFSQS

Strain: Y. pestis CO92
Gene: YPO0851

Classification: annotated as PTS transport protein (partial)
Boundaries of gene: 935741..936052

Oligo evidence:

ggtaaacgatctctccaaggttaattctgaagcgctacaacaagcgggggcattaggcgttattattctt

Peptide evidence:

VNSEALQQAGALGVIILGQEVHAIFGK

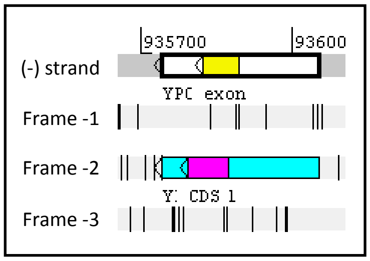

Current sequence (peptide evidence in red): 103aa
MISLKSFMHYFSHPKPTPSFTDEEKQQAEMLLHYLGGKENIKNIDACITRLRVTVNDLSKVNSEALQQAGALGVIILGQEVHAIFGKQSDNLRKILDEHFSQS

Strain: Y. pseudotuberculosis PB1/+
Gene: YPTS_3218

Classification: annotated as PTS system transporter subunit EIIB
Boundaries of gene: 3567644..3567955

Oligo evidence:

ggtaaacgatctctccaaggttaattctgaagcgctacaacaagcgggggcattaggcgttattattctt

Peptide evidence:

VNSEALQQAGALGVIILGQEVHAIFGK

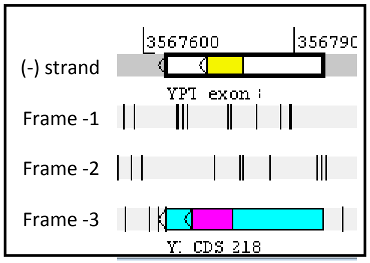

Current sequence (peptide evidence in red): 103aa
MISLKSFMHYFSHPKPTPSFTDEEKQQAEMLLHYLGGKENIKNIDACITRLRVTVNDLSKVNSEALQQAGALGVIILGQEVHAIFGKQSDNLRKILDEHFSQS

Error 55

Strain: Y. pestis pestoides F
Gene: YPDSF_0859

Classification: expressed pseudogene/upstream start site
Boundaries of gene: 978809..979435

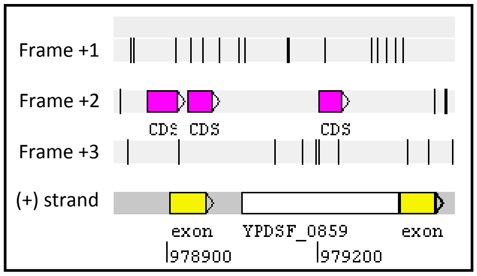

Oligo evidence:

tacagtgggttaaaacagtaggagactccggcaaatatgttgaactggttggtgcgccgtcagataacaa
tttttgactgtatgttgatcacaaaagcgaatgtcgacaaatatacatcaccgtttgtgatgtcagagta

Peptide evidence:

AQLVSNNAQGAALGAIQWVK
VGAFDGSPDAVDAVK
YVELVGAPSDNNAATR

Proposed sequence (peptide evidence in red): 208aa
LPVFIINAELNQSGLAKAQLVSNNAQGAALGAIQWVKTVGDSGKYVELVGAPSDNNAATRANGYSSVLSQYPDLKRVGKEVANWDRTQGYNKMQSMLQANPDIKGVISGNDEMALGAI
AALKEAGKIDQVKVGAFDGSPDAVDAVKAGTLAYTVLQPVAVFSAKAIEQADNFLKTGKTGARGEKQLFDCMLITKANVDKYTSPFVMSE

Strain: Y. pestis CO92
Gene: YPO2285

Classification: annotated as putative ribose-binding periplasmic protein (partial)
Boundaries of gene: 2565022..2565645

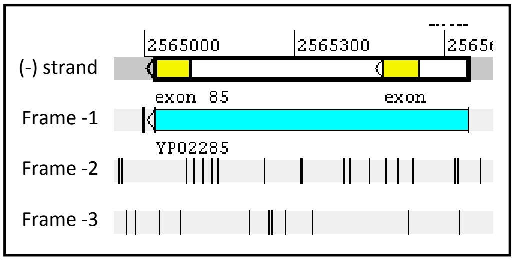

Oligo evidence:

tacagtgggttaaaacagtaggagactccggcaaatatgttgaactggttggtgcgccgtcagataacaa
tttttgactgtatgttgatcacaaaagcgaatgtcgacaaatatacatcaccgtttgtgatgtcagagta

Peptide evidence:

NA

Current sequence (peptide evidence in red): 208aa
LPVFIINAELNQSGLAKAQLVSNNAQGAALGAIQWVKTVGDSGKYVELVGAPSDNNAATRANGYSSVLSQYPDLKRVGKEVANWDRTQGYNKMQSMLQANPDIKGVISGNDEMALGAI
AALKEAGKIDQVKVGAFDGSPDAVDAVKAGTLAYTVLQPVAVFSAKAIEQADNFLKTGKTGARGEKQLFDCMLITKANVDKYTSPFVMSE

Strain: Y. pseudotuberculosis PB1/+
Gene: YPTS_2281

Classification: annotated as periplasmic binding protein/LacI transcriptional regulator
Boundaries of gene: 2516314..2517264

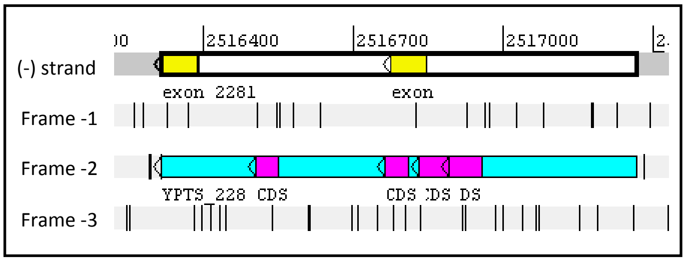

Oligo evidence:

tacagtgggttaaaacagtaggagactccggcaaatatgttgaactggttggtgcgccgtcagataacaa
tttttgactgtatgttgatcacaaaagcgaatgtcgacaaatatacatcaccgtttgtgatgtcagagta

Peptide evidence:

AIAANIPVFIINAELNQSGLAK
AQLVSNNAQGAALGAIQWVK
VGAFDGSPDAVDAVK
YVELVGAPSDNNAATR

Current sequence (peptide evidence in red): 316aa
MFNSKHFLLLVAMTFATVFSTASWANNGLMTIIINDPSNPYWFTEGLVAKATAEKMGYSATVSAHKGDTNTESQLIDTAITNKSKAIILDPANADGSIGSVKKAIAANIPVFIINAELNQSGLAK
AQLVSNNAQGAALGAIQWVKTVGDSGKYVELVGAPSDNNAATRANGYSSVLSQYPDLKRVGKEVANWDRTQGYNKMQSMLQANPDIKGVISGNDEMALGAIAALKEAGKIDQVKVGA
FDGSPDAVDAVKAGTLAYTVLQPVAVFSAKAIEQADNFLKTGKTGARGEKQLFDCMLITKANVDKYTSPFVMSE

Error 56

Strain: Y. pestis pestoides F
Gene: YPDSF_1048/1049

Classification: expressed pseudogene
Boundaries of gene: frame-2: complement (1180811..1180966)
frame-1: complement (1180366..1180815)

Oligo evidence:

tacgtcaatgactcagtctaagaaatttagaaatcataaatggatgtatggttggctaggtttgggggct
caccgttgcaagatcttaatcagatcagctccaaagctatttattggtcaggaagcgcgctttggcggta
tctggaaccggttgatttccgtggacatttagtgacagttgttgggccgattgtcggggttgaagccggc

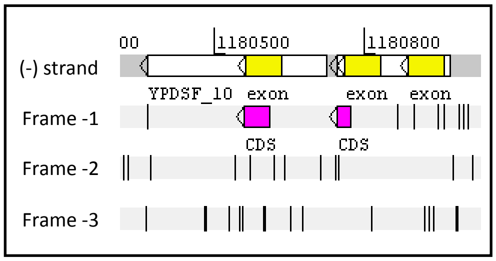

Peptide evidence:

FGGTVVNVK
GHLVTVVGPIVGVEAGK

Proposed sequence (peptide evidence in red):
frame-2: 52aa
MAMNTSMTQSKKFRNHKWMYGWLGLGALLLSGCVTIPPAIQGTTATPLQDLN

frame-1: 149aa
LIRSAPKLFIGQEARFGGTVVNVKNEPNRTRLEIASVPLDSGARPILGEPSQGRVIAYVDGFLEPVDFRGHLVTVVGPIVGVEAGKIGMTPYNFVVIKATGYKRWHIAQQVIIPPTF
GPWGYRPADMWGPGWPGWYNPGPAEIQTIVTD

Strain: Y. pestis CO92
Gene: YPO2073

Classification: annotated as putative lipoprotein
Boundaries of gene: 2353262..2353849

Oligo evidence:

tacgtcaatgactcagtctaagaaatttagaaatcataaatggatgtatggttggctaggtttgggggct
acgataccgcctgccattcagggaacgacggcgacaccgttgcaagatcttaatctgatcagatcagctc
tctggaaccggttgatttccgtggacatttagtgacagttgttgggccgattgtcggggttgaagccggc

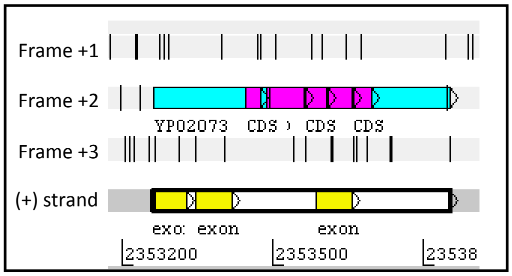

Peptide evidence:

FGGTVVNVK
GHLVTVVGPIVGVEAGK
IGMTPYNFVVIK
LEIASVPLDSGARPILGEPSQGR
TRLEIASVPLDSGARPILGEPSQGR
VIAYVDGFLEPVDFR

Current sequence (peptide evidence in red): 195aa
MTQSKKFRNHKWMYGWLGLGALLLSGCVTIPPAIQGTTATPLQDLNLIRSAPKLFIGQEARFGGTVVNVKNEPNRTRLEIASVPLDSGARPILGEPSQGRVIAYVDGFLEPVDFRGHLVTVVG
PIVGVEAGKIGMTPYNFVVIKATGYKRWHIAQQVIIPPTFGPWGYRPADMWGPGWPGWYNPGPAEIQTIVTD

Strain: Y. pseudotuberculosis PB1/+
Gene: YPTS_2117

Classification: annotated as Slp family outer membrane lipoprotein
Boundaries of gene: 2339996..2340601

Oligo evidence:

tacgtcaatgactcagtctaagaaatttagaaatcataaatggatgtatggttggctaggtttgggggct
acgataccgcctgccattcagggaacgacggcgacaccgttgcaagatcttaatctgatcagatcagctc
tctggaaccggttgatttccgtggacatttagtgacagttgttgggccgattgtcggggttgaagccggc

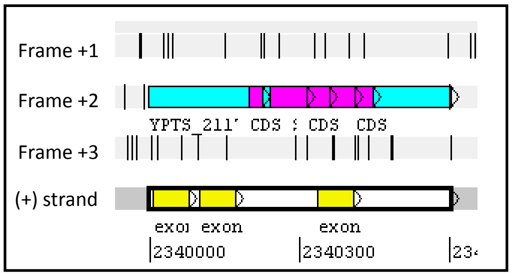

Peptide evidence:

FGGTVVNVK
GHLVTVVGPIVGVEAGK
IGMTPYNFVVIK
TRLEIASVPLDSGARPILGEPSQGR
VIAYVDGFLEPVDFR

Current sequence (peptide evidence in red): 201aa
MAMNTSMTQSKKFRNHKWMYGWLGLGALLLSGCVTIPPAIQGTTATPLQDLNLIRSAPKLFIGQEARFGGTVVNVKNEPNRTRLEIASVPLDSGARPILGEPSQGRVIAYVDGFLEPVDFRG
HLVTVVGPIVGVEAGKIGMTPYNFVVIKATGYKRWHIAQQVIIPPTFGPWGYRPADMWGPGWPGWYNPGPAEIQTIVTD

Error 57

Strain: Y. pestis pestoides F
Gene: YPDSF_1279 / YPDSF_12780

Classification: expressed pseudogene
Boundaries of gene: Frame-3: complement (1444935..1445204)
Frame-2: complement (1444409..1444939)

Oligo evidence:

aataaacatgacagtcggagtctttatgagcttttcaattgtacgtcgccgcattgttctgggcatggtg
gccaacgcattggctgaacacatgggggtcaaagctaaaatcacgccaaccaaatgggatgctggcctcg
tattcaagtactgaccaaaaaaggtaacgaaggccagtttactaagccagaagatttacagggtaagaag

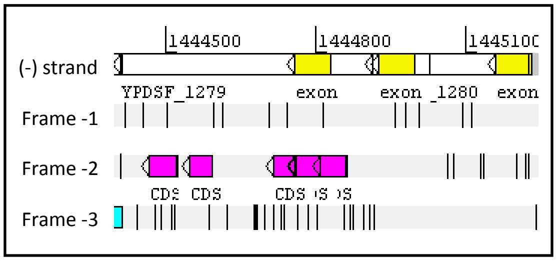

Peptide evidence:

KGNEGQFTKPEDLQGK
KGNEGQFTKPEDLQGKK
KNNPELLAAVNNAIAEMQK
KTNDALAVAGPAFAR
KVGVGLGSNYEQWLR
KYDFSTPYTVSGIQVLTK
NNPELLAAVNNAIAEMQK
VGVGLGSNYEQWLR
YDFSTPYTVSGIQVLTK

Proposed sequence (peptide evidence in red):
Frame-3: 90aa
MSFSIVRRRIVLGMVAVALATGLNVKSYAAGDLLEQVKQRGTLIVGLEGTYPPFSFQGEDGKLTGFEVDFANALAEHMGVKAKITPTKWD

Frame-2: 176aa
GMLASLESKRIDVAINQVTISDERKKKYDFSTPYTVSGIQVLTKKGNEGQFTKPEDLQGKKVGVGLGSNYEQWLRENLKDVDIRTYDDDPTKYQDLRVGRTDAILVDRLAALDLVKKTNDALAVAGPAFARQESGVAMRKNNPELLAAVNNAIAEMQKDGTLAKISEKYFGADVTQ

Strain: Y. pestis CO92
Gene: YPO1846

Classification: annotated as fliY
Boundaries of gene: 2090975..2091775

Oligo evidence:

aataaacatgacagtcggagtctttatgagcttttcaattgtacgtcgccgcattgttctgggcatggtg
tattcaagtactgaccaaaaaaggtaacgaaggccagtttactaagccagaagatttacagggtaagaag

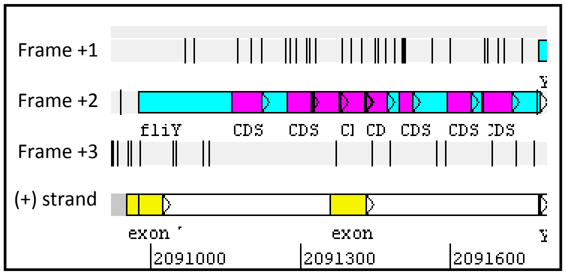

Peptide evidence:

KGNEGQFTKPEDLQGK
KGNEGQFTKPEDLQGKK
KNNPELLAAVNNAIAEMQK
KTNDALAVAGPAFAR
KYDFSTPYTVSGIQVLTK
LTGFEVDFANALAEHMGVK
NNPELLAAVNNAIAEMQK
RIDVAINQVTISDER
TYDDDPTK
VGVGLGSNYEQWLR
YDFSTPYTVSGIQVLTK

Current sequence (peptide evidence in red): 266aa
MSFSIVRRRIVLGMVAVALATGLNVKSYAAGDLLEQVKQRGTLIVGLEGTYPPFSFQGEDGKLTGFEVDFANALAEHMGVKAKITPTKWDGMLASLESKRIDVAINQVTISDERKKKYDFSTPYTVSGIQVLTKKGNEGQFTKPEDLQGKKVGVGLGSNYEQWLRENLKDVDIRTYDDDPTKYQDLRVGRTDAILVDRLAALDLVKKTNDALAVAGPAFARQESGVAMRKNNPELLAAVNNAIAEMQKDGTLAKISEKYFGADVTQ

Strain: Y. pseudotuberculosis PB1/+
Gene: YPTS_1847

Classification: annotated as cystine transporter subunit
Boundaries of gene: 2076110..2076910

Oligo evidence:

aataaacatgacagtcggagtctttatgagcttttcaattgtacgtcgccgcattgttctgggcatggtg
tattcaagtactgaccaaaaaaggtaacgaaggccagtttactaagccagaagatttacagggtaagaag

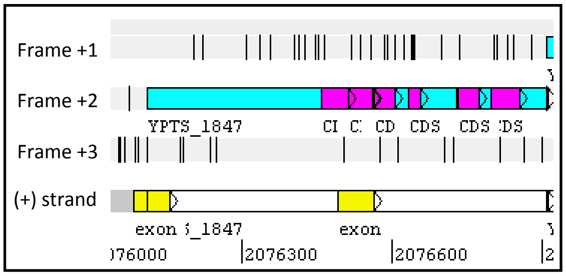

Peptide evidence:

KGNEGQFTKPEDLQGK
KGNEGQFTKPEDLQGKK
KNNPELLAAVNNAIAEMQK
KTNDALAVAGPAFAR
KVGVGLGSNYEQWLR
KYDFSTPYTVSGIQVLTK
TNDALAVAGPAFAR
TYDDDPTK
VGVGLGSNYEQWLR

Current sequence (peptide evidence in red): 266aa
MSFSIVRRRIVLGMVAVALATGLNVKSYAAGDLLEQVKQRGTLIVGLEGTYPPFSFQGEDGKLTGFEVDFANALAEHMGVKAKITPTKWDGMLASLESKRIDVAINQVTISDERKKKYDFSTPYTVSGIQVLTKKGNEGQFTKPEDLQGKKVGVGLGSNYEQWLRENLKDVDIRTYDDDPTKYQDLRVGRTDAILVDRLAALDLVKKTNDALAVAGPAFARQESGVAMRKNNPELLAAVNNAIAEMQKDGTLAKISEKYFGADVTQ

Error 58

Strain: Y. pestis pestoides F
Gene: YPDSF_1364 / YPDSF_1365

Classification: expressed pseudogene
Boundaries of gene: Frame-3: complement (1530987..1531292)
Frame-1: complement (1529950..1530987)
Frame-2:complement (1529726..1529950)

Oligo evidence:

tatttattgccattttggcagataaactgccgcctaaacaaagggataaagcccgcattatcggtttatc
aaattcgcgagcagttgttagataccccacacagtttgttccctgtttgccgcgactctttggatcaaat

Peptide evidence:

AKDLLVAIER
DSLDQIIGVVR

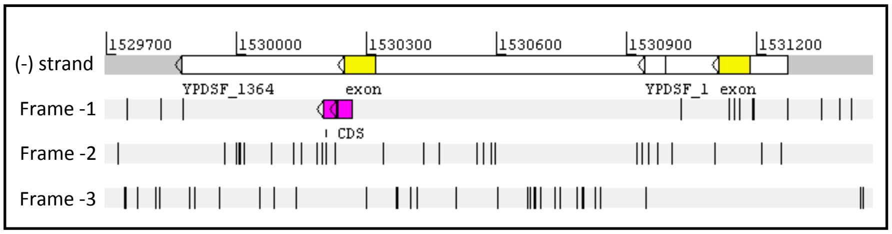

Proposed sequence (peptide evidence in red):
Frame-3: 102aa
MFLFSESDMELLMDPSIWAGLLTLVVLEIVLGIDNLVFIAILADKLPPKQRDKARIIGLSLALIMRLGLLSVISWMVTLTTPLFSVGAFNFAGRDLILLVGG

Frame-1: 347aa
MFLLFKATTELHERLEGNQHNDGANRGYASFWAVVVQIVILDAVFSLDAVITAVGMVNDLPIMMTAVVIAMGVMLLASKSLTRFVNEHPTVVVLCLSFLLMIGLSLIAEGFGFHIPKGYLYAAIGFSILIELFNQIARRNFIKHESRLPRRQRTAEAIIRLMGGRQQQEQQSGDPQQAVPTEAFAEEERYMISGVLTLASRSLRSVMTPRTEISWVDCNRSQAEIREQLLDTPHSLFPVCRDSLDQIIGVVRAKDLLVAIERGESICDFAATTPPIVVPDTMDVINLLGVLRKAKGRLVVVNDEFGVVQGLVTPLDVLEAIAGEFPDEDETPDIIADGDGWLVKGG

Frame-2: 74aa
ADLHSLEQALDCQTLVSPTADYASLAGLLLSHSGHMPTAGDVIELHNLRFQIMDVSDYRIELVRITKLSNELEE

Strain: Y. pestis CO92
Gene: YPO1759

Classification: annotated as putative membrane protein yoaE
Boundaries of gene: 2004523..2005917

Oligo evidence:

ggtggtattagagattgttctgggcattgatcatgcgtttggggctgttgtcggtcatttcatggatggt
aaattcgcgagcagttgttagataccccacacagtttgttccctgtttgccgcgactctttggatcaaat

Peptide evidence:

NA

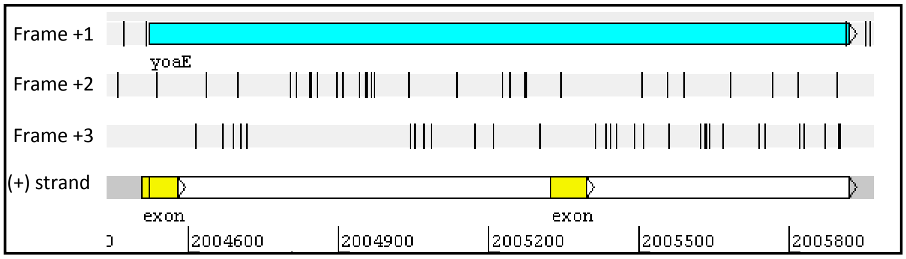

Current sequence (peptide evidence in red): 464aa
LFWALIMRLGLLSVISWMVTLTTPLFSVGAFNFAGRDLILLVGGLFLLFKATTELHERLEGNQHNDGANRGYASFWAVVVQIVILDAVFSLDAVITAVGMVNDLPIMMTAVVIAMGVMLLASKSLTRFVNEHPTVVVLCLSFLLMIGLSLIAEGFGFHIPKGYLYAAIGFSILIELFNQIARRNFIKHESRLPRRQRTAEAIIRLMGGRQQQEQQSGDPQQAVPTEAFAEEERYMISGVLTLASRSLRSVMTPRTEISWVDCNRSQAEIREQLLDTPHSLFPVCRDSLDQIIGVVRAKDLLVAIERGESICDFAATTPPIVVPDTMDVINLLGVLRKAKGRLVVVNDEFGVVQGLVTPLDVLEAIAGEFPDEDETPDIIADGDGWLVKGGADLHSLEQALDCQTLVSPTADYASLAGLLLSHSGHMPTAGDVIELHNLRFQIMDVSDYRIELVRITKLSNELEE

Strain: Y. pseudotuberculosis PB1/+
Gene: YPTS_1759

Classification: annotated as integral membrane protein TerC
Boundaries of gene: 1986376..1987920

Oligo evidence:

tatttattgccattttggcagataaactgccgcctaaacaaagggataaagcccgcattatcggtttatc
aaattcgcgagcagttgttagataccccacacagtttgttccctgtttgccgcgactctttggatcaaat

Peptide evidence:

DSLDQIIGVVR

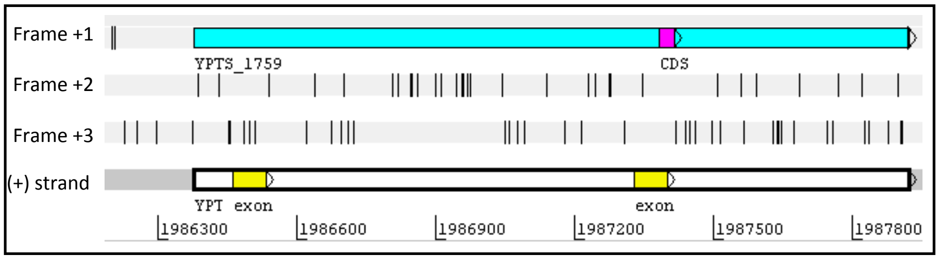

Current sequence (peptide evidence in red): 514aa
MELLMDPSIWAGLLTLVVLEIVLGIDNLVFIAILADKLPPKQRDKARIIGLSLALIMRLGLLSVISWMVTLTTPLFSVGAFNFAGRDLILLVGGLFLLFKATTELHERLEGNQHNDGANRGYASFWAVVVQIVILDAVFSLDAVITAVGMVNDLPIMMTAVVIAMGVMLLASKSLTRFVNEHPTVVVLCLSFLLMIGLSLIAEGFGFHIPKGYLYAAIGFSILIELFNQIARRNFIKHESRLPRRQRTAEAIIRLMGGRQQQEQQSGDPQQAVPTEAFAEEERYMISGVLTLASRSLRSVMTPRTEISWVDCNRSQAEIREQLLDTPHSLFPVCRDSLDQIIGVVRAKDLLVAIERGESICDFAATTPPIVVPDTMDVINLLGVLRKAKGRLVVVNDEFGVVQGLVTPLDVLEAIAGEFPDEDETPDIIADGDGWLVKGGADLHSLEQALDCQTLVSPTADYASLAGLLLSHSGHMPTAGDVIELHNLRFQIMDVSDYRIELVRITKLSNELEE

Error 59

Strain: Y. pestis pestoides F
Gene: YPDSF_1421 / YPDSF_1422/ YPDSF_1423

Classification: expressed pseudogene
Boundaries of gene: Frame+1: 1596334..1597083
Frame+3: 1597083..1597907

Oligo evidence:

atatttctatctctttctctgggtttcatgccttgcaagaagttgattttactctagaagggggttcaac
cgcgtcgtgcgtcgcttgcaatctgagggcatcggcattgtctttatttcgcaccgtattcatgaactgc
aactggcccacctctctgggggaatcagcaaaaagtggcccacctctctgggggaatcagcaaaaagt
atcaggtgaattttcagaattagtcggtttatgtgatcgtatctgcgtattgtgggatggccgaattgtt

Peptide evidence:

STLMAILSGAHNHYR

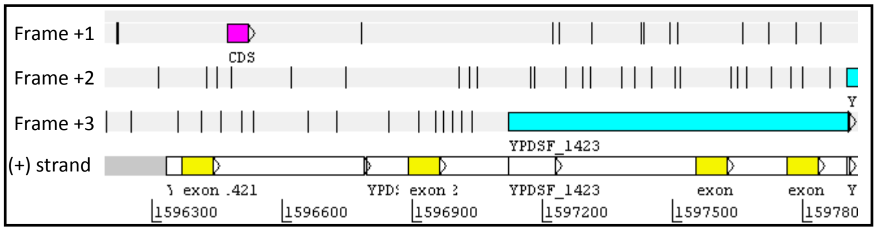

Proposed sequence (peptide evidence in red):

Frame+1: 250aa
MSSIPPSRLEMRNISISFSGFHALQEVDFTLEGGSTHALIGANGAGKSTLMAILSGAHNHYRGEIFIDGQPIDIHSPRQARQHGIHVVQQEVDVALIPTLSVAENIMLDELADRGHLFNWPQLY
RQAEALLEQLELKLNVRQRLESCTLA+KQQVLLARALSHQCRFLILDEPTAPLDQEESARLFRVVRRLQSEGIGIVFISHRIHELREVCDQLTVLRDGRRVSHDTMEGMSGEQIVEKMLGHTLED
I

Frame+3:274aa
FPPRRAAVSSKTRLSKTLLPKTLLSVSGLHDQYKLRDISLTLHQGEILGIAGLAGAGKTELCKALFGDTPSTLERGELSGKAWRPRSPDRSVAQGLALVPEERRKEGIFIDEGIPMNLSVAADDSFS
RWSLFSRRQELSWAKELIERLGIRTSSPQQKLAHLSGGNQQKVAHLSGGNQQKVAIGKWLRGDAQVLIFDEPTKGVDIKAKQDLFSLIDQLAQQGKGIIYASGEFSELVGLCDRICVLWDGRI
VAELNAAEVDEETLLLFSTGGTPQ

Strain: Y. pestis CO92
Gene: YPO1554

Classification: pseudo gene
Boundaries of gene: Frame-2: complement (1769183.. 1769932)
Frame-2: complement (1768392.. 1769183)

Oligo evidence:

atatttctatctctttctctgggtttcatgccttgcaagaagttgattttactctagaagggggttcaac
cgcgtcgtgcgtcgcttgcaatctgagggcatcggcattgtctttatttcgcaccgtattcatgaactgc
tcatccccacaacaaaaactggcccacctctctgggggaatcagcaaaaagtggccattggtaaatggc
atcaggtgaattttcagaattagtcggtttatgtgatcgtatctgcgtattgtgggatggccgaattgtt

Peptide evidence:

NA

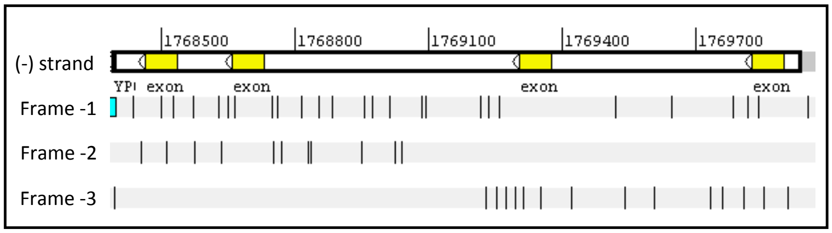

Current sequence (peptide evidence in red):
Frame-2: 250aa
MSSIPPSRLEMRNISISFSGFHALQEVDFTLEGGSTHALIGANGAGKSTLMAILSGAHNHYRGEIFIDGQPIDIHSPRQARQHGIHVVQQEVDVALIPTLSVAENIMLDELADRGHLFNWPQLY
RQAEALLEQLELKLNVRQRLESCTLAEKQQVLLARALSHQCRFLILDEPTAPLDQEESARLFRVVRRLQSEGIGIVFISHRIHELREVCDQLTVLRDGRRVSHDTMEGMSGEQIVEKMLGHTLED
I

Frame-3: 264aa
FPPRRAAVSSKTRLSKTLLPKTLLSVSGLHDQYKLRDISLTLHQGEILGIAGLAGAGKTELCKALFGDTPSTLERGELSGKAWRPRSPDRSVAQGLALVPEERRKEGIFIDEGIPMNLSVAADDSFS
RWSLFSRRQELSWAKELIERLGIRTSSPQQKLAHLSGGNQQKVAIGKWLRGDAQVLIFDEPTKGVDIKAKQDLFSLIDQLAQQGKGIIYASGEFSELVGLCDRICVLWDGRIVAELNAAEVDEE
TLLLFSTGGTPQ

Strain: Y. pseudotuberculosis PB1/+
Gene: YPTS_1677

Classification: annotated as ABC transporter-like protein
Boundaries of gene: 1885230..1886786

Oligo evidence:

cgcgtcgtgcgtcgcttgcaatctgagggcatcggcattgtctttatttcgcaccgtattcatgaactgc
atcaggtgaattttcagaattagtcggtttatgtgatcgtatctgcgtattgtgggatggccgaattgtt

Peptide evidence:

NA

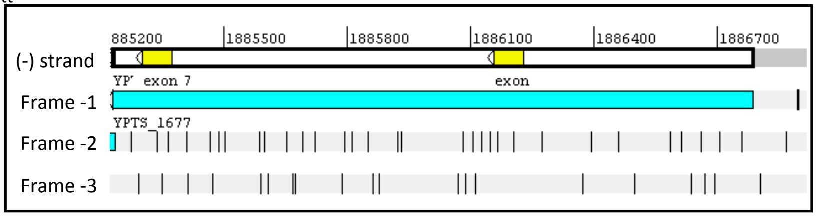

Current sequence (peptide evidence in red): 518aa
MSSIPPSRLEMRNISISFSGFHALQEVDFTLEGGSTHALIGANGAGKSTLMAILSGAHNHYRGEIFIDGQPIDIHSPRQARQHGIHVVQQEVDVALIPTLSVAENIMLDELADRGHLFNWPQLY
RQAEALLEQLELKLNVRQRLESCTLAEKQQVLLARALSHQCRFLILDEPTAPLDQEESARLFRVVRRLQSEGIGIVFISHRIHELREVCDQLTVLRDGRRVSHDTMEGMSGEQIVEKMLGHTLED
IFPPRRAAVSSKTLLSKTRLSKTLLPKTLLSVSGLHDQYKLRDISLTLHQGEILGIADLAGAGKTELCKALFGDTPSTLERGELSGKAWRPRSPDRSVAQGLALVPEERRKEGIFIDEGIPMNLSVAA
DDSFSRWSLFSRRQELSWAKELIERLGIRTSSPQQKLARLSGGNQQKVAIGKWLRGDAQVLIFDEPTKGVDIKAKQDLFSLIDQLAQQGKGIIYASGEFSELVGLCDRICVLWDGRIVAELNAAE
VDEETLLLFSTGGTPQ

Error 60

Strain: Y. pestis pestoides F
Gene: YPDSF_1980 /YPDSF_1981

Classification: expressed pseudogene
Boundaries of gene:Frame-1: complement(2238436..2238726)
Frame-3: complement(2238138..2238434)

Oligo evidence:

cagtgaagtcgatcaaatgctacttcaattcccccatcatggcgggttggcagcagattattatgcctga
ggacaagggtgttttgcaggttcttaccctacaaggtgatgaaattgtcgcccgattggcactaaaaaat

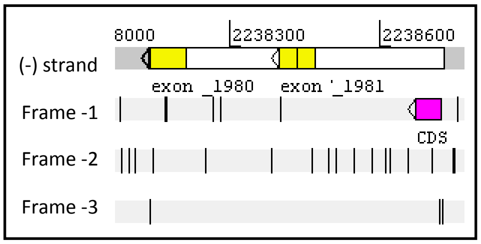

Peptide evidence:

LMFASDLHGSLPATER

Proposed sequence (peptide evidence in red):
Frame-1: 97aa
MKLMFASDLHGSLPATERVLAIFDHSGAQWLVLLGDLLNHGPRNALPEGYQPAAVAESLNAYKPAAVAESLNAYKDSIIAVRGNCDSEVDQMLLQFP

Frame-3: 98aa
IMAGWQQIIMPETRLFLTHGHLYHLGALPPLRHGDVLAYGHTHLPQAQWQGDIICFNPGSVSIPKGEYPASYGMLDKGVLQVLTLQGDEIVARLALKN

Strain: Y. pestis CO92
Gene: YPO2780

Classification: annotated as phosphodiesterase
Boundaries of gene: 3114471..3115046

Oligo evidence:

ggacaagggtgttttgcaggttcttaccctacaaggtgatgaaattgtcgcccgattggcactaaaaaat

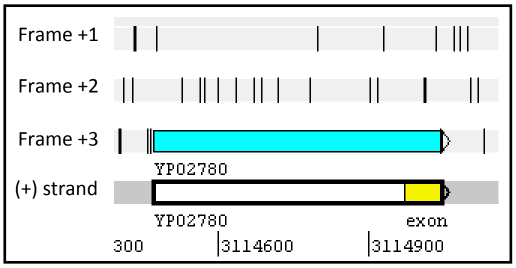

Peptide evidence:

NA

Current sequence (peptide evidence in red): 191aa
LLLTCMVRYRPRNVFWRSFDHSGAQWLVLLGDLLNHGPRNALPEGYQPAAVAESLNAYKPAAVAESLNAYKDSIIAVRGNCDSEVDQMLLQFPIMAGWQQIIMPETRLFLTHGHLYHPGA
LPPLRHGDVLAYGHTHLPQAQWQGDIICFNPGSVSIPKGEYPASYGMLDKGVLQVLTLQGDEIVARLALKN

Strain: Y. pseudotuberculosis PB1/+
Gene: YPTS_2698

Classification: annotated as phosphodiesterase
Boundaries of gene: 2997788..2998339

Oligo evidence:

ggacaagggtgttttgcaggttcttaccctacaaggtgatgaaattgtcgcccgattggcactaaaaaat

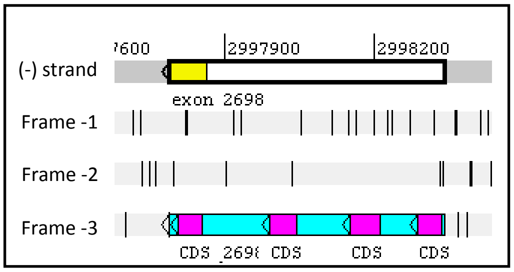

Peptide evidence:

GVLQVLTLQGDEIVAR
LFLTHGHLYHPGALPPLR
LMFASDLHGSLPATER
NALPEGYQPAAVAESLNAYK

Current sequence (peptide evidence in red): 183aa
MKLMFASDLHGSLPATERVLAIFDHSGAQWLVLLGDLLNHGPRNALPEGYQPAAVAESLNAYKDSIIAVRGNCDSEVDQMLLQFPIMAGWQQIIMPETRLFLTHGHLYHPGALPPLRHGD
VLAYGHTHLPQAQWQGDIICFNPGSVSIPKGEYPASYGMLDKGVLQVLTLQGDEIVARLALKN

Error 61

Strain: Y. pestis pestoides F
Gene: YPDSF_2565

Classification: expressed pseudogene
Boundaries of gene:Frame-1: complement(2913694..2914515)
Frame-3: complement(2913453..2913683)

Oligo evidence:

tttcaaaagcagatggaagtgggaacggatgtttgccaacaaattgcgcaaggtgaaaaatctatcatag
aaggtagccaaaacctggaaagtggtgaaccactaacctatggccagagtattaccgatgcctgtattgg

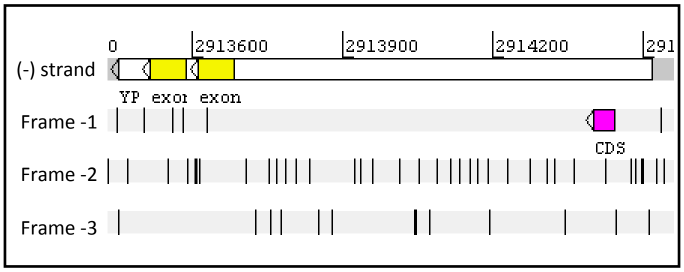

Peptide evidence:

FPSTVNAAETVAK

Proposed sequence (peptide evidence in red):
Frame-1: 274aa
MNYQNDDLRIKEIKELLPPVALLEKFPSTVNAAETVAKTRNAIHNILRAQDDRLLVVIGPCSIHDTQAAKEYAARLLALREELQGELEVVMRVYFEKPRTTVGWKGLINDPHMDNSYDINEGLR
LARELLVEINDSGLPAAGEFLDMITPQYLADLMSWGAIGARTTESQVHRELASGLSCPVGFKNGTDGTIKVAIDAINAASAPHCFLSVTKWGHSAIVNTAGNGDCHIILRGGKEPNYSSKHVN
AVKEGLIKAGLEPQIMIDFSHANSCKQ

Frame-3: 76aa
FQKQMEVGTDVCQQIAQGEKSIIGVMIESHLVEGSQNLESGEPLTYGQSITDACIGWKDTEALLRQLAGAVRARRQ

Strain: Y. pestis CO92
Gene: YPO1130

Classification: pseudogene
Boundaries of gene: Frame+3: 1277103..1280114

Oligo evidence:

ctggaaccccagattatgatcgatttcagccacgctaacagttgcaaacagtttcaaaagcagatggaag
tttcaaaagcagatggaagtgggaacggatgtttgccaacaaattgcgcaaggtgaaaaatctatcatag
agccaaaacctggaaagtggtgaaccactaacctattgtcaacgacggatgaaaagtgatccacttatat
tccacttatatctccaccaacggcccaatattgatccaccgttttactcaggattagcttctgctataac

Peptide evidence:
NA

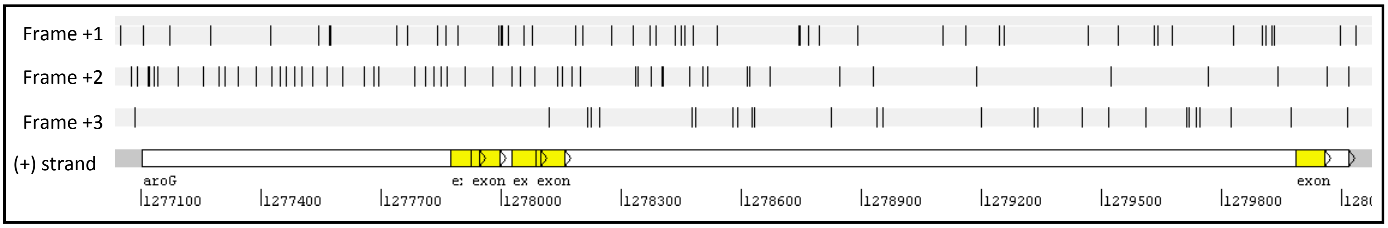

Current sequence (peptide evidence in red):
Frame+3: 338aa
MNYQNDDLRIKEIKELLPPVALLEKFPSTVNAAETVAKTRNAIHNILRAQDDRLLVVIGPCSIHDTQAAKEYAARLLALREELQGELEVVMRVYFEKPRTTVGWKGLINDPHMDNSYDINEGLR
LARELLVEINDSGLPAAGEFLDMITPQYLADLMSWGAIGARTTESQVHRELASGLSCPVGFKNGTDGTIKVAIDAINAASAPHCFLSVTKWGHSAIVNTAGNGDCHIILRGGKEPNYSSKHVN
AVKEGLIKAGLEPQIMIDFSHANSCKQFQKQMEVGTDVCQQIAQGEKSIIGVMIESHLVEGSQNLESGEPLTYCQRRMKSDPLISPPTAQY

Strain: Y. pseudotuberculosis PB1/+
Gene: YPTS_1243

Classification: annotated as phospho-2-dehydro-3-deoxyheptonate aldolase
Boundaries of gene: 1388767..1389819

Oligo evidence:

ctggaaccccagattatgatcgatttcagccacgctaacagttgcaaacagtttcaaaagcagatggaag
tttcaaaagcagatggaagtgggaacggatgtttgccaacaaattgcgcaaggtgaaaaatctatcatag
aaggtagccaaaacctggaaagtggtgaaccactaacctatggccagagtattaccgatgcctgtattgg

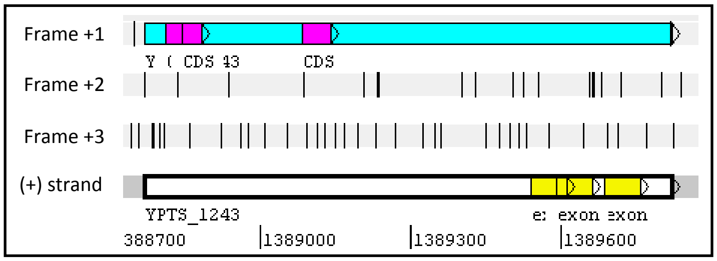

Peptide evidence:

ELLPPVALLEKFPSTVNAAETVAK
FPSTVNAAETVAK
GLINDPHMDNSYDINEGLR

Current sequence (peptide evidence in red): 350aa
MNYQNDDLRIKEIKELLPPVALLEKFPSTVNAAETVAKTRNAIHNILRAQDDRLLVVIGPCSIHDTQAAKEYAARLLALREELQGELEVVMRVYFEKPRTTVGWKGLINDPHMDNSYDINEGLR
LARELLVEINDSGLPAAGEFLDMITPQYLADLMSWGAIGARTTESQVHRELASGLSCPVGFKNGTDGTIKVAIDAINAASAPHCFLSVTKWGHSAIVNTAGNGDCHIILRGGKEPNYSSKHVN
AVKEGLIKAGLEPQIMIDFSHANSCKQFQKQMEVGTDVCQQIAQGEKSIIGVMIESHLVEGSQNLESGEPLTYGQSITDACIGWKDTEALLRQLAGAVRARRQ

Error 62

Strain: Y. pestis pestoides F
Gene: YPDSF_3023/ YPDSF_3024

Classification: expressed pseudogene
Boundaries of gene: Frame-3: complement (3430740..3431348)
Frame-2: complement (3430565..3430735)

Oligo evidence:

aaagtgcttgaaatgataaaaccctatgttcttgctgggataacgaccgatgaaattgatcggctatgcc
tgccgttaccgaatcgggatacgacctgctgacgccctggcctgaaggcactggcgaatacgaagccatc

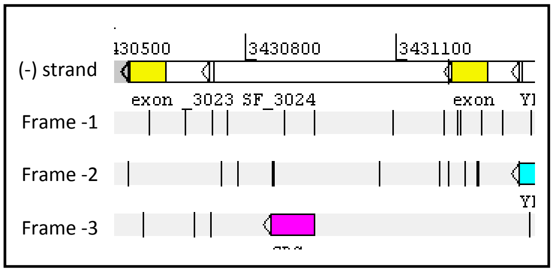

Peptide evidence:

AVRPGATLGDIGASIQQLAEGAGFSVVR

Proposed sequence (peptide evidence in red):
Frame-3: 203aa
MTEVKIHSPAEIEMARAAGQAAAKVLEMIKPYVLAGITTDEIDRLCHDYIVNELKVIPANIGYHGYTRTVCTSVNHVVCHGIPSDKKLKNGDIVNIDVAIIKEGWYGDTSRMYFVGEPSVRAKRLVDITYQSMVAGIKAVRPGATLGDIGASIQQLAEGAGFSVVREYCGHGVGQEYHTAPQVLHYGIAQTGMLLKPGMIFTI

Frame-2: 56aa
MINAGKAATSVLSDGWTVVTKDRSLSAQWEHTIAVTESGYDLLTPWPEGTGEYEAI

Strain: Y. pestis CO92
Gene: YPO3337

Classification: annotated as methionine aminopeptidase
Boundaries of gene: 3723448..3724233

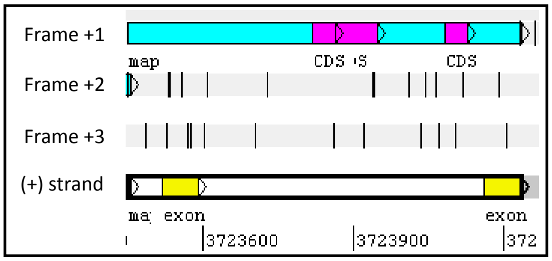

Oligo evidence:

aaagtgcttgaaatgataaaaccctatgttcttgctgggataacgaccgatgaaattgatcggctatgcc
tgccgttaccgaatcgggatacgacctgctgacgccctggcctgaaggcactggcgaatacgaagccatc

Peptide evidence:

AATSVLSDGWTVVTK
AVRPGATLGDIGASIQQLAEGAGFSVVR
RLVDITYQSMVAGIK

Current sequence (peptide evidence in red): 261aa
MTEVKIHSPAEIEMARAAGQAAAKVLEMIKPYVLAGITTDEIDRLCHDYIVNELKVIPANIGYHGYTRTVCTSVNHVVCHGIPSDKKLKNGDIVNIDVAIIKEGWYGDTSRMYFVGEPSVRAKRLVDITYQSMVAGIKAVRPGATLGDIGASIQQLAEGAGFSVVREYCGHGVGQEYHTAPQVLHYGIAQTGMLLKPGMIFTIEPMINAGKAATSVLSDGWTVVTKDRSLSAQWEHTIAVTESGYDLLTPWPEGTGEYEAI

Strain: Y. pseudotuberculosis PB1/+
Gene: YPTS_0829

Classification: annotated as methionine aminopeptidase, type I
Boundaries of gene: 933532:..34317

Oligo evidence:

aaagtgcttgaaatgataaaaccctatgttcttgctgggataacgaccgatgaaattgatcggctatgcc
tgccgttaccgaatcgggatacgacctgctgacgccctggcctgaaggcactggcgaatacgaagccatc

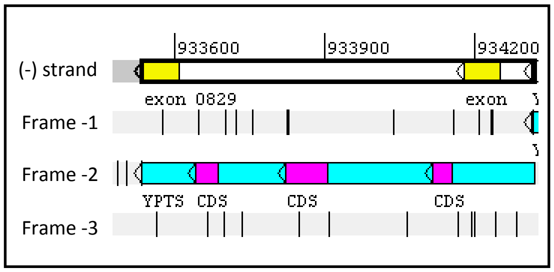

Peptide evidence:

AATSVLSDGWTVVTK
AVRPGATLGDIGASIQQLAEGAGFSVVR
VIPANIGYHGYTR

Current sequence (peptide evidence in red): 261aa
MTEVKIHSPAEIEMARAAGQAAAKVLEMIKPYVLAGITTDEIDRLCHDYIVNELKVIPANIGYHGYTRTVCTSVNHVVCHGIPSDKKLKNGDIVNIDVAIIKEGWYGDTSRMYFVGEPSVRAKRLVDITYQSMVAGIKAVRPGATLGDIGASIQQLAEGAGFSVVREYCGHGVGQEYHTAPQVLHYGIAQTGMLLKPGMIFTIEPMINAGKAATSVLSDGWTVVTKDRSLSAQWEHTIAVTESGYDLLTPWPEGTGEYEAI

Error 63

Strain: Y. pestis pestoides F
Gene: YPDSF_3360 /YPDSF_3361
Oligo evidence:

cagtatgtcgataatgtactgaacaaagcatcagatacttatcacggtgataaacctagcccattgctgg
tttatcgaattacactctgccacgtgatggttattacggcaaaaaaggatcggtactcaagccttataag

Classification: expressed pseudogene
Boundaries of gene: Frame-1: complement(3848809..3849825)
Frame-2: complement(3848108..3848809)

Peptide evidence:

HGEYGKPMGALWESTFEQQPPFFATK

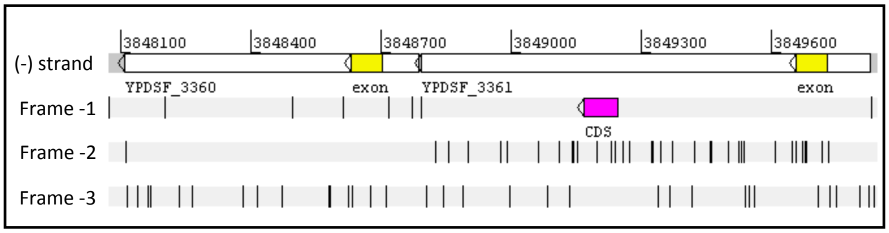

Proposed sequence (peptide evidence in red): 572aa

Frame-1: 339aa
MKKRALLLSMSVLAMLYIPAGQAAEIDRLTVVKQYVDNVLNKASDTYHGDKPSPLLADGVDPRTGQQMEWIFPDGRRAVLSNFSAQQNLMRVMSGLSELSGDPQYQKRAEDIVRYHFQN
YQDNSGLLYWGGHRFVDLKTLQPEGPSEKEKVHELKNAYPYYDLMFSVDSDATTRFIRGFWNAHVYDWRILETSRHGEYGKPMGALWESTFEQQPPFFATKGLSFLNAGNDLIYSASLLYKY
QQDQGALVWAKRLADQYVLPRDAKTGLGVYQFTQALKREEPTDDADTHSKFGDRAQRQFGPEFGPTALEGNMMLKGRTSTLYSENALMQLQLGKDLGG

Frame-2: 233aa
QGDDLLKWTVDGLKAFAKYGYNEQDNTFRPMIANGQDLSNYTLPRDGYYGKKGSVLKPYKAGNEFLISYARAYAVDNDPLLWKVARGIASDQGLGDIGSAPGKEMKVKLDTTNSDPYALFA
LLDLYNASQVAEYRSLAEKVADNIIKTRYIDGFFMASPDRQYADVDAIEPYALLALEASLRNKPQAVAPFLNGAGFTEGAYLMADGSARISTRDNELFLLNVGETLQPNGRK

Strain: Y. pestis CO92
Gene: YPO3994

Classification: annotated as periplasmic pectate lyase precursor
Boundaries of gene: 4500698..4502416

Oligo evidence:
cagtatgtcgataatgtactgaacaaagcatcagatacttatcacggtgataaacctagcccattgctgg
tttatcgaattacactctgccacgtgatggttattacggcaaaaaaggatcggtactcaagccttataag

Peptide evidence:

DTTNSDPYALFALLDLYNASQVAEYR

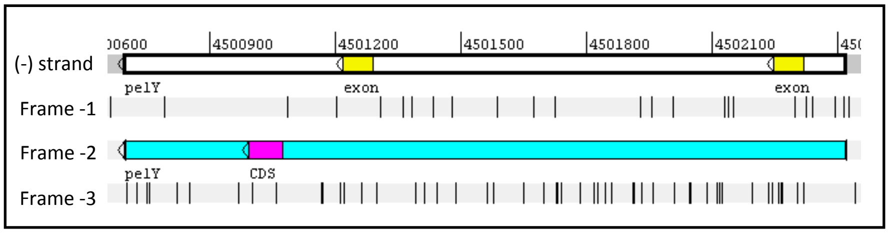

Current sequence (peptide evidence in red): 572aa
MKKRALLLSMSVLAMLYIPAGQAAEIDRLTVVKQYVDNVLNKASDTYHGDKPSPLLADGVDPRTGQQMEWIFPDGRRAVLSNFSAQQNLMRVMSGLSELSGDPQYQKRAEDIVRYHFQN
YQDNSGLLYWGGHRFVDLKTLQPEGPSEKEKVHELKNAYPYYDLMFSVDSDATTRFIRGFWNAHVYDWRILETSRHGEYGKPMGALWESTFEQQPPFFATKGLSFLNAGNDLIYSASLLYKY
QQDQGALVWAKRLADQYVLPRDAKTGLGVYQFTQALKREEPTDDADTHSKFGDRAQRQFGPEFGPTALEGNMMLKGRTSTLYSENALMQLQLGKDLGGQGDDLLKWTVDGLKAFAKY
GYNEQDNTFRPMIANGQDLSNYTLPRDGYYGKKGSVLKPYKAGNEFLISYARAYAVDNDPLLWKVARGIASDQGLGDIGSAPGKEMKVKLDTTNSDPYALFALLDLYNASQVAEYRSLAEKV
ADNIIKTRYIDGFFMASPDRQYADVDAIEPYALLALEASLRNKPQAVAPFLNGAGFTEGAYLMADGSARISTRDNELFLLNVGETLQPNGRK

Strain: Y. pseudotuberculosis PB1/+
Gene: YPTS_4050

Classification: annotated as periplasmic pectate lyase
Boundaries of gene: 4523321..4525039

Oligo evidence:
cagtatgtcgataatgtactgaacaaagcatcagatacttatcacggtgataaacctagcccattgctgg
tttatcgaattacactctgccacgtgatggttattacggcaaaaaaggatcggtactcaagccttataag

Peptide evidence:

NA

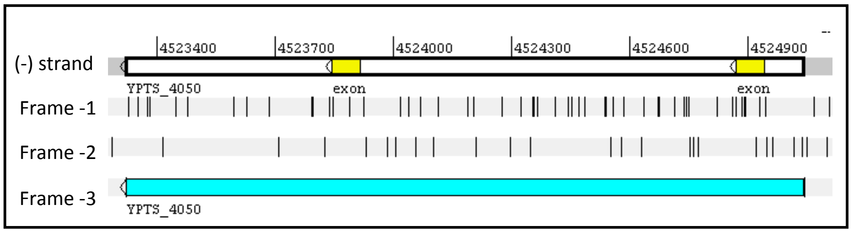

Current sequence (peptide evidence in red): 572aa
MKKRALLLSMSVLAMLYIPAGQAAEIDRLTVVKQYVDNVLNKASDTYHGDKPSPLLADGVDPRTGQQMEWIFPDGRRAVLSNFSAQQNLMRVMSGLSELSGDPQYQKRAEDIVRYHFQN
YQDNSGLLYWGGHRFVDLKTLQPEGPSEKEKVHELKNAYPYYDLMFSVDSDATTRFIRGFWNAHVYDWRILETSRHGEYGKPMGALWESTFEQQPPFFATKGLSFLNAGNDLIYSASLLYKY
QQDQGALVWAKRLADQYVLPRDAKTGLGVYQFTQALKREEPTDDADTHSKFGDRAQRQFGPEFGPTALEGNMMLKGRTSTLYSENALMQLQLGKDLGGQGDDLLKWTVDGLKAFAKY
GYNEQDNTFRPMIANGQDLSNYTLPRDGYYGKKGSVLKPYKAGNEFLISYARAYAVDNDPLLWKVARGIASDQGLGDIGSAPGKEMKVKLDTTNSDPYALFALLDLYNASQVAEYRSLAEKV
ADNIIKTRYIDGFFMASPDRQYADVDAIEPYALLALEASLRNKPQAVAPFLNGAGFTEGAYLMADGSARISTRDNELFLLNVGETLQPNGRK

Error 64

Strain: Y. pestis pestoides F
Gene: YPDSF_3740 / YPDSF_3741

Classification: expressed pseudogene/ frameshift
Boundaries of gene Frame+3:4298835..4299173
Frame+2: 4299179..4299652

Oligo evidence:

aaaccgctgaggaagctatttttgccgcacgtctggcccgcgaagccttgaatacccattgggtaaaact
ttgggtaaaactggaaattcatccagatgtgagatatttgctgcctgacccgatagaaacgctcaaggcc
aagtgctggtgaaagaggggtttgttgttttaccgaccccgtcttgtgtaaacgactggaagaagtgggt
tggctgggcttggcgcttcaccgttcaatccttcacaacctgatacattacaactacgggccaccgccac

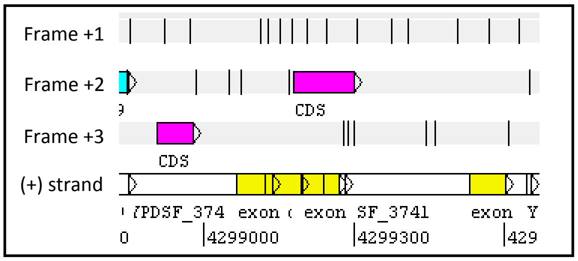

Peptide evidence:

FSSPELMLEALRASGSQLITMAMK
NAQGRRSAGERGVCCFTDPVLCKRLEEVGCAAVMPLGSP

Proposed sequence (peptide evidence in red):
Frame3: 109aa
MLKIADTTFTSRLFTGTGKFSSPELMLEALRASGSQLITMAMKRVDLQSGNDAILAPLRQLGVRLLPNTSGAKTAEEAIFAARLAREALNTHWVKLEIHPDVRYLLPD

Frame2: 156aa
NAQGRRSAGERGVCCFTDPVLCKRLEEVGCAAVMPLGSPIGSNLGLRTRDFLQIIIEQSKVPVVVDAGIGAPSHALEALELGADAVLVNTAIAVAHSPVQMAHAFRLAVESGERARLA
GLGASPFNPSQPDTLQLRATATSPLTGFLSQLEEQDHV

Strain: Y. pestis CO92
Gene: YPO3742

Classification: annotated as thiazole synthase ThiG
Boundaries of gene: 4194309..4195124

Oligo evidence:

aaaccgctgaggaagctatttttgccgcacgtctggcccgcgaagccttgaatacccattgggtaaaact
tggctgggcttggcgcttcaccgttcaatccttcacaacctgatacattacaactacgggccaccgccac

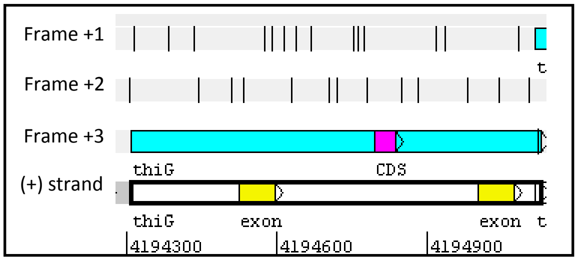

Peptide evidence:

TRDFLQIIIEQSK

Current sequence (peptide evidence in red): 271aa
MLKIADTTFTSRLFTGTGKFSSPELMLEALRASGSQLITMAMKRVDLQSGNDAILAPLRQLGVRLLPNTSGAKTAEEAIFAARLAREALNTHWVKLEVHPDVRYLLPDPIETLKAAEVLVKEGFV
VLPYCGADPVLCKRLEEVGCAAVMPLGSPIGSNLGLRTRDFLQIIIEQSKVPVVVDAGIGAPSHALEALELGADAVLVNTAIAVAHSPVQMAHAFRLAVESGERARLAGLGASPFNPSQPDTL
QLRATATSPLTGFLSQLEEQDHV

Strain: Y. pseudotuberculosis PB1/+
Gene: YPTS_0307

Classification: annotated as thiazole synthase
Boundaries of gene: complement (340282..341097)

Oligo evidence:

aaaccgctgaggaagctatttttgccgcacgtctggcccgcgaagccttgaatacccattgggtaaaact
ttgggtaaaactggaaattcatccagatgtgagatatttgctgcctgacccgatagaaacgctcaaggcc
tggctgggcttggcgcttcaccgttcaatccttcacaacctgatacattacaactacgggccaccgccac

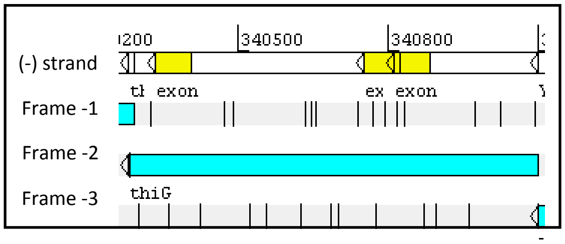

Peptide evidence:

NA

Current sequence (peptide evidence in red): 271aa
MLKIADTTFTSRLFTGTGKFSSPELMLEALRASGSQLITMAMKRVDLQSGNDAILAPLRQLGVRLLPNTSGAKTAEEAIFAARLAREALNTHWVKLEIHPDVRYLLPDPIETLKAAEVLVKEGFV
VLPYCGADPVLCKRLEEVGCAAVMPLGSPIGSNLGLRTRDFLQIIIEQSKVPVVVDAGIGAPSHALEALELGADAVLVNTAIAVAHSPVQMAHAFRLAVESGERARLAGLGASPFNPSQPDTL
QLRATATSPLTGFLSQLEEQDHV

Error 65

Strain: Y. pestis pestoides F
Gene: YPDSF_3742/YPDSF_3743

Classification: expressed pseudogene
Boundaries of gene:Frame3: 4299630..4300193
Frame1: 4300198..4300776

Oligo evidence:
ggcagaagatgaatatactgaattaaaggcgctgggattagatggcgtgatggtttatcaggataaccta

Peptide evidence:

DNVIPVAINNVSAGSKTQPGGYADDHPELEQF

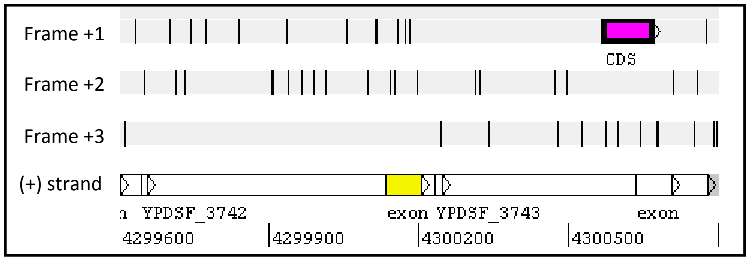

Proposed sequence (peptide evidence in red):
Frame3: 188aa
LRSKTMSEDFNQRWQQLDWDDISLTINSKKPADVERALNAIKPTREDLMALISPAALAYLEPMAQKAQQLTRQRFGNTVSFYVPLYLSNLCANDCTYCGFSMSNRIKRKTLDEAEIIRECEAIK
ALGFEHLLLVTGEHQTKVGMDYFRRHLPTIRSRFSSLMMEVQPLAEDEYTELKALGLDGVMVYQ

Frame1: 192aa
TYHPATYQQHHLRGHKQDFHWRLATPDRLGRAGIDKIGLGALIGLSNSWRTDCYMLAEHLFYLQQTYWQTRYSISFPRLRPCAGGIEPASIMSEPQLLQLICAFRLFAPDVELSLSTRESPFFR
DNVIPVAINNVSAGSKTQPGGYADDHPELEQFAPHDNRSPEQVAQALTKAGLQPVWKDWDSHLGRSLR

Strain: Y. pestis CO92
Gene: YPO3743

Classification: annotated as thiH
Boundaries of gene: 4195117..4196247

Oligo evidence:
tgccgatgatcatcccgaactggaacaatttgcgccccatgataaccgctccccggaacaggtcgcacaa

Peptide evidence:

DNVIPVAINNVSAGSKTQPGGYADDHPELEQF

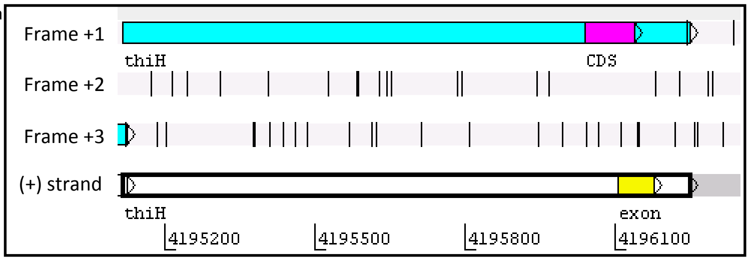

Current sequence (peptide evidence in red): 376aa
MSEDFNQRWQQLDWDDISLTINSKKPADVERALNAIKPTREDLMALISPAALAYLEPMAQKAQQLTRQRFGNTVSFYVPLYLSNLCANDCTYCGFSMSNRIKRKTLDEAEIIRECEAIKALGFE
HLLLVTGEHQTKVGMDYFRRHLPTIRSRFSSLMMEVQPLAEDEYTELKALGLDGVMVYQETYHPATYQQHHLRGHKQDFHWRLATPDRLGRAGIDKIGLGALIGLSNSWRTDCYMLAEHL
FYLQQTYWQTRYSISFPRLRPCAGGIEPASIMSEPQLLQLICAFRLFAPDVELSLSTRESPFFRDNVIPVAINNVSAGSKTQPGGYADDHPELEQFAPHDNRSPEQVAQALTKAGLQPVWKDW
DSHLGRSLR

Strain: Y. pseudotuberculosis PB1/+
Gene: YPTS_0306

Classification: annotated as thiamine biosynthesis protein ThiH
Boundaries of gene: complement (339159..340289)

Oligo evidence:
tgccgatgatcatcccgaactggaacaatttgcgccccatgataaccgctccccggaacaggtcgcacaa

Peptide evidence:

LRPCAGGIEPASIMSEPQLLQLICAF

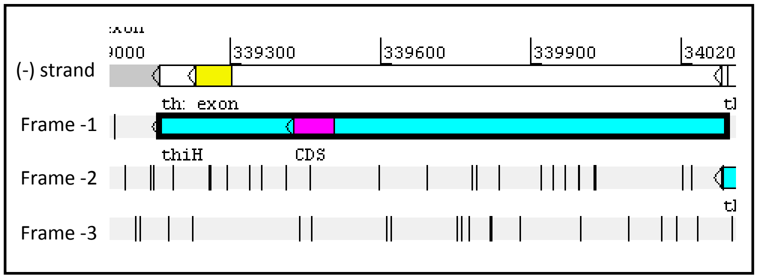

Current sequence (peptide evidence in red): 376aa
MSEDFNQRWQQLDWDDISLTINSKKPADVERALNAIKPTREDLMALISPAALAYLEPMAQKAQQLTRQRFGNTVSFYVPLYLSNLCANDCTYCGFSMSNRIKRKTLDEAEIIRECEAIKALGFE
HLLLVTGEHQTKVGMDYFRRHLPTIRSRFSSLMMEVQPLAEDEYTELKALGLDGVMVYQETYHPATYQQHHLRGHKQDFHWRLATPDRLGRAGIDKIGLGALIGLSNSWRTDCYMLAEHL
FYLQQTYWQTRYSISFPRLRPCAGGIEPASIMSEPQLLQLICAFRLFAPDVELSLSTRESPFFRDNVIPVAINNVSAGSKTQPGGYADDHPELEQFAPHDNRSPEQVAQALTKAGLQPVWKDW
DSHLGRSLR

Errors 66/67

Strain: Y. pestis pestoides F
Gene: YPDSF_3897/ YPDSF_3896

Classification: expressed pseudogenes
Boundaries of gene: complement (4474104..4475030)

Oligo evidence:

ttaagggggcaactaccggtgtggcattgatcttcgtgaatagcgatggcgaaaatgtgattggtattaa
aggcattgctaccgtgatcatcaccttaggtagccgtggcgtatggctcagtgaacaaggtaaaggtaaa

Peptide evidence:

QQLTADKIDTQPIEAIK
AAQILHDK
FAHAAAAIAVTRPGAQPSIPWR

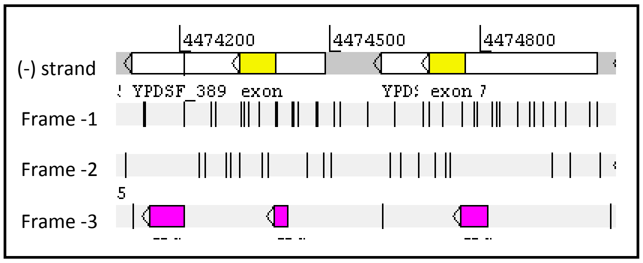

Proposed sequence (peptide evidence in red): 308aa
METGKLVVLGSINADHILNIEQFPRPGETVIGQQYNVAFGGKGANQAVAAGRSGADIAFIACVGDDDIGERVRQQLTADKIDTQPIEAIKGATTGVALIFVNSDGENVIGINAGANSAVTPEYL
RRYQQQVIDADALLMQLE+PLDTVIAAAKLAKQHQTQVILNPAPARKLPDELLTLVDMITPNETEAERLTGIHIEQDDDAAKAAQILHDKGIATVIITLGSRGVWLSEQGKGKLVAGFKVNAV
DTIAAGDTFNGALLTALLEGQSMDVAVRFAHAAAAIAVTRPGAQPSIPWRAEIDSFLQERV

Strain: Y. pestis CO92
Gene: YPO0008

Classification: annotated as rbsK
Boundaries of gene: 8366..9292

Oligo evidence:

ttaagggggcaactaccggtgtggcattgatcttcgtgaatagcgatggcgaaaatgtgattggtattaa
aggcattgctaccgtgatcatcaccttaggtagccgtggcgtatggctcagtgaacaaggtaaaggtaaa

Peptide evidence:

FAHAAAAIAVTRPGAQPSIPWR
GANQAVAAGR
KLPDELLTLVDMITPNETEAER
QHQTQVILNPAPAR
YQQQVIDADALLMQLESPLDTVIAAAK

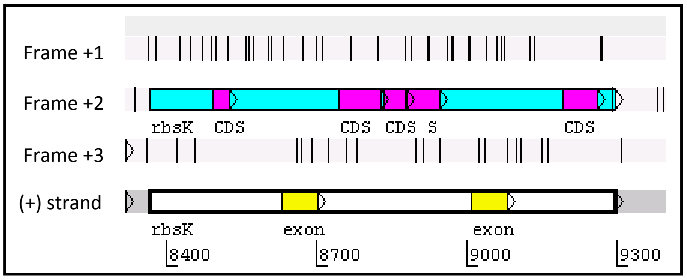

Current sequence (peptide evidence in red): 308aa
METGKLVVLGSINADHILNIEQFPRPGETVIGQQYNVAFGGKGANQAVAAGRSGADIAFIACVGDDDIGERVRQQLTADKIDTQPIEAIKGATTGVALIFVNSDGENVIGINAGANSAVTPEYL
RRYQQQVIDADALLMQLESPLDTVIAAAKLAKQHQTQVILNPAPARKLPDELLTLVDMITPNETEAERLTGIHIEQDDDAAKAAQILHDKGIATVIITLGSRGVWLSEQGKGKLVAGFKVNAV
DTIAAGDTFNGALLTALLEGQSMDVAVRFAHAAAAIAVTRPGAQPSIPWRAEIDSFLQERV

Strain: Y. pseudotuberculosis PB1/+
Gene: YPTS_4163

Classification: annotated as ribokinase
Boundaries of gene: 4652302..4653228

Oligo evidence:

ttaagggggcaactaccggtgtggcattgatcttcgtgaatagcgatggcgaaaatgtgattggtattaa

Peptide evidence:

FAHAAAAIAVTRPGAQPSIPWR
KLPDELLTLVDMITPNETEAER
LTGIHIEQDDDAAK
QHQTQVILNPAPAR
QQLTADKIDTQPIEAIK
RYQQQVIDADALLMQLESPLDTVIAAAK
VNAVDTIAAGDTFNGALLTALLEGQSMDAAVR
YQQQVIDADALLMQLESPLDTVIAAAK

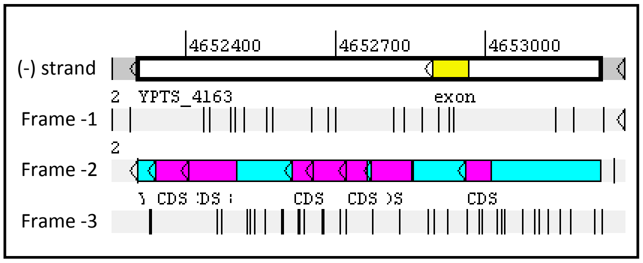

Current sequence (peptide evidence in red): 308aa
METGKLVVLGSINADHILNIEQFPRPGETVIGQQYNVAFGGKGANQAVAAGRSGADIAFIACVGDDDIGERVRQQLTADKIDTQPIEAIKGATTGVALIFVNSDGENVIGINAGANSAVTPEYL
RRYQQQVIDADALLMQLESPLDTVIAAAKLAKQHQTQVILNPAPARKLPDELLTLVDMITPNETEAERLTGIHIEQDDDAAKAAQILHDKGIATVIITLGSRGVWLSEQGKGKLVAGFKVNAV
DTIAAGDTFNGALLTALLEGQSMDAAVRFAHAAAAIAVTRPGAQPSIPWRAEIDSFLQERV

Error 68

Strain: Y. pestis pestoides F
Gene: YPDSF_0150.5

Classification: novel gene
Boundaries of gene: 156733..156909

Oligo evidence:

tagagcgtgaggatactcctgctgtgcgtggtatggtcaacttggtttcctacatggttaaagttgagga

Peptide evidence:

GMVNLVSYMVK
HKATLIGLGLR
IGHTVEREDTPAVR
RIGHTVER
RIGHTVEREDTPAVR
VTQTKSSIGR

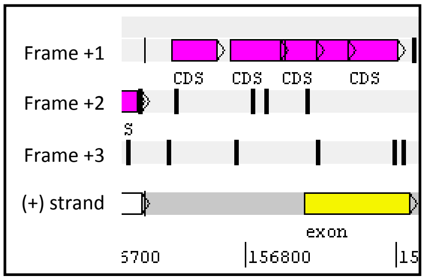

Proposed sequence (peptide evidence in red): 59aa
MAKTIKVTQTKSSIGRLPKHKATLIGLGLRRIGHTVEREDTPAVRGMVNLVSYMVKVEE

Strain: Y. pestis CO92
Gene: YPO0227

Classification: annotated as rpmD
Boundaries of gene: 230222..230401

Oligo evidence:

tagagcgtgaggatactcctgctgtgcgtggtatggtcaacttggtttcctacatggttaaagttgagga

Peptide evidence:

GMVNLVSYMVK
GMVNLVSYMVKVEE
HKATLIGLG
HKATLIGLGLR
IGHTVER
IGHTVEREDTPAVR
MVNLVSYMVK
RIGHTVER
RIGHTVEREDTPAVR

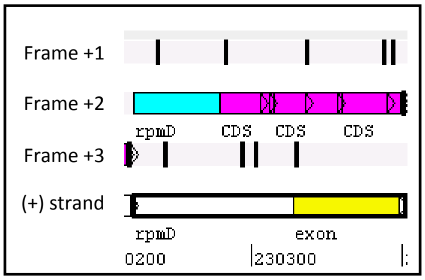

Current sequence (peptide evidence in red): 59aa
MAKTIKVTQTKSSIGRLPKHKATLIGLGLRRIGHTVEREDTPAVRGMVNLVSYMVKVEE

Strain: Y. pseudotuberculosis PB1/+
Gene: YPTS_3872

Classification: annotated as 50S ribosomal protein L30
Boundaries of gene: complement (4319871..4320050)

Oligo evidence:

tagagcgtgaggatactcctgctgtgcgtggtatggtcaacttggtttcctacatggttaaagttgagga

Peptide evidence:

GMVNLVSYMVK
GMVNLVSYMVKVEE
HKATLIGLGLR
IGHTVEREDTPAVR
RIGHTVER
RIGHTVEREDTPAVR

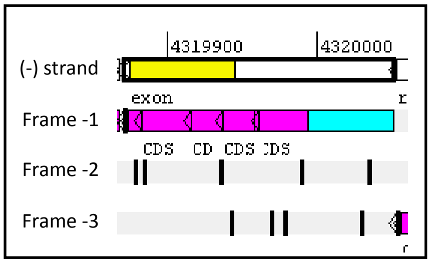

Current sequence (peptide evidence in red): 59aa
MAKTIKVTQTKSSIGRLPKHKATLIGLGLRRIGHTVEREDTPAVRGMVNLVSYMVKVEE

Error 70

Strain: Y. pestis pestoides F
Gene: YPDSF_1419.5

Classification: novel gene
Boundaries of gene: complement (1594488..1594703)

Oligo evidence:

ttacaccgctcttatttccccacttacgtatcccaggcagatacgaagtttccccgtcacttttaaccgc

Peptide evidence:

CETFANRVEGMGR

Proposed sequence (peptide evidence in red): 71aa
MGQLFILAPVPTGMRCTCSDYGAAGMANTRYTALISPLTYPRQIRSFPVTFNRCETFANRVEGMGRLNHGA

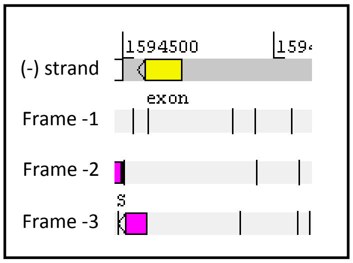

Error lacking threshold of evidence

Strain: Y. pestis CO92
Gene: YPO1556.5

Classification: novel gene
Boundaries of gene: 1771564..1771779

Oligo evidence:

ttacaccgctcttatttccccacttacgtatcccaggcagatacgaagtttccccgtcacttttaaccgc

Peptide evidence:

NA

Proposed sequence (peptide evidence in red): 71aa
MGQLFILAPVPTGMRCTCSDYGAAGMANTRYTALISPLTYPRQIRSFPVTFNRCETFANRVEGMGRLNHGA

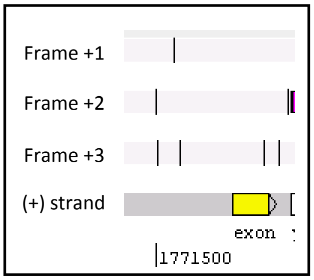

Strain: Y. pseudotuberculosis PB1/+
Gene: YPTS_1679

Classification: annotated as hypothetical protein
Boundaries of gene: 1888418..1888633

Oligo evidence:

ttacaccgctcttatttccccacttacgtatcccaggcagatacgaagtttccccgtcacttttaaccgc

Peptide evidence:

NA

Current sequence (peptide evidence in red): 71aa
MGQLFILAPVPTGMRCTCSDYGAAGMANTRYTALISPLTYPRQIRSFPVTFNRCETFANRVEGMGRLNHGA

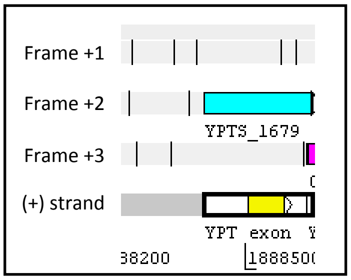

Error 71

Strain: Y. pestis pestoides F
Gene: YPDSF_1512.5

Classification: novel gene
Boundaries of gene: complement (1710507..1710755)

Oligo evidence:

gtactgagctaaaaaatattaaatctataaacggactgattgatctgtttgtaacgagcagttcagaatc

Peptide evidence:

HIVNSIDGAK
SINGLIDLFVTSSSES

Proposed sequence (peptide evidence in red): 82aa
MDRDHIASIVKKHIVNSIDGAKEHEIDLQKSMADYGASSLDIVSVVSGVMRELKIKIPRTELKNIKSINGLIDLFVTSSSES

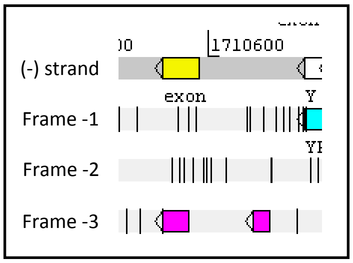

Strain: Y. pestis CO92
Gene: YPO1462

Classification: annotated as putative acyl carrier protein
Boundaries of gene: 1654210..1654458

Oligo evidence:

gtactgagctaaaaaatattaaatctataaacggactgattgatctgtttgtaacgagcagttcagaatc

Peptide evidence:

HIVNSIDGAKEHEIDLQK

Current sequence (peptide evidence in red): 82aa
MDRDHIASIVKKHIVNSIDGAKEHEIDLQKSMADYGASSLDIVSVVSGVMRELKIKIPRTELKNIKSINGLIDLFVTSSSES

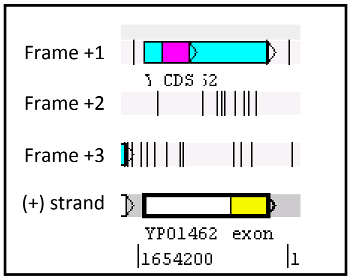

Strain: Y. pseudotuberculosis PB1/+
Gene: YPTS_1587

Classification: annotated as putative acyl carrier protein
Boundaries of gene: 1773856..1774104

Oligo evidence:

gtactgagctaaaaaatattaaatctataaacggactgattgatctgtttgtaacgagcagttcagaatc

Peptide evidence:

HIVNSIDGAKEHEIDLQK
MDRDHIASIVKK

Current sequence (peptide evidence in red): 82aa
MDRDHIASIVKKHIVNSIDGAKEHEIDLQKSMADYGASSLDIVSVVSGVMRELKIKIPRTELKNIKSINGLIDLFVTSSSES

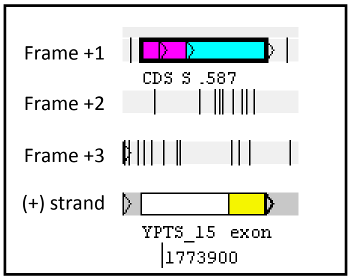

Error 72

Strain: Y. pestis pestoides F
Gene: YPDSF_1707.5

Classification: novel gene
Boundaries of gene: complement (1919272..1919697)

Oligo evidence:

tcaatttctgaacagggatataataatgaccaagagtctgtttaatacacttcaccgtttaacctcaact
ttcaccgtttaacctcaacttccacagcgaacgagttaacaaagcacactcctgagcaattacttgtctg
atttctcataaatttaagatagccgctgattctgagtatcagaatttccaacctgcagatgacttatatc

Peptide evidence:

LTFSPDNASFSWQ

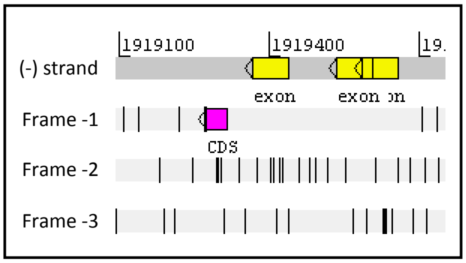

Proposed sequence (peptide evidence in red): 141aa
LLVKFKNRHAVAFNQFLNRDIIMTKSLFNTLHRLTSTSTANELTKHTPEQLLVWAQGCMLEGLPDQFYEAFIECIQCQTEDGKQRLDISHKFKIAADSEYQNFQPADDLYPAQCIEQALEGKQ
WSKARLTFSPDNASFSWQ

Strain: Y. pestis CO92
Gene: YPO0791

Classification: annotated as hypothetical protein
Boundaries of gene: complement(867363..867788)

Oligo evidence:

tcaatttctgaacagggatataataatgaccaagagtctgtttaatacacttcaccgtttaacctcaact
ttcaccgtttaacctcaacttccacagcgaacgagttaacaaagcacactcctgagcaattacttgtctg
atttctcataaatttaagatagccgctgattctgagtatcagaatttccaacctgcagatgacttatatc

Peptide evidence:

NA

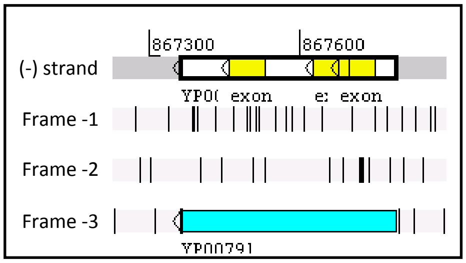

Current sequence (peptide evidence in red): 141aa
LLVKFKNRHAVAFNQFLNRDIIMTKSLFNTLHRLTSTSTANELTKHTPEQLLVWAQGCMLEGLPDQFYEAFIECIQCQTEDGKQRLDISHKFKIAADSEYQNFQPADDLYPAQCIEQALEGKQ
WSKARLTFSPDNASFSWQ

Error 98

Strain: Y. pseudotuberculosis PB1/+
Gene: YPTS_3161

Classification: upstream start site
Boundaries of gene: complement (3501896..3502321)

Oligo evidence:

tcaatttctgaacagggatataataatgaccaagagtctgtttaatacacttcaccgtttaacctcaact
ttcaccgtttaacctcaacttccacagcgaacgagttaacaaagcacactcctgagcaattacttgtctg
atttctcataaatttaagatagccgctgattctgagtatcagaatttccaacctgcagatgacttatatc

Peptide evidence:

LTSTSTANELTK

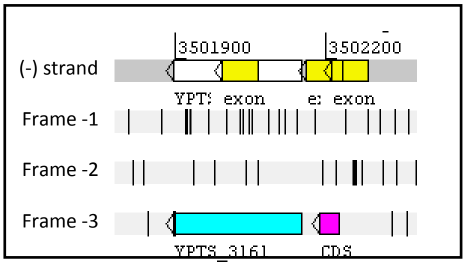

Proposed sequence (peptide evidence in red): 141aa
LLVKFKNRHAVAFNQFLNRDIIMTKSLFNTLHRLTSTSTANELTKHTPEQLLVWAQGCMLEGLPDQFYEAFIECIQCQTEDGKQRLDISHKFKIAADSEYQNFQPADDLYPAQCIEQALEGKQ
WSKARLTFSPDNASFSWQ

Error 74

Strain: Y. pestis pestoides F
Gene: YPDSF_2438.5

Classification: novel gene
Boundaries of gene: 2779318..2779680

Oligo evidence:

tttcttgcttcctgacacccatactgattacgccttcccagcttacatcaacaaacatattgatcttatt

Peptide evidence:

DISLYLASQNIK
HIDLIWR
ITVGYHSFLLPDTHTDYAFPAYINK

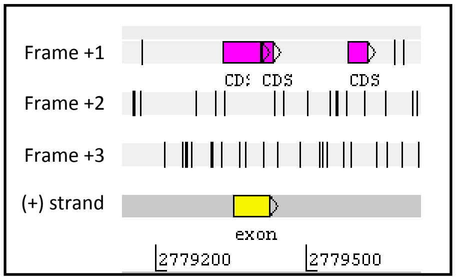

Proposed sequence (peptide evidence in red): 120aa
MTSAIKITVGYHSFLLPDTHTDYAFPAYINKHIDLIWRYIENNDKIEELSSNPFSKGRTAVLVKAKFLSSELKEFKLKTGIIGYPFDMKDISLYLASQNIKITLCTEFKRNGTLVNSLPS

Strain: Y. pestis CO92
Gene: YPO1255

Classification: annotated as hypothetical protein
Boundaries of gene: complement (1413073..1413522)

Oligo evidence:

tttcttgcttcctgacacccatactgattacgccttcccagcttacatcaacaaacatattgatcttatt

Peptide evidence:

AKFLSSELKEFK
ITVGYHSFLLPDTHTDYAFPAYINK

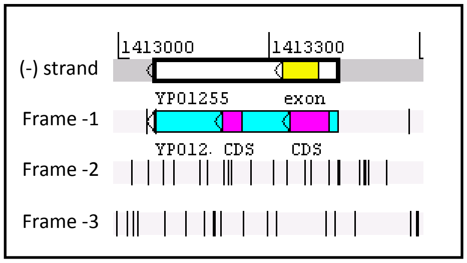

Current sequence (peptide evidence in red):120 aa
MTSAIKITVGYHSFLLPDTHTDYAFPAYINKHIDLIWRYIENNDKIEELSSNPFSKGRTAVLVKAKFLSSELKEFKLKTGIIGYPFDMKDISLYLASQNIKITLCTEFKRNGTLVNSLPS

Strain: Y. pseudotuberculosis PB1/+
Gene: YPTS_1381

Classification: annotated as hypothetical protein
Boundaries of gene: complement (1538397..1538801)

Oligo evidence:

tttcttgcttcctgacacccatactgattacgccttcccagcttacatcaacaaacatattgatcttatt

Peptide evidence:

AKFLSSELKEFK

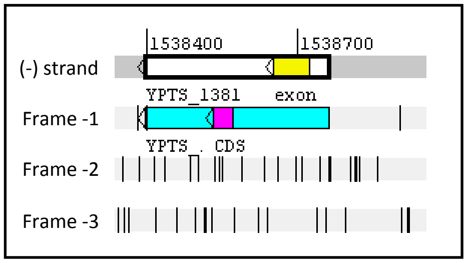

Current sequence (peptide evidence in red): 120aa
MTSAIKITVGYHSFLLPDTHTDYAFPAYINKHIDLIWRYIENNDKIEELSSNPFSKGRTAVLVKAKFLSSELKEFKLKTGIIGYPFDMKDISLYLASQNIKITLCTEFKRNGTLVNSLPS

Error 75

Strain: Y. pestis pestoides F
Gene: YPDSF_2615.5

Classification: novel gene
Boundaries of gene: complement (2961352..2961603)

Oligo evidence:

acaccaaatcagacaagtggggaaatggagcataaagcatctgctgcactcgataatccacgttccagtg

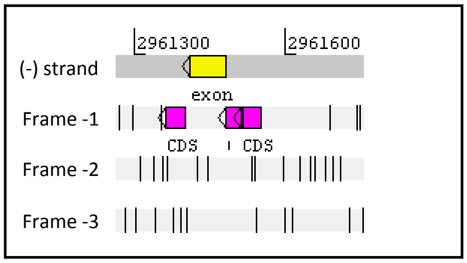

Peptide evidence:

QLAASVLAQANK
TLNDPNSSAIQR
SLAGSALSQR

Proposed sequence (peptide evidence in red): 83aa
MSKNTKQTSGKVASIAAKTLNDPNSSAIQRSLAGSALSQRGTPNQTSGEMEHKASAALDNPRSSALTKQLAASVLAQANKDRK

Error 41

Strain: Y. pestis CO92
Gene: YPO2638.5

Classification: novel gene
Boundaries of gene: 2967439..2967690

Oligo evidence:

acaccaaatcagacaagtggggaaatggagcataaagcatctgctgcactcgataatccacgttccagtg

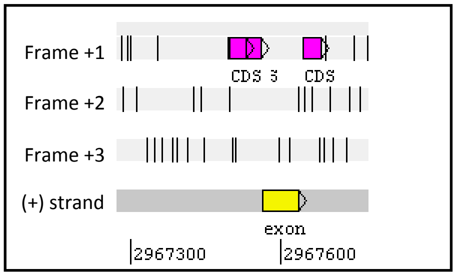

Peptide evidence:

LNDPNSSAIQR
QLAASVLAQANK
SLAGSALSQR
TLNDPNSSAIQR

Proposed sequence (peptide evidence in red): 83aa
MSKNTKQTSGKVASIAAKTLNDPNSSAIQRSLAGSALSQRGTPNQTSGEMEHKASAALDNPRSSALTKQLAASVLAQANKDRK

Strain: Y. pseudotuberculosis
Gene: YPTS_1189

Classification: annotated as hypothetical protein
Boundaries of gene: 1338362..1338613

Oligo evidence:

acaccaaatcagacaagtggggaaatggagcataaagcatctgctgcactcgataatccacgttccagtg

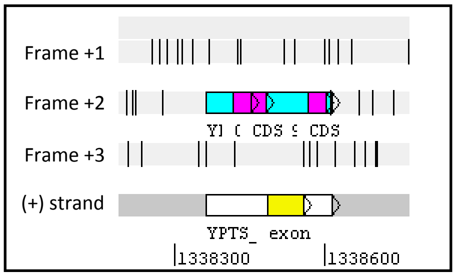

Peptide evidence:

QLAASVLAQANK
SLAGSALSQR
TLNDPNSSAIQR

Current sequence (peptide evidence in red): 83aa
MSKNTKQTSGKVASIAAKTLNDPNSSAIQRSLAGSALSQRGTPNQTSGEMEHKASAALDNPRSSALTKQLAASVLAQANKDRK

Error 76

Strain: Y. pestis pestoides F
Gene: YPDSF_2706.5

Classification: novel gene
Boundaries of gene: 3072771..3073109

Oligo evidence:

agaagcatgaagaatcgtaaagaacgtcccctcttgatggtcgttcgtaccggctgggaaaaagccgctg
cgtaccggctgggaaaaagccgctgcctatgaacaaattcgcccagtatctgaaatcgttattgagaaaa

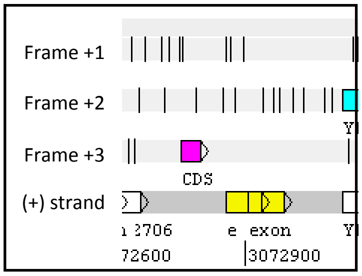

Peptide evidence:

MITHSPHTIASR

Proposed sequence (peptide evidence in red): 112aa
MITHSPHTIASRTECIGCFTVPGVLGGFEVRSMKNRKERPLLMVVRTGWEKAAAYEQIRPVSEIVIEKNGQSRPVNQAFFWNDLQDTSIDFSQKAVDLFFSKKGTLSACHPI

Strain: Y. pestis CO92
Gene: YPO2820

Classification: annotated as hypothetical protein
Boundaries of gene: 3149886..3150224

Oligo evidence:

agaagcatgaagaatcgtaaagaacgtcccctcttgatggtcgttcgtaccggctgggaaaaagccgctg
cgtaccggctgggaaaaagccgctgcctatgaacaaattcgcccagtatctgaaatcgttattgagaaaa

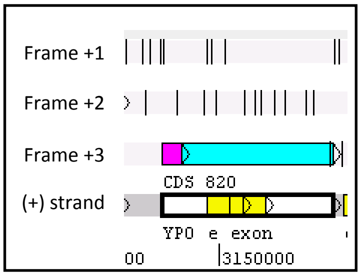

Peptide evidence:

MITHSPHTIASR

Current sequence (peptide evidence in red): 112aa
MITHSPHTIASRTECIGCFTVPGVLGGFEVRSMKNRKERPLLMVVRTGWEKAAAYEQIRPVSEIVIEKNGQSRPVNQAFFWNDLQDTSIDFSQKAVDLFFSKKGTLSACHPI

Error 101

Strain: Y. pseudotuberculosis PB1/+
Gene: YPTS_1089

Classification: **error**: annotated as hypothetical protein; upstream start site
Boundaries of gene: complement (1229284..1229619)

Oligo evidence:

agaagcatgaagaatcgtaaagaacgtcccctcttgatggtcgttcgtaccggctgggaaaaagccgctg
cgtaccggctgggaaaaagccgctgcctatgaacaaattcgcccagtatctgaaatcgttattgagaaaa

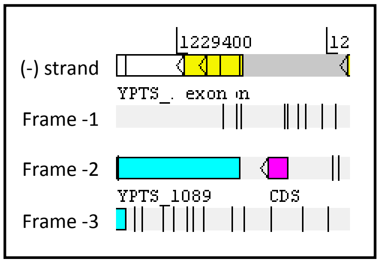

Peptide evidence:

MITHSPHTIASR

Proposed sequence (peptide evidence in red): 112aa (existing sequence underlined)
MITHSPHTIASRTECIGCFTVPGVLGGFEVRSMKNRKERPLLMVVRTGWEKAAAYEQIRPVSEIVIEKNGQSRPVNQAFFWNDLQDTSIDFSQKAVDLFFSKKGTLSACHPI

Error 78

Strain: Y. pestis pestoides F
Gene: YPDSF_3634.5

Classification: novel gene
Boundaries of gene:4178749..4179174

Oligo evidence:

attctcaacgtgtatctcggaaagttcgtgctttggcccggatagaaaaaaacataaatttaccgtgcat

Peptide evidence:

ALTANALVGFSTCISESSCFGPDR
ALTANALVGFSTCISESSCFGPDRK
APDVWTNNSFVR
DTSIWGASYNTLYDQAMYY
DTSIWGASYNTLYDQAMYYYTTGK
DTSIWGASYNTLYDQAMYYYTTGKR
EWTGDNVEGMHSGMIINK
FHSGQVDGK
FHSGQVDGKPYFCIEAFKPSTTITACSVK
VYYAPDVWTNN
VYYAPDVWTNNSFVR
YAPDVWTNNSFVR
YYAPDVWTNNSFVR

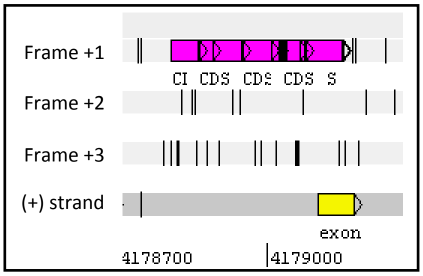

Proposed sequence (peptide evidence in red): 141aa
MRYLLSLSVFLIVSLNPAFAEWTGDNVEGMHSGMIINKFHSGQVDGKPYFCIEAFKPSTTITACSVKDTSIWGASYNTLYDQAMYYYTTGKRIRVYYAPDVWTNNSFVRALTANALVGFSTCIS
ESSCFGPDRKKHKFTVH

Strain: Y. pestis CO92
Gene: YPO0337

Classification: annotated as hypothetical protein
Boundaries of gene: complement (344915..345340)

Oligo evidence:

attctcaacgtgtatctcggaaagttcgtgctttggcccggatagaaaaaaacataaatttaccgtgcat

Peptide evidence:

ALTANALVGFSTCISESSCFGPDR
APDVWTNNSFVR
DTSIWGASYNTLYDQAMYYYTTGK
DTSIWGASYNTLYDQAMYYYTTGKR
EWTGDNVEGMHSGMIINK
FHSGQVDGK
IRVYYAPDVWTNNSFVR
VYYAPDVWTNN
VYYAPDVWTNNSFVR
WTGDNVEGMHSGMIINK
YAPDVWTNNSFVR
YYAPDVWTNNSFVR

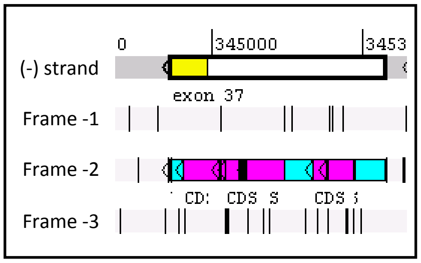

Current sequence (peptide evidence in red): 141aa
MRYLLSLSVFLIVSLNPAFAEWTGDNVEGMHSGMIINKFHSGQVDGKPYFCIEAFKPSTTITACSVKDTSIWGASYNTLYDQAMYYYTTGKRIRVYYAPDVWTNNSFVRALTANALVGFSTCIS
ESSCFGPDRKKHKFTVH

Strain: Y. pseudotuberculosis PB1/+
Gene: YPTS_0416

Classification: annotated as hypothetical protein
Boundaries of gene: complement (460869..461294)

Oligo evidence:

attctcaacgtgtatctcggaaagttcgtgctttggcccggatagaaaaaaacataaatttaccgtgcat

Peptide evidence:

ALTANALVGFSTCISESSCFGPDR
APDVWTNNSFVR
DTSIWGASYNTLYDQAMYYYTTGK
DTSIWGASYNTLYDQAMYYYTTGKR
EWTGDNVEGMHSGMIINK
FHSGQVDGK
VYYAPDVWTNN
VYYAPDVWTNNSFVR

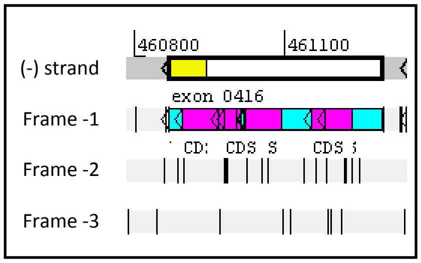

Current sequence (peptide evidence in red): 141aa
MRYLLSLSVFLIVSLNPAFAEWTGDNVEGMHSGMIINKFHSGQVDGKPYFCIEAFKPSTTITACSVKDTSIWGASYNTLYDQAMYYYTTGKRIRVYYAPDVWTNNSFVRALTANALVGFSTCIS
ESSCFGPDRKKHKFTVH

Error 96

Strain: Y. pseudotuberculosis PB1/+
Gene: YPTS_0106.5

Classification: novel gene
Boundaries of gene: 111739..111900

Oligo evidence:
tgataatatgcttttttatcgagatatacatggagggatggtatgtgagaatattcttatacaatatgtc

Peptide evidence:

KIIIDNMLFYR

Proposed sequence (peptide evidence in red): 53aa
VISNHLKTGYIPIIRSSKIIIDNMLFYRDIHGGMVCENILIQYVDIAKTILEL

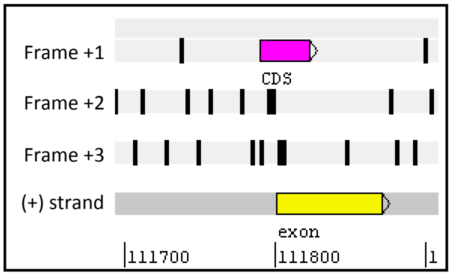

Error79

Strain: Y. pestis pestoides F
Gene: YPDSF_3794.5

Classification: novel gene
Boundaries of gene: complement (4363721..4363882)

Oligo evidence:
tgataatatgcttttttatcgagatatacatggagggatggtatgtgagaatattcttatacaatatgtc

Peptide evidence:

KIIIDNMLFYR

Proposed sequence (peptide evidence in red): 53aa
VISNHLKTGYIPIIRSSKIIIDNMLFYRDIHGGMVCENILIQYVDIAKTILEL

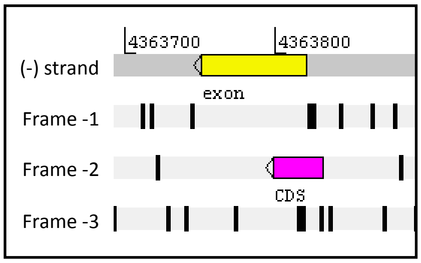

Strain: Y. pestis CO92
Gene: YPO0113

Classification: annotated as hypothetical protein
Boundaries of gene: 121254..121415

Oligo evidence:
tgataatatgcttttttatcgagatatacatggagggatggtatgtgagaatattcttatacaatatgtc

Peptide evidence:

NA

Current sequence (peptide evidence in red): 53aa
VISNHLKTGYIPIIRSSKIIIDNMLFYRDIHGGMVCENILIQYVDIAKTILEL

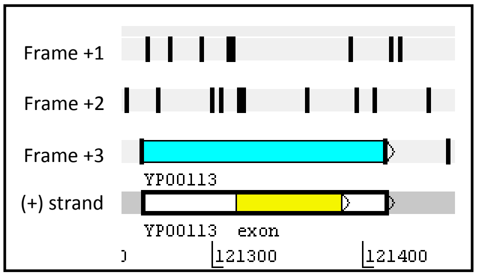

Error 80

Strain: Y. pestis pestoides F
Gene: YPDSF_3804.5

Classification: novel gene
Boundaries of gene: 4374258..4374419

Oligo evidence:

ccgatagcgtcatggcaacgatcaaagccactccagcggtagttgtactacggagaaaacagcagcctct

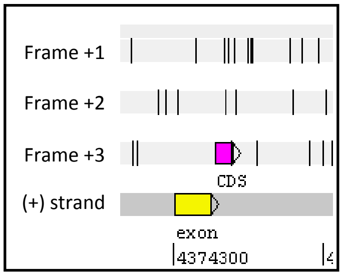

Peptide evidence:

KPHQDVNELSQ

Proposed sequence (peptide evidence in red): 53aa
VACSDFACSEIAHADTDSVMATIKATPAVVVLRRKQQPLCIKKPHQDVNELSQ

Error lacking threshold of evidence

Strain: Y. pestis CO92
Gene: YPO0101.5

Classification: novel gene
Boundaries of gene: complement (110692..110853)

Oligo evidence:

ccgatagcgtcatggcaacgatcaaagccactccagcggtagttgtactacggagaaaacagcagcctct

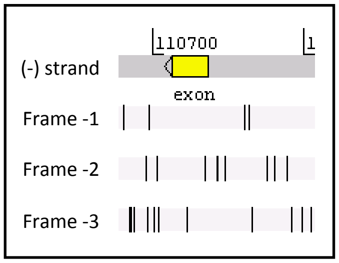

Peptide evidence:

NA

Proposed sequence (peptide evidence in red): 53aa
VACSDFACSEIAHADTDSVMATIKATPAVVVLRRKQQPLCIKKPHQDVNELSQ

Error lacking threshold of evidence

Strain: Y. pseudotuberculosis PB1/+
Gene: YPTS_0095.5

Classification: novel gene
Boundaries of gene: complement (101187..101348)

Oligo evidence:

tataaatttatggatatatgctgttttctacagtgagttttttgggttcgaactgtcacattatctttcc

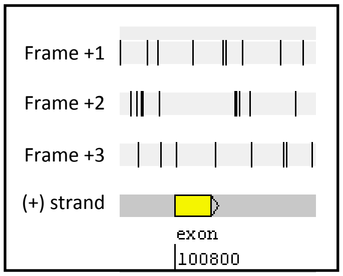

Peptide evidence:

NA

Proposed sequence (peptide evidence in red): 53aa
VACSDFACSEIAHADTDSVMATIKATPAVVVLRRKQQPLCIKKPHQDVNELSQ

Error 81

Strain: Y. pestis pestoides F
Gene: YPDSF_0215.5

Classification: novel gene
Boundaries of gene:240335..240820

Oligo evidence:

cgatggttgttataatgaaatgattagaaaaaatgtaatcccctcaaacataataccgtttggaaatgat

Peptide evidence:

DDDSVVFYATDSFDPEVSMSK
KLTSSFEEFINGLVEEDDLE
LTSSFEEFINGLVEEDDLE
TLGYSFPEAFVSHYLSFNGGVPLR
AWWACDDGCEPLEIAAFKPFK

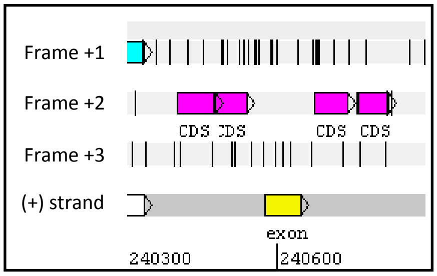

Proposed sequence (peptide evidence in red): 161aa
MKNPCQNCEKNIDLSDIKSVEKTLGYSFPEAFVSHYLSFNGGVPLRAWWACDDGCEPLEIAAFKPFKYHKMTNDNPNSLIDGCYNEMIRKNVIPSNIIPFGNDWGGNFFCLNKDDDSVVFYATDSFDPEVSMSKNHDVLQKKLTSSFEEFINGLVEEDDLE

Strain: Y. pestis CO92
Gene: YPO3610

Classification: annotated as hypothetical protein
Boundaries of gene: 4024505..4024990

Oligo evidence:

cgatggttgttataatgaaatgattagaaaaaatgtaatcccctcaaacataataccgtttggaaatgat

Peptide evidence:

DDDSVVFYATDSFDPEVSMSK
LTSSFEEFINGLVEEDDLE
TLGYSFPEAFVSHYLSFNGGVPLR

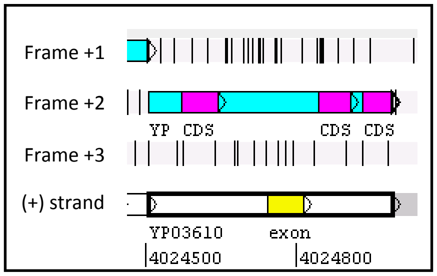

Current sequence (peptide evidence in red): 161aa
MKNPCQNCEKNIDLSDIKSVEKTLGYSFPEAFVSHYLSFNGGVPLRAWWACDDGCEPLEIAAFKPFKYHKMTNDNPNSLIDGCYNEMIRKNVIPSNIIPFGNDWGGNFFCLNKDDDSVVFYATDSFDPEVSMSKNHDVLQKKLTSSFEEFINGLVEEDDLE

Strain: Y. pseudotuberculosis PB1/+
Gene: no ortholog

Error 82

Strain: Y. pestis pestoides F
Gene: YPDSF_0388.5

Classification: novel gene
Boundaries of gene: 441966..442397

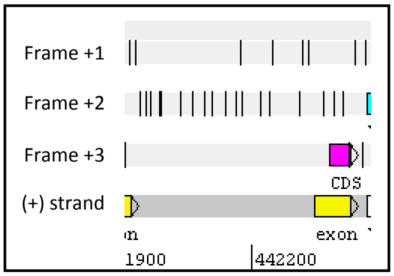

Oligo evidence:

ggaatcagaagcgcgacatgaaattcaaaaaattctggctgaatcgccttacgacgatagtctttataca

Peptide evidence:

ILAESPYDDSLYT

Proposed sequence (peptide evidence in red): 143aa
VRFADMLLSIAEIQKKVDEMALRAGLPRHSVNLCTEPIGEGTPYITFENNMYNYIYSERGYEFSRRVTKSLDELLYWIMSELAHKAAFQYELDHRVEGRDGRRIAFPKFIELMANMNSAWESE
ARHEIQKILAESPYDDSLYT

Strain: Y. pestis CO92
Gene: YPO0602

Classification: annotated as hypothetical protein
Boundaries of gene: 660010..660441

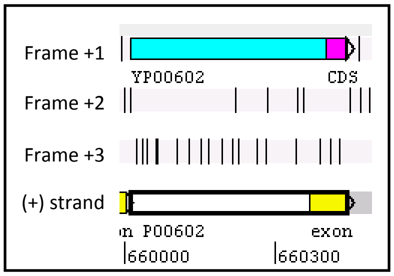

Oligo evidence:

ggaatcagaagcgcgacatgaaattcaaaaaattctggctgaatcgccttacgacgatagtctttataca

Peptide evidence:

ILAESPYDDSLYT

Current sequence (peptide evidence in red): 143aa
VRFADMLLSIAEIQKKVDEMALRAGLPRHSVNLCTEPIGEGTPYITFENNMYNYIYSERGYEFSRRVTKSLDELLYWIMSELAHKAAFQYELDHRVEGRDGRRIAFPKFIELMANMNSAWESE
ARHEIQKILAESPYDDSLYT

Strain: Y. pseudotuberculosis PB1/+
Gene: no ortholog

Error 83

Strain: Y. pestis pestoides F plasmid CD
Gene: YPDSF_4024

Classification: frameshift
Boundaries of gene: Frame+1: 68530..68769
Frame+3: 68769..69833

Oligo evidence:

atggtattcctcgtttgtcagcagttcaaataagcccaaatgttgatcctaaattgggtgtgggattata
agaaagaaatggctgaaacattggaaaatgcacgtaaagagactttggctcagtctaacgatgttttgga
ctaatcaatacacagatcataaattcagtcaacttgacaaccgtttagataaacttgacaaacgagttga

Peptide evidence:

LGVGLYPAKPILR
LPPRGPQGPEK
EMAETLENARK
ETLAQSNDVLDAAK
ETLAQSNDVLDAAKK
TDRENSVSIGHESLNR
KETLAQSNDVLDAAKK
SSQALAIGSGYR
TTLETAEEHANKK

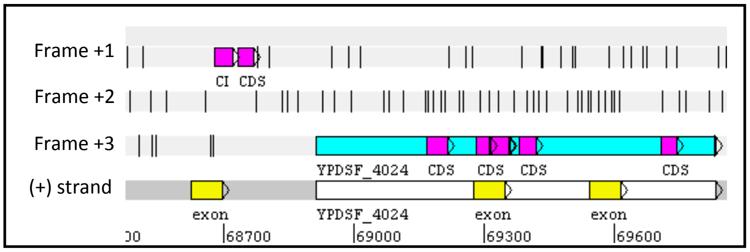

Proposed sequence (peptide evidence in red):
Frame+1: 79aa
MTKDFKISVSAALISALFSSPYAFAEEPEDGSDGIPRLSAVQISPNVDPKLGVGLYPAKPILRQENPKLPPRGPQGPEK

Frame+3: 356aa
RARLAEAIQPQVLGAGGLNARAKDPYSIAIGATAEAAKPAAIAVGSGSMATGVDSVAIGPLSKALGDSAVTYGVSSTAQKDGVAIGAKASASDTGVAVGFNSKVDAQNSVAIGHSSHVAAD
HGYSIAIGDHSKTDRENSVSIGHESLNRQLTHLAAGTEDTDAVNVAQLKKEMAETLENARKETLAQSNDVLDAAKKHSNSVARTTLETAEEHANKKSAETLVSAKVYADSNSSQTLKTANSYT
DVTVSNSTKKATRESNQYTDHKFSQLDNRLDKLDKRVDKGLASSAALNSLFQPYGVGKVNFTAGVGGYRSSQALAIGSGYRVNESVAFKAGVAYAGSSNVMYNASFNIEW

Strain: Y. pestis CO92
Gene: YPCD1.87c/ YPCD1.88c

Classification: YPCD1.88c
YPCD1.87c putative yadA yopA invasin pseudogene
Boundaries of gene: complement (60568..61632)

Oligo evidence:

agaaagaaatggctgaaacattggaaaatgcacgtaaagagactttggctcagtctaacgatgttttgga
ctaatcaatacacagatcataaattcagtcaacttgacaaccgtttagataaacttgacaaacgagttga

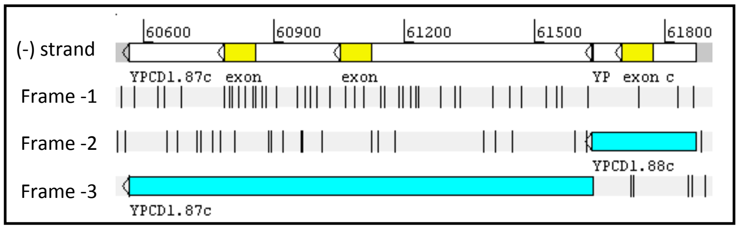

Peptide evidence:

NA

Current sequence (peptide evidence in red):
YPCD1.88c 79aa
MTKDFKISVSAALISALFSSPYAFAEEPEDGSDGIPRLSAVQISPNVDPKLGVGLYPAKPILRQENPKLPPRGPQGPEK

YPCD1.87c 355aa
KRARLAEAIQPQVLGAGGLNARAKDPYSIAIGATAEAAKPAAIAVGSGSMATGVDSVAIGPLSKALGDSAVTYGVSSTAQKDGVAIGAKASASDTGVAVGFNSKVDAQNSVAIGHSSHVAAD
HGYSIAIGDHSKTDRENSVSIGHESLNRQLTHLAAGTEDTDAVNVAQLKKEMAETLENARKETLAQSNDVLDAAKKHSNSVARTTLETAEEHANKKSAETLVSAKVYADSNSSQTLKTANSYT
DVTVSNSTKKATRESNQYTDHKFSQLDNRLDKLDKRVDKGLASSAALNSLFQPYGVGKVNFTAGVGGYRSSQALAIGSGYRVNESVAFKAGVAYAGSSNVMYNASFNIEW

Strain: Y. pseudotuberculosis PB1/+
Gene: YPTS_4238

Classification: annotated as YadA domain-containing protein
Boundaries of gene: complement(23493..24791)

Oligo evidence:

atggtattcctcgtttgtcagcagttcaaataagcccaaatgttgatcctaaattgggtgtgggattata
agaaagaaatggctgaaacattggaaaatgcacgtaaagagactttggctcagtctaacgatgttttgga
tatgcagacagcaattcttctcacacactaaaaactgcaaatagctataccgatgtgactgtaagtagtt
ctaatcaatacacagatcataaattcagtcaacttgacaaccgtttagataaacttgacaaacgagttga

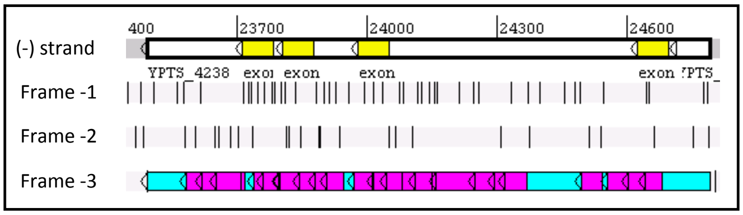

Peptide evidence:

AISESNQYTDHK
ALGDSAVTYGASSTAQK
ALGDSAVTYGASSTAQKDGVAIGAR
ASASDTGVAVGFNSK
DNDAVNVAQLKK
EMAETLENAR
ENSVSIGHESLNR
ETLAQSNDVLDAAK
GLASSAALNSLFQPYGVGK
KAISESNQYTDHK
KAISESNQYTDHKFSQLDNR
LAEAIQPQVLGGLDAR
LGVGLYPAKPILR
LSAVQISPNVDPK
QENPKLPPRGPQGPEK
QLTHLAAGTKDNDAVNVAQLKK
SSQALAIGSGYR
TANSYTDVTVSSSTK
TANSYTDVTVSSSTKK
TDRENSVSIGHESLNR
TTLETAEEHANKK
TTLETAEEHANKKSAEALVSAK
VDAQNSVAIGHSSHVAADHGYSIAIGDLSK
VDKGLASSAALNSLFQPYGVGK
VNFTAGVGGYR
VYADSNSSHTLK

Current sequence (peptide evidence in red): 432aa
MTKDFKISVSAALISALFSSPYAFAEEPEDGNDGIPRLSAVQISPNVDPKLGVGLYPAKPILRQENPKLPPRGPQGPEKKRARLAEAIQPQVLGGLDARAKGIHSIAIGATAEAAKPAAVAVGAG
SIATGVNSVAIGPLSKALGDSAVTYGASSTAQKDGVAIGARASASDTGVAVGFNSKVDAQNSVAIGHSSHVAADHGYSIAIGDLSKTDRENSVSIGHESLNRQLTHLAAGTKDNDAVNVAQLK
KEMAETLENARKETLAQSNDVLDAAKKHSNSVARTTLETAEEHANKKSAEALVSAKVYADSNSSHTLKTANSYTDVTVSSSTKKAISESNQYTDHKFSQLDNRLDKLDKRVDKGLASSAALNSL
FQPYGVGKVNFTAGVGGYRSSQALAIGSGYRVNESVALKAGVAYAGSSNVMYNASFNIEW

Error 84

Strain: Y. pestis pestoides F plasmid CD
Gene: YPDSF_4001.5

Classification: novel gene
Boundaries of gene: 50030..50578

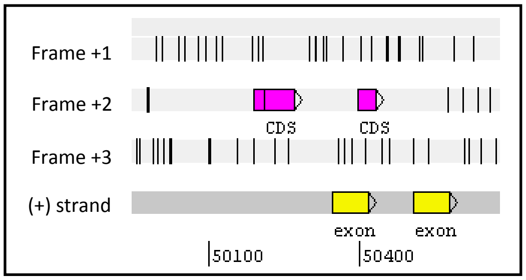

Oligo evidence:

tgttttctgtattagattgtgatatagagtatgtgttcatagggaaaaaagacagtgaagggaatataga
tgatcatctctaaatctgagttggaggaattgagtcgcgaatcctgcgatcaagaatgtattatgggatg

Peptide evidence:

DSEGNIEFYDPK
SGSILSTLEELWQAVGIVYR
VGIEPLKSGSILSTLEELWQAVGIVYR

Proposed sequence (peptide evidence in red): 182aa
MFIKDTYNMRALCTALEQSAPDTIINTSKEENNSYYCATAHLLRTDVCSLVNRVGIEPLKSGSILSTLEELWQAVGIVYRLYEWQHVSDIDTNFKKLPNNSDFGLVFSVLDCDIEYVFIGKKDSEG
NIEFYDPKNSLLIENDDIKKYLYDEDFHRFCIMLIISKSELEELSRESCDQECIMG

Strain: Y. pestis CO92
Gene: YPCD1.19c

Classification: annotated as putative virulence determinant protein
Boundaries of gene: complement (11562..12110)

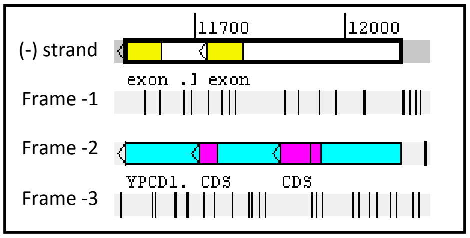

Oligo evidence:

tgttttctgtattagattgtgatatagagtatgtgttcatagggaaaaaagacagtgaagggaatataga
tgatcatctctaaatctgagttggaggaattgagtcgcgaatcctgcgatcaagaatgtattatgggatg

Peptide evidence:

DSEGNIEFYDPK
SGSILSTLEELWQAVGIVYR
VGIEPLKSGSILSTLEELWQAVGIVYR

Current sequence (peptide evidence in red): 182aa
MFIKDTYNMRALCTALEQSAPDTIINTSKEENNSYYCATAHLLRTDVCSLVNRVGIEPLKSGSILSTLEELWQAVGIVYRLYEWQHVSDIDTNFKKLPNNSDFGLVFSVLDCDIEYVFIGKKDSEG
NIEFYDPKNSLLIENDDIKKYLYDEDFHRFCIMLIISKSELEELSRESCDQECIMG

Strain: Y. pseudotuberculosis PB1/+
Gene: YPTS_4264

Classification: annotated as yop targeting protein YopK, YopQ
Boundaries of gene: complement(42819..43367)

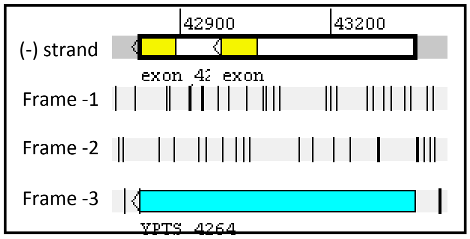

Oligo evidence:

Tgttttctgtattagattgtgatatagagtatgtgttcatagggaaaaaagacagtgaagggaatataga
tgatcatctctaaatctgagttggaggaattgagtcgcgaatcctgcgatcaagaatgtattatgggatg

Peptide evidence:

NA

Current sequence (peptide evidence in red): 182aa
MFIKDTYNMRALCTALEQSAPDTIINTSKEENNSYYCATAHLLRTDVCSLVNRVGIEPLKSGSILSTLEELWQAVGIVYRLYEWQHVSDIDTNFKKLPNNSDFGLVFSVLDCDIEYVFIGKKDSEG
NIEFYDPKNSLLIENDDIKKYLYDENFHRFCIMLIISKSELEELSRESCDQECIMG

Error 85

Strain: Y. pestis pestoides F plasmid CD
Gene: YPDSF_3943.5

Classification: novel gene
Boundaries of gene: 4758..5186

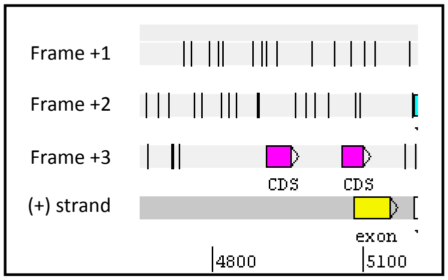

Oligo evidence:

tgagaacgttagcaaggagatagcgaacttttacgactggttgagcttggtcagtttaccagcagaaact

Peptide evidence:

LTLLGPILGFSGPEAR
HLSLDELNVENVSK

Proposed sequence (peptide evidence in red): 142aa
VINTTFTELLPKIASHFGLDKLSQDEYGLCELILNDRVVIMLRADEILNRLTLLGPILGFSGPEARSAASQLFFCYSINALNKDGPCFAWSEELGLIAFKHLSLDELNVENVSKEIANFYDWLSLVSLP
AETAPSYSIYSIG

Strain: Y. pestis CO92
Gene: YPCD1.73c

Classification: annotated as hypothetical protein
Boundaries of gene: complement(53679..54128)

Oligo evidence:

tgagaacgttagcaaggagatagcgaacttttacgactggttgagcttggtcagtttaccagcagaaact

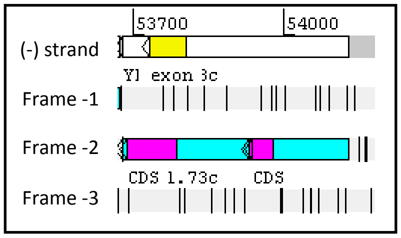

Peptide evidence:

EIANFYDWLSLVSLPAETQQELPLHTQSTQSVK
LTLLGPILGFSGPE
LTLLGPILGFSGPEAR

Current sequence (peptide evidence in red): 149aa
VINTTFTELLPKIASHFGLDKLSQDEYGLCELILNDRVVIMLRADEILNRLTLLGPILGFSGPEARSAASQLFFCYSINALNKDGPCFAWSEELGLIAFKHLSLDELNVENVSKEIANFYDWLSLVSLP
AETQQELPLHTQSTQSVKWG

Strain: Y. pseudotuberculosis PB1/+
Gene: YPTS_4225

Classification: annotated as hypothetical protein
Boundaries of gene: complement(16608..17057)

Oligo evidence:

tgagaacgttagcaaggagatagcgaacttttacgactggttgagcttggtcagtttaccagcagaaact

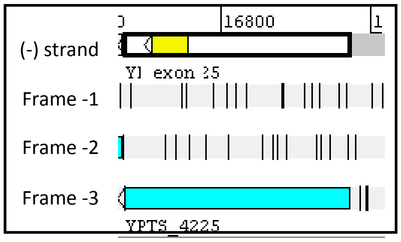

Peptide evidence:

NA

Current sequence (peptide evidence in red): 149aa
VINTTFTELLPKIASHFGLDKLSQDEYGLCELILNDRVVIMLRADEILNRLTLLGPILGFSGPEARSAASQLFFCYSINALNKDGPCFAWSEELGLIAFKHLSLDELNVENVSKEIANFYDWLSLVSLP
AETQQELPLHTQSTQSVKWG

Error 86

Strain: Y. pestis pestoides F plasmid CD
Gene: YPDSF_3969.5

Classification: novel gene
Boundaries of gene: complement (24847..25242)

Oligo evidence:

agttcgagaggttcagcgcactcatttcgaaacaggtaacctacctttattcttttctataaaactgaat

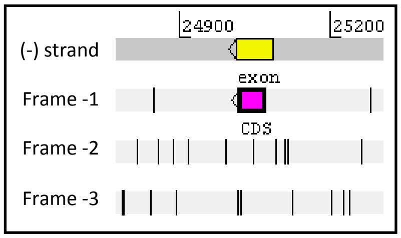

Peptide evidence:

THFETGNLPLFFSIK

Proposed sequence (peptide evidence in red): 131aa
VSRIIALIISFLLVGCATPPMPAQRIVGEVRMSRPLSRIAHIDVSMFGLYEGKVREVQRTHFETGNLPLFFSIKLNPAQRGEGELYLRSTLSFPERGVQAVAQQKLTGKNKVVLQMIPKTCYPNCQLPNTR

Strain: Y. pestis CO92
Gene: YPCD1.48

Classification: annotated as needle complex outer membrane lipoprotein precursor
Boundaries of gene: 35593..35988

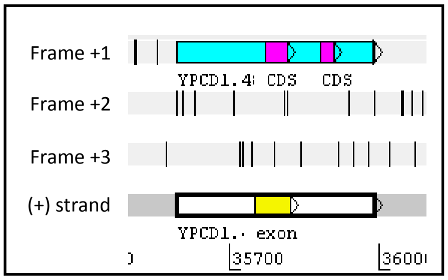

Oligo evidence:

agttcgagaggttcagcgcactcatttcgaaacaggtaacctacctttattcttttctataaaactgaat

Peptide evidence:

GVQAVAQQK
THFETGNLPLFFSIK

Current sequence (peptide evidence in red): 131aa
VSRIIALIISFLLVGCATPPMPAQRIVGEVRMSRPLSRIAHIDVSMFGLYEGKVREVQRTHFETGNLPLFFSIKLNPAQRGEGELYLRSTLSFPERGVQAVAQQKLTGKNKVVLQMIPKTCYPNCQLPNTR

Strain: Y. pseudotuberculosis PB1/+
Gene: YPTS_4293

Classification: annotated as type III secretion system chaperone YscW
Boundaries of gene: 68527..68922

Oligo evidence:

agttcgagaggttcagcgcactcatttcgaaacaggtaacctacctttattcttttctataaaactgaat

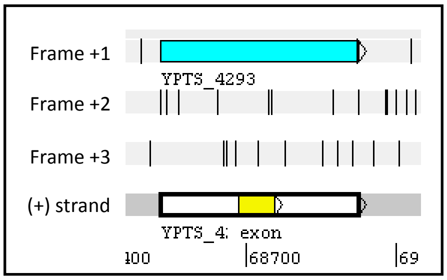

Peptide evidence:

NA

NA

Current sequence (peptide evidence in red): 131aa
VSRIIALIISFLLVGCATPPMPAQRIVGEVRMSRPLSRIAHIDVSMFGLYEGKVREVQRTHFETGNLPLFFSIKLNPAQRGEGELYLRSTLSFPERGVQAVAQQKLTGKNKVVLQMIPKTCYPNCQLPNTR

Error 87

Strain: Y. pestis pestoides F plasmid CD
Gene: YPDSF_4020.5

Classification: novel gene
Boundaries of gene: complement (64025..64498)

Oligo evidence:

atgaagtttcagtatataaagcaacaaattcctttaactcagtatgtctcggaattaaggcaagtttaaa

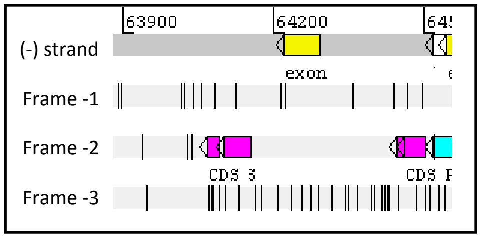

Peptide evidence:

GIPNFSNELETSMGLLR
KLSNSFK
MNLQNNLYSILEK
MNLQNNLYSILEKVESLK

Proposed sequence (peptide evidence in red): 154aa
MNLQNNLYSILEKVESLKNIKMLDKNNGEFTYIEGDYYFSDLTTDSLNIYEIKYLFTFENFDFFSNGKPNEVSVYKATNSFNSVCLGIKASLNSLDVERKELTVQFTYSMVFDASKGIPNFSNELETS
MGLLRIAPKKLSNSFKDKGIEHNYVN

Error 100

Strain: Y. pestis CO92
Gene: YPCD1.91.5

Classification: novel gene
Boundaries of gene: 65900..66361

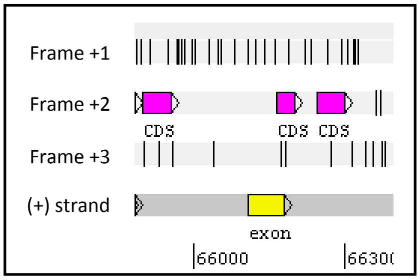

Oligo evidence:

atgaagtttcagtatataaagcaacaaattcctttaactcagtatgtctcggaattaaggcaagtttaaa

Peptide evidence:

ASLNSLDVERK
GIPNFSNELETSMGLLR
MNLQNNLYSILEKVESLK

Current sequence (peptide evidence in red): 154aa
MNLQNNLYSILEKVESLKNIKMLDKNNGEFTYIEGDYYFSDLTTDSLNIYEIKYLFTFENFDFFSNGKPNEVSVYKATNSFNSVCLGIKASLNSLDVERKELTVQFTYSMVFDASKGIPNFSNELETS
MGLLRIAPKKLSNSFKDKGIEHNYVN

Strain: Y. pseudotuberculosis PB1/+
Gene:YPTS_4242

Classification: annotated as hypothetical protein
Boundaries of gene: 28826..29290

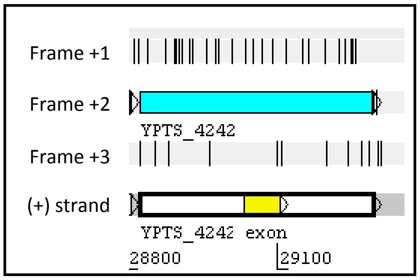

Oligo evidence:

atgaagtttcagtatataaagcaacaaattcctttaactcagtatgtctcggaattaaggcaagtttaaa

Peptide evidence:

ASLNSLDVERK
GIPNFSNELETSMGLLR
MNLQNNLYSILEKVESLK

Current sequence (peptide evidence in red): 154aa
MNLQNNLHSILEKVESLKNIKMLDKNNGEFTYIEGNYYFSDLTTDSLNIYEIKYLFTFENFDFFSNGKPNEVSVYKATNSFNSVCLGIKASLNSLDVERKELTVQFTYSMVFDASKGIPNFSNELETS
MGLLRIAPKKLSNSFKDKGIEHNYVN

Error 88

Strain: Y. pestis pestoides F plasmid MT
Gene: YPDSF_4088.5

Classification: novel gene
Boundaries of gene: 60858..61109

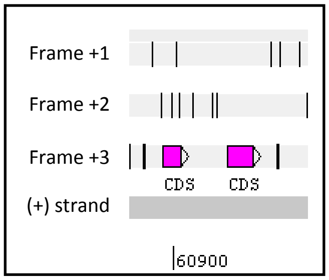

Oligo evidence:

NA

Peptide evidence:

HIQEAIEYALSK
SVWSTPDVPEHHATQIR

Proposed sequence (peptide evidence in red): 83aa
MKSHPNKHIQEAIEYALSKGWVWVPAGKSAHCFCKLRCGDKSGEHTSHHRSVWSTPDVPEHHATQIRQAVDQCGRIKNQMSKK

Error 48

Strain: Y. pestis CO92 plasmid MT
Gene: YPMT1.53c.5

Classification: novel gene
Boundaries of gene: 55421..55672

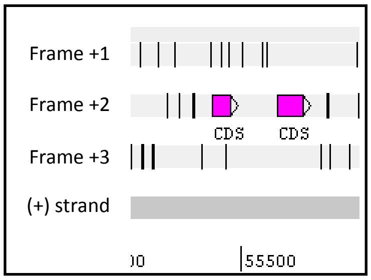

Oligo evidence:

NA

Peptide evidence:

HIQEAIEYALSK
SVWSTPDVPEHHATQIR

Proposed sequence (peptide evidence in red): 83aa
MKSHPNKHIQEAIEYALSKGWVWVPAGKSAHCFCKLRCGDKSGEHTSHHRSVWSTPDVPEHHATQIRQAVDQCGRIKNQMSKK

Strain: Y. pseudotuberculosis PB1/+
Gene: no ortholog

Error 89

Strain: Y. pestis pestoides F plasmid MT
Gene: YPDSF_4138.5

Classification: novel gene
Boundaries of gene: complement (125215..125541)

(-) strand

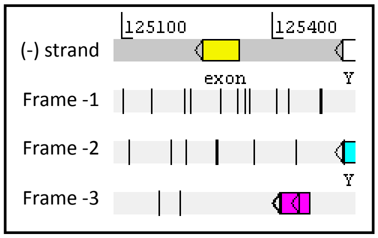

Oligo evidence:

cctgacggctacagagtattgtactcagtggaggataatgtaataacagcacacgcaatactatcccaca

Peptide evidence:

EVISGIIEHFEKR
HIESEPK
HIESEPKEVISGIIEHFEK
HIESEPKEVISGIIEHFEKR

Proposed sequence (peptide evidence in red): 108aa
MAIVIQYTRTVKTCIDDIASHLRHIESEPKEVISGIIEHFEKRVSDFPLGCQVCPELLKIGCAKYRECNTPDGYRVLYSVEDNVITAHAILSHRQDIQNLLFKRLIRI

Strain: Y. pestis CO92 plasmid MT
Gene: YPMT1.69

Classification: annotated as hypothetical protein
Boundaries of gene: 70690..71016

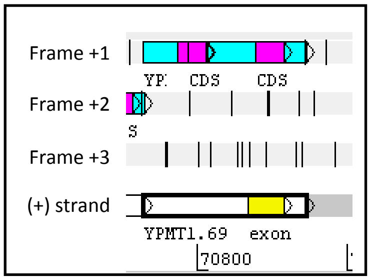

Oligo evidence:

cctgacggctacagagtattgtactcagtggaggataatgtaataacagcacacgcaatactatcccaca

Peptide evidence:

EVISGIIEHFEK
HIESEPKEVISGIIEHFEK
HIESEPKEVISGIIEHFEKR
VLYSVEDNVITAHAILSHR

Current sequence (peptide evidence in red): 108aa
MAIVIQYTRTVKTCIDDIASHLRHIESEPKEVISGIIEHFEKRVSDFPLGCQVCPELLKIGCAKYRECNTPDGYRVLYSVEDNVITAHAILSHRQDIQNLLFKRLIRI

Strain: Y. pseudotuberculosis PB1/+
Gene: no ortholog

Error 90

Strain: Y. pestis pestoides F
Gene: YPDSF_1005

Classification: frameshift
Boundaries of gene: Frame-2 1141715..1142086
Frame-3:1141443..1141703

Oligo evidence:

atgactaactgggatgatttttatgggacatgggctgatttttgttggggtgatgacagagattgccatt
ggctttccttgagcatatcgttcctacacgtaaaacatgtactcagattttagtctggcgtactgttcaa
caggcgtgaaagaattctctttgcctggatttgtaactcctcacgggtatcgtctacttgtttctgtaaa

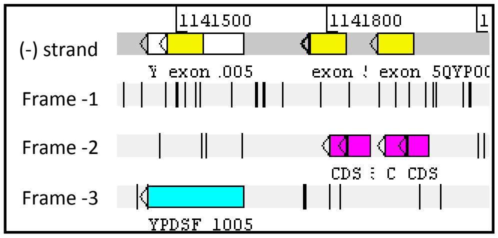

Peptide evidence:

EFSLPGFVTPHGYR
LAFLEHIVPTR
NIEHTEAFMASGVK
LVTDAAIPETVYAVK

Proposed sequence (peptide evidence in red):
Current annotation is underlined

Frame-2: 124aa
MNKYIANEKMPLLIPAAGFDAKLSMTEKNIEHTEAFMASGVKEFSLPGFVTPHGYRLLVSVNGEHYRLVTDAAIPETVYAVKLAFLEHIVPTRKTCTQILVWRTVQPQHDSAVHGLPQEFFRH
F

Frame-3: 87aa
LTQYSIVVSDSEQTNEGRRFWERMIAWAIQAEGHYVYVSNGSEEDRPLTFMTNWDDFYGTWADFCWGDDRDCHCHRLFVISTEQLH

Strain: Y. pestis CO92
Gene: YPO2124

Classification: annotated as hypothetical protein
Boundaries of gene: 2392729..2393361

Oligo evidence:

caggcgtgaaagaattctctttgcctggatttgtaactcctcacgggtatcgtctacttgtttctgtaaa
ggctttccttgagcatatcgttcctacacgtaaaacatgtactcagattttagtctggcgtactgttcaa
atgactaactgggatgatttttatgggacatgggctgatttttgttggggtgatgacagagattgccatt

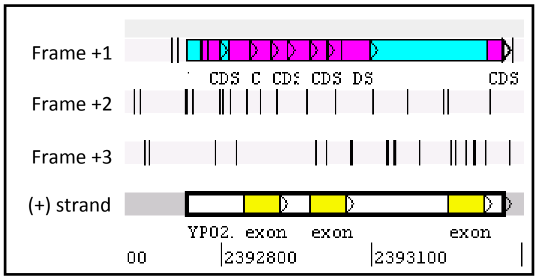

Peptide evidence:

EFSLPGFVTPHGYR
LAFLEHIVPTR
LFVISTEQLH
LLVSVNGEHYR
LVTDAAIPETVYAVK
MPLLIPAAGFDAK
NIEHTEAFMASGVK
NIEHTEAFMASGVKEFSLPGFVTPHGYR
PAAGFDAK
PLLIPAAGFDAK
TCTQILVWRTVQPQHDSAVHGLPQEFFR
TVQPQHDSAVHGLPQEFFR

Current sequence (peptide evidence in red): 210aa
MNKYIANEKMPLLIPAAGFDAKLSMTEKNIEHTEAFMASGVKEFSLPGFVTPHGYRLLVSVNGEHYRLVTDAAIPETVYAVKLAFLEHIVPTRKTCTQILVWRTVQPQHDSAVHGLPQEFFRH
FLTQYSIVVSDSEQTNEGRRFWERMIAWAIQAEGHYVYVSNGSEEDRPLTFMTNWDDFYGTWADFCWGDDRDCHCHRLFVISTEQLH

Strain: Y. pseudotuberculosis PB1/+
Gene: no ortholog

Error 91

Strain: Y. pestis pestoides F
Gene: YPDSF_2750.5

Classification: frameshift
Boundaries of gene: frame-3: complement (3119589..3119711)
frame-1: complement (3118639..3119631)

Oligo evidence:

tgattaataatagtttctggcaaggtaaacgggtttttgtaacaggccatactgggtttaaaggtggctg
aatatgccgaaggttggaactttggtcctaacgatgctgatgctactccagtaaaaaacattgttgaaca

Peptide evidence:

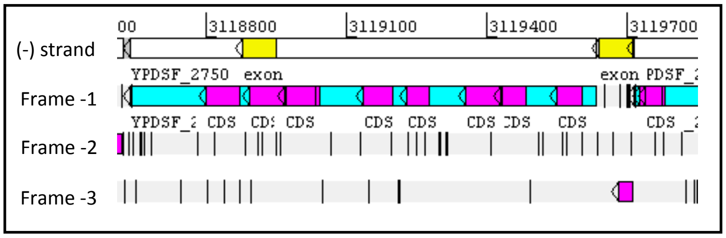

MINNSFWQGK
AIRPWQHVLEPLSGYLLLAQK
LYTDGAEYAEGWNFGPNDADATPVK
RLLFAPPTVPSLFETAR
YWGEGASWQLDGNAHPHEAHYLK
EFQPEIVFHMAAQPLVR
ENEAMGGYDPYSNSK
ENEAMGGYDPYSNSK
LSYSEPVETYSTNVMGTVYLLEAIR
NPHAIRPWQHVLEPLSGYLLLAQK
NSFFNPANYGQHGTAVATVR

Proposed sequence (peptide evidence in red):
Frame-3: 41aa
MINNSFWQGKRVFVTGHTGFKGGWLSLWLQTMGATVKGYSLPPPRCLAYLRPHELPTGCNRKSVIFVIKTNY

Frame-1: 331aa
MVANHGGNGKRLLFAPPTVPSLFETARVADGMQSEIGDIRDQNKLLESIREFQPEIVFHMAAQPLVRLSYSEPVETYSTNVMGTVYLLEAIRHVGGVKAVVNITSDKCYDNKEWIWGYRENEAMGGYDPYSNSKGCAELVTSSYRNSFFNPANYGQHGTAVATVRAGNVIGGGDWALDRIVPDILRAFEQSQPVIIRNPHAIRPWQHVLEPLSGYLLLAQKLYTDGAEYAEGWNFGPNDADATPVKNIVEQMVKYWGEGASWQLDGNAHPHEAHYLKLDCSKAKMQLGWHPRWNLNTTLEYIVGWHKNWLSGTDMHEYSITEINNYMNTK

Strain: Y. pestis CO92
Gene: YPO3114

Classification: annotated as pseudogene
Boundaries of gene: frame-2: complement (3471347..3471568)
frame-3: complement (3470493..3471437)

Oligo evidence:

tgattaataatagtttctggcaaggtaaacgggtttttgtaacaggccatactgggtttaaaggtggctg
aatatgccgaaggttggaactttggtcctaacgatgctgatgctactccagtaaaaaacattgttgaaca

Peptide evidence:

NA

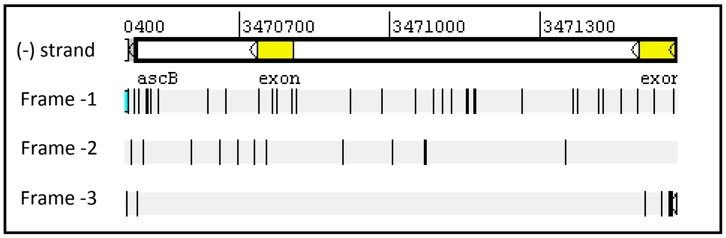

Current sequence (peptide evidence in red):
Frame-2: 41aa
MINNSFWQGKRVFVTGHTGFKGGWLSLWLQTMGATVKGYSLPPPPRCLAYLRPHELPTGCNRKSVIFVIKTNY

Frame-3: 316aa
PTVPSLFETARVADGMQSEIGDIRDQNKLLESIREFQPEIVFHMAAQPLVRLSYSEPVETYSTNVMGTVYLLEAIRHVGGVKAVVNITSDKCYDNKEWIWGYRENEAMGGYDPYSNSKGCAELVTSSYRNSFFNPANYGQHGTAVATVRAGNVIGGGDWALDRIVPDILRAFEQSQPVIIRNPHAIRPWQHVLEPLSGYLLLAQKLYTDGAEYAEGWNFGPNDADATPVKNIVEQMVKYWGEGASWQLDGNAHPHEAHYLKLDCSKAKMQLGWHPRWNLNTTLEYIVGWHKNWLSGTDMHEYSITEINNYMNTK

Strain: Y. pseudotuberculosis PB1/+
Gene: YPTS_1043

Classification: annotated as CDP-glucose 4,6-dehydratase
Boundaries of gene: 1182733..1183806

Oligo evidence:

tgattaataatagtttctggcaaggtaaacgggtttttgtaacaggccatactgggtttaaaggtggctg
aatatgccgaaggttggaactttggtcctaacgatgctgatgctactccagtaaaaaacattgttgaaca

Peptide evidence:

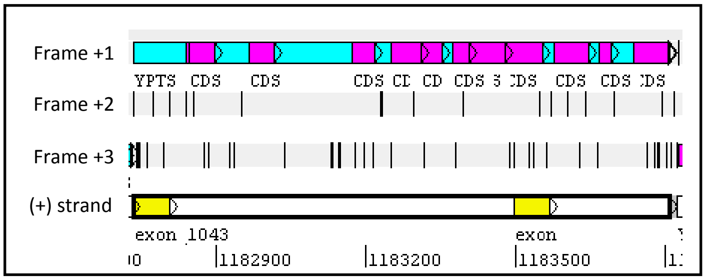

AFEQSQPVIIR
AGNVIGGGDWALDR
EFQPEIVFHMAAQPLVR
ENEAMGGYDPYSNSK
GYSLTPPTVPSLFETAR
LYTDGAEYAEGWNFGPNDADATPVK
MQLGWHPR
NPHAIRPWQHVLEPLSGYLLLAQK
NSFFNPANYGQHGTAVATVR
NWLSGTDMHEYSITEINNYMNTK
VKGYSLTPPTVPSLFETAR
YWGEGASWQLDGNAHPHEAHYLK

Current sequence (peptide evidence in red): 357aa
MINNSFWQGKRVFVTGHTGFKGGWLSLWLQTMGAKVKGYSLTPPTVPSLFETARVADGMQSEIGDIRDQNKLLESIREFQPEIVFHMAAQPLVRLSYSEPVETYSTNVMGTVYLLEAIRHVGGVKAVVNITSDKCYDNKEWIWGYRENEAMGGYDPYSNSKGCAELVTSSYRNSFFNPANYGQHGTAVATVRAGNVIGGGDWALDRIVPDILRAFEQSQPVIIRNPHAIRPWQHVLEPLSGYLLLAQKLYTDGAEYAEGWNFGPNDADATPVKNIVEQMVKYWGEGASWQLDGNAHPHEAHYLKLDCSKAKMQLGWHPRWNLNTTLEYIVGWHKNWLSGTDMHEYSITEINNYMNTK

Error 92

Strain: Y. pestis pestoides F
Gene: YPDSF_0824
Classification: upstream start site
Boundaries of gene: 930976..934863

Oligo evidence:

tccactcaacttggtgagttgatgctccgtgaccaacagcgcattcagcgtcggctacatagcgcacgaa
tgtgttggcaaccaacgtggcagaaacgtcactcacggtgccgggtatcaaatatgttatcgatcccggc
caagagtttaccggtgctcgtaacgcccgctttgctattttccccggctccggcttattcaaaaagcccc

Peptide evidence:

FSQHFNNAPIIEVSGR
GVIGHTQPR
ILQTIEQLSV
INNPDAIQAITHEIEAEIAIAMQR
KINNPDAIQAITHEIEAEIAIAMQR
LLLLNIPSPIK
LPIEPISQASANQR
SSLTALSTQLGELMLR
SVHTAILTGLLSHIGQK
SVHTAILTGLLSHIGQKDVEK
TNLASVILQMTSLGLGDIAAFPFVEAPDKR
VTPLETGLLEALER
YRPIVDDADDVDRDQLQAIFDAVDELGR

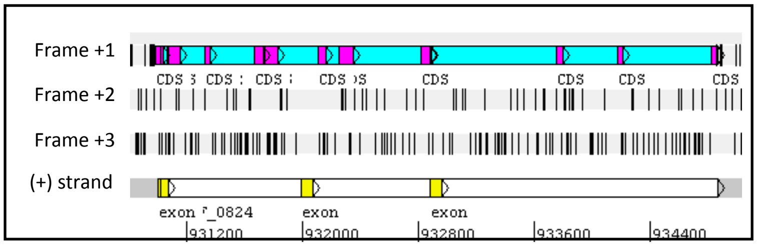

Proposed sequence (peptide evidence in red): 1295aa
Current annotation is underlined

MKSSLTALSTQLGELMLRDQQRIQRRLHSARKINNPDAIQAITHEIEAEIAIAMQRVTRRRAACPAISYPDNLPVSQKKQDIYNAIRDHQVIIVAGETGSGKTTQLPKICLELGRGVKGVIGHTQPRRLAARTVANRIADELDTSLGGCVGYKVRFNDQVGENTLV
KLMTDGILLAEIQQDRLLMQYDTLIIDEAHERSLNIDFILGYLRELLPKRPDLKVIITSATIDPQRFSQHFNNAPIIEVSGRTYPVEVRYRPIVDDADDVDRDQLQAIFDAVDELGRESAGDILIFMSGEREIRDTADALIKQNLPHTEVLPLYARLSNSEQNRVFQSHH
GRRIVLATNVAETSLTVPGIKYVIDPGTARISRYSFRTKVQRLPIEPISQASANQRKGRCGRVSDGICIRLYSEQDFLSRPEFTDPEILRTNLASVILQMTSLGLGDIAAFPFVEAPDKRNIQDGVRLLEELGAIQTASNGHQQLTPLGRQLAQLPVDPRLARMVLQA
QKSGCVRELMIITSALSIQDPRERPVDKQQASDEKHRRFADKDSDFLAFVNLWDYLKEQQKALSSAQFRKLCRSDYLNYLRVREWQDIYTQLRQVVKELGIPVNSVAADYRSVHTAILTGLLSHIGQKDVEKQEFTGARNARFAIFPGSGLFKKPPKWVMVAEL
VETSRLWGRIAARIEPEWIEPLAQHLVKHHYSDPHWEKAQGAVMASEKVTLFGLPIVTERKINYGPIDPLLCRELFIRHGLVEGDWQTRHAFFRMNLKLLAEVEELEHKSRRRDILVDDETLFNFYDQRIGRDVISALHFDSWWKQASQTKPELLNFEKTMLIKE
GANKVNPLDYPNFWYQGELKLRLFYQFEPGTDADGVTVHIPLPILNQIHEQGFDWQIPGIRRELVIALIKSLPKPVRRNFVPAPNYADAFLARVTPLETGLLEALERELRRMTGVTVSRDSWQLDQVPDHLKITFRVLDDKNRILREGKDLAALKLQLQEKVQETL
SAVADDGIEQNNLHIWSFGDLPICYEQRRGGYEVKAYPALVDEKDSVAIRLFDTESQQQQAMWQGTRRLLLLNIPSPIKYLHEKLPNKSKLGLYFNTYGNVMDLIDDCIACGVDKLVAQHGGPVWQEADFARLQEKVRAELNDTVVDIAKQVEQILTLVFSIN
KRLKGRIDISLVLALSDIKAQLAGLIYRGFVTSNGWKRLPDTLRYLKAIERRLEKLAVDPHRDRAQMLRIEHVQQVWQQWLNKLPPKRQQDEEVKEVRWMIEELRVSLFAQQLGTPYPVSDKRILQTIEQLSV

Error 99

Strain: Y. pestis CO92
Gene: YPO2322
Classification: upstream start site
Boundaries of gene: complement (2608715..2612602)

Oligo evidence:

tccactcaacttggtgagttgatgctccgtgaccaacagcgcattcagcgtcggctacatagcgcacgaa
tgtgttggcaaccaacgtggcagaaacgtcactcacggtgccgggtatcaaatatgttatcgatcccggc
caagagtttaccggtgctcgtaacgcccgctttgctattttccccggctccggcttattcaaaaagcccc

Peptide evidence:

DILVDDETLFNFYDQR
ELMIITSALSIQDPR
FADKDSDFLAFVNLWDYLK
FSQHFNNAPIIEVSGR
GVIGHTQPR
IEHVQQVWQQWLNK
ILQTIEQLSV
INNPDAIQAITHEIEAEIAIAMQR
KINNPDAIQAITHEIEAEIAIAMQR
LLLLNIPSPIK
NFVPAPNYADAFLAR
SSLTALSTQLGELMLR
SVHTAILTGLLSHIGQK
SVHTAILTGLLSHIGQKDVEKQEFTGAR
TNLASVILQMTSLGLGDIAAFPFVEAPDKR
VREWQDIYTQLR
VTLFGLPIVTER
VTPLETGLLEALER
YRPIVDDADDVDRDQLQAIFDAVDELGR

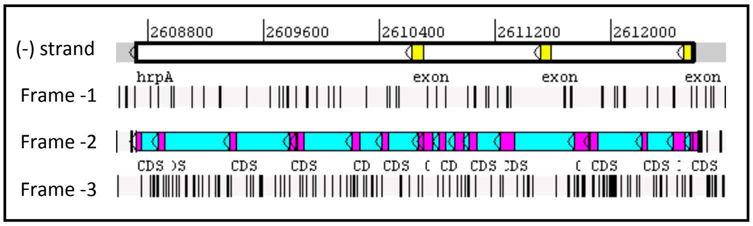

Proposed sequence (peptide evidence in red): 1295aa Current annotation is underlined

VKSSLTALSTQLGELMLRDQQRIQRRLHSARKINNPDAIQAITHEIEAEIAIAMQRVTRRRAACPAISYPDNLPVSQKKQDIYNAIRDHQVIIVAGETGSGKTTQLPKICLELGRGVKGVIGHTQPRRLAARTVANRIADELDTSLGGCVGYKVRFNDQVGENTLV
KLMTDGILLAEIQQDRLLMQYDTLIIDEAHERSLNIDFILGYLRELLPKRPDLKVIITSATIDPQRFSQHFNNAPIIEVSGRTYPVEVRYRPIVDDADDVDRDQLQAIFDAVDELGRESAGDILIFMSGEREIRDTADALIKQNLPHTEVLPLYARLSNSEQNRVFQSHH
GRRIVLATNVAETSLTVPGIKYVIDPGTARISRYSFRTKVQRLPIEPISQASANQRKGRCGRVSDGICIRLYSEQDFLSRPEFTDPEILRTNLASVILQMTSLGLGDIAAFPFVEAPDKRNIQDGVRLLEELGAIQTASNGHQQLTPLGRQLAQLPVDPRLARMVLQA
QKSGCVRELMIITSALSIQDPRERPVDKQQASDEKHRRFADKDSDFLAFVNLWDYLKEQQKALSSAQFRKLCRSDYLNYLRVREWQDIYTQLRQVVKELGIPVNSVAADYRSVHTAILTGLLSHIGQKDVEKQEFTGARNARFAIFPGSGLFKKPPKWVMVAEL
VETSRLWGRIAARIEPEWIEPLAQHLVKHHYSDPHWEKAQGAVMASEKVTLFGLPIVTERKINYGPIDPLLCRELFIRHGLVEGDWQTRHAFFRMNLKLLAEVEELEHKSRRRDILVDDETLFNFYDQRIGRDVISALHFDSWWKQASQTKPELLNFEKTMLIKE
GANKVNPLDYPNFWYQGELKLRLFYQFEPGTDADGVTVHIPLPILNQIHEQGFDWQIPGIRRELVIALIKSLPKPVRRNFVPAPNYADAFLARVTPLETGLLEALERELRRMTGVTVSRDSWQLDQVPDHLKITFRVLDDKNRILREGKDLAALKLQLQEKVQETL
SAVADDGIEQNNLHIWSFGDLPICYEQRRGGYEVKAYPALVDEKDSVAIRLFDTESQQQQAMWQGTRRLLLLNIPSPIKYLHEKLPNKSKLGLYFNTYGNVMDLIDDCIACGVDKLVAQHGGPVWQEADFARLQEKVRAELNDTVVDIAKQVEQILTLVFSIN
KRLKGRIDISLVLALSDIKAQLAGLIYRGFVTSNGWKRLPDTLRYLKAIERRLEKLAVDPHRDRAQMLRIEHVQQVWQQWLNKLPPKRQQDEEVKEVRWMIEELRVSLFAQQLGTPYPVSDKRILQTIEQLSV

Strain: Y. pseudotuberculosis PB1/+
Gene: YPTS_2317
Classification: ATP-dependent helicase HrpA
Boundaries of gene: complement (2564882..2568769)

Oligo evidence:

tccactcaacttggtgagttgatgctccgtgaccaacagcgcattcagcgtcggctacatagcgcacgaa
tgtgttggcaaccaacgtggcagaaacgtcactcacggtgccgggtatcaaatatgttatcgatcccggc
caagagtttaccggtgctcgtaacgcccgctttgctattttccccggctccggcttattcaaaaagcccc

Peptide evidence:

EGKDLAALKLQLQEK
FSQHFNNAPIIEVSGR
INNPDAIQAITHEIEAEIAIAMQR
KINNPDAIQAITHEIEAEIAIAMQR
QNLPHTEVLPLYAR
RMTGVTVSR
SSLTALSTQLGELMLR
SVHTAILTGLLSHIGQK
TNLASVILQMTSLGLGDIAAFPFVEAPDKR
VTPLETGLLEALER

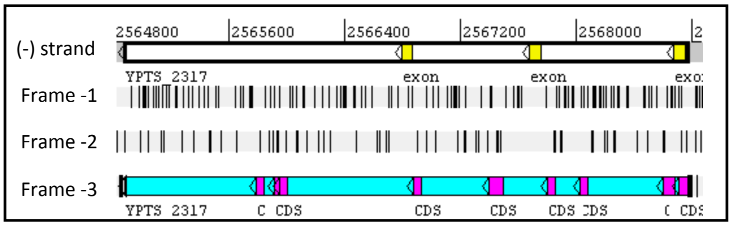

Current sequence (peptide evidence in red): 1295aa

VKSSLTALSTQLGELMLRDQQRIQRRLHSARKINNPDAIQAITHEIEAEIAIAMQRVTRRRAACPAISYPDNLPVSQKKQDIYNAIRDHQVIIVAGETGSGKTTQLPKICLELGRGVKGVIGHTQPRRLAARTVANRIADELDTSLGGCVGYKVRFNDQVGENTLV
KLMTDGILLAEIQQDRLLMQYDTLIIDEAHERSLNIDFILGYLRELLPKRPDLKVIITSATIDPQRFSQHFNNAPIIEVSGRTYPVEVRYRPIVDDADDVDRDQLQAIFDAVDELGRESAGDILIFMSGEREIRDTADALIKQNLPHTEVLPLYARLSNSEQNRVFQSHH
GRRIVLATNVAETSLTVPGIKYVIDPGTARISRYSFRTKVQRLPIEPISQASANQRKGRCGRVSDGICIRLYSEQDFLSRPEFTDPEILRTNLASVILQMTSLGLGDIAAFPFVEAPDKRNIQDGVRLLEELGAIQTASNGHQQLTPLGRQLAQLPVDPRLARMVLQA
QKSGCVRELMIITSALSIQDPRERPVDKQQASDEKHRRFADKDSDFLAFVNLWDYLKEQQKALSSAQFRKLCRSDYLNYLRVREWQDIYTQLRQVVKELGIPVNSVAADYRSVHTAILTGLLSHIGQKDVEKQEFTGARNARFAIFPGSGLFKKPPKWVMVAEL
VETSRLWGRIAARIEPEWIEPLAQHLVKHHYSDPHWEKAQGAVMASEKVTLFGLPIVTERKINYGPIDPLLCRELFIRHGLVEGDWQTRHAFFRMNLKLLAEVEELEHKSRRRDILVDDETLFNFYDQRIGRDVISARHFDSWWKQASQTKPELLNFEKTMLIKE
GANKVNPLDYPNFWYQGELKLRLSYQFEPGTDADGVTVHIPLPILNQIHEQGFDWQIPGIRRELVIALIKSLPKPVRRNFVPAPNYADAFLARVTPLETGLLEALERELRRMTGVTVSRDSWQLDQVPDHLKITFRVLDDKNRILREGKDLAALKLQLQEKVQETL
SAVADDGIEQNNLHIWSFGDLPICYEQRRGGYEVKAYPALVDEKDSVAIRLFDTESQQQQAMWQGTRRLLLLNIPSPIKYLHEKLPNKSKLGLYFNTYGNVMDLIDDCIACGVDKLVAQHGGPAWQEADFARLQEKVRAELNDTVVDIAKQVEQILTLVFSIN
KRLKGRIDISLALALSDIKAQLAGLIYRGFVTSNGWKRLPDTLRYLKAIERRLEKLAVDPHRDRAQMLRIEHVQQVWQQWLNKLPPKRQQDEEVKEVRWMIEELRVSLFAQQLGTPYPVSDKRILQTIEQLSV

Error 93

Strain: Y. pestis pestoides F plasmid CD
Gene: YPDSF_3996

Classification: upstream start site YopM
Boundaries of gene: 44576..45805

Oligo evidence:

aaatccaagaaatgtatctaatactttttgcaagaaccattacgtcattcttctaatttaactgagatg
cagcaatgaaataagatccttatgcgatttaccccccttcactggaagaacttaatgtcagtaataatag

Peptide evidence:

ALSDLPPLLEYLGVSNNQLEK
ALSDLPPLLEYLGVSNNQLEKLPELQNSSFLK
IIDVDNNSLK
KLPDLPLSLESIVAGNNILEELPELQNLPFLTTIYADNNLLK
KLPDLPPSLEFIAAGNNQLEELPELQNLPFLTAIYADNNSLK
LPELQNSSFLK
TLPDLPPSLEALNVR
PELQNLPFLTAIYADNNSLK
LIASFNHLAEVPELPQNLK
NVSNTFLQEPLR

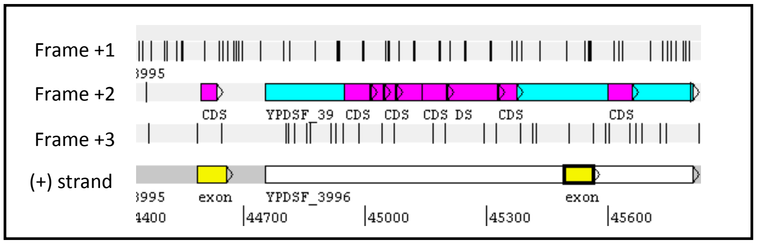

Proposed sequence (peptide evidence in red): 409aa
Current annotation is underlined

MFINPRNVSNTFLQEPLRHSSNLTEMPVEAENVKSKTEYYNAWSEWERNAPPGNGEQREMAVSRLRDCLDRQAHELELNNLGLSSLPELPPHLESLVASCNSLTELPELPQSLKSLLVDNNNL
KALSDLPPLLEYLGVSNNQLEKLPELQNSSFLKIIDVDNNSLKKLPDLPPSLEFIAAGNNQLEELPELQNLPFLTAIYADNNSLKKLPDLPLSLESIVAGNNILEELPELQNLPFLTTIYADNNLLKTLP
DLPPSLEALNVRDNYLTDLPELPQSLTFLDVSENIFSGLSELPPNLYYLNASSNEIRSLCDLPPSLEELNVSNNKLIELPALPPRLERLIASFNHLAEVPELPQNLKQLHVEYNPLREFPDIPESVEDLR
MNSERVVDPYEFAHETTDKLEDDVFE

Strain: Y. pestis CO92 plasmid CD
Gene: YPCD1.26c

Classification: annotated as YopM
Boundaries of gene: complement (16336..17565)

Oligo evidence:

aaatccaagaaatgtatctaatactttttgcaagaaccattacgtcattcttctaatttaactgagatg
cagcaatgaaataagatccttatgcgatttaccccccttcactggaagaacttaatgtcagtaataatag

Peptide evidence:

ALSDLPPLLEYLGVSNNQLEK
KLPDLPLSLESIVAGNNILEELPELQNLPFLTTIYADNNLLK
KLPDLPPSLEFIAAGNNQLEELPELQNLPFLTAIYADNNSLK
LIASFNHLAEVPELPQNLK
LPELQNSSFLK

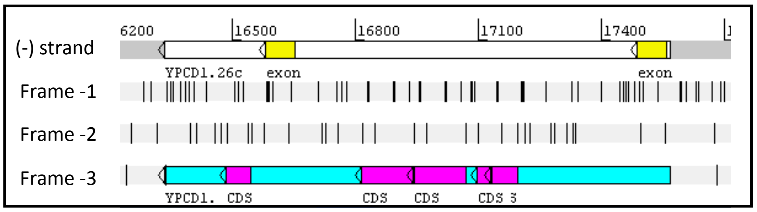

Current sequence (peptide evidence in red): 409aa
MFINPRNVSNTFLQEPLRHSSNLTEMPVEAENVKSKTEYYNAWSEWERNAPPGNGEQREMAVSRLRDCLDRQAHELELNNLGLSSLPELPPHLESLVASCNSLTELPELPQSLKSLLVDNNNL
KALSDLPPLLEYLGVSNNQLEKLPELQNSSFLKIIDVDNNSLKKLPDLPPSLEFIAAGNNQLEELPELQNLPFLTAIYADNNSLKKLPDLPLSLESIVAGNNILEELPELQNLPFLTTIYADNNLLKTLP
DLPPSLEALNVRDNYLTDLPELPQSLTFLDVSENIFSGLSELPPNLYYLNASSNEIRSLCDLPPSLEELNVSNNKLIELPALPPRLERLIASFNHLAEVPELPQNLKQLHVEYNPLREFPDIPESVEDLR
MNSERVVDPYEFAHETTDKLEDDVFE

Strain: Y. pseudotuberculosis PB1/+ plasmid
Gene: YPTS_4270

Classification: annotated as YopM
Boundaries of gene: complement(47586.. 49175)

Oligo evidence:

aaatccaagaaatgtatctaatactttttgcaagaaccattacgtcattcttctaatttaactgagatg
ataataacaatctgaaggcattatccgatttaccttccttcactggaatatcttactgctagtagtaataa

Peptide evidence:

NA

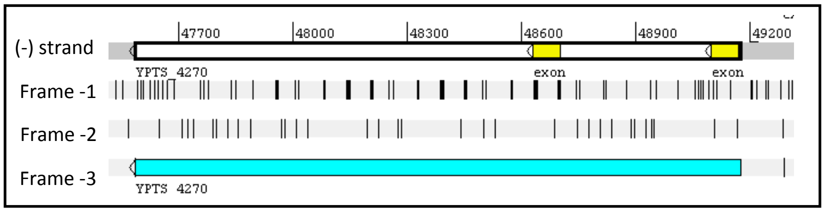

Current sequence (peptide evidence in red): 529aa
MFINPRNVSNTFLQEPLRHSSNLTEMPVEAENVKSKTEYYNAWSEWERNAPPGNGEQREMAVSRLRDCLDRQAHELELNNLGLSSLPELPPHLESLVASCNSLTELPELPQSLKSLQVENNN
LKALPDLPPSLKKLHVRENDLTDLPELPQSLESLRVDNNNLKALSDLPPSLEYLTASSNKLEELPELQNLPFLAAIYADNNLLETLPDLPPSLKKLHVRENDLTDLPELPQSLESLQVDNNNLKALSD
LPPSLEYLTASSNKLEELPELQNLPFLAAIYADNNLLETLPDLPPHLEILVASYNSLTELPELPQSLKSLRVDNNNLKALSDLPPSLEYLTASSNKLEELPELQNLPFLAAIYADNNLLETLPDLPPSLKK
LHVRENDLTDLPELPQSLTFLDVSDNNISGLSELPPNLYYLDASSNEIRSLCDLPPSLVDLNVKSNQLSELPALPPHLERLIASFNYLAEVPELPQNLKQLHVEQNALREFPDIPESLEELEMDSERV
VDPYEFAHETTDKLEDDVFE

Error 94

Strain: Y. pseudotuberculosis PB1/+
Gene: YPTS_1064
Classification: pseudogene
Boundaries of gene: frame+3:1206042..1207007
frame +2 1207007..1207696

Oligo evidence:

gggcgagtgcctgggaaaaagataaaacttacgacatcaccatattgcacaccaatgatcatcatggtca
tccgtaatggcgaattaacattggtcaactaccagttaatcccgatcaatttaaagaaaaaattgagaaa
ttaacattggtcaactaccagttaatcccgatcaatttaaagaaaaaattgagaaagctgatggcactcg
taatatggcgcgtttattattagcctctcaaatagaacgttcaggagctgactttgccgtaatgagtggt
tgcaggtgatatcacttataaagatgtgctcaaggttcaaccctttggtaatacgctggtttacgttgat

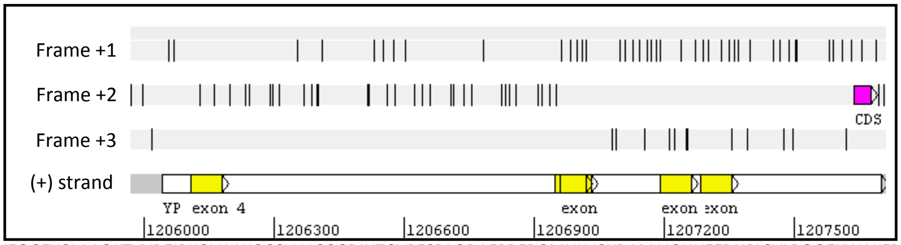

Peptide evidence:

HSPLDAEQYQPK

Proposed sequence (peptide evidence in red): 551aa
Frame+3:
MRFSLTTTLGALAVSLALAPGWASAWEKDKTYDITILHTNDHHGHFWQNEQGEYGLAAQKTVVDEIRKQVAAKGGSLLLLSGGDINTGVPESDLQDAEPDFRGMNLVGYDAMAIGNHEFDNPLSVLRQQEKWAKFPLLSANIYQKGTQQRLFKPYALFDKQGIKIAVLGLTTDDTPKVSSPENVVDIEFRVPAVEAKHVVEQLRKTEKPDIIIAATHMGHYDDGKHGSNAPGDVEMARSLPAGYLDMIVGGHSQNPVCMASENHRQVDYVPGSPCVPDRQNGTWIVQAHEWGKYVGRADFTFRNGELTLVNYQLIPINLKKK
Frame+2:
IEKADGTREHVFYTQEIAQDPTMLKLLTPFEERGKAQLGVRIGSVNGRLEGDRNKVRFEQTNMARLLLASQIERSGADFAVMSGGGVRDSIGAGDITYKDVLKVQPFGNTLVYVDMKGSEVEKYLAVVANKQPDSGAYAQFANVSLVADGQGVSNVKIQGEPLDPNKTYRMATLNFNALGGDGYPRIDNSPSYVNTGFIDAEVLKQYIEKHSPLDAEQYQPKGEIVYNQ

Strain: Y. pestis pestoides F
Gene: YPDSF_2730/ YPDSF_2729/ YPDSF_2728
Classification: annotated as 5'-nucleotidase/2' 3'-cyclic phosphodiesterase
Boundaries of gene: YPDSF_2730 complement(3096680..3096937)
YPDSF_2729 complement(3095899..3096867)
YPDSF_2728 complement(3095282..3095905)

Oligo evidence:
gggcgagtgcctgggaaaaagataaaacttacgacatcaccatattgcacaccaatgatcatcatggtca
tccgtaatggcgaattaacattggtcaactaccagttaatcccgatcaatttaaagaaaaaattgagaaa
ttaacattggtcaactaccagttaatcccgatcaatttaaagaaaaaattgagaaagctgatggcactcg
tgcaggtgatatcacttataaagatgtgctcaaggttcaaccctttggtaatacgctggtttacgttgat

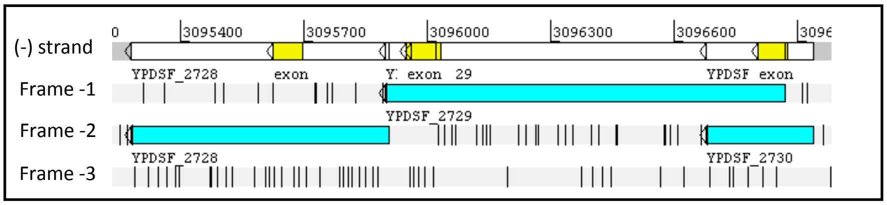

Peptide evidence:

NA

Current sequence (peptide evidence in red):
YPDSF_2730 85aa
MRFSLTTTLGALAVSLALAPGWASAWEKDKTYDITILHTNDHHGHFWQNEQGEYGLAAQKTVVDEIRKQVAAKGGSLLLLSGGDY

YPDSF_2729 322 aa
MPGKKIKLTTSPYCTPMIIMVTSGKTSKVSMVLLHRKPWWMKFVNKWPQKEEAYCYSRAVIINTGVPESDLQDAEPDFRGMNLVGYDAMAIGNHEFDNPLSVLRQQEKWAKFPLLSANIYQKGTQQRLFKPYALFDKQGIKIAVLGLTTDDTPKVSSPENVVDIEFRVPAVEAKHVVEQLRKTEKPDIIIAATHMGHYDDGKHGSNAPGDVEMARSLPAGYLDMIVGGHSQNPVCMASENHRQVDYVPGSPCVPDRQNGTWIVQAHEWGKYVGRADFTFRNGELTLVNYQLIPINLKKKLRKLMALASTSFIPKRLLRTPQC

YPDSF_2728 207 aa
MLKLLTPFEERGKAQLGVRIGSVNGRLEGDRNKVRFEQTNMARLLLASQIERSGADFAVMSGGGVRDSIGAGDITYKDVLKVQPFGNTLVYVDMKGSEVEKYLAVVANKQPDSGAYAQFANVSLVADGQGVSNVKIQGEPLDPNKTYRMATLNFNALGGDGYPRIDNSPSYVNTGFIDAEVLKQYIEKHSPLDAEQYQLKGEIVYNQ

Strain: Y. pestis CO92
Gene: YPO3090
Classification: annotated as pseudogene
Boundaries of gene: frame-2: complement (3448532..3448783)
frame-1: complement (3447817..3448530)
frame-2: complement (3447128..3447817)

Oligo evidence:
gggcgagtgcctgggaaaaagataaaacttacgacatcaccatattgcacaccaatgatcatcatggtca
tccgtaatggcgaattaacattggtcaactaccagttaatcccgatcaatttaaagaaaaaattgagaaa
ttaacattggtcaactaccagttaatcccgatcaatttaaagaaaaaattgagaaagctgatggcactcg
tgcaggtgatatcacttataaagatgtgctcaaggttcaaccctttggtaatacgctggtttacgttgat

Peptide evidence:

NA

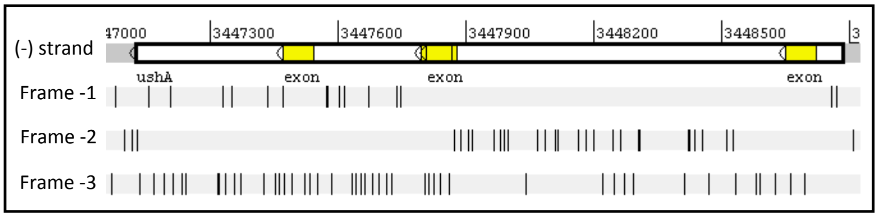

Current sequence (peptide evidence in red):
Frame-2: 84aa
MRFSLTTTLGALAVSLALAPGWASAWEKDKTYDITILHTNDHHGHFWQNEQGEYGLAAQKTVVDEIRKQVAAKGGSLLLLSGGD

Frame-1: 238aa
INTGVPESDLQDAEPDFRGMNLVGYDAMAIGNHEFDNPLSVLRQQEKWAKFPLLSANIYQKGTQQRLFKPYALFDKQGIKIAVLGLTTDDTPKVSSPENVVDIEFRVPAVEAKHVVEQLRKTEKPDIIIAATHMGHYDDGKHGSNAPGDVEMARSLPAGYLDMIVGGHSQNPVCMASENHRQVDYVPGSPCVPDRQNGTWIVQAHEWGKYVGRADFTFRNGELTLVNYQLIPINLKKK

Frame-2: 229aa
IEKADGTREHVFYTQEIAQDPTMLKLLTPFEERGKAQLGVRIGSVNGRLEGDRNKVRFEQTNMARLLLASQIERSGADFAVMSGGGVRDSIGAGDITYKDVLKVQPFGNTLVYVDMKGSEVEKYLAVVANKQPDSGAYAQFANVSLVADGQGVSNVKIQGEPLDPNKTYRMATLNFNALGGDGYPRIDNSPSYVNTGFIDAEVLKQYIEKHSPLDAEQYQLKGEIVYNQ

Error 95

Strain: Y. pseudotuberculosis PB1/+
Gene: YPTS_2214.5

Classification: novel gene
Boundaries of gene: 2440905..2441096

Oligo evidence:
tagtcggctgctgttcaacaaagcagcgatagataaagagggaaatacattgtatgcctatattttgtta

Peptide evidence:

LNKKISVITIGYIAISFSR

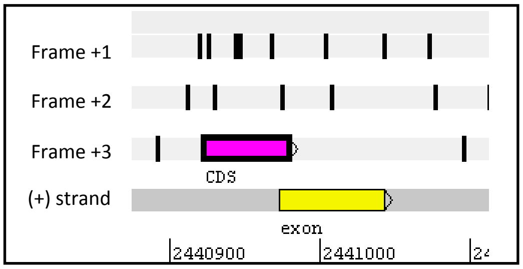

Proposed sequence (peptide evidence in red): 63aa
LLRYIGLNKKISVITIGYIAISFSRLLFNKAAIDKEGNTLYAYILLTRKIYVSYQLTSITYII

Error lacking threshold of evidence

Strain: Y. pestis pestoides F
Gene: YPDSF_0911.5

Classification: novel gene
Boundaries of gene: complement (1035790..1035981)

Oligo evidence:
tagtcggctgctgttcaacaaagcagcgatagataaagagggaaatacattgtatgcctatattttgtta

Peptide evidence:

NA

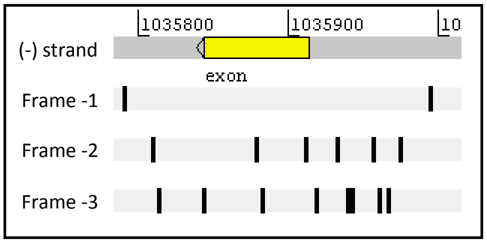

Current sequence (peptide evidence in red): 63aa
LLRYIGLNKKISVITISYIAISFSRLLFNKAAIDKEGNTLYAYILLTRKIYVSYQLTSITYII

Error lacking threshold of evidence

Strain: Y. pestis CO92
Gene: YPO2222.5

Classification: novel gene
Boundaries of gene: 2500177..2500368

Oligo evidence:
tagtcggctgctgttcaacaaagcagcgatagataaagagggaaatacattgtatgcctatattttgtta

Peptide evidence:

NA

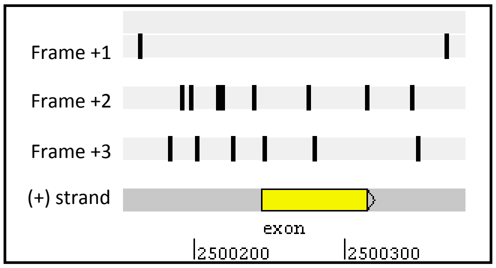

Current sequence (peptide evidence in red): 63aa
LLRYIGLNKKISVITISYIAISFSRLLFNKAAIDKEGNTLYAYILLTRKIYVSYQLTSITYII

Error 96

Strain: Y. pseudotuberculosis PB1/+
Gene: YPTS_0106.5

Classification: novel gene
Boundaries of gene: 111739..111900

Oligo evidence:

tgataatatgctttttttatcgagatatacatggagggatggtatgtgagaatattcttatacaatatgtc

Peptide evidence:

KIIIDNMLFYR

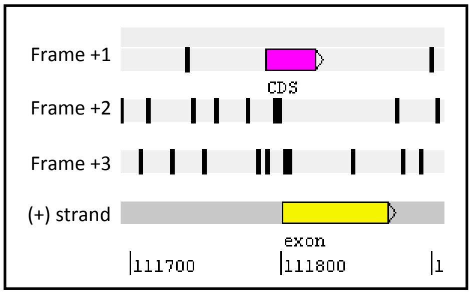

Proposed sequence (peptide evidence in red): 53aa
VISNHLKTGYIPIIRSSKIIIDNMLFYRDIHGGMVCENILIQYVDIAKTILEL

Error 79

Strain: Y. pestis pestoides F
Gene: YPDSF_3794.5

Classification: novel gene
Boundaries of gene: complement (4363721..4363882)

Oligo evidence:

tgataatatgctttttttatcgagatatacatggagggatggtatgtgagaatattcttatacaatatgtc

Peptide evidence:

KIIIDNMLFYR

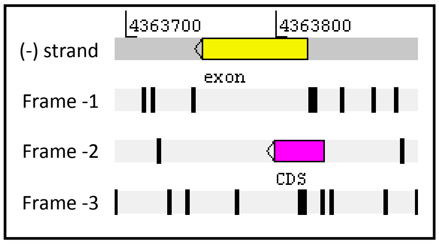

Proposed sequence (peptide evidence in red): 53aa
VISNHLKTGYIPIIRSSKIIIDNMLFYRDIHGGMVCENILIQYVDIAKTILEL

Strain: Y. pestis CO92
Gene: YPO0113

Classification: annotated as hypothetical protein
Boundaries of gene: 121254..121415

Oligo evidence:

tgataatatgctttttttatcgagatatacatggagggatggtatgtgagaatattcttatacaatatgtc

Peptide evidence:

NA

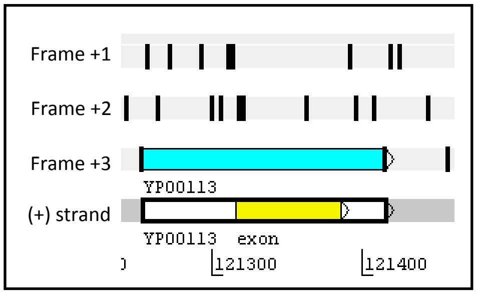

Current sequence (peptide evidence in red): 53aa
VISNHLKTGYIPIIRSSKIIIDNMLFYRDIHGGMVCENILIQYVDIAKTILEL
