# Supplementary figures and images for: Comparative Omics-Driven Genome Annotation Refinement: Application across Yersiniae
Source: PLoS One. 2012 Mar 27;7(3):e33903. doi: 10.1371/journal.pone.0033903 (PMC3313959; doi:10.1371/journal.pone.0033903)

## Yersinia pestis CO92

argD locus split by insertion

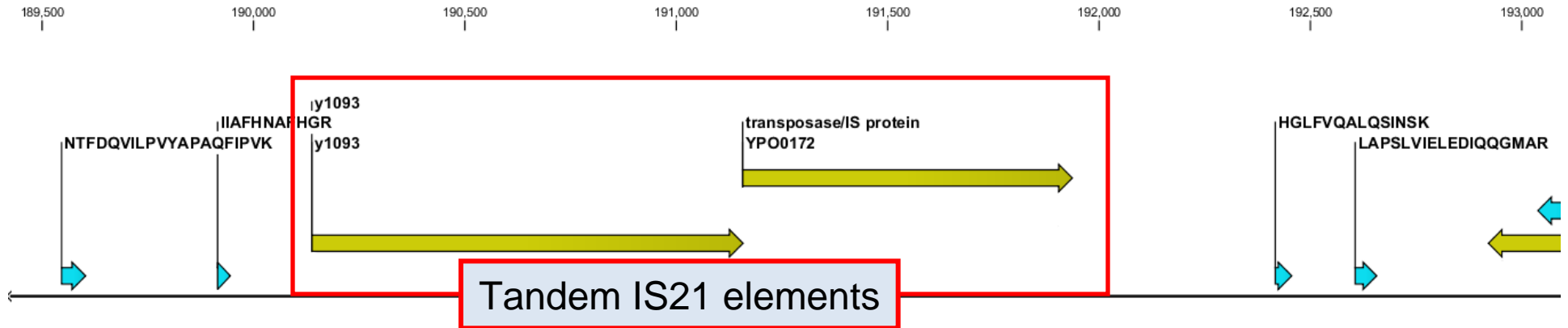

## Yersinia pestis Pestoides F

argD locus

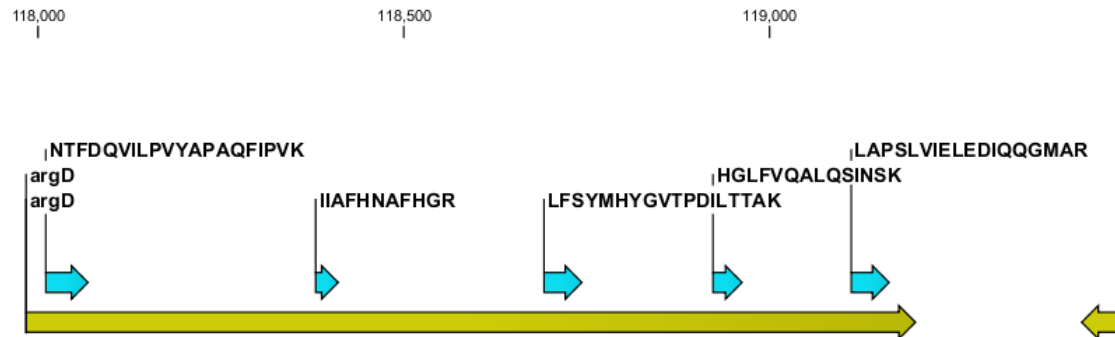

Supplement: File S3 — Peptide evidence related to the insertion-ablated pseudogene, argD . The regions encompassing argD loci from Yersinia pestis strains CO92 and Pestoides F are shown. Annotated open reading frames are colored in yellow. Detected peptide evidence is mapped by blue arrows and sequences are provided. (PDF) [file pone.0033903.s003.pdf]

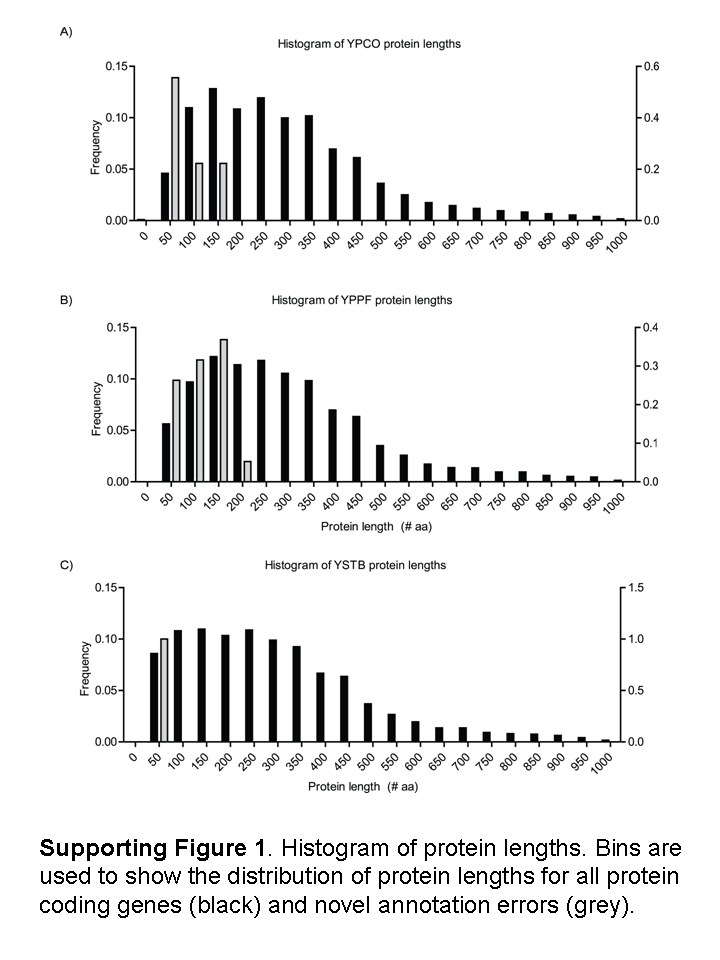

Supplement: Figure S1 — Protein length histograms. Bins are used to show the distribution of protein lengths for all protein coding genes (black) and novel annotation errors (grey). (TIF) [file pone.0033903.s007.tif]
