# Supplementary material for: Comparative Omics-Driven Genome Annotation Refinement: Application across Yersiniae
Source: PLoS One. 2012 Mar 27;7(3):e33903. doi: 10.1371/journal.pone.0033903 (PMC3313959; doi:10.1371/journal.pone.0033903)
Supplement: File S4 — Sanger sequencing. Methodology, primer sequences, primer maps, and sequence alignments are shown for YPO1195 and YPDSF_1005. (PDF) [file pone.0033903.s004.pdf]

Sequence Validation:

50 ng of CO92 genomic DNA was used as template for amplification of target PCR products. Phusion High Fidelity DNA Polymerase (New England BioLabs, Inc.) with the Phusion HF Reaction Buffer using gene specific primers in Table 1 at a final concentration of 500 nM were used for targeted amplification. The sample was amplified for 30 cycles of 98C for 30 seconds, 55C for 30 seconds and 72C for 60 seconds. The amplified products were purified over a QIAQuick column per manufacturer's recommendation (Qiagen). Purified products were sequenced on the Applied BioSystems 3730/XL instrument using manufacturer's standard operating procedures. Amplified and purified PCR products for YPDSF1005 and YPO1195 were sequenced on an Applied BioSystems 3730/XL per manufacturer's recommendation. Gene YPDSF1005 was sequenced in 4 independent lanes using primers YPDSF1005F1, YPDSF1005R1, YPDSF1005RSEQ2 and YPDSF1005FSEQ2. Gene YPO1195 was amplified using primers YPO1195F1 and YPO1195R1. The amplified material was sequenced by all primers in Table 1 to increase sequence resolution and coverage over the amplicon.

TABLE 1. PCR AND SEQUENCING PRIMERS

PRIMER NAME	SEQUENCE (5'-3')	GENE TARGET	COORDINATES (5'-3')
YPDSF1005F1	AGCAGATAAGGGCTACATAGG	YPDSF1005	1142260
YPDSF1005R1	CGATTGACCGAACTTGGCTAT	YPDSF1005	1141287
YPDSF1005FSEQ2	GCTATCAGAAACAACGATT	YPDSF1005	1141671
YPDSF1005RSEQ2	TTCCTTGAGCATATCGTTCC	YPDSF1005	1141834
YPO1195F1	TAGCCCGTAGAACCACCAACG	YPO1195	1345419
YPO1195R1	CCATGAAACGAATAGCACCA	YPO1195	1346505
YPO1195FSEQ1	ATGGCACTGTTCCTAACTC	YPO1195	1345498
YPO1195RSEQ1	TTAGTTTTTAATAAATCCGTTATC	YPO1195	1346430
YPO1195FSEQ2	GCTCCAATCATCCGTGAAGT	YPO1195	1346245
YPO1195RSEQ2	AAGCTTATAGTGCCACACTGGTC	YPO1195	1346475
YPO1195FSEQ3	ATTGGTATGGCTCTCTCC	YPO1195	1345860
YPO1195RSEQ3	CAATCTCACCGGCAGTAATCG	YPO1195	1345738
YPO1195FSEQ4	TGAAAGCTCACCCGAACATC	YPO1195	1346270
YPO1195RSEQ4	CATTGATCCACTAACCTAAATC	YPO1195	1345975

Primer Maps

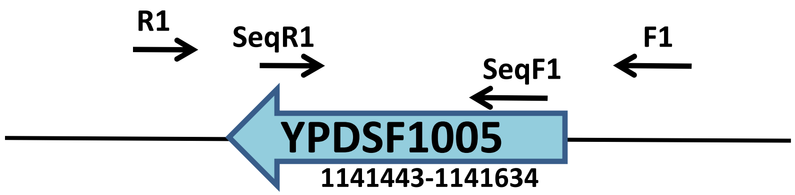

R1
SeqR1
SeqF1
F1
YPDSF1005
1141443-1141634

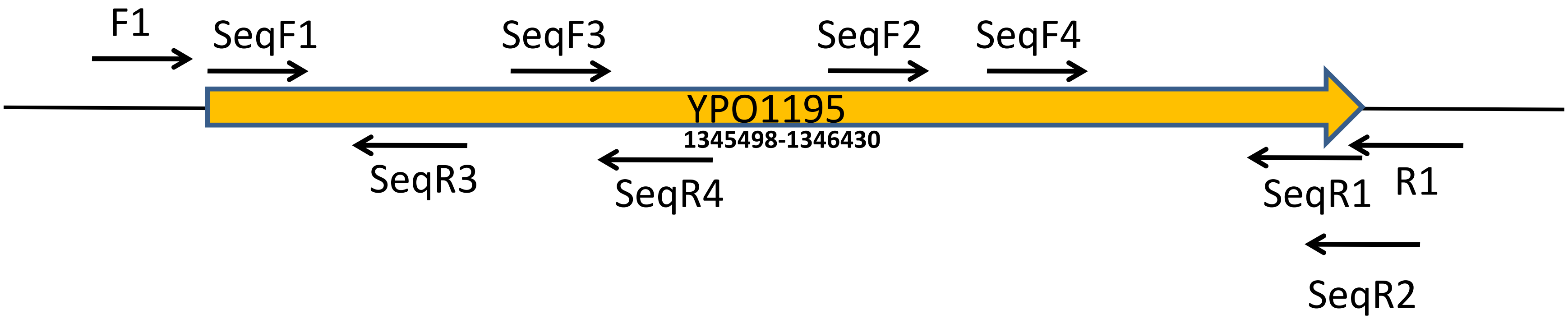

F1
SeqF1
SeqF3
SeqF2
SeqF4
YPO1195
1345498-1346430
SeqR3
SeqR4
SeqR1
R1
SeqR2

YPDSF_1005
Query: Sanger sequence of sample
Subject: Y. pestis Pestoides F genome sequence

```
Query  1        ATGAACAAATATATAGCGAACGAGAAAATGCCATTACTTATCCCCGCGGCTGGCTTTGAT  60
                ||||||||||||||||||||||||||||||||||||||||||||||||||||||||||||
Sbjct  1142086  ATGAACAAATATATAGCGAACGAGAAAATGCCATTACTTATCCCCGCGGCTGGCTTTGAT  1142027

Query  61       GCCAAACTAAGCATGACAGAGAAAAACATAGAGCATACTGAAGCCTTTATGGCATCAGGC  120
                ||||||||||||||||||||||||||||||||||||||||||||||||||||||||||||
Sbjct  1142026  GCCAAACTAAGCATGACAGAGAAAAACATAGAGCATACTGAAGCCTTTATGGCATCAGGC  1141967

Query  121      GTGAAAGAATTCTCTTTGCCTGGATTTGTAACTCCTCACGGGTATCGTCTACTTGTTTCT  180
                ||||||||||||||||||||||||||||||||||||||||||||||||||||||||||||
Sbjct  1141966  GTGAAAGAATTCTCTTTGCCTGGATTTGTAACTCCTCACGGGTATCGTCTACTTGTTTCT  1141907

Query  181      GTAAACGGGGAGCATTACCGCCTTGTTACAGACGCAGCTATACCGGAAACAGTATATGCG  240
                ||||||||||||||||||||||||||||||||||||||||||||||||||||||||||||
Sbjct  1141906  GTAAACGGGGAGCATTACCGCCTTGTTACAGACGCAGCTATACCGGAAACAGTATATGCG  1141847

Query  241      GTGAAACTGGCTTTCCTTGAGCATATCGTTCCTACACGTAAAACATGTACTCAGATTTTA  300
                ||||||||||||||||||||||||||||||||||||||||||||||||||||||||||||
Sbjct  1141846  GTGAAACTGGCTTTCCTTGAGCATATCGTTCCTACACGTAAAACATGTACTCAGATTTTA  1141787

Query  301      GTCTGGCGTACTGTTCAACCACAGCATGATAGTGCAGTCCATGGTTTGCCTCAGGAGTTT  360
                ||||||||||||||||||||||||||||||||||||||||||||||||||||||||||||
Sbjct  1141786  GTCTGGCGTACTGTTCAACCACAGCATGATAGTGCAGTCCATGGTTTGCCTCAGGAGTTT  1141727

Query  361      TTTCGCCACTTCCTGACTCAGTACTCACTCAGTACTCAATCGTTGTTTCTGATAGCGAAC  420
                ||||||||||||||||||||||||||||||||||||||||||||||||||||||||||||
Sbjct  1141726  TTTCGCCACTTCCTGACTCAGTACTCACTCAGTACTCAATCGTTGTTTCTGATAGCGAAC  1141667

Query  421      AGACTAATGAAGGTCGTCGTTTCTGGGAGCGAATGATTGCATGGGCTATTCAGGCTGAAG  480
                ||||||||||||||||||||||||||||||||||||||||||||||||||||||||||||
Sbjct  1141666  AGACTAATGAAGGTCGTCGTTTCTGGGAGCGAATGATTGCATGGGCTATTCAGGCTGAAG  1141607

Query  481      GGCATTATGTATATGTGTCGAATGGCTCAGAGGAAGATCGCCCACTGACTTTTATGACTA  540
                ||||||||||||||||||||||||||||||||||||||||||||||||||||||||||||
Sbjct  1141606  GGCATTATGTATATGTGTCGAATGGCTCAGAGGAAGATCGCCCACTGACTTTTATGACTA  1141547

Query  541      ACTGGGATGATTTTTATGGGACATGGGCTGATTTTTGTTGGGGTGATGACAGAGATTGCC  600
                ||||||||||||||||||||||||||||||||||||||||||||||||||||||||||||
Sbjct  1141546  ACTGGGATGATTTTTATGGGACATGGGCTGATTTTTGTTGGGGTGATGACAGAGATTGCC  1141487

Query  601      ATTGCCATAGACTATTCGTGATCAGTACAGAACAATTGCACTAA  644
                ||||||||||||||||||||||||||||||||||||||||||||
Sbjct  1141486  ATTGCCATAGACTATTCGTGATCAGTACAGAACAATTGCACTAA  1141443
```

YPO1195
Query: Sanger sequence of sample
Subject: Y. pestis CO92 genome sequence

```
Query  1        GTGGCTGCTATCATGCTGTCTGCATCGAGTGGTATATCGCACGCGGCAGATAACGTCCGT  60
                ||||||||||||||||||||||||||||||||||||||||||||||||||||||||||||
Sbjct  1345534  GTGGCTGCTATCATGCTGTCTGCATCGAGTGGTATATCGCACGCGGCAGATAACGTCCGT  1345593

Query  61       GTAGGCTCTAAAATTGATACCGAAGGCTCCTTGCTGGGCAATATTATTGTGCAGGTTTTG  120
                ||||||||||||||||||||||||||||||||||||||||||||||||||||||||||||
Sbjct  1345594  GTAGGCTCTAAAATTGATACCGAAGGCTCCTTGCTGGGCAATATTATTGTGCAGGTTTTG  1345653

Query  121      GAGGCTAACGGGATTAAAACCACCAATAAATCCCAACTGGGAGCAACCAAAGTCGTACGT  180
                ||||||||||||||||||||||||||||||||||||||||||||||||||||||||||||
Sbjct  1345654  GAGGCTAACGGGATTAAAACCACCAATAAATCCCAACTGGGAGCAACCAAAGTCGTACGT  1345713

Query  181      GGGGCGATTACTGCCGGTGAGATTGATATTTATCCTGAATATACGGGGAATGGGGCTTTC  240
                ||||||||||||||||||||||||||||||||||||||||||||||||||||||||||||
Sbjct  1345714  GGGGCGATTACTGCCGGTGAGATTGATATTTATCCTGAATATACGGGGAATGGGGCTTTC  1345773

Query  241      TTCTTTTCTGATGAACAAGATCCTGCCTGGAAGAGCGCCAAAGCGGGCTATGAGAAAGTA  300
                ||||||||||||||||||||||||||||||||||||||||||||||||||||||||||||
Sbjct  1345774  TTCTTTTCTGATGAACAAGATCCTGCCTGGAAGAGCGCCAAAGCGGGCTATGAGAAAGTA  1345833

Query  301      AAAGCACTGGATTACGaaaaaaaTAAATTGGTATGGCTCTCTCCCGCGCCAGCGAACAAT  360
                ||||||||||||||||||||||||||||||||||||||||||||||||||||||||||||
Sbjct  1345834  AAAGCACTGGATTACGAAAAAAATAAATTGGTATGGCTCTCTCCCGCGCCAGCGAACAAT  1345893

Query  361      ACCTGGACCATTGCTGTACGAAAAGACTTAGCCACGGCCAATAATTTGCGAACGCTGGAT  420
                ||||||||||||||||||||||||||||||||||||||||||||||||||||||||||||
Sbjct  1345894  ACCTGGACCATTGCTGTACGAAAAGACTTAGCCACGGCCAATAATTTGCGAACGCTGGAT  1345953

Query  421      GATTTAGGTTAGTGGATCAATGGCGGAGGTCAGTTTAAATTAGCCGCCTCAGCCGAGTTT  480
                ||||||||||||||||||||||||||||||||||||||||||||||||||||||||||||
Sbjct  1345954  GATTTAGGTTAGTGGATCAATGGCGGAGGTCAGTTTAAATTAGCCGCCTCAGCCGAGTTT  1346013

Query  481      ATCGAGCGGCCTGATGCGTTACCCGCTTTCCAACAGGCGTATGGTTTTACGCTGAATCAA  540
                ||||||||||||||||||||||||||||||||||||||||||||||||||||||||||||
Sbjct  1346014  ATCGAGCGGCCTGATGCGTTACCCGCTTTCCAACAGGCGTATGGTTTTACGCTGAATCAA  1346073

Query  541      GATCAACTACTGTCCCTGGCTGGGGGAGATACCGCTGTGACGATTAAAGCGGCAGCAGA-  599
                |||||||||||||||||||||||||||||||||||||||||||||||||||||||||||
Sbjct  1346074  GATCAACTACTGTCCCTGGCTGGGGGAGATACCGCTGTGACGATTAAAGCGGCAGCAGAA  1346133

Query  600      CAGATCTCAGGAGTGAATGCCGCCATGGCTTATGGTACCGATGGTCCTGTAGCGGCACTG  659
                ||||||||||||||||||||||||||||||||||||||||||||||||||||||||||||
Sbjct  1346134  CAGATCTCAGGAGTGAATGCCGCCATGGCTTATGGTACCGATGGTCCTGTAGCGGCACTG  1346193

Query  660      GGTTTACAGACTCTGGAAGATACTAAAGGCGTGCAGCCGATTTATGCGCCAGCTCCAATC  719
                ||||||||||||||||||||||||||||||||||||||||||||||||||||||||||||
Sbjct  1346194  GGTTTACAGACTCTGGAAGATACTAAAGGCGTGCAGCCGATTTATGCGCCAGCTCCAATC  1346253

Query  720      ATCCGTGAAGTTACTCTGAAAGCTCACCCGAACATCCCGGCTTTGCTTAATCCGGTATTC  779
                ||||||||||||||||||||||||||||||||||||||||||||||||||||||||||||
Sbjct  1346254  ATCCGTGAAGTTACTCTGAAAGCTCACCCGAACATCCCGGCTTTGCTTAATCCGGTATTC  1346313

Query  780      GCCACACTGGATGGCCCAACGTTACAAAAGCTGAATGCACGTATTGCGGTGGA  832
                |||||||||||||||||||||||||||||||||||||||||||||||||||||
Sbjct  1346314  GCCACACTGGATGGCCCAACGTTACAAAAGCTGAATGCACGTATTGCGGTGGA  1346366
```
