# Supplementary material for: Comparative Omics-Driven Genome Annotation Refinement: Application across Yersiniae
Source: PLoS One. 2012 Mar 27;7(3):e33903. doi: 10.1371/journal.pone.0033903 (PMC3313959; doi:10.1371/journal.pone.0033903)
Supplement: File S5 — Singlet peptide validations. Three errors were supported by singlet identifications. Annotated spectra are provided for these peptides. (PDF) [file pone.0033903.s005.pdf]

Error 33: YPO3723
ORF location (reading frame : base position): COchrom1:4167022-4167372

Peptide: AATQNTPAATGQVQSLTR
MS-GF p-value: 4.51E-19

Scan	Charge	Xcorr	Sp	DeltaCn	ORF	Peptide
2464	2	3.8359	437.2	0.4123	COchrom1:4167022-4167372	K.AATQNTPAATGQVQSLTR.G

COchrom1_4167022_4167372 #2464 RT: 13.49 AV: 1 NL: 1.78E5
T: ITMS + c NSI d Full ms2 908.66@cid35.

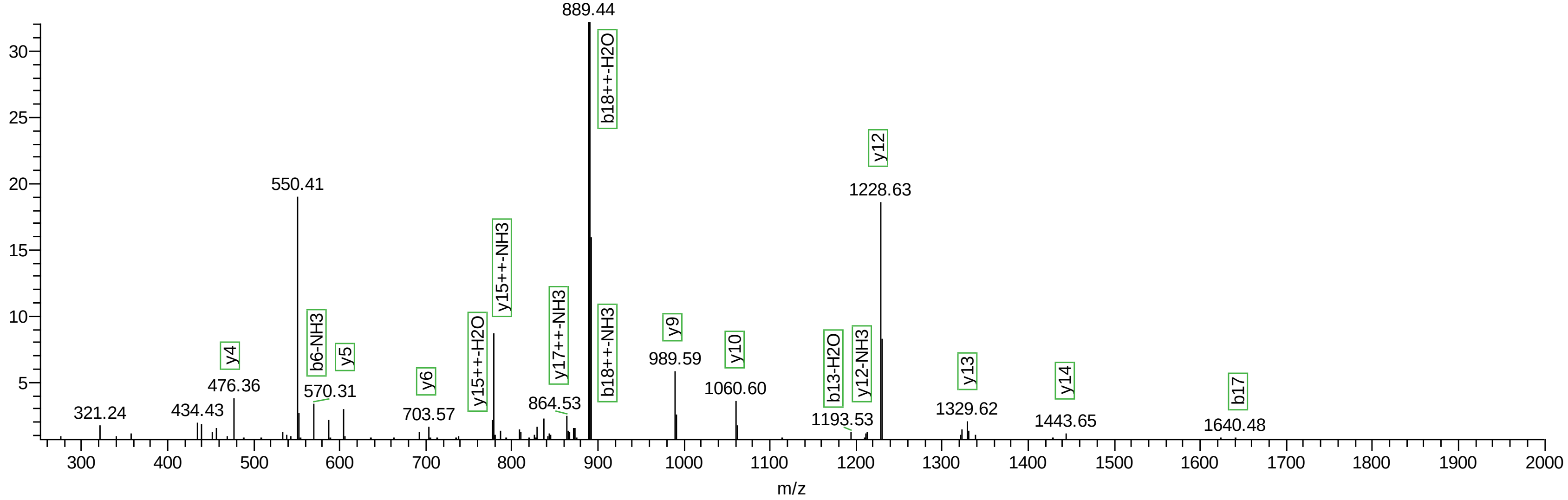

Fragment Ladder (cyan shows ions that match, yellow shows ions with shoulders) :

#	Immon.	b	b++	b++-H2O	b++-NH3	b-H2O	b-NH3	Seq.	y	y++	y++-H2O	y++-NH3	y-H2O	y-NH3	#
1	44.05							A	1814.94	907.97	898.97	899.46	1796.92	1797.91	18
2	44.05	143.08						A	1743.90	872.45	863.45	863.94	1725.89	1726.87	17
3	74.06	244.13				226.12		T	1672.86	836.93	827.93	828.42	1654.85	1655.83	16
4	101.07	372.19				354.18	355.16	Q	1571.81	786.41	777.40	777.90	1553.80	1554.79	15
5	87.06	486.23				468.22	469.20	N	1443.75	722.38	713.38	713.87	1425.74	1426.73	14
6	74.06	587.28				569.27	570.25	T	1329.71	665.36	656.35	656.85	1311.70	1312.69	13
7	70.07	684.33				666.32	667.31	P	1228.66	614.84	605.83	606.32	1210.65	1211.64	12
8	44.05	755.37				737.36	738.34	A	1131.61	566.31	557.30	557.80	1113.60	1114.58	11
9	44.05	826.41	413.71	404.70	405.19	808.40	809.38	A	1060.57	530.79	521.79	522.28	1042.56	1043.55	10
10	74.06	927.45	464.23	455.23	455.72	909.44	910.43	T	989.54	495.27	486.27	486.76	971.53	972.51	9
11	30.03	984.48	492.74	483.74	484.23	966.46	967.45	G	888.49	444.75	435.74	436.23	870.48	871.46	8
12	101.07	1112.53	556.77	547.77	548.26	1094.52	1095.51	Q	831.47	416.24	407.23	407.72	813.46	814.44	7
13	72.08	1211.60	606.30	597.30	597.79	1193.59	1194.58	V	703.41				685.40	686.38	6
14	101.07	1339.66	670.33	661.33	661.82	1321.65	1322.63	Q	604.34				586.33	587.31	5
15	60.04	1426.69	713.85	704.84	705.34	1408.68	1409.67	S	476.28				458.27	459.26	4
16	86.10	1539.78	770.39	761.39	761.88	1521.77	1522.75	L	389.25				371.24	372.22	3
17	74.06	1640.82	820.92	811.91	812.40	1622.81	1623.80	T	276.17				258.16	259.14	2
18	129.11							R	175.12					158.09	1

Error 47: YPO2654.5
ORF location (reading frame : base position): COchrom6:2982147-2982434

Peptide: DAAAFNLHLNSQHK
MS-GF p-value: 9.38E-17

Scan	Charge	Xcorr	Sp	DeltaCn	ORF	Peptide
3724	2	4.2457	827.5	0.4156	COchrom6:2982147-2982434	K.DAAAFNLHLNSQHK.N

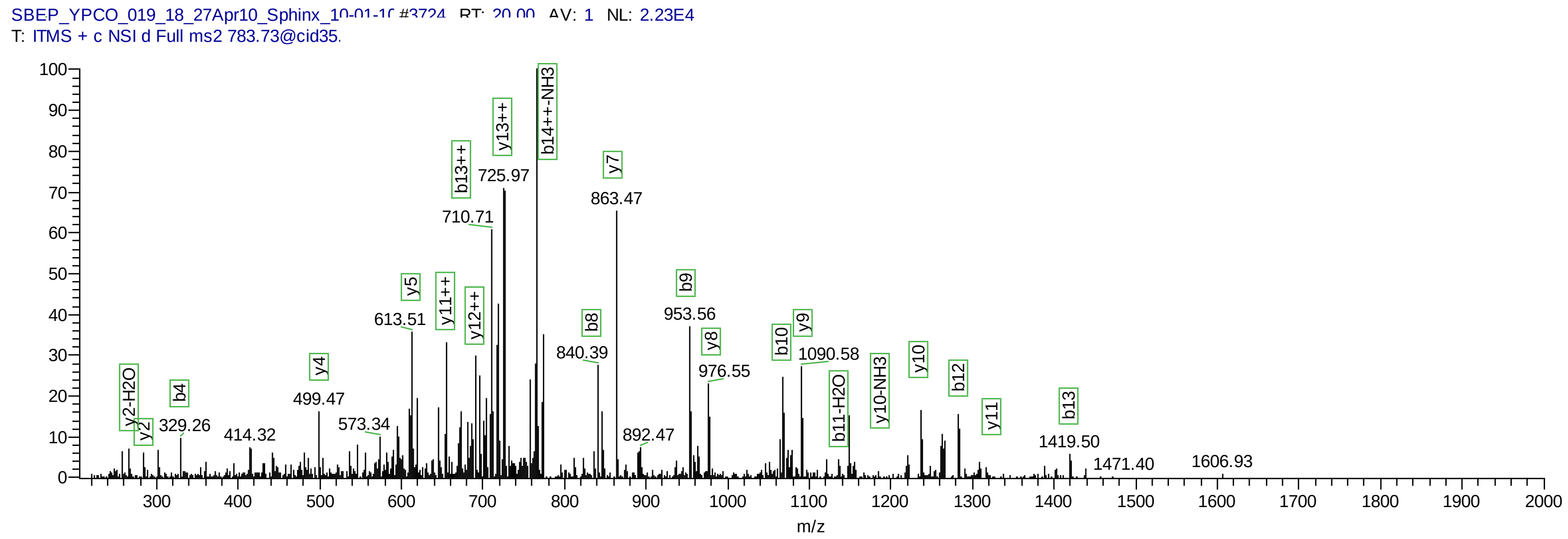

Fragment Ladder (cyan shows ions that match, yellow shows ions with shoulders) :

#	Immon.	b	b++	b++-H2O	b++-NH3	b-H2O	b-NH3	Seq.	y	y++	y++-H2O	y++-NH3	y-H2O	y-NH3	#
1	88.04							D	1565.78	783.39	774.39	774.88	1547.77	1548.76	14
2	44.05	187.07				169.06		A	1450.75	725.88	716.88	717.37	1432.74	1433.73	13
3	44.05	258.11				240.10		A	1379.72	690.36	681.36	681.85	1361.71	1362.69	12
4	44.05	329.15				311.14		A	1308.68	654.84	645.84	646.33	1290.67	1291.65	11
5	120.08	476.21				458.20		F	1237.64	619.33	610.32	610.81	1219.63	1220.62	10
6	87.06	590.26				572.25	573.23	N	1090.57	545.79	536.79	537.28	1072.56	1073.55	9
7	86.10	703.34				685.33	686.31	L	976.53	488.77	479.76	480.26	958.52	959.51	8
8	110.07	840.40	420.70	411.70	412.19	822.39	823.37	H	863.45	432.23	423.22	423.71	845.44	846.42	7
9	86.10	953.48	477.25	468.24	468.73	935.47	936.46	L	726.39				708.38	709.36	6
10	87.06	1067.53	534.27	525.26	525.75	1049.52	1050.50	N	613.30				595.29	596.28	5
11	60.04	1154.56	577.78	568.78	569.27	1136.55	1137.53	S	499.26				481.25	482.24	4
12	101.07	1282.62	641.81	632.81	633.30	1264.61	1265.59	Q	412.23					395.20	3
13	110.07	1419.68	710.34	701.34	701.83	1401.67	1402.65	H	284.17					267.14	2
14	101.11							K	147.11					130.09	1

Error 61: YPDSF_2565
ORF location (reading frame : base position): PFchrom4:2913628-2914533

Peptide: FPSTVNAAETVAK
MS-GF p-value: 9.84E-14

Scan	Charge	Xcorr	Sp	DeltaCn	ORF	Peptide
2998	2	2.9174	579.1	0.2803	PFchrom4:2913628-2914533	K.FPSTVNAAETVAK.T

SBEP_YPPF_011_13_20Apr10_Sphinx_10-01-07 #2998 RT: 15.74 AV: 1 NL: 6.25E4
T: ITMS + c NSI d Full ms2 669.57@cid35.

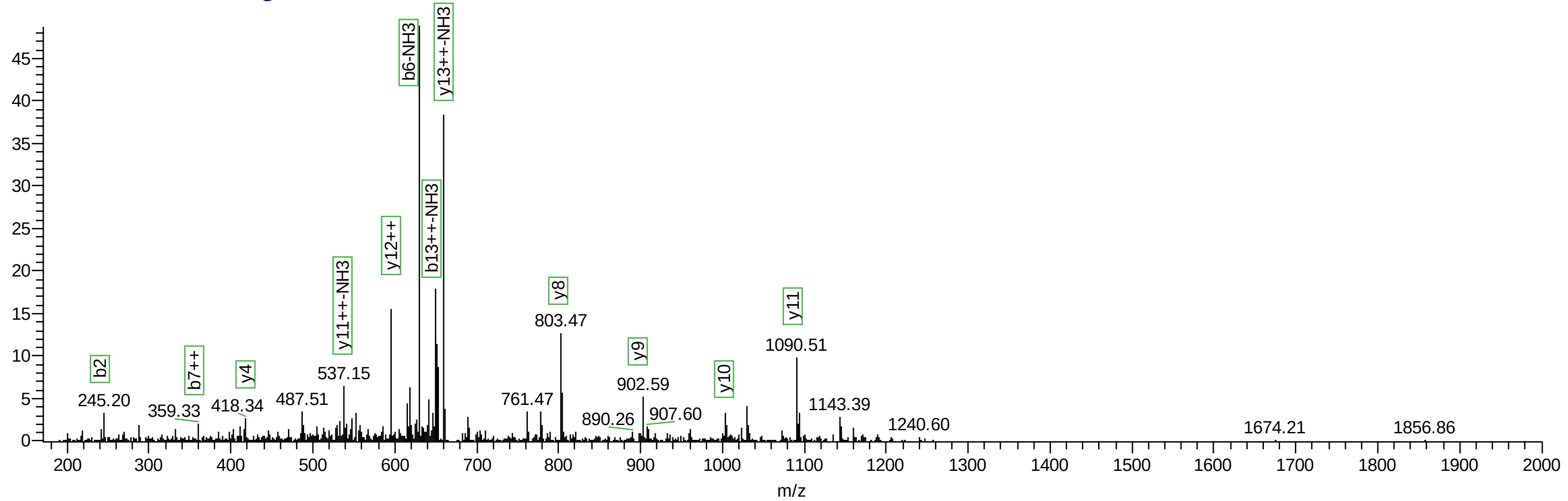

Fragment Ladder (cyan shows ions that match, yellow shows ions with shoulders) :

#	Immon.	b	b++	b++-H2O	b++-NH3	b-H2O	b-NH3	Seq.	y	y++	y++-H2O	y++-NH3	y-H2O	y-NH3	#
1	120.08							F	1334.70	667.85	658.85	659.34	1316.68	1317.67	13
2	70.07	245.13						P	1187.63	594.32	585.31	585.80	1169.62	1170.60	12
3	60.04	332.16				314.15		S	1090.57	545.79	536.79	537.28	1072.56	1073.55	11
4	74.06	433.21				415.20		T	1003.54	502.27	493.27	493.76	985.53	986.51	10
5	72.08	532.28				514.27		V	902.49	451.75	442.75	443.24	884.48	885.47	9
6	87.06	646.32				628.31	629.29	N	803.43	402.22	393.21	393.70	785.41	786.40	8
7	44.05	717.36				699.35	700.33	A	689.38				671.37	672.36	7
8	44.05	788.39				770.38	771.37	A	618.35				600.33	601.32	6
9	102.06	917.44	459.22	450.22	450.71	899.43	900.41	E	547.31				529.30	530.28	5
10	74.06	1018.48	509.75	500.74	501.23	1000.47	1001.46	T	418.27				400.25	401.24	4
11	72.08	1117.55	559.28	550.27	550.77	1099.54	1100.53	V	317.22					300.19	3
12	44.05	1188.59	594.80	585.79	586.29	1170.58	1171.56	A	218.15					201.12	2
13	101.11							K	147.11					130.09	1
